# N‐Heterocyclic Carbene Acyl Anion Organocatalysis by Ball‐Milling

**DOI:** 10.1002/cssc.201902346

**Published:** 2019-11-27

**Authors:** William I. Nicholson, Alex C. Seastram, Saqib A. Iqbal, Benjamin G. Reed‐Berendt, Louis C. Morrill, Duncan L. Browne

**Affiliations:** ^1^ Cardiff Catalysis Institute School of Chemistry Cardiff University Park Place Cardiff CF10 3AT United Kingdom

**Keywords:** acyl anion, ball milling, mechanochemistry, N-heterocyclic carbenes, organocatalysis

## Abstract

The ability to conduct N‐heterocyclic carbene‐catalysed acyl anion chemistry under ball‐milling conditions is reported for the first time. This process has been exemplified through applications to intermolecular‐benzoin, intramolecular‐benzoin, intermolecular‐Stetter and intramolecular‐Stetter reactions including asymmetric examples and demonstrates that this mode of mechanistically complex organocatalytic reaction can operate under solvent‐minimised conditions.

## Graphical abstract

**Having a ball**: The ability to conduct mechanistically complex N‐heterocyclic carbene‐catalysed acyl anion chemistry under ball‐milling conditions is reported for the first time. This process is exemplified through applications to intermolecular‐benzoin, intramolecular‐benzoin, intermolecular‐Stetter and intramolecular‐Stetter reactions including asymmetric examples.

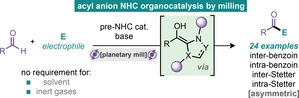

Mechanochemistry is characterised by the input of mechanical energy into chemical bonds to initiate reactivity of those bonds.^1^ Chemical reactions brought about by ball‐milling constitute an area of mechanochemistry. That a reaction takes place under ball‐milling conditions does, however, not necessarily mean that the process is mechanically driven. Indeed, ball‐milling reactions also typically feature solvent‐free or solvent‐minimised, high‐concentration and occasionally high‐instantaneous/bulk‐temperature conditions. Many of these factors are inextricably linked and may never be fully delineated. Nonetheless, the fields of mechanochemistry and ball‐milling, in combination with reactive extrusion, are capable of delivering a more sustainable approach to some aspects of chemical synthesis and chemical manufacturing.^2^ Given the complex inter‐related nature of parameters, one approach to gain a better insight into these techniques is through attrition, that is, gaining many experimental data points and building a picture of understanding as a whole. In recent years the community in this area has been building towards this vision.^3^ In several instances there have emerged trends and conceptual frameworks, and in others there is simply a translation to a solvent‐minimised process. However, perhaps the most exciting aspect of this approach is the increased opportunity for serendipitous discoveries by exploring this unchartered chemical reactor environment.^4^ As part of this process the field has recently been moving towards assessing the possibility of running complex catalytic reactions and enantioselective processes under milling conditions.^5^

The area of organocatalysis is one such area where the proposed reaction pathways require several discrete steps and enantioselectivity rests on the organisation of complex transition states. Of the many areas of organocatalysis (some of which are shown in Figure 1 A), only secondary amine systems have been well studied under milling conditions, with pioneering contributions from Bolm and co‐workers.^6^ It has been established that several reaction manifolds accessible by secondary amine organocatalysis in solution can also operate under milling conditions.^7^ Given the wealth of transformations and activation modes we were intrigued by the prospect of conducting nucleophilic heterocyclic carbene (NHC) catalysis under milling conditions. NHC organocatalysis has been rapidly established as a key area for catalyst‐mediated synthesis, with many activation modes established for a wide range of substrates (Figure 1 B).^8^ The first established activation mode of acyl anions stemmed from the pioneering work of Breslow on thiamine‐catalysed reactions and has led to numerous examples across a range of carbonyl functional groups.^9^ These include benzoin, Stetter and hydroacylation reactions, with demonstration of homo‐, cross‐, inter‐ and intramolecular examples. Herein we report the first results of combining this NHC activation mode with ball‐milling (Figure 1 C).^10^

**Figure 1:**
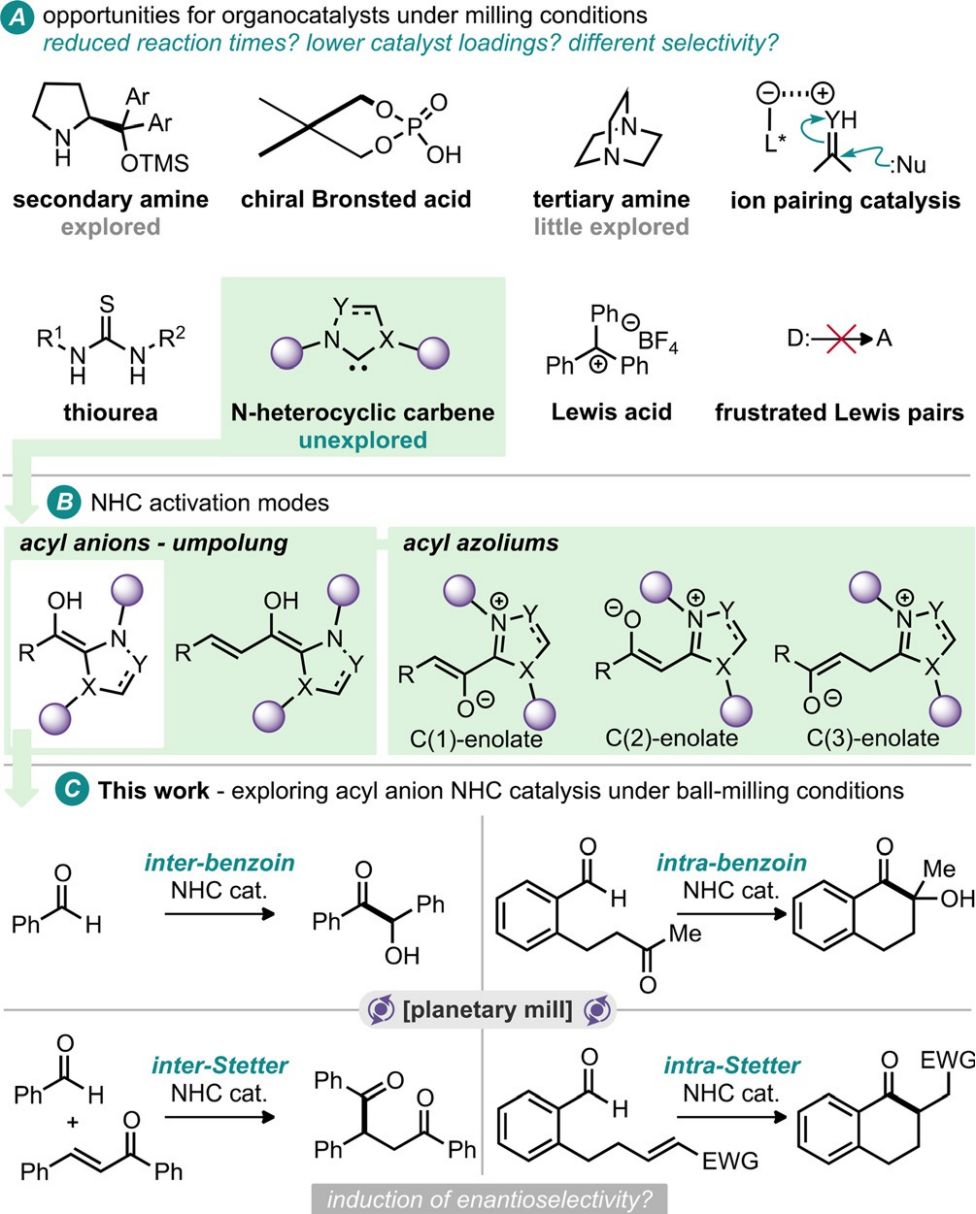
(a) Some areas of organocatalysis; (b) example activation modes of NHC catalysis; (c) this work: NHC acyl anion organocatalysis under ball‐milling conditions.

Our initial investigations commenced with the intermolecular homo‐benzoin reaction of 4‐chlorobenzaldehyde under planetary milling conditions. A range of ten NHC precatalysts based on thiazolium, imidazolium and triazolium heterocycles were screened, along with five bases [K_3_PO_4_, K_2_CO_3_, Cs_2_CO_3_, 1,8‐diazabicyclo[5.4.0]undec‐7‐ene (DBU) and Et_3_N] and three grinding auxiliaries.^11^ Pleasingly, it was found that the pentafluorophenyl bearing triazolium tetrafluoroborate NHC precatalyst **8** was the most effective at 10 mol % loading.

Combining the 4‐chlorobenzaldehyde with the precatalyst, Cs_2_CO_3_, sand (as a grinding auxiliary) and grinding in a planetary mill at 300 rpm for 15 min furnished the homo‐benzoin product **11** in 72 % isolated yield (Scheme 1, conditions A). However, extending these conditions to a small range of substrates did not return positive outcomes in every case. The literature concerning NHC‐catalysed benzoin reactions demonstrates that several of the reaction steps can be equilibrium processes, in which the reversibility of individual steps is highly dependent on the catalyst and substrate combinations.^12^ We hypothesised that the presence of liquids/solvents may help to stabilise or drive reactions forward, and, in the solid state, crystal lattice enthalpies may also play a critical role in determining the position of equilibrium for reactions featuring solid products and/or starting materials (notably, 4‐chlorobenzaldehyde is a solid, and other benzaldehyde derivatives are liquids). With this in mind, we screened the addition of 100 μL of several LAG (Liquid‐Assisted Grinding) materials, including EtOAc, tetrahydrofuran (THF), dichloromethane (DCM), hexafluoroisopropanol (HFIP), isopropanol (IPA), EtOH, dimethylformamide (DMF), MeCN, dimethylacetamide (DMA) and dimethyl sulfoxide (DMSO). It was found that addition of IPA was optimal and permitted 76 % isolated yield of **14** (in the case of benzaldehyde) and 63 % yield of **12** (in the case of 4‐tolaldehyde). With these conditions in hand, a total of six aldehydes were then assessed under “no LAG” and “IPA LAG” (100 μL) conditions (Scheme 1, inter‐benzoin). It appears that milling with LAG gives the most robust conditions, that is, those that permit the greatest chances of success under this milling protocol. As common in many solvent‐based approaches, it too was found that the reaction is highly dependent on the purity of the aldehyde introduced into the reaction; trace carboxylic acid appears to have a disproportionately negative effect on the outcome of the reaction. Pleasingly, the “no LAG” conditions could also be directly applied to the intra‐cross‐benzoin reaction of tethered ketone–aldehyde substrates to yield α‐hydroxychromanone products in good yields (Scheme 1, intra‐benzoin). With confirmation in hand of carbene generation and its engagement in catalysis through acyl anion activation, our attention turned to demonstrating this reactivity also in the case of the Stetter reaction. Application of previously optimised conditions A to a model inter‐Stetter reaction featuring 4‐chlorobenzaldehyde and chalcone did not return favourable yields of the desired product. However, conditions A (5 mol % precatalyst) were applicable to a range of intramolecular Stetter reactions to furnish the corresponding chromanones and 3‐oxo‐2,3‐dihydrobenzofurans in good‐to‐excellent yields (Scheme 2, intra‐Stetter 6,6 and intra‐Stetter 6,5). Indeed, further reaction screening was required to deliver the intermolecular Stetter reaction under milling conditions. Again, screening a range of precatalysts, bases, grinding auxiliaries, LAGs and milling speeds delivered optimal results. We were pleased to find that the archetypal thiazolium catalysts were highly effective under these conditions with K_3_PO_4_ serving as base (and likely the grinding auxiliary). Thiazolium pre‐NHC **1** was optimal and afforded conditions B (Scheme 2), requiring 3 h milling at 700 rpm. With these conditions in hand, a sample of nine intermolecular Stetter reactions was explored. A combination of three aldehydes with three different chalcone derivatives afforded moderate‐to‐good yields in all nine cases. Attention was then turned to exploring if enantioselectivity could be imparted to this reaction manifold under milling conditions. Conditions A were chosen to be explored, and the pentafluorophenyl bearing triazolium pre‐NHC **8** was switched for the privileged aminoindane‐based triazolium pre‐NHC **33**, reported by Kerr and Rovis.^13^ Three of the reaction modes were explored, including intra‐benzoin, inter‐benzoin and both the 6,6‐ and 6,5‐intra‐Stetter reactions. All experiments were run with 10 mol % catalyst loading, and preliminary results show that it is indeed possible to transmit enantioselectivity under these conditions (Scheme 3). For the intra‐benzoin reaction, it was found that addition of IPA as LAG permitted the reaction to return increased yield and slightly increased enantiomeric excess (24 % yield, 72 % *ee* without LAG vs. 52 % yield and 82 % *ee* with LAG). The 6,6‐intra‐molecular Stetter reaction returned the highest *ee* of 92 % (with an isolated yield of 96 %).

**Scheme 1:**
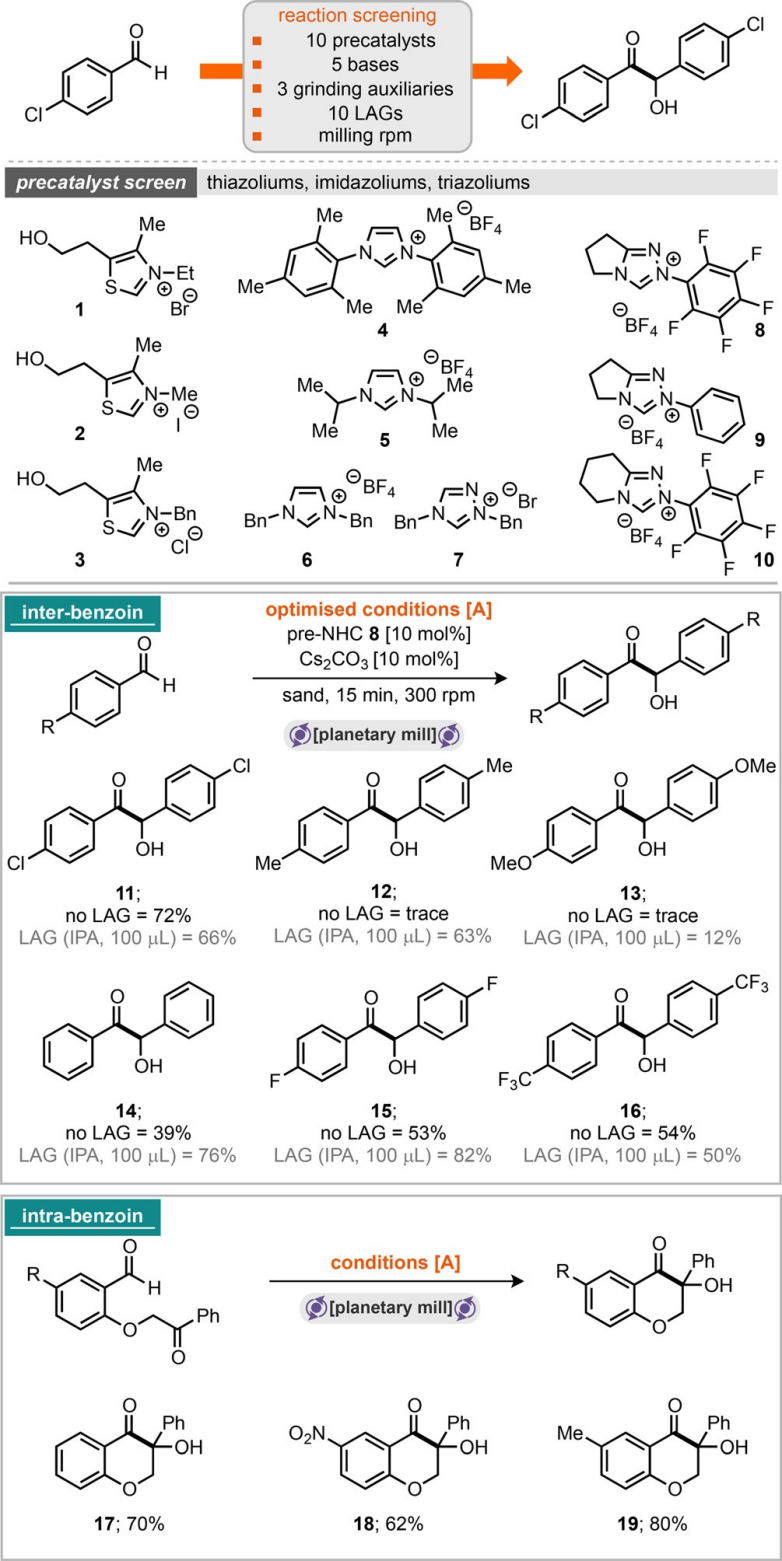
NHC‐catalysed benzoin reaction under milling conditions.

**Scheme 2:**
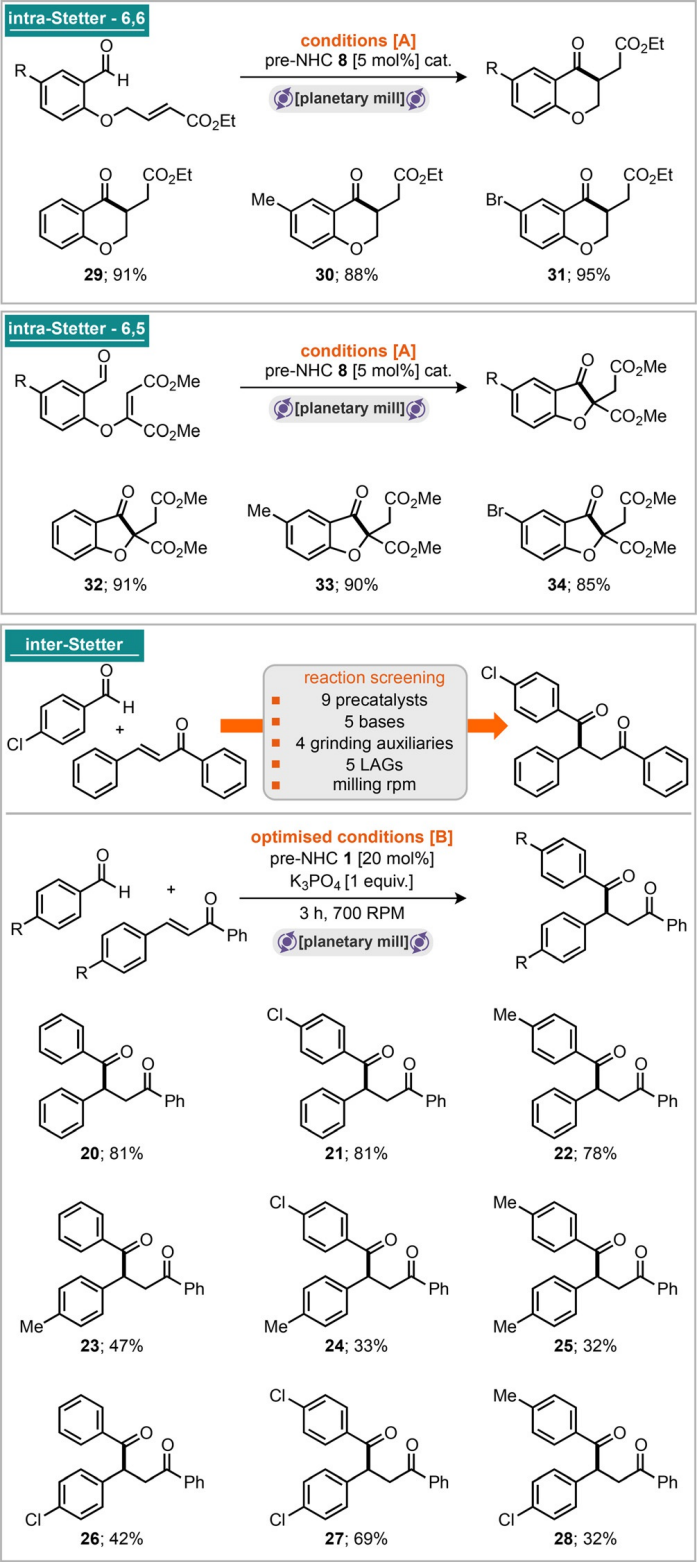
NHC‐catalysed Stetter reaction under milling conditions.

**Scheme 3:**
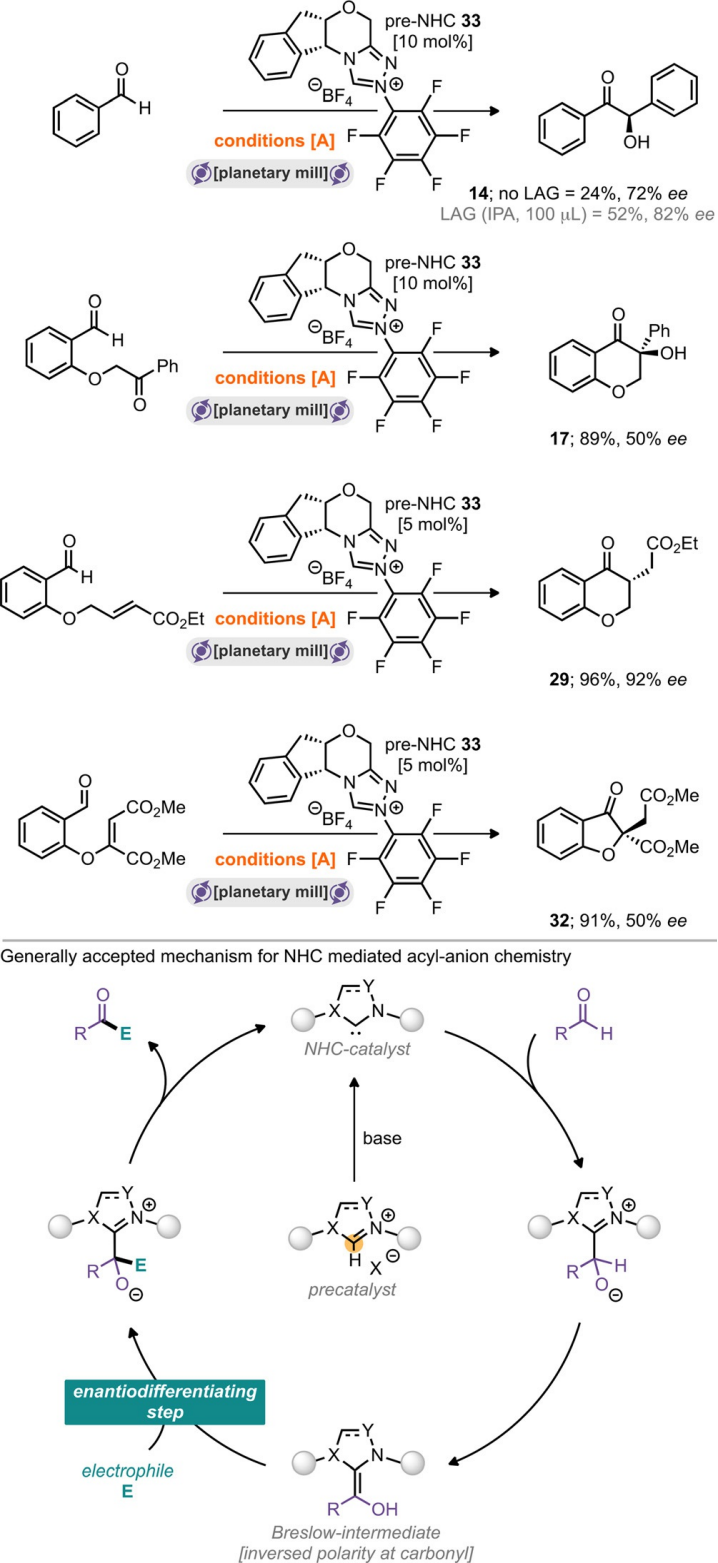
Overview of asymmetric NHC‐mediated acyl anion chemistry under milling conditions.

Despite these results, some of the *ee* values are lower than those reported under solution conditions, perhaps pointing towards poorer temperature control of the milled process.5k However, these are complex reactions, and clearly the balance of all reaction parameters requires significant fine‐tuning to deliver optimal conditions for maximum enantioselectivity, and this would have to be compared directly with a fully optimized solution protocol. Thus, the conclusion proposed here is that imparting enantioselectivity of NHC‐catalysed reactions under ball‐milling is possible and can deliver excellent results, although how these compare directly against solution‐phase conditions is not yet clear. Nonetheless, it is particularly notable that carbenes can be used catalytically in complex reaction pathways and also impart stereocontrol in the absence of solvent and under milling conditions.

In summary, ball‐milling has been used as a technique to conduct NHC catalysis for the first time. A range of catalysts, bases and grinding auxiliaries have been screened to reach general conditions for the acyl anion activation mode, which has been demonstrated in four application areas: intermolecular‐benzoin, intramolecular‐benzoin, intermolecular‐Stetter and intramolecular‐Stetter. Notably, these reactions are run without precaution to obscure air and moisture from the reaction vessel, and in general this works well, although, in some instances, increased catalyst loadings compared to solvent‐based techniques are used. Notably, among all catalysts screened, perhaps the simplest and longest‐known thiazolium pre‐NHC **1** is the most optimal catalyst for the intermolecular Stetter reaction under ball‐milling conditions. Finally, it has been demonstrated that several of the reaction modes can also be rendered asymmetric under milling conditions.

## Experimental Section

Information about the data that underpins the results presented in this article, including how to access them, can be found in the Cardiff University data catalogue at https://doi.org/10.17035/d.2019.0087455694. Further experimental details can be found in the Supporting Information⋅

## Conflict of interest

*The authors declare no conflict of interest*

## Supporting information

As a service to our authors and readers, this journal provides supporting information supplied by the authors. Such materials are peer reviewed and may be re‐organized for online delivery, but are not copy‐edited or typeset. Technical support issues arising from supporting information (other than missing files) should be addressed to the authors.

Supplementary

## References

[1] For the IUPAC Gold Book definition of mechanochemistry see: https://goldbook.iupac.org/terms/view/MT07141 or 10.1351/goldbook.MT07141.

[2] For selected reviews in the area of mechanochemistry see:

[2a] J. L. Howard , Q. Cao , D. L. Browne , Chem. Sci. 2018, 9, 3080–3094; 29780455 10.1039/c7sc05371a PMC5933221

[2b] J. Andersen , J. Mack , Green Chem. 2018, 20, 1435–1443;

[2c] J.-L. Do , T. Friščić , ACS Cent. Sci. 2017, 3, 13–19; 28149948 10.1021/acscentsci.6b00277 PMC5269651

[2d] J. G. Hernández , C. Bolm , J. Org. Chem. 2017, 82, 4007–4019; 28080050 10.1021/acs.joc.6b02887

[2e] T.-X. Métro , J. Martinez , F. Lamaty , ACS Sustainable Chem. Eng. 2017, 5, 9599–9602;

[2f] D. Tan , F. García , Chem. Soc. Rev. 2019, 48, 2274–2292; 30806391 10.1039/c7cs00813a

[2g] N. Willis-Fox , E. Rognin , T. A. Aljohani , R. Daly , Chem 2018, 4, 2499–2537;

[2h] M. J. Muñoz-Batista , D. Rodriguez-Padron , A. R. Puente-Santiago , R. Luque , ACS Sustainable Chem. Eng. 2018, 6, 9530–9544;

[2i] J. C. Robertson , M. L. Coote , A. C. Bissember , Nat. Chem. Rev. 2019, 3, 290–304.

[3] For some recent publications in the area of mechanochemistry see:

[3a] K. L. Denlinger , L. Ortiz-Trankina , P. Carr , K. Benson , D. C. Waddell , J. Mack , Beilstein J. Org. Chem. 2018, 14, 688–696; 29623132 10.3762/bjoc.14.57 PMC5870152

[3b] L. Appy , A. Depaix , X. Bantreil , F. Lamaty , S. Peyrottes , B. Roy , Chem. Eur. J. 2019, 25, 2477–2481; 30549335 10.1002/chem.201805924

[3c] M. Ferguson , M. S. Moyano , G. A. Tribello , D. E. Crawford , E. M. Bringa , S. L. James , J. Kohanoff , M. G. D. Pópolo , Chem. Sci. 2019, 10, 2924–2929; 30996870 10.1039/c8sc04971h PMC6427933

[3d] K. J. Ardila-Fierro , D. E. Crawford , A. Körner , S. L. James , C. Bolm , J. G. Hernández , Green Chem. 2018, 20, 1262–1269;

[3e] Y. Yeboue , B. Gallard , N. Le Moigne , M. Jean , F. Lamaty , J. Martinez , T.-X. Métro , ACS Sustainable Chem. Eng. 2018, 6, 16001–16004;

[3f] K. J. Ardila-Fierro , C. Bolm , J. G. Hernández , Angew. Chem. Int. Ed. 2019, 58, 12945–12949; 10.1002/anie.201905670 PMC6773223 31265746

[3g] K. J. Ardila-Fierro , A. Pich , M. Spehr , J. G. Hernández , C. Bolm , Beilstein J. Org. Chem. 2019, 15, 811–817; 30992730 10.3762/bjoc.15.78 PMC6444433

[3h] Q. Cao , R. T. Stark , I. A. Fallis , D. L. Browne , ChemSusChem 2019, 12, 2554–2557; 31033237 10.1002/cssc.201900886 PMC6619031

[3i] A. Porcheddu , F. Delogu , L. De Luca , E. Colacino , ACS Sustainable Chem. Eng. 2019, 7, 12044–12051;

[3j] Z.-Z. Gu , F.-C. Guo , P. Zhang , Y.-J. Qin , Z.-X. Guo , Tetrahedron Lett. 2019, 60, 1687–1690;

[3k] K. Kubota , R. Takahashi , H. Ito , Chem. Sci. 2019, 10, 5837–5842. 31293773 10.1039/c9sc01711a PMC6566379

[4] We have been benefactors of serendipitous observations under ball-milling conditions in the following publications:

[4a] J. L. Howard , M. C. Brand , D. L. Browne , Angew. Chem. Int. Ed. 2018, 57, 16104–16108; 10.1002/anie.201810141 PMC6282732 30335216

[4b] J. L. Howard , Y. Sagatov , L. Repusseau , C. Schotten , D. L. Browne , Green Chem. 2017, 19, 2798–2802.

[5] For some recent examples of catalytic and/or enantioselective processes under ball-milling conditions see:

[5a] Q. Cao , J. L. Howard , E. Wheatley , D. L. Browne , Angew. Chem. Int. Ed. 2018, 57, 11339–11343; 10.1002/anie.201806480 PMC6220771 30015403

[5b] Q. Cao , W. I. Nicholson , A. C. Jones , D. L. Browne , Org. Biomol. Chem. 2019, 17, 1722–1726; 30226258 10.1039/c8ob01781f

[5c] J. Yu , Z. Li , K. Jia , Z. Jiang , M. Liu , W. Su , Tetrahedron 2013, 54, 2006–2009;

[5d] Z. Li , Z. Jiang , W. Su , Green Chem. 2015, 17, 2330–2334;

[5e] Y. Wang , H. Wang , Y. Jiang , C. Zhang , J. Shao , D. Xu , Green Chem. 2017, 19, 1674–1677;

[5f] P. Staleva , J. G. Hernández , C. Bolm , Chem. Eur. J. 2019, 25, 9202–9205; 31106927 10.1002/chem.201901826

[5g] Y. Pang , T. Ishiyama , K. Kubota , H. Ito , Chem. Eur. J. 2019, 25, 4654–4659; 30762271 10.1002/chem.201900685

[5h] Z. A. Ignatiuk , M. J. Janicki , R. W. Góra , K. Koniecznym , R. Kowalczyk , Adv. Synth. Catal. 2019, 361, 1108–1116;

[5i] K. Kubota , T. Seo , K. Koide , Y. Hasegawa , H. Ito , Nat. Commun. 2019, 10, 111; 30631071 10.1038/s41467-018-08017-9 PMC6328594

[5j] J. Andersen , J. Brunemann , J. Mack , React. Chem. Eng. 2019, 4, 1229–1236;

[5k] J. Andersen , J. Mack , Angew. Chem. Int. Ed. 2018, 57, 13062–13065; 10.1002/anie.201805263 30101557

[5l] C. G. Avila-Ortiza , M. Pérez-Venegasa , J. Vargas-Caporalia , E. Juaristi , Tetrahedron Lett. 2019, 60, 1749–1757.

[6] For the seminal work of secondary amine organocatalysis under ball-milling conditions see: B. Rodríguez , T. Rantanen , C. Bolm , Angew. Chem. Int. Ed. 2006, 45, 6924–6926; 10.1002/anie.200602820 17001709

[7] For papers concerning secondary amine organocatalysis under milling conditions see:

[7a] G. Guillena , M. del. Carmen Hita , C. Nájera , S. F. Viózquez , Tetrahedron: Asymmetry 2007, 18, 2300–2304;

[7b] A. Bruckmann , A. Krebs , C. Bolm , Green Chem. 2008, 10, 1131–1141;

[7c] G. Guillena , M. del Carmen Hita , C. Nájera , S. F. Viózquez , J. Org. Chem. 2008, 73, 5933–5943; 18598088 10.1021/jo800773q

[7d] J. G. Hernández , E. Juaristi , J. Org. Chem. 2011, 76, 1464–1467; 21250720 10.1021/jo1022469

[7e] J. G. Hernández , E. Juaristi , Tetrahedron 2011, 67, 6953–6959;

[7f] J. G. Hernández , V. García-López , E. Juaristi , Tetrahedron 2012, 68, 92–97;

[7g] P. Chauhan , S. S. Chimni , Asian J. Org. Chem. 2012, 1, 138–141;

[7h] E. Veverková , V. Poláčková , L. Liptáková , E. Kázmerová , M. Mečiarová , Š. Toma , R. Šebesta , ChemCatChem 2012, 4, 1013–1018;

[7i] J. G. Hernández , E. Juaristi , Chem. Commun. 2012, 48, 5396–5409; 10.1039/c2cc30951c 22517403

[7j] P. Chauhan , S. S. Chimni , Beilstein J. Org. Chem. 2012, 8, 2132–2141. 23243475 10.3762/bjoc.8.240 PMC3520570

[8] For reviews on NHC organocatalysis see:

[8a] D. Enders , O. Niemeier , A. Henseler , Chem. Rev. 2007, 107, 5606–5655; 17956132 10.1021/cr068372z

[8b] N. Marion , S. Díez-González , S. P. Nolan , Angew. Chem. Int. Ed. 2007, 46, 2988–3000; 10.1002/anie.200603380 17348057

[8c] J. Read de Alaniz , T. Rovis , Synlett 2009, 1189–1207; 20585467 10.1055/s-0029-1216654 PMC2888141

[8d] V. Nair , R. S. Menon , A. T. Biju , C. R. Sinu , C. R. R. R. Paul , A. Jose , V. Sreekumar , Chem. Soc. Rev. 2011, 40, 5336–5346; 21776483 10.1039/c1cs15139h

[8e] X. Bugaut , F. Glorius , Chem. Soc. Rev. 2012, 41, 3511–3522; 22377957 10.1039/c2cs15333e

[8f] J. Douglas , G. Churchill , A. D. Smith , Synthesis 2012, 44, 2295–2309;

[8g] D. M. Flanigan , F. Romanov-Michailidis , N. A. White , T. Rovis , Chem. Rev. 2015, 115, 9307–9387; 25992594 10.1021/acs.chemrev.5b00060 PMC4986729

[8h] R. S. Menon , A. T. Biju , V. Nair , Chem. Soc. Rev. 2015, 44, 5040–5052. 26014054 10.1039/c5cs00162e

[9] R. Breslow , J. Am. Chem. Soc. 1958, 80, 3719–3726.

[10] Ema et al. have reported acyl anion NHC organocatalysis under solvent-free conditions (stirring materials neat in a reaction tube): T. Ema , Y. Nanjo , S. Shiratori , Y. Terao , R. Kimura , Org. Lett. 2016, 18, 5764–5767. 27779419 10.1021/acs.orglett.6b03115

[11] For further details see the Supporting Information.

[12] J. Mahatthananchai , J. W. Bode , Chem. Sci. 2012, 3, 192–197. 23687565 10.1039/C1SC00397F PMC3655724

[13] M. S. Kerr , T. Rovis , J. Am. Chem. Soc. 2004, 126, 8876–8877. 15264801 10.1021/ja047644h

