# Supplementary material for: N‐Heterocyclic Carbene Acyl Anion Organocatalysis by Ball‐Milling
Source: ChemSusChem. 2019 Nov 27;13(1):131–5. doi: 10.1002/cssc.201902346 (PMC6972762; doi:10.1002/cssc.201902346)

CHEMISTRY & SUSTAINABILITY
CHEMSUSCHEM
ENERGY & MATERIALS

Supporting Information

N-Heterocyclic Carbene Acyl Anion Organocatalysis by Ball-Milling

William I. Nicholson, Alex C. Seastram, Saqib A. Iqbal, Benjamin G. Reed-Berendt, Louis C. Morrill,* and Duncan L. Browne*[a]

cssc_201902346_sm_miscellaneous_information.pdf

Supporting Information

Table of Contents

1. General Information..S2
2. Optimisation……………...S4
3. Pre NHC Characterization Data...................…………………………………….......S9
4. Starting material and Product Characterization Data..………………………………..S16
5. HPLC Traces and Optical Rotation Measurements…………………………………...S33
6. NMR Spectra...S38

General Information

All aldehydes that are liquid at room temperature were freshly distilled before use, all other reagents were purchased from commercial sources and used without further purification. Thin layer chromatography (TLC) was carried out using Merck TLC silica gel 60 sheet and visualized with ultraviolet light or potassium permanganate stain.

Flash column chromatography (FCC) was performed with Sigma Aldrich silica gel 40-60 Å as the stationary phase and solvents employed were analytical grade.

^{1}H, ^{13}C, and ^{19}F NMR spectra were recorded on a Bruker 400 Ultrashield or Bruker AVX500 (500 MHz) spectrometer at ambient temperature. The obtained chemical shifts; δ, are reported in ppm and are referenced to the residual solvent signal. Spin-spin coupling constants; J, are given in Hz. NMR yields were measured using 0.33 mmol or 0.066 mmol of Mesitylene as an internal standard.

Melting points were measured on a Gallenkamp melting point apparatus and are reported corrected by linear calibration to benzophenone (47 - 49 °C) and benzoic acid (121 - 123 °C).

High resolution mass spectral (HRMS) data were obtained on a Thermo Scientific LTQ Orbitrap XL by the EPSRC UK National Mass Spectrometry Facility at Swansea University or on a Waters MALDI-TOF mx in Cardiff University. Spectra were obtained using electron impact ionization (EI), chemical ionization (CI), positive electrospray (ES), pneumatically assisted electrospray (pNSI) or atmospheric solids analysis probe (ASAP+).

Infrared spectra were recorded on a Shimadzu IR-Affinity-1S FTIR spectrometer.

The ball mill used was a Fritsch Planetary Micro Mill model “Pulverisette 7” using 12 mL Zirconium oxide grinding bowls containing 50 mm Zirconium oxide grinding balls. The grinding cycle was set to 15 minutes and to alternate direction between cycles after a 1 minute pause, unless stated otherwise. The mill was set to repeat these cycles until the reaction time had been reached. Reactions for 15 minutes or less were set for only a single cycle with no pause.

Optical rotation measurements were taken on a Bellingham and Stanley ADP410 polarimeter at ambient temperature, using a LED light source filtered to 589.3 nm.

HPLC analyses were obtained on a Gilson HPLC consisting of a Gilson 305 pump, Gilson 306 pump, Gilson 811C dynamic mixer, Gilson 805 manometric module, Gilson 401C dilutor, Gilson 213XL sample injector and sample detection was performed with a Gilson 118 UV/vis detector. Separation was achieved using a Chiralpak IC column.

Aldehyde Distillation Conditions

All aldehydes were distilled using a short path distillation head under reduced pressure and the aldehydes were used immediately following purification.

**Substrate**	**Temperature** (˚C)	**Pressure** (mbar)
Benzaldehyde	100	30
4-Fluorobenzaldehyde	118	35
p-Tolaldehyde	120	33
p-Anisaldehyde	162	35
4-(Trifluoromethyl)-benzaldehyde	96	35

Table S1

Optimisation

Intermolecular benzoin [Conditions A] – catalyst screening

Early work on the discovery of reaction conditions for the ball-milled NHC catalysed intermolecular benzoin reaction delivered good initial results with Cs_2CO_3 as base, sand as a grinding auxiliary and a milling speed of 300 rpm. With these results we explored a range of known pre-NHC catalysts as well as reaction times, the results are shown in Table S1.

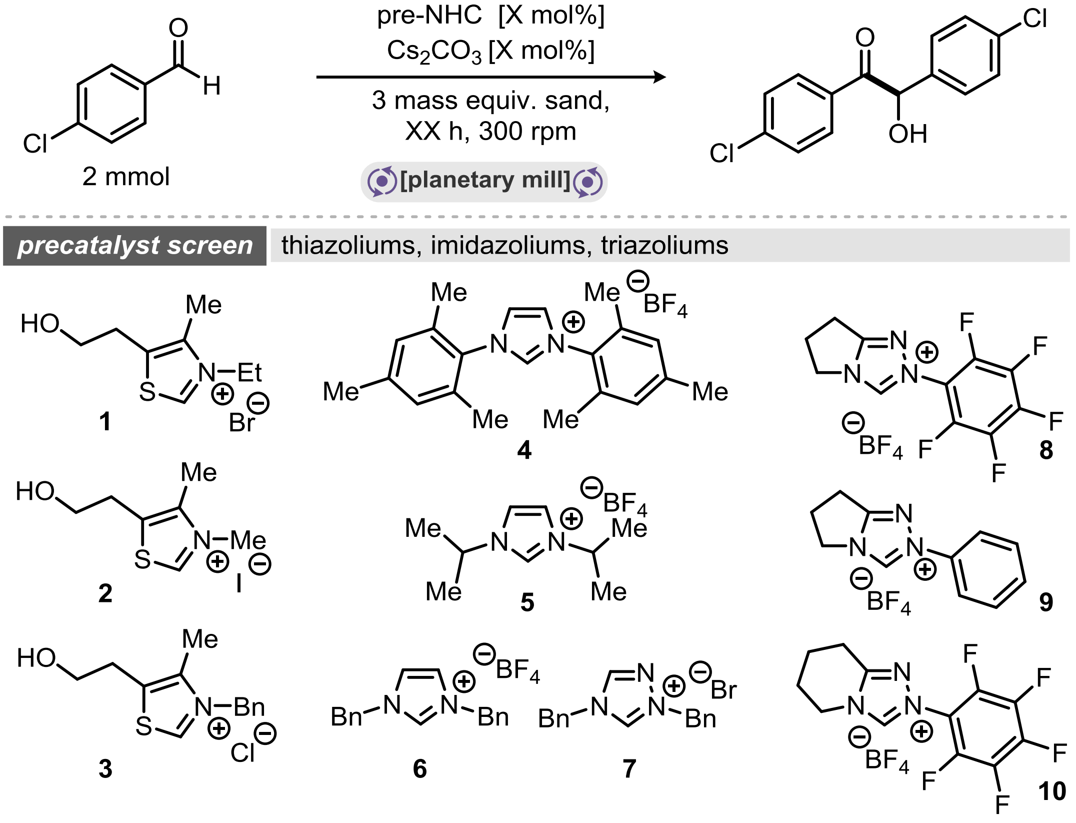

NHC	NHC (X mol%)	Base (X mol%)	Time (h)	NMR Yield
1	20	20	3	65%
2	20	20	3	37%
3	20	20	3	67%
4	20	20	3	0
5	20	20	3	0
6	20	20	3	0
7	20	20	3	52%
8	20	20	3	70%
9	20	20	3	63%
10	20	20	3	67%
1	1	1	1	Trace
3	1	1	1	Trace
8	1	1	1	Trace
8	1	1	1	8%
8	1	1	0.25	10%
8	1	1	0.5	6%

Table S2

Intermolecular benzoin **[Conditions A]** **– screening reaction time and grinding auxiliary loading**

The scale of the reaction was reduced to permit increased number of screening reactions per mmol of catalyst. This was offset by the inclusion of an increased quantity of snad (grinding auxiliary).

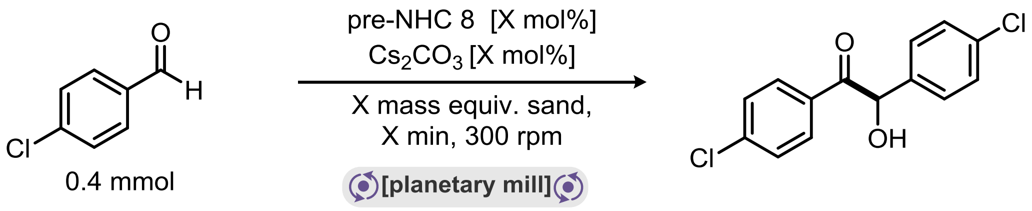

Entry	Time (min)	Mass equiv. of sand	Cat and base (mol%)	NMR Yield
1	15	15	10	79% (71%)
2	30	15	10	78%
3	15	15	5	33%
4	30	15	5	50%
5	60	15	5	45%

Table S3 – Isolated yields appear in parenthesis

Once the reaction was shown to be effective at reduced scale a range of bases, grinding auxiliaries, and mill speeds were investigated.

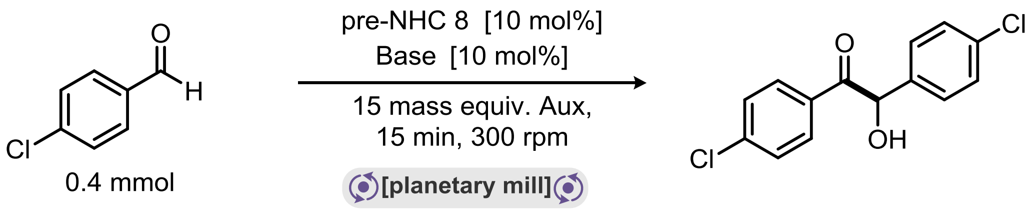

Entry	Grinding Aux.	Base	RPM	NMR Yield
1	Sand	Cs_2CO_3	300	79% (71%)
2	Sand	Na_2CO_3	300	75%
3	Sand	K_3PO_4	300	62%
4	Sand	DBU	300	10%
5	Sand	NEt_3	300	0%
6	NaCl	Cs_2CO_3	300	38%
7	$MgSO_4$	Cs_2CO_3	300	Trace
8	Sand	Cs_2CO_3	400	74%
9	Sand	Cs_2CO_3	500	61%
10	Sand	Cs_2CO_3	600	40%
11	Sand	Cs_2CO_3	700	15%
12	Sand	Cs_2CO_3	800	Trace
13	Sand	Cs_2CO_3	250	72%

Table S4 – Isolated yields appear in parenthesis

Intermolecular benzoin – application of [Conditions A] to other substrates

Upon exploring these reaction conditions to two other substrates it was found that the conditions were not broadly applicable. A series of LAG (Liquid Assisted Grinding) agents was next explored using benzaldehyde as a substrate.

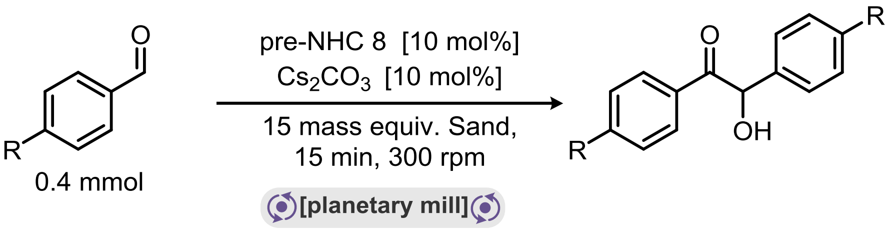

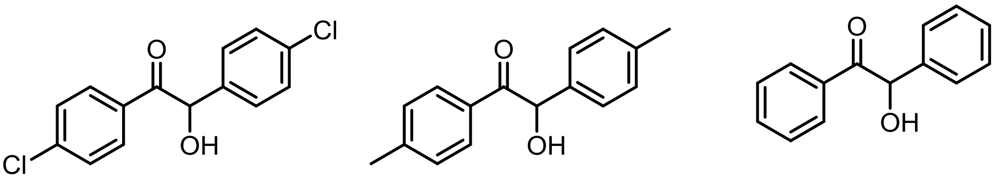

Isolated 72% | Trace by NMR | 39% isolated

Intermolecular benzoin – exploring LAG agents

Finally, a variety of liquid additives (LAGs) were screened in the reaction with 2-propanol returning the highest yield using 100 μL of the additive. These conditions were then applied to a range of substrates with and with the liquid additive.

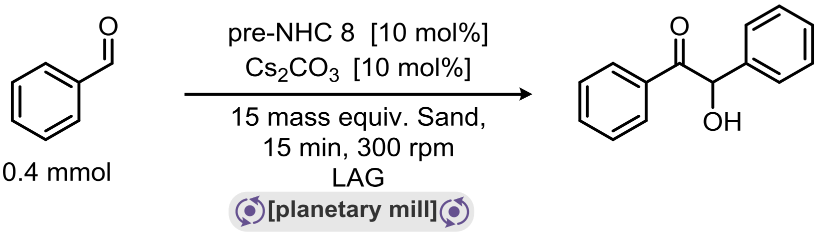

Entry	LAG	Quantity (μL)	Dielec. Const.	NMR Yield
1	EtOAC	100	6.02	33%
2	THF	100	7.58	29%
3	DCM	100	8.93	58%
4	HFIP	100	16.7	Trace
5	IPA	100	17.9	82% (76%)
6	EtOH	100	24.5	58%
7	DMF	100	36.7	12%
8	MeCN	100	37.5	19%
9	DMA	100	37.8	17%
10	DMSO	100	46.7	56%
11	IPA	50	17.9	60%
12	IPA	200	17.9	76%

Table S5 – Isolated yields appear in parenthesis

Intermolecular Stetter – exploring [Conditions A]

The previously optimised conditions were applicable to the intermolecular benzoin, intramolecular benzoin and intramolecular Stetter reactions. However, exploring several variations of these conditions they could not be applied to the intermolecular Stetter and instead lead to significant amounts of the benzoin product as may be expected.[1]

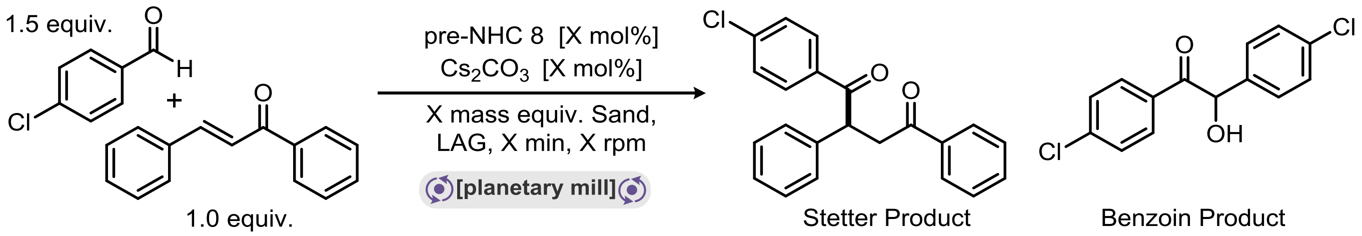

Entry	Scale (mmol)	Sand mass equiv.	LAG	Base (mol%)	Cat. (mol%)	Time (mins)	NMR Yield	
							Benzoin	Stetter
1	1	3	IPA	5	5	15	48%	Trace
2	1	3	-	5	5	15	50%	Trace
3	1	0	-	5	5	15	42%	Trace
4	0.2	15	IPA	5	5	30	41%	6%
5	0.2	15	-	5	5	30	41%	8%
6	0.2	0	-	5	5	30	43%	3%
7	0.2	0	-	100	5	60	18%	Trace

Table S6 – Isolated yields appear in parenthesis

Once it was clear that the previously optimised conditions were unable to be transferred to the intermolecular Stetter reaction this lead to its complete reoptimisation. The Stetter reaction was then explored using 4-chlorobenzaldehyde, and chalcone as substrates with all variables investigated in this system as before.

[1] H. Stetter; *Angew Chem. Int. Ed*., 1976, **15**, 639-647

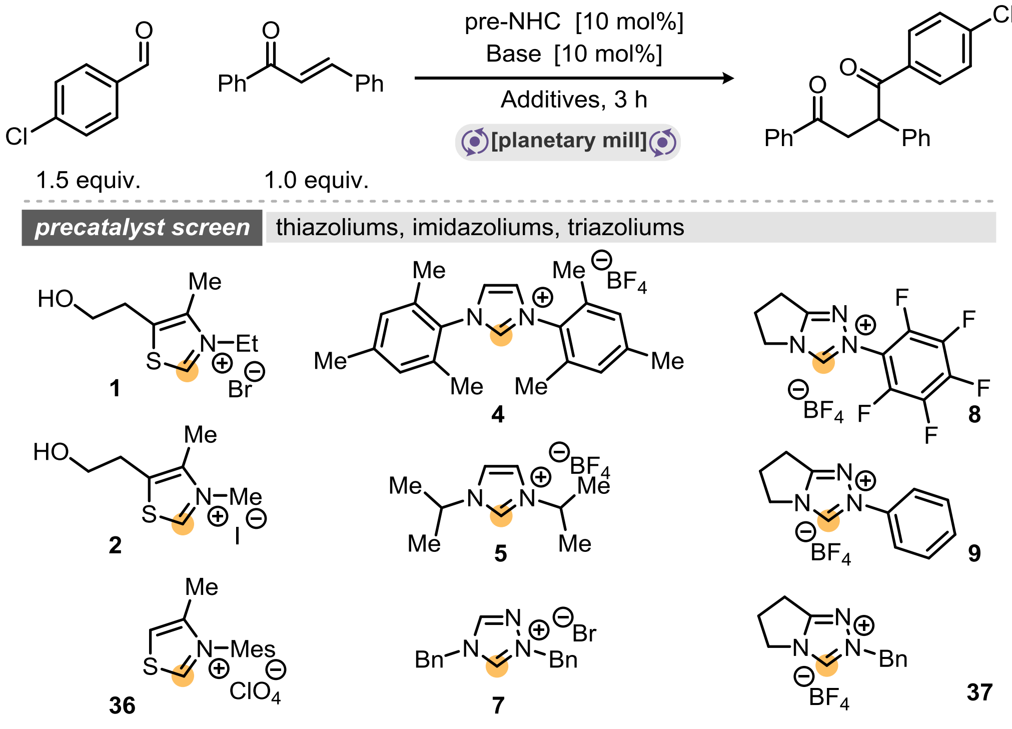

Entry	Base	NHC Pre cat	Grinding Aux.	Rotation speed	Liquid additive	NMR Yield %
1	K_3PO_4	1	-	500	-	34
2	K_3PO_4	2	-	500	-	19
3	K_3PO_4	36	-	500	-	Traces
4	K_3PO_4	4	-	500	-	6
5	K_3PO_4	5	-	500	-	Traces
6	K_3PO_4	7	-	500	-	31
7	K_3PO_4	9	-	500	-	7
8	K_3PO_4	37	-	500	-	29
9	K_3PO_4	8	-	500	-	Traces
10	K_2CO_3	1	-	500	-	36
11	DBU	1	-	500	-	33
12	Cs_2CO_3	1	-	500	-	33
13	K_3PO_4	1	Sand 3 m. equiv.	500	-	31
14	K_3PO_4	1	NaCl 3 m. equiv.	500	-	28
15	K_3PO_4	1	1 eq Base	500	-	72
16	K_2CO_3	1	1 eq Base	500	-	55
17	K_3PO_4	1	1 eq Base	400	-	32
18	K_3PO_4	1	1 eq Base	600	-	55
19	K_3PO_4	1	1 eq Base	700	-	81 (81%)
20	K_3PO_4	1	1 eq Base	800	-	77
21	K_3PO_4	1	1 eq Base	700	MeCN 200 µL	72
22	K_3PO_4	1	1 eq Base	700	DCM 200 µL	76
23	K_3PO_4	1	1 eq Base	700	PhMe 200 µL	77
24	K_3PO_4	1	1 eq Base	700	IPA 200 µL	75
25	K_3PO_4	1	1 eq Base	700	THF 200 µL	79

Table S7 – Isolated yields appear in parenthesis

Pre NHC Characterization Data

5-(2-hydroxyethyl)-3,4-dimethylthiazol-3-ium iodide (2)

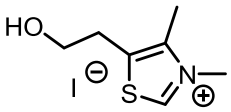

The title compound was prepared using a method modified from the literature.[2] To a 50 mL round bottom flask was added 5-(2-hydroxyethyl)-4-methylthiazole (1.20 mL, 10.1 mmol) and methyl iodide (20.9 mmol) this mixture was stirred and heated at reflux for 2 hours. The resulting solution was concentrated *in vacuo*, before diethyl ether (20 mL) was added to the brown residue and the mixture was stirred for 30 minutes. The resulting precipitate was then filtered to give **2** as a pale-yellow solid (2.51 g, 88%); **mp** 79 – 82 ˚C, **^{1}H NMR** (500 MHz, D_2O) δ 9.71 (1H, s), 4.13 (3H, s), 3.89 (2H, t, *J* = 5.9 Hz), 3.18 (2H, t, *J* = 5.9 Hz), 2.52 (3H, s). **^{13}C NMR** (126 MHz, D_2O) δ 155.0, 143.1, 134.7, 60.5, 40.4, 29.3, 11.1. **HRMS** (NSI+) calcd for $C_7H_{12}NOS$ [M]+ 158.0634, found 158.0631. **IR** (cm^{-1}) 3283, 3022, 1593, 1474, 1439, 1406, 1364, 1055.

3-mesityl-4-methylthiazol-3-ium perchlorate (36)

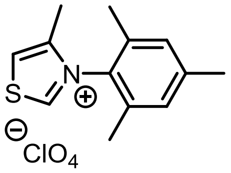

The title compound was prepared using a method modified from the literature.[3] A solution of 2,4,6-trimethyl aniline (1.40 ml, 10.0 mmol) in DMSO (5.00 mL) was treated with 20 N aqueous NaOH (0.50 mL, 10.0 mmol) at ambient temperature. The mixture was cooled to 0 °C and CS_2 (0.70 mL, 10.0 mmol) was added. Upon stirring for 1 h at ambient temperature, a change of colour from dark-red to orange occurred. The mixture was cooled to 0 °C and chloroacetone (0.8 ml, 4.0 mmol) was added. After 1 h of stirring at ambient temperature, water (10 mL) was added and the mixture was stirred for an additional 10 min at 0 °C upon which a yellow solid precipitated. The solid was filtered, dissolved in ethanol (10 mL) and treated with 36% hydrochloric acid (0.5 mL) for 1 h at 80 °C. Upon cooling to ambient temperature, the crude thione precipitated. After filtration and recrystallization from ethanol, the thione was then used without further purification.

Thione (2.12 g, 8.5 mmol) was dissolved in acetic acid (35 mL) and treated with 30% aqueous H_2O_2 (2.4 mL, 25.0 mmol). Upon stirring for 30 min at ambient temperature the colour of the solution turned from pale yellow to orange. The solvent was removed *in vacuo*. The residue was dissolved in methanol (5 mL) and treated with a solution of $NaClO_4$ (4.8 g, 34 mmol) in a 2:1 (v/v) mixture of methanol/water

[2] W. Fan, Y. Wu, X-K. Li, N. Yao, X. Li, Y-G. Yu, and L. Hai; *Eur. J. Med. Chem.*, 2011, **46**, 3651-3651.
[3] I. Piel, M. D. Pawelczyk, K. Hirano, R. Frolich, and F. Glorius; *Eur. J. Org. Chem*. 2011, **28**, 5475–5484

(100 mL). Upon stirring at 0 °C, a white solid precipitated, which was filtered and washed subsequently with water (25 mL) and Et2O (25 mL). After recrystallisation from methanol, compound 36 (7.12 g, 74%) was obtained as a colourless crystalline solid**. mp** 164 - 165 ˚C. **^{1}H NMR** (500 MHz, DMSO) δ 10.41 (d, J = 2.6 Hz, 1H), 8.35 (dd, J = 2.6, 1.1 Hz, 1H), 7.22 (d, J = 0.4 Hz, 2H), 2.36 (s, 3H), 2.18 (d, J = 0.9 Hz, 3H), 1.92 (s, 6H). **^{13}C NMR** (126 MHz, DMSO) δ 161.2, 145.7, 141.1, 133.8, 132.4, 129.7, 123.2, 20.6, 16.7, 12.5.

1,3-dimesityl-1H-imidazol-3-ium tetrafluoroborate (4)

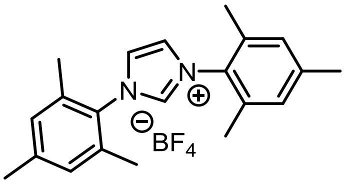

The title compound was prepared using a method modified from the literature.[4] To a 250 mL round bottom flask, 2,4,6-trimethylaniline (21.7 mL, 154 mmol), isopropanol (75.0 mL, 2.00 M) were added and the mixture was then stirred. Glyoxal (40% aq. solution, 8.70 mL, 75.9 mmol) in water (25.0 mL) and isopropanol (25.0 mL) was added in one portion and the solution was stirred at room temperature for 24 hours. The resulting suspension was then filtered and rinsed with water (2 × 50 mL) before being recrystallised from toluene to afford *N,N*-dimesitylethane-1,2-diimine as a yellow solid (16.6 g, 74%) that was used in the next step without further purification. mp 150 – 152 ˚C

N,N-dimesitylethane-1,2-diimine (4.39 g, 15.0 mmol) was added over the course of 10 minutes to a solution of paraformaldehyde (544 mg, 18.1 mmol) in toluene (60 mL, 0.3 M) and tetrafluoroboric acid (48% aq. solution, 2.80 mL, 21.4 mmol). This afforded a brown solution that was then stirred at 60 ˚C for 4 hours to give a black slurry which was cooled to rt and stirred overnight. The resulting precipitate was filtered through a Büchner funnel, washed with EtOAc, and dried under high vacuum to give the title compound as an off-white powder (3.94 g, 67% yield). **mp** 240 – 243 ˚C; **^{1}H NMR** (500 MHz, $CDCl_3$) δ 8.86 (1H, s), 7.56 (2H, d, J = 1.3), 7.03 (4H, s), 2.35 (6H, s), 2.11 (12H, s). **^{13}C NMR** (126 MHz, $CDCl_3$) δ 141.7, 137.5, 134.2, 130.5, 130.1, 125.2, 21.3, 17.3

1,3-diisopropyl-1H-imidazol-3-ium tetrafluoroborate (5)

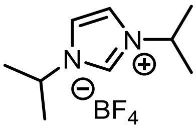

The title compound was prepared using a method modified from the literature.[5] To a 50 mL round bottom flask equipped with a magnetic stirrer was added isopropylamine (1.70 mL, 20.0 mmol) and isopropanol (10 mL, 2.00 M) and the mixture was then stirred. Glyoxal (40% aq. solution, 1.15 mL, 10 mmol) in water (5.0 mL) and isopropanol (5.0 mL) was added in one portion and the solution was stirred at room temperature for 24 hours. The resulting suspension was then filtered and rinsed with water (2 × 10 mL)

---

[4] M. Hans, J. Lorowski, A. Demonceau, and L. Delaude; *Beilstein J. Org. Chem*., 2015, **11**, 2318-2325.
[5] D. J. Kim, K. H. Oh, and J. K. Park; *Green Chem*., 2014,**16**, 4098-4101.

before being recrystallised from toluene to afford a yellow solid (1.12 g, 80%) that was used in the next step without further purification.

N,N-diisopropyl-1,2-diimine (0.70 g, 5.0 mmol) was added over the course of 10 minutes to a solution of paraformaldehyde (0.225 g, 7.50 mmol) in toluene (18 mL, 0.3 M) and tetrafluoroboric acid (48% aq. solution, 1.30 mL, 10 mmol). This afforded a brown solution that was then stirred at 60 ˚C for 4 hours to give a black slurry which was cooled to rt and stirred overnight. The resulting precipitate was filtered and washed with EtOAc and dried under high vacuum to give the title compound as a white powder (0.636 g, 53% yield). **mp** 80 - 82 ˚C, **^{1}H NMR** (400 MHz, DMSO) δ 9.24 (s, 1H), 7.92 (d, *J* = 1.7 Hz, 2H), 4.60 (hept, *J* = 6.7 Hz, 2H), 1.48 (d, *J* = 6.7 Hz, 12H). **^{13}C NMR** (101 MHz, DMSO) δ 133.5, 120.7, 52.3, 22.3.

1,4-dibenzyl-4H-1,2,4-triazol-1-ium bromide (7)

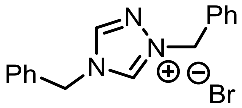

The title compound was prepared using a method modified from the literature.[6] A 250 mL round bottom flask was charged with 1,2,4 triazole (1.38 g, 20 mmol) in dry MeCN (100 mL) under N_2. After 10 minutes of stirring, benzyl bromide (4.70 mL, 40 mmol) was added and the reaction stirred for 2 days at 50 °C under N_2. The reaction was cooled, and the white precipitate filtered. The solid was washed with DCM (3 x 20 mL) and dried to afford a white solid 79% (2.51 g, 15.8 mmol). The compound was used immediately in the next reaction without further purification.

1H-benzyl-1,2,4 triazolium (1.91 g, 12 mmol), benzyl bromide (2.85 mL, 24 mmol) in MeCN (15 mL) were heated at reflux overnight under N_2. Acetonitrile was removed *in vacuo* to yield a solid orange residue which was subsequently washed with diethyl ether (20 mL). The residue was dissolved in DCM and the solution was added dropwise to diethyl ether. The solid was filtered to yield the pure dibenzyl triazolium salt 7, 85% (3.34 g, 10.20 mmol) as a white powder. **mp** 155 - 158 °C. **^{1}H NMR** (400 MHz, $CDCl_3$) δ 12.07 (s, 1H), 8.34 (s, 1H), 7.55 (m, 4H), 7.44 – 7.34 (m, 6H), 5.74 (s, 2H), 5.69 (s, 2H). **^{13}C NMR** (101 MHz, $CDCl_3$) δ 143.1, 142.8, 131.6, 131.3, 130.2, 129.9, 129.8, 129.5, 129.5, 129.4, 56.5, 52.4. HRMS calcd for C16H16N3Br [M-Br]+: 250.1336, found 250.1344. IR (cm^{-1}): 2972, 2941, 1572, 1456, 1354, 1150, 1007, 908, 793, 723, 696, 633, 470

[6] L. Myles, N. Gathergood, and S. Connon; *Chem. Commun*., 2013, **49**, 5316-5318

1,3-dibenzyl-1H-imidazol-3-ium tetrafluoroborate (6)

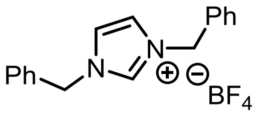

The title compound was prepared using a method modified from the literature.[4] A 500 mL round bottomed flask was charged with a magnetic stirrer bar, toluene (100 mL), benzylamine (10.92 mL, 100 mmol), and paraformaldehyde (3.00 g, 100 mmol). The resultant suspension was stirred at room temperature for 30 minutes, before cooling to 0 ˚C. Benzylamine (10.92 mL, 100 mmol) was added, and after a further 10 minutes stirring at 0 ˚C tetrafluoroboric acid (48% w/w in H_2O, 16.3 mL, 125 mmol) was added portion wise over 15 minutes. The reaction was then warmed to room temperature, and glyoxal (40% w/w in H_2O, 11.5 mL, 100 mmol) was added. The resultant mixture was then heated to 50 ˚C and stirred at this temperature for ~16 hours. The reaction was then cooled to room temperature. Dichloromethane (100 mL) and H_2O (50 mL) were added, and the layers separated. The aqueous phase was then extracted with dichloromethane (3 × 100 mL), and the combined organic phases dried over magnesium sulfate, filtered, and concentrated under reduced pressure. The residue was then purified by recrystallisation from IPA, to give the title compound (28.9 g, 89%) as a beige solid; **mp** 88 - 91 ˚C **^{1}H NMR** (500 MHz, $CDCl_3$) δ 9.12 (1H, s), 7.35-7.39 (10 H), 7.10 (2H, d, *J* = 1.6), 5.32 (4H, s). **^{13}C NMR** (126 MHz, $CDCl_3$) δ 135.9, 132.9, 129.6, 129.5, 129.1, 122.3, 53.5.

2-phenyl-6,7-dihydro-5H-pyrrolo[2,1-c][1,2,4]triazol-2-ium tetrafluoroborate (9)

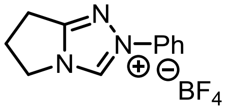

The title compound was prepared using a method modified from the literature.[7] An oven dried flask containing a magnetic stirrer was placed under atmosphere of nitrogen. The vessel was then charged with dry DCM (120 mL) and pyrollidin-2-one (1.15 g, 18.1 mmol). The reaction was stirred vigorously, and trimethyloxonium tetrafluoroborate (2.00 g, 20.0 mmol) was added in a single portion. The reaction mixture was stirred overnight before phenyl hydrazine (1.30 mL, 18.1 mmol) and the mixture was left to stir for 2 more days before removing the solvent *in vacuo*. The residue was dissolved in MeOH (10 mL) and triethylorthoformate (40 mL) and refluxed overnight. After cooling the resulting precipitate was filtered and recrystallised from MeOH to give the title compound as a tan solid (2.17 g, 44%) **mp** 151-153 ˚C. **^{1}H NMR** (500 MHz, DMSO) δ 10.69 (s, 1H), 7.90 – 7.85 (m, 2H), 7.72 – 7.66 (m, 2H), 7.65 – 7.60 (m, 1H), 4.41 (2H, t, J = 7.19), 3.21 (2H, t, J = 7.54), 2.75 (m, 2H). **^{13}C NMR** (126 MHz, DMSO) δ 163.0, 138.4, 135.6, 130.4, 130.3, 120.7, 46.9, 26.6, 21.3.

[7] J. E. Thomson, K. Rix, and A. D. Smith; *Org, Lett.*, 2006, **8**, 3785-3788

2-(perfluorophenyl)-6,7-dihydro-5H-pyrrolo[2,1-c][1,2,4]triazol-2-ium tetrafluoroborate (8)

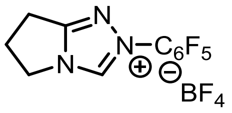

The title compound was prepared using a method modified from the literature.[8] A flame dried flask containing a magnetic stirrer was placed under atmosphere of nitrogen. The vessel was then charged with dry DCM (10 mL) and pyrollidin-2-one (152 μL, 2.0 mmol). The reaction was stirred vigorously, and trimethyloxonium tetrafluoroborate (299 mg, 2.02 mmol) was added in a single portion. The flask was then purged with nitrogen, and stirred overnight at room temperature. To this solution was added (perfluorophenyl)hydrazine (396 mg, 2 mmol) in a single portion. The flask was then purged with nitrogen and stirred at room temperature for 2 hours. The flask was then transferred to a rotary evaporator, and the solvent removed. The resultant orange oil was stirred under vacuum at 110 ˚C for 2 hours, at which point a reflux condenser was attached, and the system subjected to three rounds of vacuum-nitrogen exchange. The nitrogen flow rate was then increased, and the condenser fitting lifted slightly. To the hot flask was added carefully triethylorthoformate (1.66 mL, 10 mmol), and the reflux condenser replaced. The reaction was then stirred at this temperature (110 ˚C) for 3 hours, then cooled to room temperature. Toluene (10 mL) was then added, and the resulting suspension filtered over a porosity 4 filter. The filtrate, a black sticky tar, was then washed thoroughly with toluene, until the eluent was clear. Methanol in toluene solutions (ranging from 1-20%) were then used to wash the crude oil, yielding a light beige solid. Note - the filtrate should be conserved, then the solvent removed under reduced pressure to yield additional crops that can be washed as described above. The resultant solid is then dried for several hours under vacuum at 150 ˚C to give the title compound (234.5 mg, 32 %) as a beige powder; **mp** 239 - 242 ˚C, **^{1}H NMR** (500 MHz, $CDCl_3$) δ 10.53 (1H, s), 4.47 (2H, t, *J* = 7.7), 3.24 (2H, t, *J* = 7.7), 2.72-2.78 (2H, m). **^{13}C NMR** (126 MHz, $CDCl_3$) δ 164.4 (s), 144.0 (s), 143.4-143.6 (m), 141.9-142 (m), 141.4-141.6 (m), 138.5-138.7 (m), 136.3-136.8 (m), 111.4 (s), 48.1 (s), 26.5 (s), 21.5 (s).

2-(Perfluorophenyl)-5,6,7,8-tetrahydro-[1,2,4]triazolo[4,3-a]pyridin-2-ium tetrafluoroborate (10)

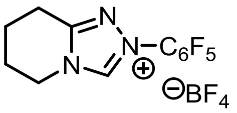

The title compound was prepared using a method modified from the literature.[8] A flame dried flask containing a magnetic stirrer was placed under atmosphere of nitrogen. The vessel was then charged with dry DCM (10 mL) and 2-Piperidone (0.198 g, 2 mmol). The reaction was stirred vigorously, and trimethyloxonium tetrafluoroborate (299 mg, 2.02 mmol) was added in a single portion. The flask was then purged with nitrogen and stirred overnight at room temperature. To this solution was added (perfluorophenyl)hydrazine (396 mg, 2 mmol) in a single portion. The flask was then purged with nitrogen, and stirred at room temperature for 2 hours. The flask was then transferred to a rotary evaporator, and the solvent removed. The resultant orange oil was stirred under vacuum at 110 ˚C for

[8] H. U. Vora, S. P. Lathrop, N. T. Reynolds, M. S. Kerr, J. R. Alaniz, and T. Rovis; *Organic Syntheses*, 2010, **87**, p. 350 – 361.

2 hours, at which point a reflux condenser was attached, and the system subjected to three rounds of vacuum-nitrogen exchange. The nitrogen flow rate was then increased, and the condenser fitting lifted slightly. To the hot flask was added carefully triethylorthoformate (1.66 mL, 10 mmol), and the reflux condenser replaced. The reaction was then stirred at this temperature (110 ˚C) for 3 hours, then cooled to room temperature. Toluene (10 mL) was then added, and the resulting suspension filtered over a porosity 4 filter. The filtrate, a black sticky tar, was then washed thoroughly with toluene, until the eluent was clear. Methanol in toluene solutions (ranging from 1-20%) were then used to wash the crude oil, yielding a light beige solid. Note - the filtrate should be conserved, then the solvent removed under reduced pressure to yield additional crops that can be washed as described above. The resultant solid is then dried for several hours under vacuum at 150 ˚C to give the title compound (136 mg, 18 %) as a beige powder; **mp** 248 - 250 ˚C, **^{1}H NMR** (500 MHz, Acetone) δ 10.32 (s, 1H), 4.70 (t, J = 6.1 Hz, 2H), 3.28 (t, J = 6.5 Hz, 2H), 2.33 – 2.25 (m, 2H), 2.25 – 2.15 (m, 2H). **^{13}C NMR** (126 MHz, Acetone) δ 156.1 (s), 146.7 (s), 145.7 – 145.3 (m), 145.1 (dd, J = 13.5, 3.6 Hz), 143.7 – 143.3 (m), 143.2 – 142.8 (m), 140.4 – 139.9 (m), 138.2 – 137.8 (m), 47.8 (s), 21.9 (s), 21.5 (s), 19.2 (s). **^{19}F NMR** (471 MHz, Acetone) δ -147.2 (d, J = 19.7 Hz), -150.5, -151.0 (m), -152.0 (d, J = 25.4 Hz), -162.2 162.5 (m).

2-benzyl-6,7-dihydro-5H-pyrrolo[2,1-c][1,2,4]triazol-2-ium tetrafluoroborate (37)

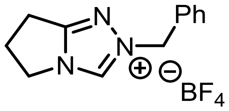

The title compound was prepared using a method modified from the literature.[9]

Benzylhydrazine dihydrochloride (1.5 g, 9.46 mmol) was suspended in diethyl ether (30 mL), cooled to -10 °C and stirred vigorously. After 5 minutes, 2 N NaOH (40 mL) was slowly added resulting in a biphasic mixture which was stirred vigorously for 90 minutes. The aqueous layer was extracted with diethyl ether (5 x 20 mL) and the combined organic layers were washed with brine, and dried over $MgSO_4$. The mixture was filtered and the filtrate concentrated *in vacuo* to yield a light orange oil 42% (0.399 g, 3.26 mmol) which was used immediately without further purification.

A 50 mL oven-dried round-bottomed flask was flushed with N_2. Pyrollidin-2-one (0.151 mL, 2 mmol), pre-dried over molecular sieves was added to the 50 mL round-bottomed flask containing anhydrous DCM (15 mL). Trimethyloxonium tetrafluoroborate (0.326 g, 2.2 mmol) was added and the reaction was stirred overnight at ambient temperature under nitrogen. Freshly prepared benzylhydrazine (0.399 g, 3.26 mmol) was added (neat) and the mixture was stirred overnight at ambient temperature. The solvent was removed *in vacuo* and triethyl orthoformate (12 mL) was added and the mixture was stirred overnight at reflux. The reaction was cooled and passed through a silica plug to remove the excess orthoformate. The crude product was obtained by washing the silica plug with MeOH: DCM (5:95). The crude product was purified by flash chromatography on silica gel (DCM:MeOH) to afford the pure

[9] F. Romanov-Michailidis, C. Besnard and A. Alexakis; *Org. let.*, 2012, **14**, 4906-4909.

compound 37 (35%, 0.201 g) as a dark orange oil. **^{1}H NMR** (500 MHz, $CDCl_3$) δ 9.65 (s, 1H), 7.47 (dd, J = 7.5, 2.0 Hz, 2H), 7.40 (dd, J = 4.9, 2.3 Hz, 3H), 5.48 (s, 2H), 4.48 (s, 2H), 3.10 (s, 2H), 2.80 (s, 2H). **^{13}C NMR** (101 MHz, $CDCl_3$) δ 162.7, 139.4, 132.3, 129.7, 129.5, 129.3, 56.6, 47.3, 26.7, 21.8. **HRMS** calcd for $C_{12}H_{14}N_3BF_4$ [M-BF_4]+: 200.1181, found 200.1188. **IR** (cm-1): 1730, 1589, 1294, 1047, 1032, 750, 727, 706, 521

Starting material and Product Characterization Data

General Procedure A: Intermolecular Benzoin Reaction

To a 12 mL zirconium oxide jar was added 50 zirconium oxide balls, the appropriate aldehyde (0.4 mmol, 2 equiv.), **NHC pre-catalyst 8** (10 mol %, 0.02 mmol), Cs_2CO_3 (10 mol %, 0.02 mmol), 2-propanol (100 µL) (if required) and sand (15 mass equiv.). Grinding was performed using a planetary mill with a rotation speed of 300 rpm for 15 minutes. After the grinding was complete, the crude mixture was washed out of the vessel with EtOAc (10 mL) before filtration and concentration *in vacuo*. The crude product was purified by flash column chromatography using silica gel and a petrol:EtOAc eluent system.

1,2-bis(4-chlorophenyl)-2-hydroxyethan-1-one (11)

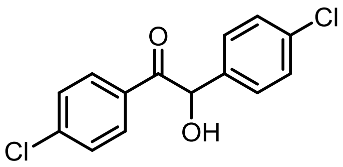

The title compound was prepared using general procedure A as a white solid (72%, 40 mg); **mp** 88 - 90 ˚C, HRMS calcd for $C_{14}H_{11}O_2Cl_2$ [M-H]+: 278.9985, found 278.9988, **^{1}H NMR** (500 MHz, $CDCl_3$) δ 7.82 (d, J = 8.7 Hz, 2H), 7.38 (d, J = 8.8 Hz, 2H), 7.30 (d, J = 8.6 Hz, 2H), 7.25 (d, J = 8.5 Hz, 2H), 5.88 (s, 1H), 4.49 (s, 1H). **^{13}C NMR** (126 MHz, $CDCl_3$) δ 197.6, 140.9, 137.3, 135.0, 131.7, 130.6, 129.6, 129.4, 129.2, 75.6.

Characterisation data is in accordance with previous report.[10]

2-hydroxy-1,2-diphenylethan-1-one (14)

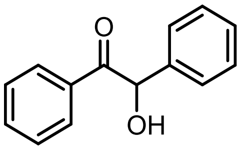

The title compound was prepared using general procedure A as a white solid (76%, 32 mg); **mp** 134 - 137 ˚C, **^{1}H NMR** (500 MHz, $CDCl_3$) δ 7.92 (dd, *J* = 8.3, 1.2 Hz, 2H), 7.52 (dd, *J* = 10.6, 4.3 Hz, 1H), 7.40 (t, *J* = 7.8 Hz, 2H), 7.36 – 7.30 (m, 4H), 7.30 – 7.24 (m, 1H), 5.96 (s, 1H), 4.56 (s, 1H). **^{13}C NMR** (126 MHz, $CDCl_3$) δ 199.1, 139.1, 134.1, 133.6, 129.3, 129.3, 128.8, 128.7, 127.9, 76.4. **HRMS** calcd for $C_{14}H_{13}O_2$ [M-H]+: 213.0916, found 213.0915

Characterisation data is in accordance with previous report.[10]

[10] Y. Tachibana, N. Kihara, and T. Takata; *Tetrahedron*, 2018, **68**, 894-899.

2-hydroxy-1,2-di-p-tolylethan-1-one (12)

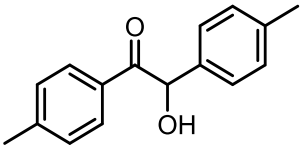

The title compound was prepared using general procedure A as a white solid (63%, 30 mg); **mp** 81 - 83 ˚C, **^{1}H NMR** (400 MHz, $CDCl_3$) δ 7.81 (d, J = 7.6 Hz, 2H), 7.20 (t, J = 9.1 Hz, 4H), 7.12 (d, J = 7.8 Hz, 2H), 5.89 (s, 1H), 2.35 (s, 3H), 2.28 (s, 3H). **^{13}C NMR** (126 MHz, $CDCl_3$) δ 198.7, 145.0, 138.5, 136.5, 131.1, 129.9, 129.5, 129.4, 127.8, 75.9, 21.9, 21.3. **HRMS** calcd for $C_{16}H_{17}O_2$ [M-H]+: 241.1229, found 241.1232

Characterisation data is in accordance with previous report.[10]

1,2-bis(4-fluorophenyl)-2-hydroxyethan-1-one (15)

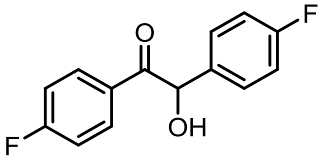

The title compound was prepared using general procedure A as a white solid (82%, 41 mg); **mp** 75 - 77 ˚C, **^{1}H NMR** (400 MHz, $CDCl_3$) δ 7.98 – 7.88 (m, 4H), 7.30 (dd, *J* = 8.7, 5.2 Hz, 4H), 7.05 (dt, *J* = 24.3, 8.8 Hz, 8H), 5.89 (s, 2H), 4.51 (s, 1H). **^{13}C NMR** (126 MHz, $CDCl_3$) δ 197.3 (s), 165.5 (d, J = 422.0 Hz), 163.5 (d, J = 413.0 Hz), 134.3 (s), 134.9 (s), 132.0 (d, J = 9.5 Hz), 129.7 (d, J = 8.4 Hz), 116.4 (d, J = 21.1 Hz), 116.2 (d, J = 21.5 Hz), 75.5 (s). **^{19}F NMR** (376 MHz, CDCl3) δ -102.5, -112.6. **HRMS** calcd for $C_{14}H_9O_2F_2$ [M-H]+: 247.0571, found 247.0596

Characterisation data is in accordance with previous report.[10]

2-hydroxy-1,2-bis(4-methoxyphenyl)ethan-1-one (13)

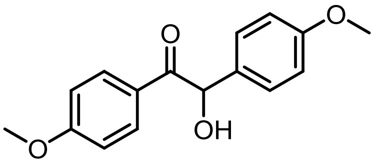

The title compound was prepared using general procedure A as a white solid (12%, 6.5 mg); **mp** 115 - 116 ˚C, **^{1}H NMR** (400 MHz, $CDCl_3$) δ 7.96 – 7.86 (m, 2H), 7.29 – 7.22 (m, 2H), 6.91 – 6.81 (m, 4H), 5.85 (d, *J* = 4.9 Hz, 1H), 4.59 (d, *J* = 5.7 Hz, 1H), 3.82 (s, 3H), 3.75 (s, 3H). **^{13}C NMR** (101 MHz, $CDCl_3$) δ 197.5, 164.1, 159.8, 132.0, 131.7, 129.1, 126.4, 114.6, 114.1, 75.4. **HRMS** calcd for $C_{16}H_{17}O_4$ [M-H]+: 273.1127, found 273.1123

Characterisation data is in accordance with previous report.[10]

2-hydroxy-1,2-bis(4-(trifluoromethyl)phenyl)ethan-1-one (16)

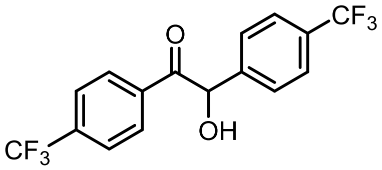

The title compound was prepared using general procedure A as a white solid (50%, 35 mg); **mp** 84 - 86 ˚C **^{1}H NMR** (500 MHz, $CDCl_3$) δ 8.00 (dd, J = 8.8, 0.7 Hz, 2H), 7.69 (dd, J = 8.8, 0.6 Hz, 2H), 7.60 (d, J = 8.1 Hz, 2H), 7.46 (d, J = 8.2 Hz, 2H), 6.02 (s, 1H), 4.49 (s, 1H). **^{13}C NMR** (126 MHz, $CDCl_3$) δ 197.8 (s), 142.1 (s), 136.1 (s), 135.6 (q, J = 33.1 Hz), 131.3 (q, J = 32.7 Hz), 129.5 (s), 128.2 (s), 126.5 (q, J = 3.7 Hz), 126.1 (q, J = 3.7 Hz), 123.9 (q, J = 272.3 Hz), 123.4 (q, J = 273.0 Hz), 76.1 (s). **^{19}F NMR** (376 MHz, CDCl3) δ -62.83, -63.42. **HRMS** calcd for $C_{16}H_9O_4F_6$ [M-H]+: 347.0507, found 347.0505

Characterisation data is in accordance with previous report.[10]

General Procedure B: Synthesis of 2-(2-oxo-2-phenylethoxy)benzaldehyde Derivatives

The title compounds were prepared using a method modified from the literature.[11] To a round bottom flask fitted with a condenser was added a magnetic stirrer, the appropriate salicylaldehyde (1 equiv.) in MeOH (0.2 M), trimethyl orthoformate (4.5 equiv.), and para-toluenesulfonic acid (0.01 equiv.). The mixture was then stirred under N_2 at 50 ˚C for 72 hours, before being cooled to room temperature. Sodium hydride (60% dispersion in paraffin oil, 0.08 equiv.) was then added and the mixture was concentrated *in vacuo*. The crude material was then dissolved in EtOAc and saturated aq. $NaHCO_3$, and the aqueous layer was extracted with ethyl acetate. The combined organic layers were then washed with saturated aq. NaCl, dried over $MgSO_4$ and concentrated *in vacuo* to give the product as an oil that was used immediately in the next step without further purification. The oil (1 equiv.) was dissolved in acetone (1.2 M), and to it was added K_2CO_3 (0.8 equiv.) and the mixture was stirred for 30 minutes. Then 2-bromo-1-phenylethan-1-one (0.8 equiv.) was added, and the mixture was stirred at room temperature overnight. Upon reaction completion, as monitored by TLC, water (10 mL) was added, and then the mixture was extracted with EtOAc. The combined organic extracts were then washed with brine, dried over $MgSO_4$ and concentrated *in vacuo*. The crude product was then purified by flash silica column chromatography to give a mixture of both the deprotected aldehyde and acetal in a given ratio. This mixture was then dissolved in a 4:1 mixture of THF/H_2O and to it was added para-toluenesulfonic acid (0.1 equiv.) and the solution was heated to 80 ˚C for 2 hours. The mixture was then cooled to room temperature before it was extracted with DCM. The combined organic layers were washed with saturated aq. $NaHCO_3$ and saturated aq. NaCl, dried over $MgSO_4$, and then concentrated *in vacuo* to give the product without further purification required.

[11] V. Tran, and T. Minehan; *Org. Lett.*, 2012, **14**, 6100-6103.

2-(2-oxo-2-phenylethoxy)benzaldehyde (17a)

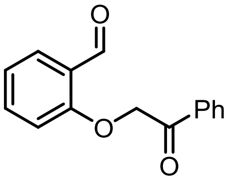

The title compound was prepared using general procedure B as a pale yellow solid (2.21 g, 89%); **mp** 106 – 108 ˚C, Rf = 0.18 (15% EtOAc in petrol); **^{1}H NMR** (500 MHz, $CDCl_3$) δ: 10.59 (s, 1H), 8.04 – 7.96 (m, 2H), 7.88 (dd, J = 7.7, 1.8 Hz, 1H), 7.65 (t, J = 7.4 Hz, 1H), 7.51 (dt, J = 9.5, 4.8 Hz, 3H), 7.08 (t, J = 7.5 Hz, 1H), 6.87 (d, J = 8.4 Hz, 1H), 5.43 (s, 2H). **^{13}C NMR** (126 MHz, $CDCl_3$) δ: 193.7, 189.7, 160.5, 135.9, 134.4, 134.4, 129.2, 128.9, 128.2, 125.5, 121.9, 112.9, 71.1.

Characterisation data is in accordance with previous report.[12]

5-methyl-2-(2-oxo-2-phenylethoxy)benzaldehyde (19a)

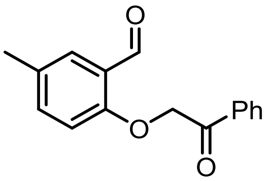

The title compound was prepared according to general procedure B as a pale yellow solid (812 mg, 40%); **mp** 102 – 105 ˚C, Rf = 0.23 (15% EtOAc in petrol); **^{1}H NMR** (500 MHz, $CDCl_3$) δ 10.55 (s, 1H), 8.01 – 7.95 (m, 2H), 7.67 (d, J = 2.1 Hz, 1H), 7.64 (s, 1H), 7.51 (t, J = 7.8 Hz, 2H), 7.30 (dd, J = 8.5, 2.3 Hz, 1H), 6.78 (d, J = 8.5 Hz, 1H), 5.39 (s, 2H), 2.31 (s, 3H). **^{13}C NMR** (126 MHz, $CDCl_3$) δ 194.0, 189.9, 158.6, 136.5, 134.5, 134.3, 131.4, 129.1, 128.9, 128.2, 125.2, 112.9, 71.2, 20.4.

Characterisation data is in accordance with previous report.[12]

5-nitro-2-(2-oxo-2-phenylethoxy)benzaldehyde (18a)

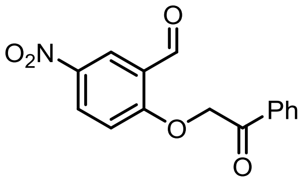

The title compound was prepared according to general procedure B as an orange solid (1.09 g, 76%); **mp** 159 – 162 ˚C, Rf = 0.09 (15% EtOAc in petrol), **^{1}H NMR** (500 MHz, DMSO) δ 10.47 (s, 1H), 8.53 – 8.41 (m, 2H), 8.04 (dd, J = 8.2, 1.1 Hz, 2H), 7.73 (dd, J = 10.6, 4.3 Hz, 1H), 7.61 (t, J = 7.8 Hz, 2H),

[12] Y. Li, Z. Feng and S. You; *Chem. Commun.*, 2008, 2263-2265.

7.47 (d, J = 9.0 Hz, 1H), 6.05 (s, 2H). **^{13}C NMR** (126 MHz, DMSO) δ 193.1, 187.9, 164.6, 141.2, 134.2, 133.9, 130.6, 128.9, 128.0, 124.1, 123.4, 115.5, 71.7.

Characterisation data is in accordance with previous report.[12]

General Procedure C: Intramolecular Benzoin Reaction

To a 12 mL zirconium oxide jar was added 50 zirconium oxide balls, the appropriate keto-aldehyde (0.2 mmol), NHC pre-catalyst 8 (10 mol %, 0.02 mmol), Cs_2CO_3 (10 mol %, 0.02 mmol), and sand (15 mass equiv.). Grinding was performed using a planetary mill with a rotation speed of 300 rpm for 15 minutes. After the grinding was complete, the crude mixture was washed out of the vessel with EtOAc (10 mL) before filtration and concentration *in vacuo*. The crude product was purified by flash column chromatography using silica gel and a petrol:EtOAc eluent system.

3-hydroxy-3-phenylchroman-4-one (17)

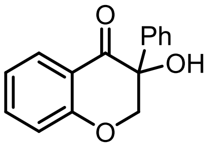

The title compound was prepared according to general procedure C, as a colourless solid (33.9 mg, 70%); **mp** 79 – 81 ˚C, 31 Rf = 0.5 (20% EtOAc in petrol), **^{1}H NMR** (500 MHz, $CDCl_3$) δ 7.94 (dd, J = 7.9, 1.7 Hz, 1H), 7.52 (ddd, J = 8.6, 7.2, 1.7 Hz, 1H), 7.49 – 7.43 (m, 2H), 7.36 – 7.27 (m, 3H), 7.10 – 7.05 (m, 1H), 6.97 (d, J = 8.4 Hz, 1H), 4.86 (d, J = 11.6 Hz, 1H), 4.48 (d, J = 11.6 Hz, 1H), 4.15 (s, 1H). **^{13}C NMR** (126 MHz, $CDCl_3$) δ 194.7, 161.7, 138.7, 137.0, 129.0, 128.9, 127.8, 126.2, 122.2, 119.3, 118.2, 73.9, 73.5.

Characterisation data is in accordance with previous report.[12]

3-hydroxy-6-methyl-3-phenylchroman-4-one (19)

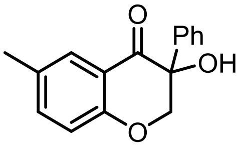

The title compound was prepared according to general procedure C as a colourless solid (40.9 mg, 80%); **mp** 92 – 95 ˚C, Rf = 0.5 (20% EtOAc in petrol); **^{1}H NMR** (500 MHz, $CDCl_3$) δ 7.75 – 7.68 (m, 1H), 7.51 – 7.42 (m, 2H), 7.36 – 7.28 (m, 4H), 6.87 (d, J = 8.5 Hz, 1H), 4.82 (d, J = 11.6 Hz, 1H), 4.44 (d, J = 11.6 Hz, 1H), 4.15 (s, 1H), 2.32 (s, 3H). **^{13}C NMR** (126 MHz, $CDCl_3$) δ 194.9, 159.8, 138.8, 138.2, 131.7, 128.9, 128.8, 127.2, 126.2, 118.9, 118.0, 74.0, 73.5, 20.5.

Characterisation data is in accordance with previous report.[12]

3-hydroxy-6-nitro-3-phenylchroman-4-one (18)

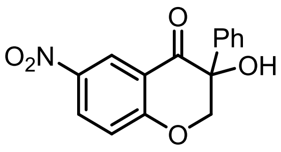

The title compound was prepared according to general procedure C as a colourless solid (35.0 mg, 62%). **mp** 147 – 150 ˚C, Rf = 0.23 (20% EtOAc in petrol); **^{1}H NMR** (500 MHz, $CDCl_3$) δ 8.83 (d, J = 2.8 Hz, 1H), 8.35 (dd, J = 9.2, 2.8 Hz, 1H), 7.47 – 7.40 (m, 2H), 7.36 (tt, J = 4.1, 2.2 Hz, 3H), 7.10 (d, J = 9.2 Hz, 1H), 5.03 (d, J = 12.0 Hz, 1H), 4.57 (d, J = 12.0 Hz, 1H), 4.07 (s, 1H).**^{13}C NMR** (126 MHz, $CDCl_3$) δ 193.0, 165.2, 142.8, 137.2, 131.2, 129.6, 129.2, 126.1, 124.3, 119.5, 119.0, 73.9, 73.7. **HRMS** (NSI+) calculated for $C_{15}H_{11}NO_5Na$ [M+Na]+ : m/z 308.0529, found 308.0526. **IR** (cm^{-1}) 3416, 3080, 2980, 2926, 2887, 1701, 1618, 1581, 1526, 1479, 1441, 1346, 1337, 1277, 1217;

Characterisation data is in accordance with previous report.[12]

ethyl 4-bromobut-2-enoate (29a)

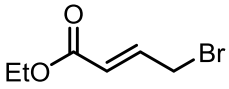

The title compound was prepared using a method modified from the literature.[13] To a suspension of N-bromosuccinimide (17.8 g, 100 mmol) in chlorobenzene (128 mL), ethyl crotonate (12.4 mL, 100 mmol) and benzoyl peroxide (0.121 g, 0.5 mmol) were added. The mixture was slowly heated to 85 °C over 1 hour. The mixture was left to stir for 2.5 days at this temperature. The mixture was then cooled, filtered and washed with diethyl ether (200 mL). The filtrate and washings were washed with a 5% aqueous NaOH solution (4 x 100 mL) and then with brine (2 x 50 mL). The combined organic layers were dried over $MgSO_4$, filtered and concentrated *in vacuo* to give the crude product as an orange oil. The chlorobenzene was removed by distillation (120 °C/ 300 mbar) and the desired product (at 200 °C/ 50 mbar) as a pale yellow oil. The product was measured to be 83% pure using ^{1}H NMR and was used without further purification.

General procedure D: synthesis of ethyl (E)-4-(2-formylphenoxy)but-2-enoate derivatives

The title compounds were prepared using a method modified from the literature.[14] Ethyl 4-bromobut-2-enoate (1 equiv.) and K_2CO_3 (1.5 equiv.) were added to a solution of salicylaldehyde derivative

[13] J. Laurenson, J. Parkinson, J. Percy, G. Rinaudo and R. Roig, *Beilstein J. Org. Chem.*, 2013, **9**, 2660-2668.
[14] K. Mantelingu, Y. Lin and D. Seidel, *Org. Lett.*, 2014, **16**, 5910-5913.

(1 equiv.) in acetone. The mixture was stirred overnight at room temperature. The reaction mixture was then passed through a plug of celite, washed with acetone (200 mL) and concentrated *in vacuo*. The resulting brown oil was washed with H_2O (4 x 100 mL) and extracted with EtOAc (3 x 100 mL). The combined organic phases were dried over $MgSO_4$, filtered and concentrated *in vacuo*. The crude product was purified by flash column chromatography using silica gel and a petrol:EtOAc eluent system.

ethyl (E)-4-(2-formylphenoxy)but-2-enoate (29b)

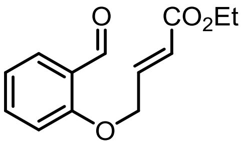

The title compound was prepared according to general procedure D as an off white solid (66%, 5.910 g); **mp** 64 - 67 °C. **^{1}H NMR** (500 MHz, $CDCl_3$) δ 10.56 (d, J = 0.7 Hz, 1H), 7.87 (dd, J = 7.7, 1.8 Hz, 1H), 7.60 – 7.42 (m, 1H), 7.17 – 7.03 (m, 2H), 6.94 (d, J = 8.4 Hz, 1H), 6.22 (dt, J = 15.8, 2.1 Hz, 1H), 4.84 (dd, J = 4.1, 2.1 Hz, 2H), 4.23 (q, J = 7.1 Hz, 2H), 1.31 (t, J = 7.1 Hz, 3H). **^{13}C NMR** (126 MHz, $CDCl_3$) δ 189.4, 166.0, 160.3, 141.3, 136.1, 129.0, 125.3, 122.7, 121.6, 112.7, 67.0, 60.9, 14.4. **HRMS** calcd for $C_{13}H_{14}O_4$ [M]+: 234.0892, found 234.0893 **IR** (cm^{-1}): 2688, 1710, 1597, 1481, 1460, 1438, 1302, 1426, 1182, 1157, 970, 757.

Characterisation data is in accordance with previous report.[15]

ethyl (E)-4-(2-formyl-4-methylphenoxy)but-2-enoate (30a)

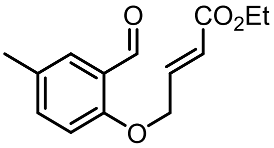

The title compound was prepared according to general procedure D as an off white solid (63%, 1.56 g); **mp** 58 - 60 °C **^{1}H NMR** (500 MHz, $CDCl_3$) δ 10.52 (s, 1H), 7.66 (s, 1H), 7.34 (d, J = 8.5 Hz, 1H), 7.09 (d, J = 17.4 Hz, 1H), 6.84 (d, J = 8.5 Hz, 1H), 6.20 (d, J = 15.9 Hz, 1H), 4.80 (s, 2H), 4.22 (q, J = 7.1 Hz, 2H), 2.32 (s, 3H), 1.31 (t, J = 7.1 Hz, 3H). **^{13}C NMR** (126 MHz, $CDCl_3$) δ 189.6, 166.0, 158.5, 141.5, 136.7, 131.1, 129.0, 125.0, 122.6, 112.7, 67.1, 60.9, 20.4, 14.4. **HRMS** calcd for $C_{14}H_{16}O_4$ [M+Na]+: 271.0942, found 271.0946; **IR** (cm^{-1}): 2970, 2359, 1740, 1361, 1229, 1217

Characterisation data is in accordance with previous report.[15]

[15] T. Ema, Y. Nanjo, S. Shiratori, Y. Terao, and R Kimura; *Org. Lett.*, 2016, **18**, 5764-5767.

ethyl (E)-4-(4-bromo-2-formylphenoxy)but-2-enoate (31a)

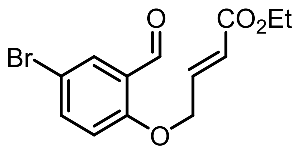

The title compound was prepared according to general procedure D as an off white solid (39%, 1.22 g); **mp** 72 - 74 °C; **^{1}H NMR** (500 MHz, $CDCl_3$) δ 10.46 (s, 1H), 7.95 (d, J = 2.4 Hz, 1H), 7.62 (dd, J = 8.8, 2.5 Hz, 1H), 7.07 (dt, J = 15.8, 4.1 Hz, 1H), 6.85 (d, J = 8.9 Hz, 1H), 6.18 (d, J = 14.4 Hz, 1H), 4.86-4.78 (m, 2H), 4.23 (q, J = 7.1 Hz, 2H), 1.31 (t, J = 7.1 Hz, 3H); **^{13}C NMR** (126 MHz, $CDCl_3$) δ 187.9, 165.8 159.2, 140.7, 138.4, 131.6, 126.5, 123.0, 114.7, 114.4, 67.4, 61.0, 14.3; **HRMS** calcd for $C_{13}H_{13}BrO_4$ [M+Na]+: 334.9893, found 334.9895; **IR** (cm^{-1}): 2970, 2866, 2359, 1709, 1676, 1668, 1474, 1445, 1387, 1366, 1306, 1229 1178, 1126, 1059, 1086, 1028, 964, 945, 837, 814, 669

Characterisation data is in accordance with previous report.[15]

General Procedure E: Synthesis of Ethyl Dimethyl 2-(2-formylphenoxy)maleate Derivatives

The title compounds were prepared using a method modified from the literature.[16] DMAD (1 equiv.), substituted salicylaldehyde (1.1 equiv.), DABCO (0.2 equiv.) and dry DCM were added to a flask. The mixture was stirred at room temperature for 15 minutes under N_2 after which it was concentrated *in vacuo* and the resulting crude product was purified by flash column chromatography using silica gel and a petrol:EtOAc eluent system.

dimethyl 2-(2-formylphenoxy)maleate (32a)

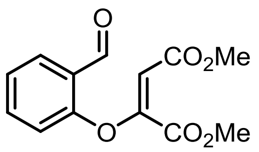

The title compound was prepared according to general procedure E as a viscous colourless oil (99%, 2.6 g, E/Z 90:10); **^{1}H NMR** (400 MHz, $CDCl_3$) δ 10.48 (E, s, 0.1H), 10.22 (Z, s, 1H), 7.90 (E, dd, J = 7.8, 1.7 Hz, 1H), 7.84 (Z, dd, J = 7.7, 1.7 Hz, 0.1H), 7.65 – 7.59 (E, m, 1H), 7.47 – 7.42 (Z, m, 0.1H), 7.35 (E, t, J = 7.6 Hz, 1H), 7.16 (E, d, J = 8.2 Hz, 1H), 7.12 (Z, d, J = 7.6 Hz, 0.1H), 6.79 (Z, d, J = 8.3 Hz, 0.1H), 6.69 (Z, s, 0.1H), 5.18 (E, s, 1H), 3.86 (E, s, 3H), 3.72 (Z, s, 0.3H), 3.65 (Z, s, 0.3H), 3.63 (E, s, 3H); **^{13}C NMR** (101 MHz, $CDCl_3$) δ 187.9, 165.3, 162.7, 160.0, 155.3, 136.1, 129.4, 127.9, 127.0, 121.8, 101.8, 53.4, 52.2. (All values reported for ^{13}C NMR are for the E isomer) **HRMS** calcd for $C_{13}H_{12}O_6$ [M+Na]+: 287.0544, found 287.0532; **IR** (cm^{-1}): 2970, 1740, 1695, 1635, 1600, 1577, 1479, 1456, 1436, 1363, 1273, 1273, 1124, 1091, 1028, 858, 785, 632

[16] P. Wang, Z. Li, S. Cao and H. Rao, *RSC Adv.*, 2015, **5**, 106350-106354.

Characterisation data is in accordance with previous report.[17]

dimethyl 2-(2-formyl-4-methylphenoxy)maleate (33a)

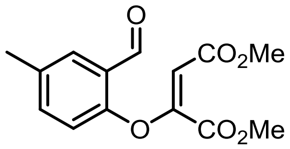

The title compound was prepared according to general procedure E as a viscous pale yellow oil (36%, 1.00 g, E/Z 90:10).

^{1}H NMR (500 MHz, $CDCl_3$) δ 10.50 (Z, s, 0.1H), 10.22 (E, s, 1H), 7.75 (E, s, 1H), 7.70 (Z, s, 0.1H), 7.45 (E, d, J = 8.0 Hz, 1H), 7.28 (Z, d, J = 8.4 Hz, 0.1H), 7.08 (E, d, J = 8.3 Hz, 1H), 6.73 (Z, d, J = 8.3 Hz, 0.1H), 5.29 (Z, s, 0.1H), 5.17 (E, s, 1H), 3.92 (E, s, 3H), 3.75 (Z, s, 0.3H), 3.71 (Z, s, 0.3H), 3.68 (E, s, 3H), 2.40 (E, s, 3H), 2.33 (Z, s, 0.3H). **^{13}C NMR** (126 MHz, $CDCl_3$) δ (Z, 189.3), (E, 188.0), (E, 165.4), (Z, 163.7), (E, 162.8), (Z, 162.3), (E, 160.6), (Z, 157.0), (E, 153.1), (Z, 149.4), (E, 137.2), (E, 136.82), (Z, 136.3), (Z, 133.6), (E, 129.4), (Z, 128.7), (E, 127.53), (Z, 125.5), (E, 121.7), (Z, 116.5), (Z, 115.5), (E, 101.0), (Z, 53.6), (E, 53.4), (Z, 52.3), (E, 52.0), (E, 20.9), (Z, 20.6); **HRMS** calcd for $C_{14}H_{14}O_6$ [M-H]+: 279.0865, found 279.0869; **IR** (cm^{-1}): 2953, 2851, 2278, 1740, 1715, 1686, 1636, 1491, 1437, 1371, 1308, 1279, 1234, 1199, 1171, 1155, 1132, 1032, 849, 833, 764, 745, 702, 640, 627

Characterisation data is in accordance with previous report.[17]

dimethyl 2-(4-bromo-2-formylphenoxy)maleate (34a)

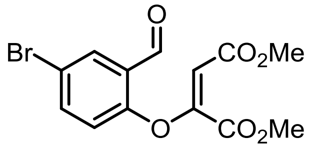

The title compound was prepared according to general procedure E as a white solid (58%, 1.11 g,); **mp** 41-43 °C, **^{1}H NMR** (500 MHz, $CDCl_3$) δ 10.22 (s, 1H), 8.07 (d, J = 2.3 Hz, 1H), 7.75 (dd, J = 8.6, 2.4 Hz, 1H), 7.10 (d, J = 8.6 Hz, 1H), 5.33 (s, 1H), 3.91 (s, 3H), 3.71 (s, 3H). **^{13}C NMR** (126 MHz, $CDCl_3$) δ 186.5, 165.0, 162.3, 158.8, 154.4, 138.8, 132.1, 129.1, 123.3, 120.4, 103.4, 53.5, 52.3. **HRMS** calcd for $C_{13}H_{11}O_6Br$ [M+H]+: 342.9828, found 342.9817 **IR** (cm^{-1}): 2900.924, 2358.94, 1747.51, 1712.79, 1685.79, 1645.28, 1627.92, 1467.83, 1436.97, 1361.74, 1303.88, 1247.94, 1192.01, 1163.08, 1126.43, 1031.92, 1014.56, 827.46, 817.82

Characterisation data is in accordance with previous report.[17]

[17] C. M. Filloux, S. P. Lathrop, and T. Rovis; *PNAS.*, 2010, **107**, 20667-20671.

General Procedure F: Intramolecular Stetter Reaction

To a 12 mL zirconium oxide jar was added 50 zirconium oxide balls, the appropriate Substituted salicylaldehyde starting material (0.2 mmol), NHC pre-catalyst 8 (5 mol %, 0.02 mmol), Cs_2CO_3 (5 mol%, 0.01 mmol), and sand (15 mass equiv.). Grinding was performed using a planetary mill with a rotation speed of 300 rpm for 15 minutes. After the grinding was complete, the crude mixture was washed out of the vessel with EtOAc (10 mL) before filtration and concentration *in vacuo*. The crude product was purified by flash column chromatography using silica gel and a petrol:EtOAc eluent system.

ethyl 2-(4-oxochroman-3-yl)acetate (29)

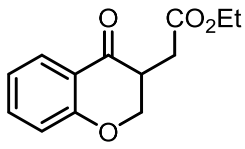

The title compound was prepared according to general procedure F as a pale yellow oil (91%, 43.0 mg) as a pale yellow oil. **^{1}H NMR** (500 MHz, $CDCl_3$) δ 7.89 (dd, J = 7.9, 1.6 Hz, 1H), 7.48 (m, 1H), 7.06 – 6.99 (m, 1H), 6.97 (d, J = 8.4 Hz, 1H), 4.60 (dd, J = 11.2, 5.3 Hz, 1H), 4.30 (t, J = 11.6 Hz, 1H), 4.24 – 4.13 (m, 2H), 3.33 (ddt, J = 13.2, 8.2, 5.1 Hz, 1H), 2.93 (dd, J = 16.9, 4.8 Hz, 1H), 2.41 (dd, J = 16.9, 8.2 Hz, 1H), 1.28 (t, J = 7.1 Hz, 3H). **^{13}C NMR** (126 MHz, $CDCl_3$) δ 192.8, 171.5, 161.9, 136.2, 127.5, 121.7, 120.6, 118.0, 70.4, 61.1, 42.7, 30.5, 14.3. **HRMS** calcd for $C_{12}H_{12}O_4$ [M]+: 234.0892, found 234.0893 **IR** (cm^{-1}): 2982, 1730, 1686, 1605, 1479, 1466, 1456, 1373, 1323, 1296, 1213, 1175, 1148, 1036, 1011, 957, 926, 756

Characterisation data is in accordance with previous report.[15]

ethyl 2-(6-methyl-4-oxochroman-3-yl)acetate (30)

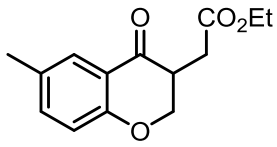

The title compound was prepared according to general procedure F as a pale yellow oil (88%, 44.3 mg); **^{1}H NMR** (500 MHz, $CDCl_3$) δ 7.67 (d, J = 1.5 Hz, 1H), 7.28 (dd, J = 8.3, 2.0 Hz, 1H), 6.87 (d, J = 8.4 Hz, 1H), 4.56 (dd, J = 11.1, 5.2 Hz, 1H), 4.26 (t, J = 11.5 Hz, 1H), 4.21 – 4.13 (m, 2H), 3.30 (m, 1H), 2.92 (dd, J = 16.9, 4.8 Hz, 1H), 2.40 (dd, J = 16.9, 8.3 Hz, 1H), 2.30 (s, 3H), 1.28 (t, J = 7.1 Hz, 3H). **^{13}C NMR** (126 MHz, $CDCl_3$) δ 193.0, 171.6, 159.9, 137.2, 131.1, 127.1, 120.2, 117.7, 70.4, 61.1, 42.7, 30.6, 20.5, 14.3. **HRMS** calcd for $C_{13}H_{14}O_4$ [M+H]+: 249.1132, found 249.1127 **IR** (cm^{-1}): 2890, 2278, 1717,1689, 1609, 1496, 1375, 1296, 1219, 1010, 824, 548

Characterisation data is in accordance with previous report.[15]

ethyl 2-(6-bromo-4-oxochroman-3-yl)acetate (31)

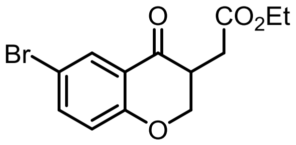

The title compound was prepared according to general procedure F as a pale yellow oil (95%, 59.1 mg); **^{1}H NMR** (500 MHz, $CDCl_3$) δ 7.98 (d, J = 2.2 Hz, 1H), 7.54 (dd, J = 8.8, 2.3 Hz, 1H), 6.88 (d, J = 8.8 Hz, 1H), 4.60 (dd, J = 11.2, 5.3 Hz, 1H), 4.29 (t, J = 11.7 Hz, 1H), 4.21 – 4.14 (m, 2H), 3.31 (td, J = 12.7, 5.0 Hz, 1H), 2.91 (dd, J = 17.1, 4.7 Hz, 1H), 2.43 (dd, J = 17.0, 8.1 Hz, 1H), 1.28 (t, J = 7.1 Hz, 3H). **^{13}C NMR** (126 MHz, $CDCl_3$) δ 191.6, 171.3, 160.8, 138.8, 129.9, 121.9, 120.1, 114.3, 70.5, 61.2, 42.4, 30.4, 14.3. **HRMS** calcd for $C_{12}H_{11}O_4Br$ [M+H]+: 313.0068, found 313.0075, **IR** (cm^{-1}): 2980, 1730, 1692, 1599, 1474, 1416, 1373, 1275, 1165, 1015, 822, 532

Characterisation data is in accordance with previous report.[15]

methyl 2-(2-methoxy-2-oxoethyl)-3-oxo-2,3-dihydrobenzofuran-2-carboxylate (32)

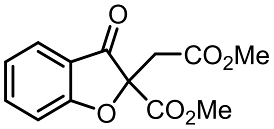

The title compound was prepared according to general procedure F as a white crystalline solid (90%, 51.8 mg); **mp** 102 - 105 °C, **^{1}H NMR** (400 MHz, $CDCl_3$) δ 7.72 – 7.65 (m, 2H), 7.24 (dd, J = 8.4, 0.7 Hz, 1H), 7.19 – 7.13 (m, 1H), 3.78 (s, 3H), 3.66 (s, 3H), 3.49 (d, J = 17.5 Hz, 1H), 3.11 (d, J = 17.5 Hz, 1H). **^{13}C NMR** (126 MHz, $CDCl_3$) δ 194.7, 172.4, 169.0, 165.7, 138.8, 125.2, 123.1, 119.6, 113.8, 88.1, 53.8, 52.4, 38.6. **HRMS** calcd for $C_{13}H_{12}O_6$ [M+H]: 287.0540, found 287.0532 **IR** (cm^{-1}): 2878, 2359, 1736, 1717, 1609, 1456, 1439, 1366, 1296, 1279, 1196, 1140, 1043, 995, 959, 901, 847, 768

Characterisation data is in accordance with previous report.[17]

methyl 2-(2-methoxy-2-oxoethyl)-5-methyl-3-oxo-2,3-dihydrobenzofuran-2-carboxylate (33)

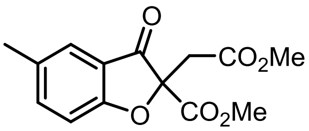

The title compound was prepared according to general procedure F as a yellow solid (90%, 52.6 mg) **mp** 80 - 84 °C; **^{1}H NMR** (500 MHz, $CDCl_3$) δ 7.47 (d, J = 8.1 Hz, 2H), 7.12 (d, J = 8.2 Hz, 1H), 3.76 (s, 3H), 3.65 (s, 3H), 3.46 (d, J = 17.4 Hz, 1H), 3.09 (d, J = 17.4 Hz, 1H), 2.36 (s, 3H). **^{13}C NMR** (126 MHz, $CDCl_3$) δ 194.7, 170.9, 169.1, 165.9, 140.0, 132.9, 124.5, 119.4, 113.3, 88.3, 53.8, 52.4, 38.6, 20.8. **HRMS** calcd for $C_{14}H_{14}O_6$ [M+Na]+: 301.0694, found 301.0688; **IR** (cm^{-1}): 2955, 2361, 1736, 1717, 1437, 1364, 1290, 1267, 1209, 1132, 1123, 1045, 991, 962, 860, 834, 791, 768, 669, 523

Characterisation data is in accordance with previous report.[17]

methyl 5-bromo-2-(2-methoxy-2-oxoethyl)-3-oxo-2,3-dihydrobenzofuran-2-carboxylate (34)

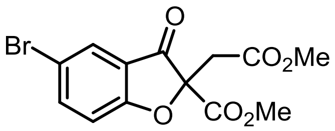

The title compound was prepared according to general procedure F as a white crystalline solid (85%, 58.8 mg); **mp** 104 - 108 °C; **^{1}H NMR** (500 MHz, $CDCl_3$) δ 7.81 (s, 1H), 7.73 (d, J = 8.8 Hz, 1H), 7.13 (d, J = 8.8 Hz, 1H), 3.77 (s, 3H), 3.65 (s, 3H), 3.45 (d, J = 17.6 Hz, 1H), 3.22 (d, J = 17.6 Hz, 1H). **^{13}C NMR** (126 MHz, $CDCl_3$) δ 193.5, 171.1, 168.8, 165.3, 141.2, 127.6, 121.6, 115.7, 115.4, 88.7, 54.0, 52.5, 38.5. **HRMS** calcd for $C_{13}H_{11}O_6Br$ [M+Na]+: 364.9640, found 364.9637; **IR** (cm^{-1}): 2890, 2279, 1728, 1605, 1466, 1458, 1637, 1217, 1169, 1128, 1063, 997, 841, 685, 667, 633, 521

Characterisation data is in accordance with previous report.[17]

General procedure G: Synthesis of Chalcone Derivatives

The title compounds were prepared using a method modified from the literature.[18] Substituted benzaldehyde (1 equiv.) was added to a solution of acetophenone (1.1 eqiuv.) in ethanol (2 M). The mixture was cooled to 0 °C in an ice bath and a solution of 5% aqueous NaOH (1 equiv.) was added dropwise resulting in precipitation. Once addition of the NaOH solution was complete, the mixture was stirred for an hour at this temperature. It was then filtered, washed with cold toluene, and dried. The solid was recrystallized from ethanol to give the desired compound.

(E)-1-phenyl-3-(p-tolyl)prop-2-en-1-one (23a)

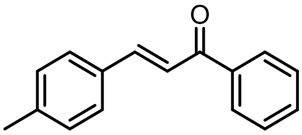

The title compound was prepared according to general procedure G as an off white solid (10%, 0.681g); **mp** 89 - 99 °C; **^{1}H NMR** (400 MHz, $CDCl_3$) δ 8.01 (m, 2H), 7.79 (d, J = 15.8 Hz, 1H), 7.61 – 7.46 (m, 6H), 7.29 – 7.14 (m, 2H), 2.39 (s, 3H). **^{13}C NMR** (126 MHz, $CDCl_3$) δ 190.8, 145.1, 141.3, 138.5, 132.8, 132.3, 129.9, 128.7, 128.6, 128.6, 121.3, 21.7. **HRMS** calcd for $C_{16}H_{14}O$ [M]+: 222.1049, found 222.1045. **IR** (cm^{-1}): 1655, 1592, 1566, 1331, 1207, 1179, 1016, 984, 816, 773, 693

Characterisation data is in accordance with previous report.[19]

---

[18] C. T. Salfeena, K. T. Ashtha, B.S. Asidhar., *Org. Biomol. Chem*., 2016,**14**, 10165-10169.

[19] D. Enders, J. Han, and A. Henseler; *Chem. Commun*., 2008, 3989-3991.

(E)-3-(4-chlorophenyl)-1-phenylprop-2-en-1-one (26a)

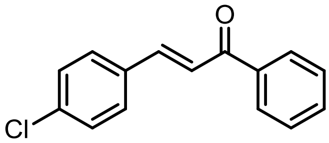

The title compound was prepared according to general procedure G as a yellow solid (42%, 3.06 g); **mp** 109 - 110 °C; **^{1}H NMR** (500 MHz, $CDCl_3$) δ 8.02 (d, J = 7.1 Hz, 2H), 7.76 (d, J = 15.7 Hz, 1H), 7.63 – 7.55 (m, 3H), 7.55 – 7.48 (m, 3H), 7.43 – 7.38 (m, 2H); **^{13}C NMR** (126 MHz, $CDCl_3$) δ 190.4, 143.5, 138.2, 136.6, 133.5, 133.1, 129.7, 129.4, 128.8, 128.7, 122.6. **HRMS** calcd for $C_{15}H_{11}OCl$ [M]+: 242.0495, found 242.0498. **IR** (cm^{-1}): 1657, 1589, 1487, 1402, 1292, 1215, 982, 820, 773, 683, 494

Characterisation data is in accordance with previous report.[19]

General Procedure H: Intramolecular Stetter Reaction

To a 12 mL zirconium oxide jar was added 50 zirconium oxide balls Chalcone (1 mmol), benzaldehyde derivative (1.5 mmol), K_3PO_4 (0.212 g, 1 mmol) and NHC pre-catalyst (0.2 mmol). The reaction was milled using the following settings (3 hours, X RPM, 12 cycles, 15 minutes per cycle, 2 minutes pause between cycles and a directional change). After the reaction the jars were washed with EtOAc (10 mL) and the crude mixture passed through a silica plug. The crude product was purified by flash column chromatography.

1,2,4-triphenylbutane-1,4-dione (20)

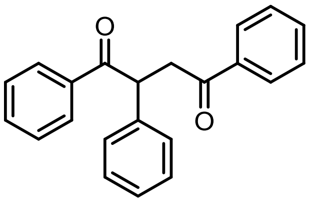

The title compound was prepared according to GP H as a white solid (81%, 0.254 g); **mp** 114 - 116 ˚C **^{1}H NMR** (500 MHz, $CDCl_3$) δ 8.04 (dd, *J* = 8.4, 1.3 Hz, 2H), 7.99 (dd, *J* = 8.4, 1.3 Hz, 2H), 7.60 – 7.52 (m, 1H), 7.52 – 7.47 (m, 1H), 7.47 – 7.27 (m, 8H), 7.25 – 7.20 (m, 1H), 5.33 (dd, *J* = 10.1, 3.7 Hz, 1H), 4.22 (dd, *J* = 18.0, 10.1 Hz, 1H), 3.31 (dd, *J* = 18.0, 3.7 Hz, 1H). **^{13}C NMR** (126 MHz, $CDCl_3$) δ 198.9, 198.1, 138.7, 136.5, 133.3, 132.9, 129.2, 129.0, 128.6, 128.5, 128.3, 128.2, 127.4, 48.7, 43.9. **HRMS** calcd for $C_{22}H_{19}O_2$ [M-H]+: 315.1385, found 315.1397

Characterisation data is in accordance with previous report.[20]

[20] Y. Liu, Y. Li, Y. Qi, and J. Wan; *Synthesis*, 2010, **24**, 4188-4192

1-(4-chlorophenyl)-2,4-diphenylbutane-1,4-dione (21)

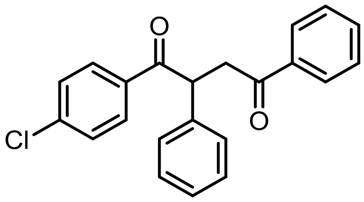

The title compound was prepared according to GP H as a yellow oil (81%, 0.281 g); **^{1}H NMR** (500 MHz, $CDCl_3$) δ 8.01 – 7.93 (m, 4H), 7.55 (d, J = 7.4 Hz, 1H), 7.45 (dd, J = 10.9, 4.5 Hz, 1H), 7.39 – 7.28 (m, 12H), 7.28 – 7.21 (m, 1H), 5.26 (dd, J = 10.2, 3.6 Hz, 1H), 4.21 (dd, J = 18.1, 10.2 Hz, 1H), 3.31 (dd, J = 18.1, 3.6 Hz, 1H). **^{13}C NMR** (126 MHz, $CDCl_3$) δ 198.0, 197.8, 139.3, 138.3, 136.4, 134.8, 133.4, 130.4, 129.3, 128.9, 128.6, 128.2, 127.6, 48.8, 43.9. **HRMS** calcd for $C_{22}H_{18}O_2Cl$ [M+H]+: 349.0995, found 349.0992

Characterisation data is in accordance with previous report.[19]

2,4-diphenyl-1-(p-tolyl)butane-1,4-dione (22)

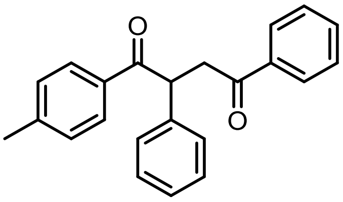

The title compound was prepared according to GP H as a white solid (78%, 0.256 g); **mp** 114 - 117 ˚C **^{1}H NMR** (500 MHz, $CDCl_3$) δ 7.98 (dd, *J* = 8.4, 1.3 Hz, 2H), 7.94 (d, *J* = 8.3 Hz, 2H), 7.58 – 7.52 (m, 1H), 7.44 (t, *J* = 7.7 Hz, 2H), 7.36 (dd, *J* = 8.2, 1.2 Hz, 2H), 7.30 (t, *J* = 7.6 Hz, 2H), 7.24 – 7.17 (m, 3H), 5.31 (dd, *J* = 9.9, 3.8 Hz, 1H), 4.20 (dd, *J* = 18.0, 10.0 Hz, 1H), 3.29 (dd, *J* = 18.0, 3.8 Hz, 1H), 2.36 (s, 3H). **^{13}C NMR** (126 MHz, $CDCl_3$) δ 198.5, 198.1, 143.7, 139.0, 136.6, 133.9, 133.2, 129.2, 129.2, 129.1, 128.6, 128.2, 128.2, 127.3, 48.6, 43.8, 21.6. **HRMS** calcd for $C_{23}H_{21}O_2$[M+H]+: 329.1542, found 329.1544

Characterisation data is in accordance with previous report.[20]

1,4-diphenyl-2-(p-tolyl)butane-1,4-dione (23)

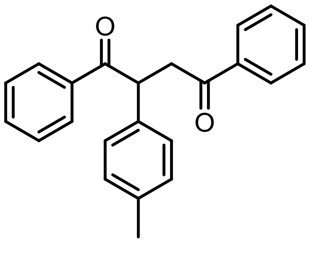

The title compound was prepared according to GP H as a white solid (47%, 0.153 g) **mp** 72 - 74 ˚C **^{1}H NMR** (500 MHz, $CDCl_3$) δ 7.96 – 7.83 (m, 4H), 7.44 (t, *J* = 7.4 Hz, 1H), 7.39 – 7.27 (m, 5H), 7.14 (d,

J = 7.2 Hz, 2H), 7.04 – 6.98 (m, 2H), 5.19 (dd, *J* = 10.1, 3.7 Hz, 1H), 4.09 (dd, *J* = 18.0, 10.1 Hz, 1H), 3.18 (dd, *J* = 18.0, 3.7 Hz, 1H), 2.18 (s, 3H). **^{13}C NMR** (126 MHz, $CDCl_3$) δ 199.1, 198.2, 137.0, 136.5, 136.5, 135.6, 133.2, 132.9, 129.9, 129.0, 128.6, 128.5, 128.2, 128.1, 48.3, 43.9, 21.0. **HRMS** calcd for $C_{23}H_{21}O_2$[M+H]+: 329.1542, found 329.1545

Characterisation data is in accordance with previous report.[20]

1-(4-chlorophenyl)-4-phenyl-2-(p-tolyl)butane-1,4-dione (24)

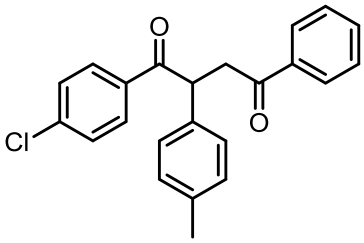

The title compound was prepared according to GP H as a white solid (33%, 0.120 g); **mp** 92-95 ˚C **^{1}H NMR** (500 MHz, $CDCl_3$) δ 7.92 – 7.85 (m, 4H), 7.48 (t, *J* = 7.4 Hz, 1H), 7.36 (t, *J* = 7.7 Hz, 2H), 7.29 (d, *J* = 8.5 Hz, 2H), 7.14 (d, *J* = 8.0 Hz, 2H), 7.05 (d, *J* = 8.0 Hz, 2H), 5.14 (dd, *J* = 10.1, 3.5 Hz, 1H), 4.11 (dd, *J* = 18.1, 10.2 Hz, 1H), 3.20 (dd, *J* = 18.1, 3.6 Hz, 1H), 2.22 (s, 3H). **^{13}C NMR** (126 MHz, $CDCl_3$) δ 198.1, 197.9, 139.3, 137.3, 136.4, 135.2, 134.8, 133.3, 130.4, 130.0, 128.8, 128.6, 128.2, 128.1, 48.4, 43.9, 21.1. **HRMS** calcd for $C_{23}H_{20}O_2Cl$[M+H]+: 363.1152, found 363.1150

Characterisation data is in accordance with previous report.[20]

4-phenyl-1,2-di-p-tolylbutane-1,4-dione (25)

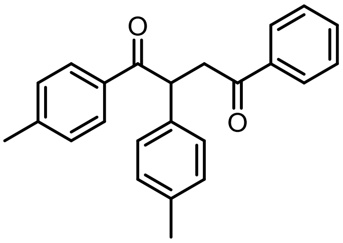

The title compound was prepared according to GP H as a white solid (32%, 0.110 g) **mp** 132 - 134 ˚C **^{1}H NMR** (500 MHz, $CDCl_3$) δ 8.02 – 7.92 (m, 4H), 7.58 – 7.51 (m, 1H), 7.47 – 7.41 (m, 2H), 7.26 (d, *J* = 8.1 Hz, 2H), 7.20 (d, *J* = 8.0 Hz, 2H), 7.12 (d, *J* = 7.9 Hz, 2H), 5.29 (dd, *J* = 9.9, 3.8 Hz, 1H), 4.19 (dd, *J* = 18.0, 10.0 Hz, 1H), 3.27 (dd, *J* = 18.0, 3.8 Hz, 1H), 2.35 (s, 3H), 2.29 (s, 3H). **^{13}C NMR** (126 MHz, $CDCl_3$) δ 198.7, 198.3, 143.7, 137.1, 136.7, 136.0, 134.0, 133.3, 130.0, 129.3, 129.2, 128.7, 128.3, 128.2, 48.3, 43.9, 21.7, 21.1. **HRMS** calcd for $C_{24}H_{23}O_2$ [M+H]+: 343.1698, found 343.1701

Characterisation data is in accordance with previous report.[20]

2-(4-chlorophenyl)-1,4-diphenylbutane-1,4-dione (26)

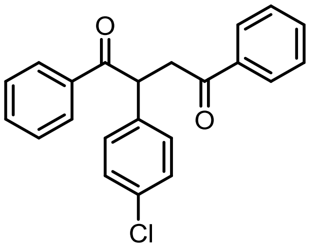

The title compound was prepared according to GP H as a white solid (42%, 0.147 g); **mp** 111 - 112 ˚C ^{1}**H NMR** (500 MHz, $CDCl_3$) δ 8.04 – 7.94 (m, 4H), 7.59 – 7.54 (m, 1H), 7.54 – 7.49 (m, 1H), 7.43 (dt, *J* = 18.1, 7.7 Hz, 4H), 7.32 – 7.26 (m, 4H), 5.31 (dd, *J* = 9.8, 4.0 Hz, 1H), 4.17 (dd, *J* = 18.0, 9.8 Hz, 1H), 3.30 (dd, *J* = 18.0, 4.0 Hz, 1H). ^{13}**C NMR** (126 MHz, $CDCl_3$) δ 198.7, 197.8, 137.1, 136.3, 136.2, 133.4, 133.4, 133.1, 129.6, 129.4, 128.9, 128.6, 128.6, 128.2, 48.0, 43.7. **HRMS** calcd for $C_{22}H_{18}O_2Cl$ [M+H]+: 349.0995, found 349.0989

Characterisation data is in accordance with previous report.[20]

1,2-bis(4-chlorophenyl)-4-phenylbutane-1,4-dione (27)

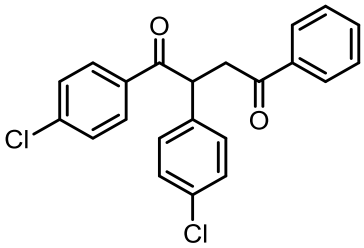

The title compound was prepared according to GP H as a white solid (69%, 0.299 g) **mp** 130 - 131 ˚C ^{1}**H NMR** (500 MHz, $CDCl_3$) δ 8.00 – 7.90 (m, 4H), 7.55 (d, *J* = 7.4 Hz, 1H), 7.45 (t, *J* = 7.8 Hz, 2H), 7.40 – 7.35 (m, 2H), 7.28 (s, 4H), 5.25 (dd, *J* = 10.0, 3.8 Hz, 1H), 4.17 (dd, *J* = 18.0, 10.0 Hz, 1H), 3.30 (dd, *J* = 18.0, 3.8 Hz, 1H). ^{13}**C NMR** (126 MHz, $CDCl_3$) δ 197.8, 197.6, 139.6, 136.8, 136.3, 134.7, 133.6, 133.6, 130.4, 129.6, 129.6, 129.0, 128.7, 128.2, 48.1, 43.8. **HRMS** calcd for $C_{22}H_{17}O_2Cl_2$ [M+H]+: 383.0606, found 383.0605

Characterisation data is in accordance with previous report.[20]

2-(4-chlorophenyl)-4-phenyl-1-(p-tolyl)butane-1,4-dione (28)

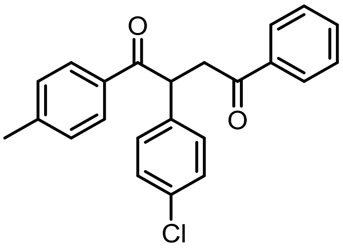

The title compound was prepared according to GP H as a white solid (32%, 0.116 g); **mp** 172 - 174 ˚C ^{1}**H NMR** (400 MHz, $CDCl_3$) δ 8.03 – 7.97 (m, 2H), 7.94 (d, *J* = 8.2 Hz, 2H), 7.62 – 7.54 (m, 1H), 7.47 (t, *J* = 7.6 Hz, 2H), 7.35 – 7.27 (m, 4H), 7.23 (d, *J* = 8.0 Hz, 2H), 5.32 (dd, *J* = 9.7, 4.0 Hz, 1H), 4.18 (dd, *J* = 18.0, 9.7 Hz, 1H), 3.31 (dd, *J* = 18.0, 4.1 Hz, 1H), 2.41 (d, *J* = 14.0 Hz, 3H). ^{13}**C NMR** (126 MHz, $CDCl_3$) δ 198.3, 197.9, 144.1, 137.5, 136.5, 133.7, 133.4, 133.3, 129.7, 129.4, 129.1, 128.7, 128.2, 47.9, 43.6, 21.7. **HRMS** calcd for $C_{23}H_{20}O_2Cl$ [M+H]+: 363.1152, found 363.1148

Characterisation data is in accordance with previous report.[20]

HPLC Traces and Optical Rotation Measurements

ethyl 2-(4-oxochroman-3-yl)acetate (29)

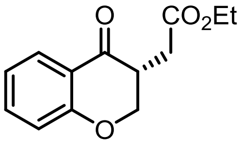

HPLC Chiralpak IC Hexane:IPA 90:10 0.5 mLmin^{-1} 100 min, $t_{r\ maj}$ = 30.9 min $t_{r\ min}$ = 35.9 min

$[\alpha]_D^{22}$ = -14.3˚ (MeCN, c = 1.01)

Racemic

	Inj. Number	Peak Name	R. Time	Area	Area %
1	1.00	*1	30.94	39346372.00	50.04
2	1.00	*2	35.86	39277596.00	49.96
3					

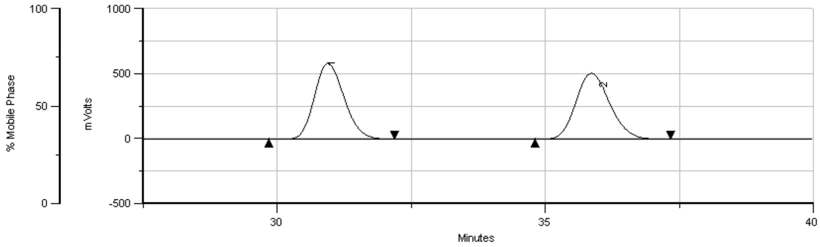

Enantio-enriched

	Inj. Number	Peak Name	R. Time	Area	Area %	Sample Descrip.
1	1.00	*1	32.03	10189560.0[illegible]	96.11	WN5.113
2	1.00	*2	37.24	4459701.00	3.89	WN5.113
3						

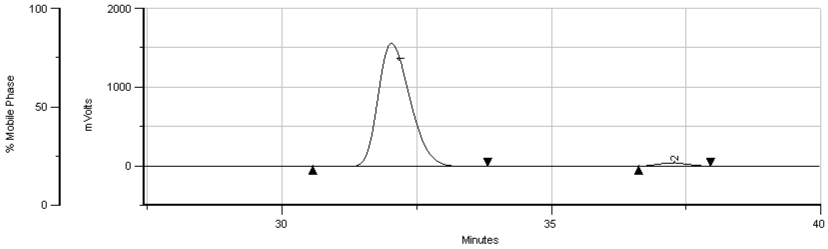

methyl 2-(2-methoxy-2-oxoethyl)-3-oxo-2,3-dihydrobenzofuran-2-carboxylate (32)

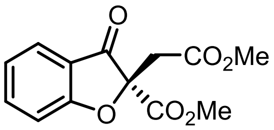

HPLC Chiralpak IC Hexane:IPA 60:40 1.0 mLmin^{-1} 60 min, $t_{r\ maj}$ = 49.8 min $t_{r\ min}$ = 50.2 min

$[\alpha]_D^{22}$ = -67.4° (MeCN, c = 1.04)

Racemic

Inj. Number	Peak Name	R. Time	Area	Area %	Sample Descrip.
1.00	*1	13.19	23899528.00	49.84	WNSI131
1.00	*2	26.28	24050222.00	50.16	WNSI131

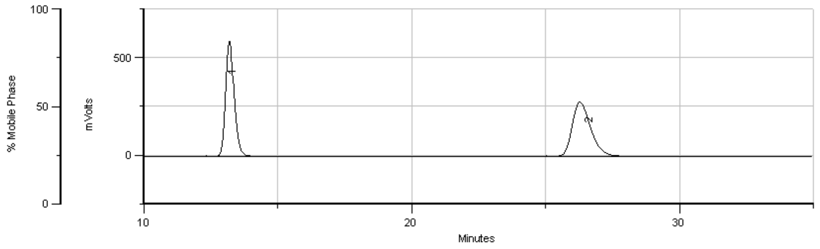

Enantio-enriched

Inj. Number	Peak Name	R. Time	Area	Area %	Sample Descrip.
1.00	*1	13.60	9233911.00	24.53	WN5.119
1.00	*2	27.45	28401788.00	75.47	WN5.119

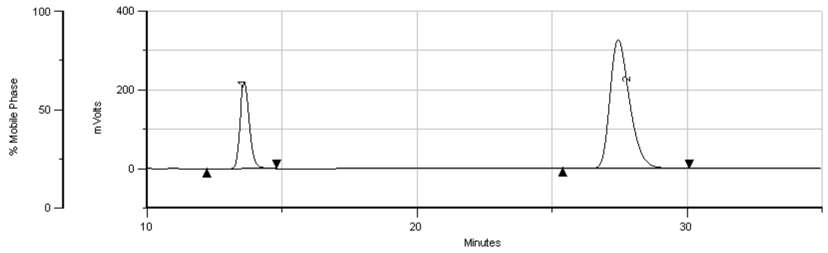

3-hydroxy-3-phenylchroman-4-one (17)

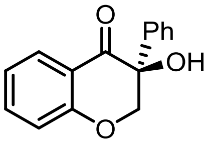

HPLC Chiralpak IC Hexane:IPA 90:10 0.5 mLmin^{-1} 100 min, $t_{r\ maj}$ = 18.0 min $t_{r\ min}$ = 20.7 min

$[\alpha]_D^{22}$ = -35.4° (MeCN, c = 1.02)

Racemic

	Inj. Number	Peak Name	R. Time	Area	Area %	Sample Descrip.
1	1.00	*1	18.03	8453226.00	50.14	ACS2-077
2	1.00	*2	20.74	8404354.00	49.86	ACS2-077
3						

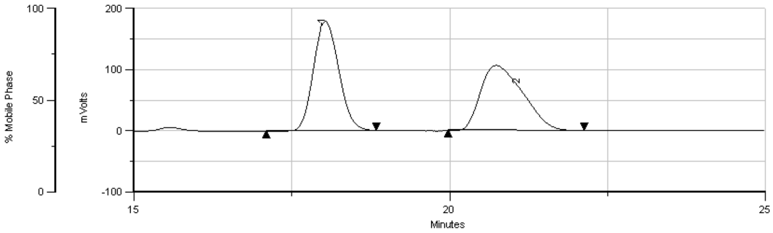

Enantio-enriched

Inj. Number	Peak Name	R. Time	Area	Area %	Sample Descrip.
1.00	*1	17.76	30311776.0(	74.57	WN5.114
1.00	*2	20.73	14442812.00	25.43	WN5.114

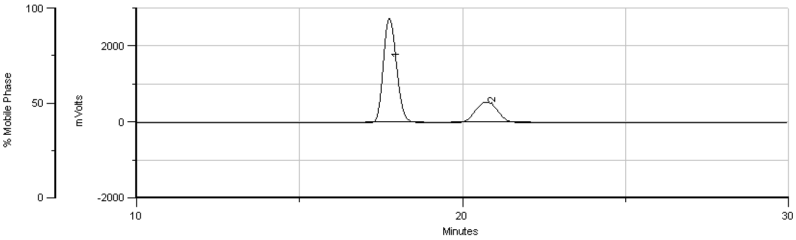

2-hydroxy-1,2-diphenylethan-1-one (14)

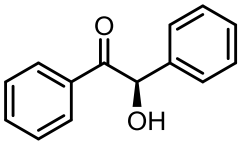

HPLC Chiralpak IC Hexane:IPA 90:10 0.5 mLmin^{-1} 100 min, $t_{r\ maj}$ = 30.4 min $t_{r\ min}$ = 33.2 min

$[\alpha]_D^{22}$ = 64.6˚ (MeCN, c = 0.98) With 100 μL of 2-Propanol

$[\alpha]_D^{22}$ = 23.8˚ (MeCN, c = 0.84)

Racemic

	Inj. Number	Peak Name	R. Time	Area	Area %	Sample Descrip.
1	1.00	*1	30.40	15730539.00	49.90	WN5.95
2	1.00	*2	33.21	15796370.00	50.10	WN5.95
3						

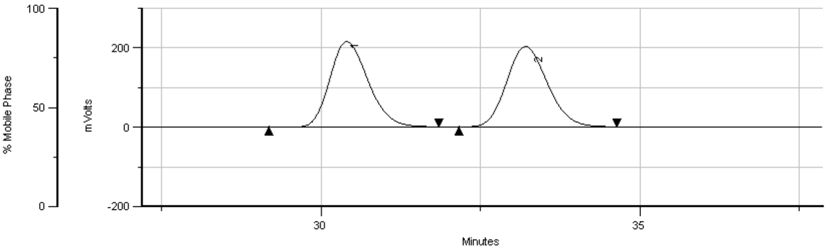

Enantio-enriched No liquid additive

Inj. Number	Peak Name	R. Time	Area	Area %	Sample Descrip.
1.00	*1	30.87	17643864.00	86.29	WN5.117
1.00	*2	33.78	2802642.00	13.71	WN5.117

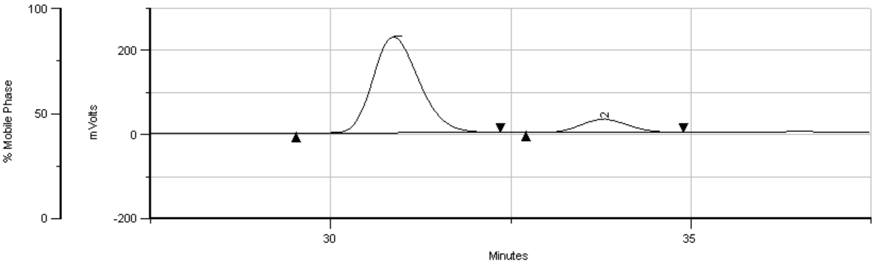

Enantio-enriched with liquid additive

Inj. Number	Peak Name	R. Time	Area	Area %	Sample Descrip.
1.00	*1	30.62	62672240.00	91.01	WN5.118
1.00	*2	33.59	6191123.50	8.99	WN5.118

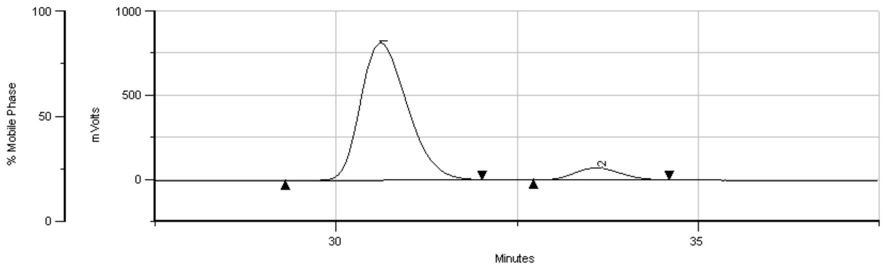

NMR Spectra

5-(2-hydroxyethyl)-3,4-dimethylthiazol-3-ium iodide (2)

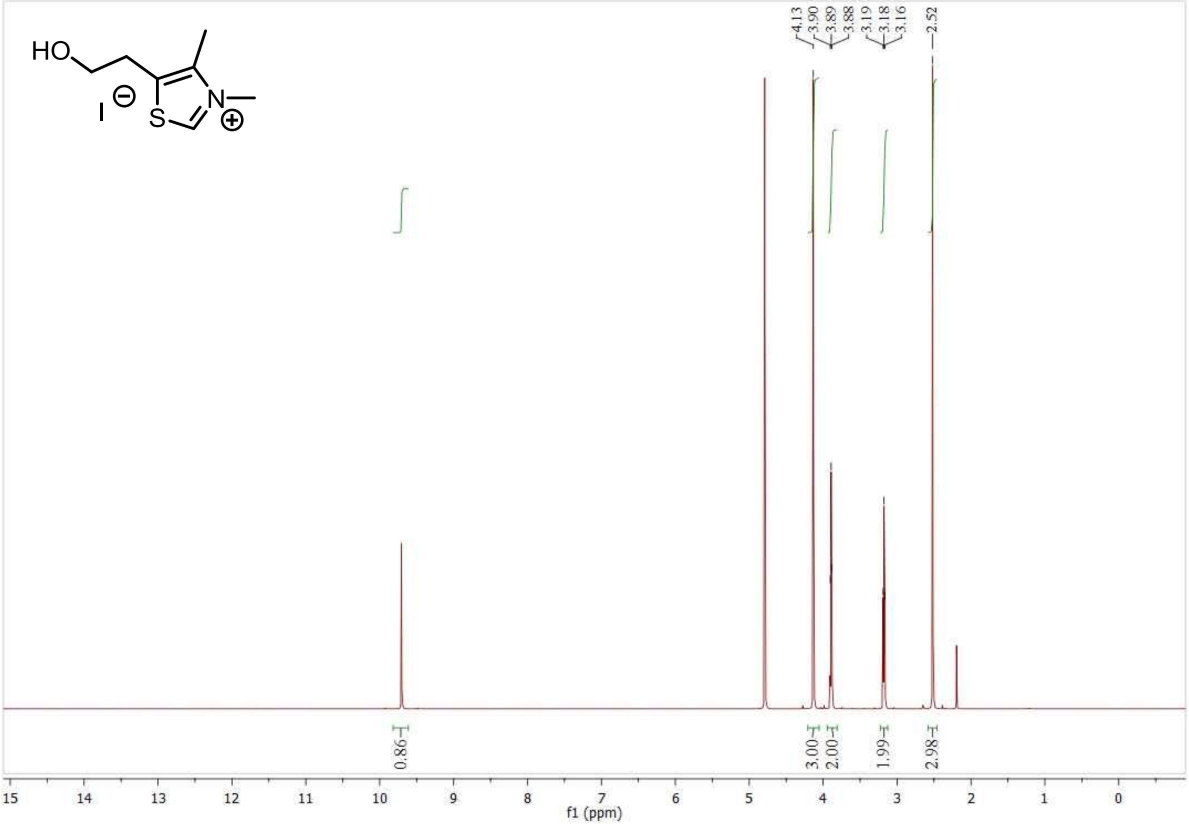

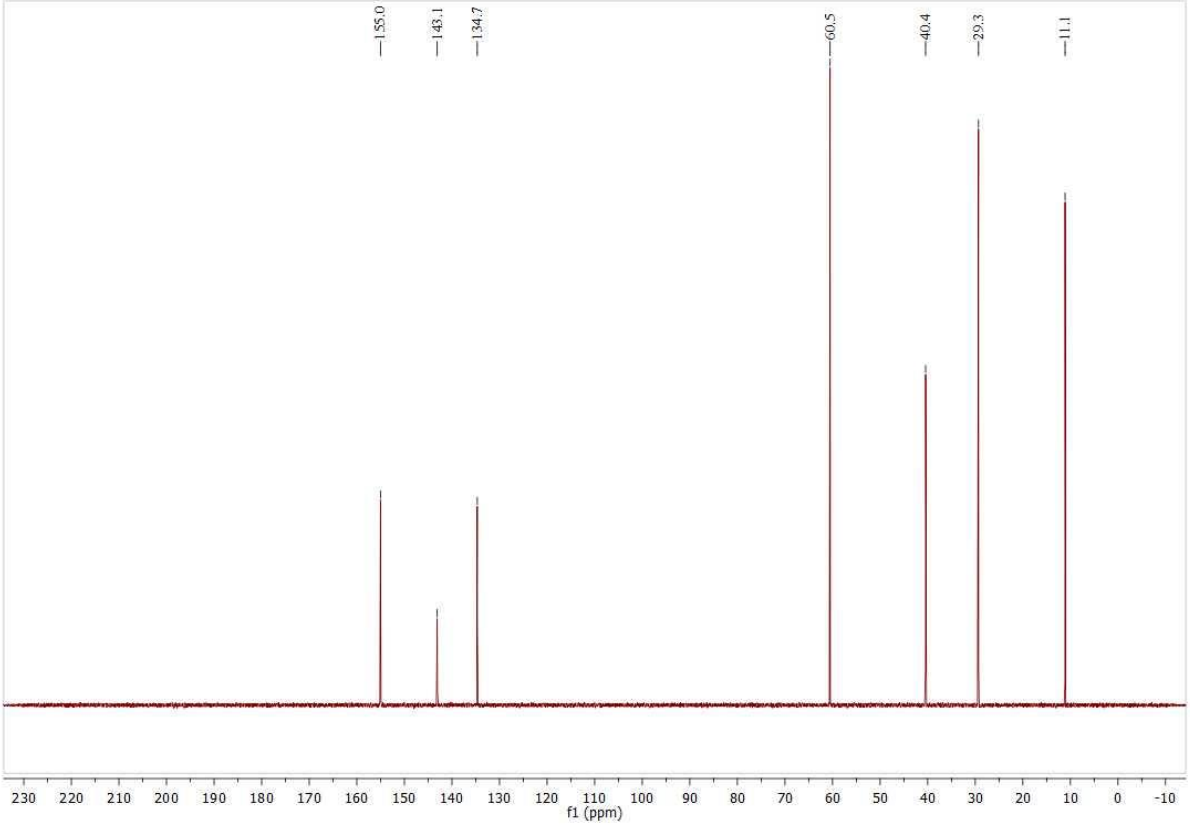

3-mesityl-4-methylthiazol-3-ium perchlorate (36)

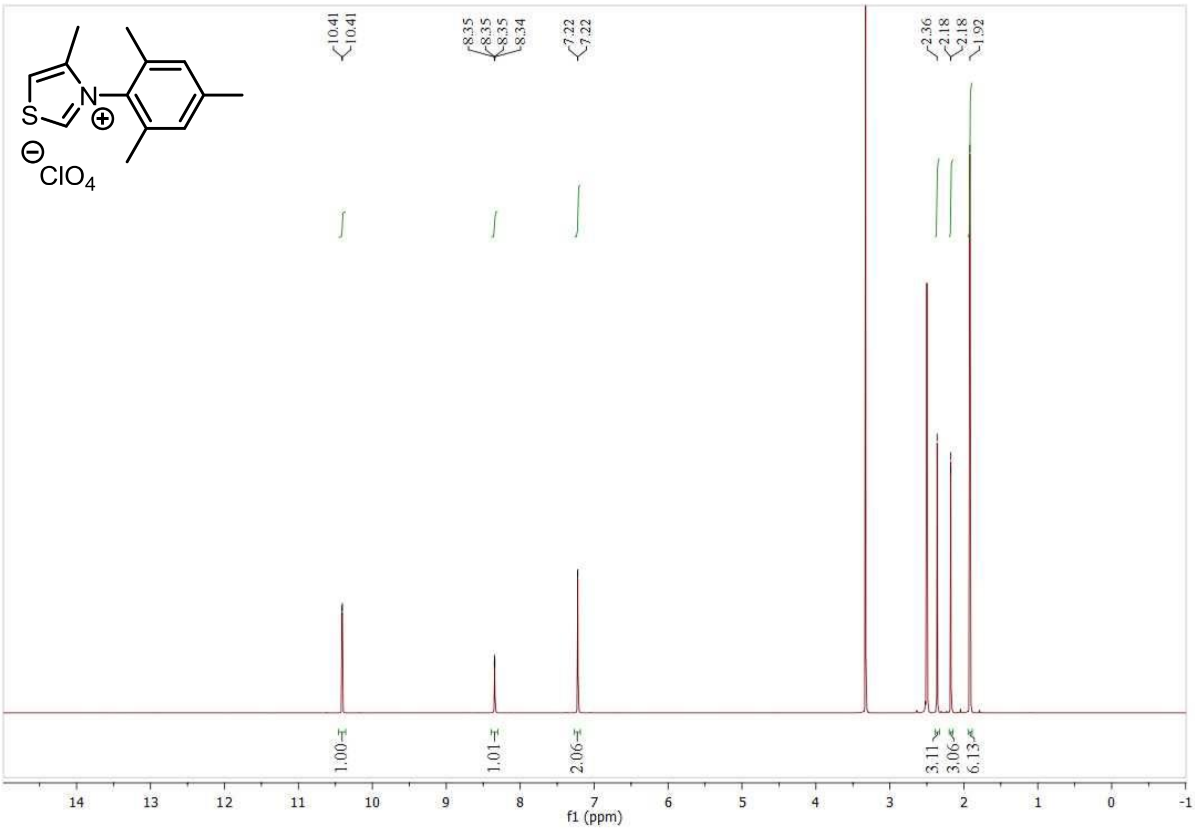

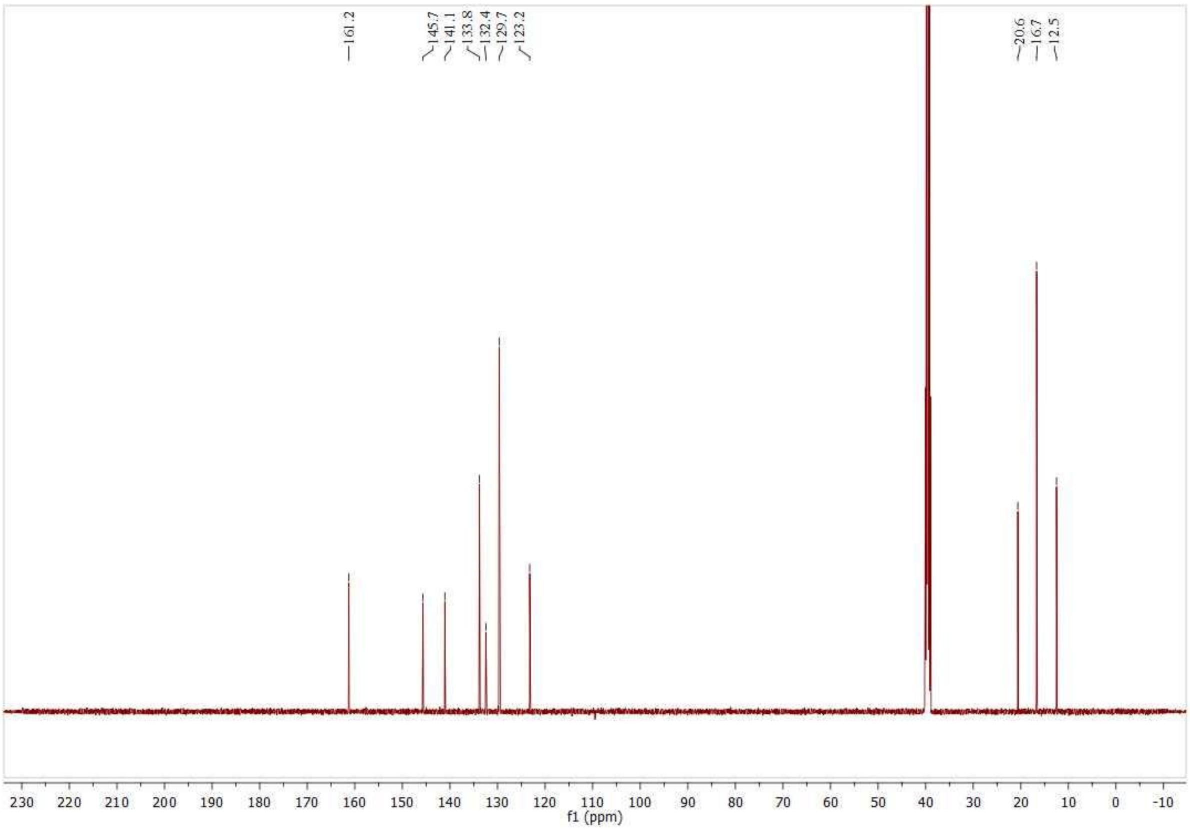

N,N-dimesitylethane-1,2-diimine (4a)

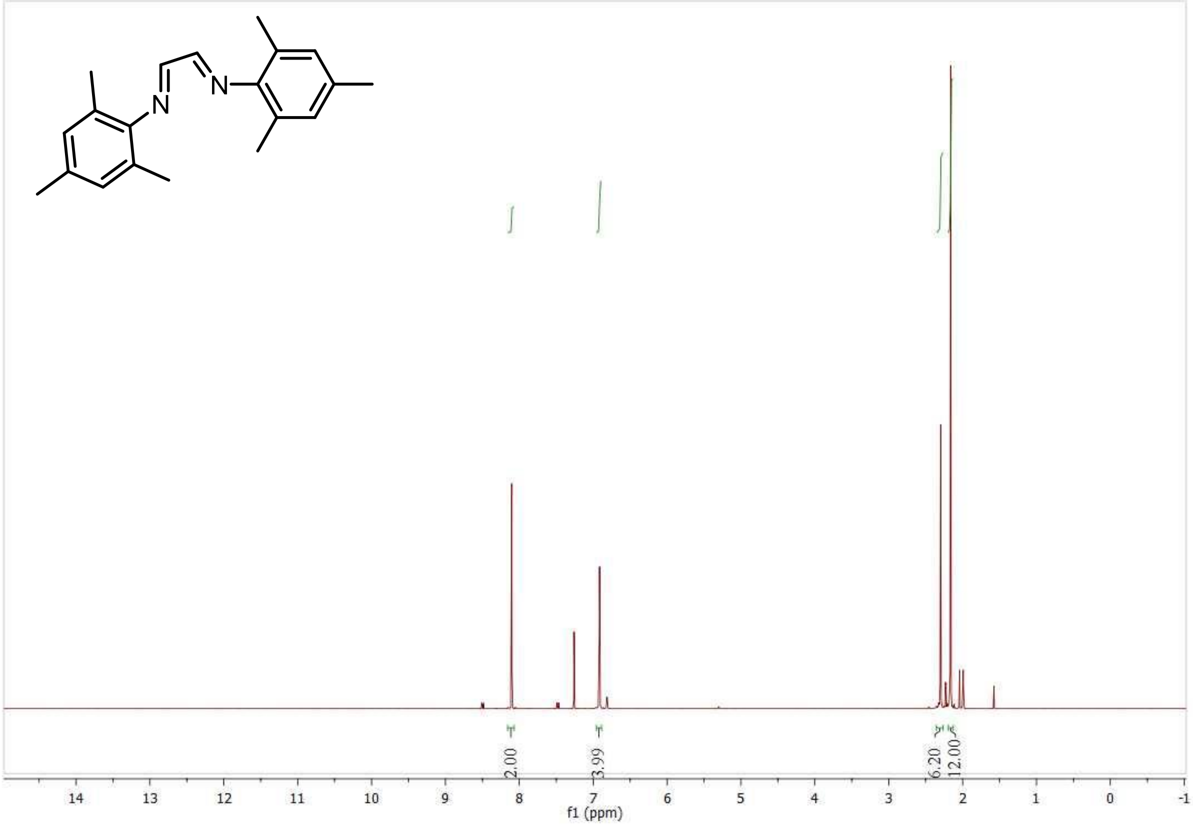

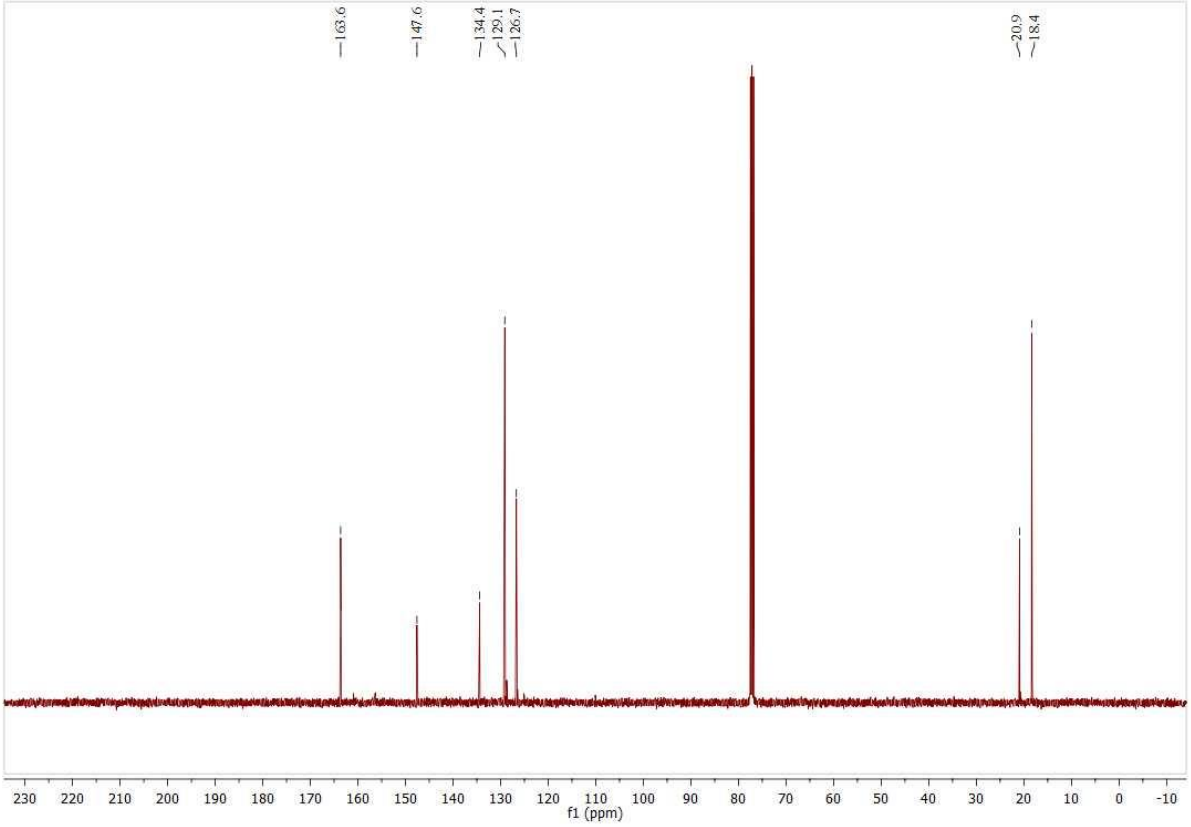

1,3-dimesityl-1H-imidazol-3-ium tetrafluoroborate (4)

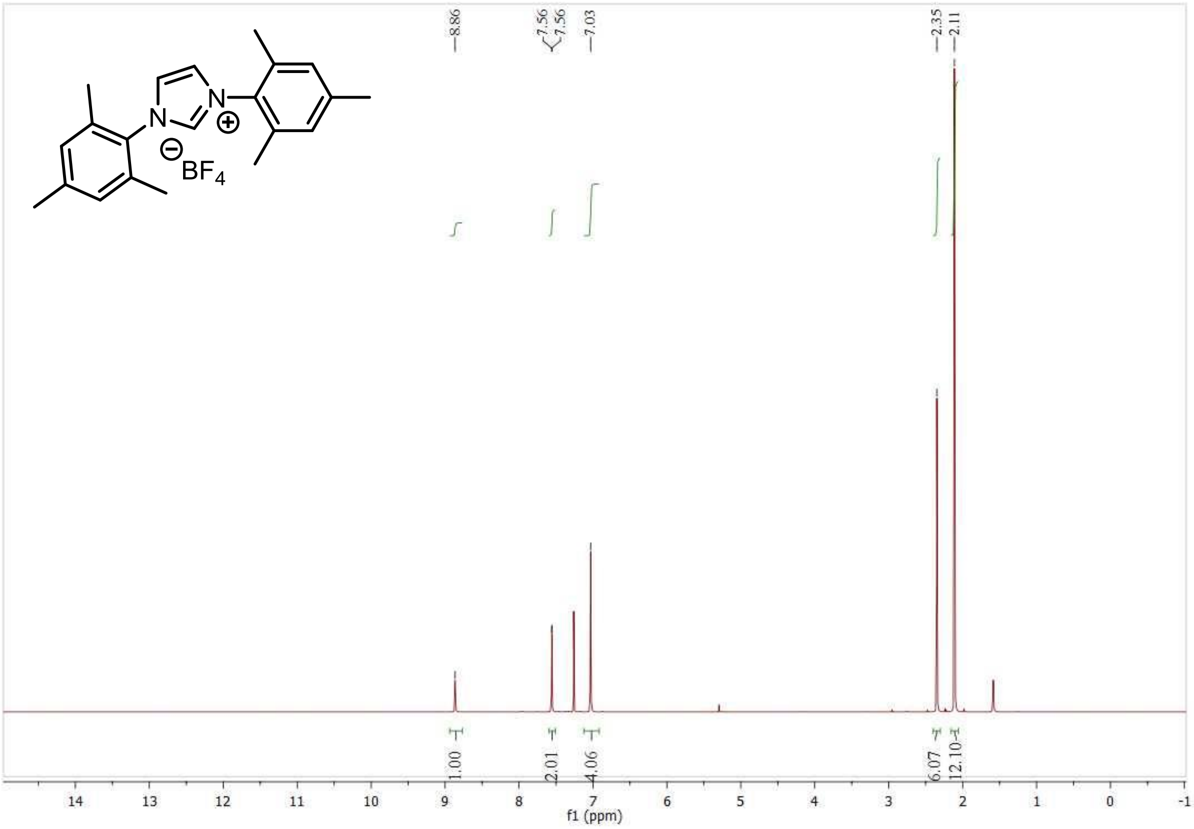

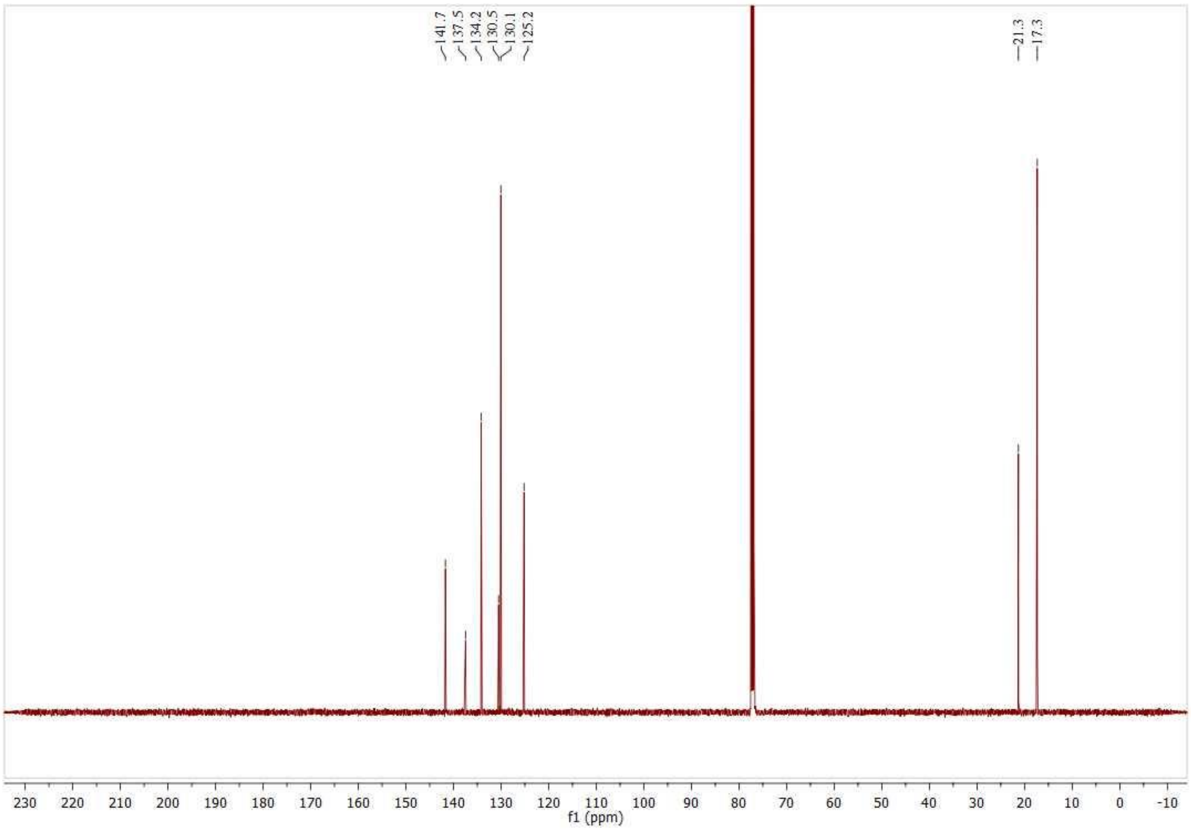

1,3-diisopropyl-1H-imidazol-3-ium tetrafluoroborate (5)

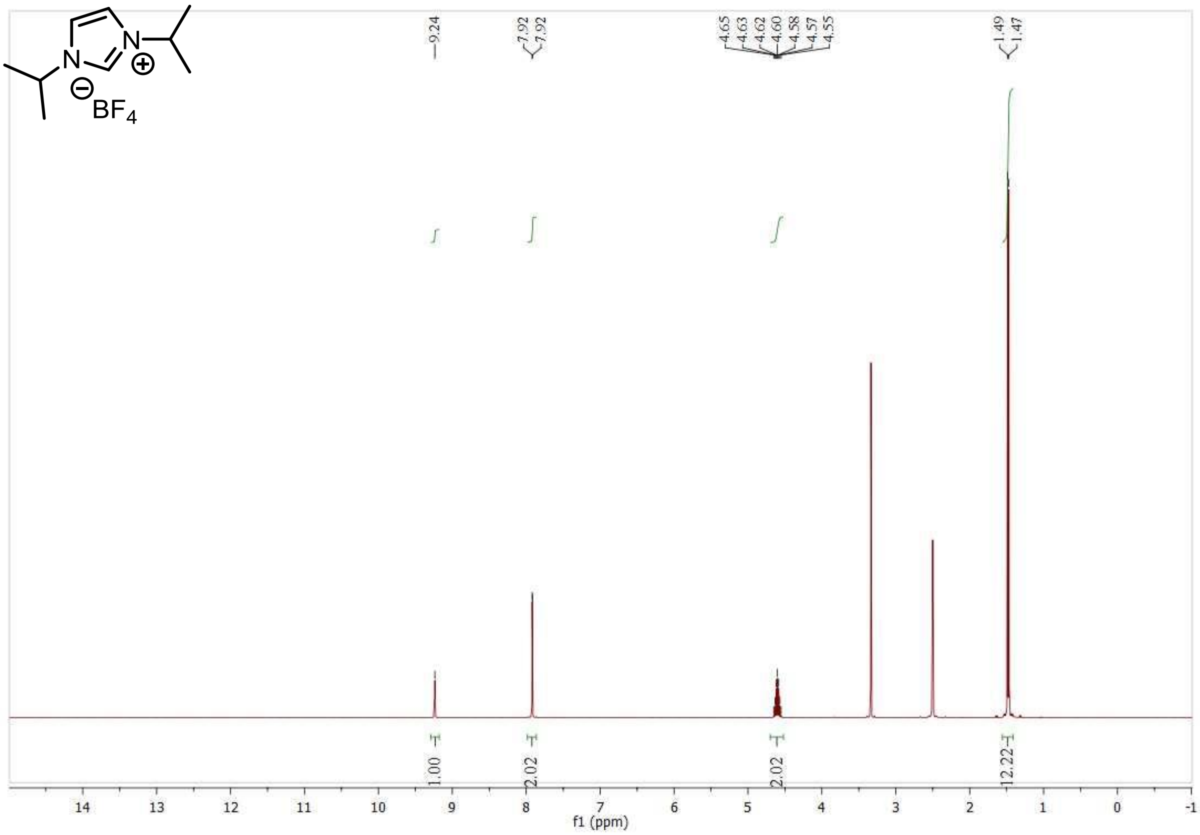

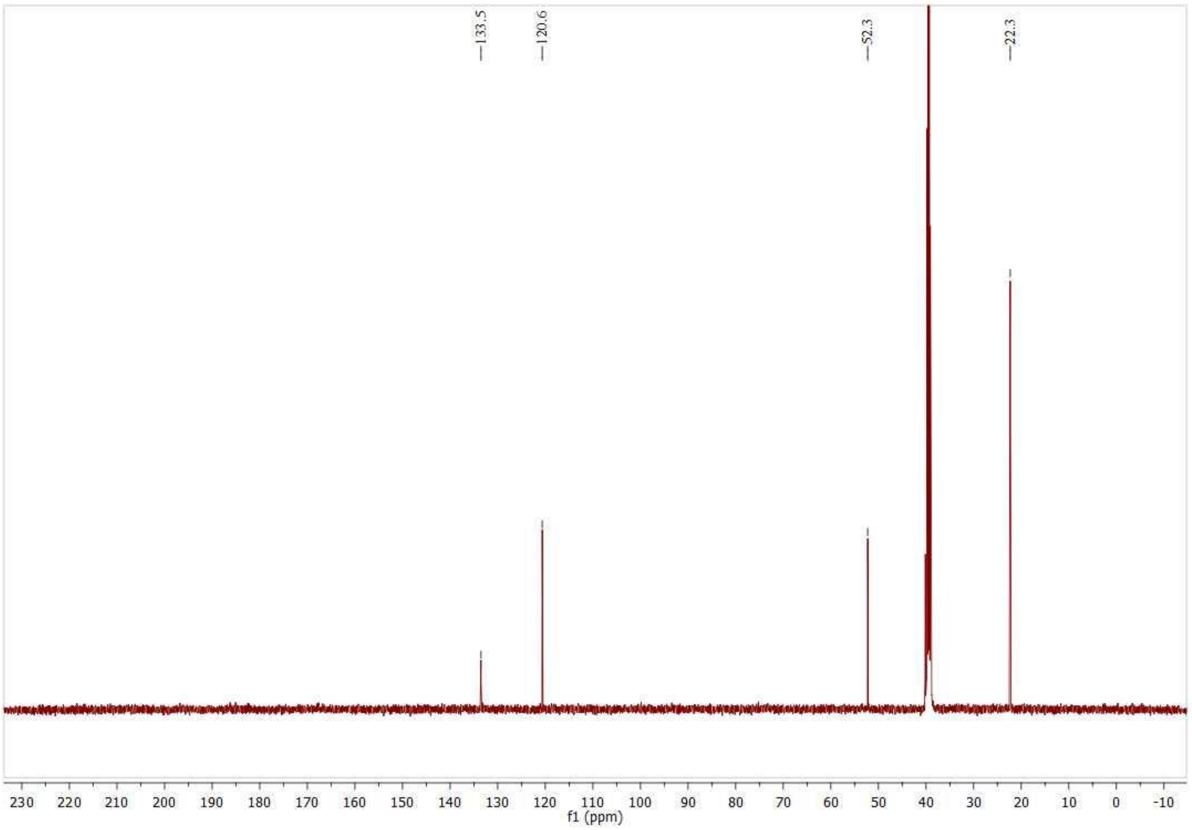

1,4-dibenzyl-4H-1,2,4-triazol-1-ium bromide (7)

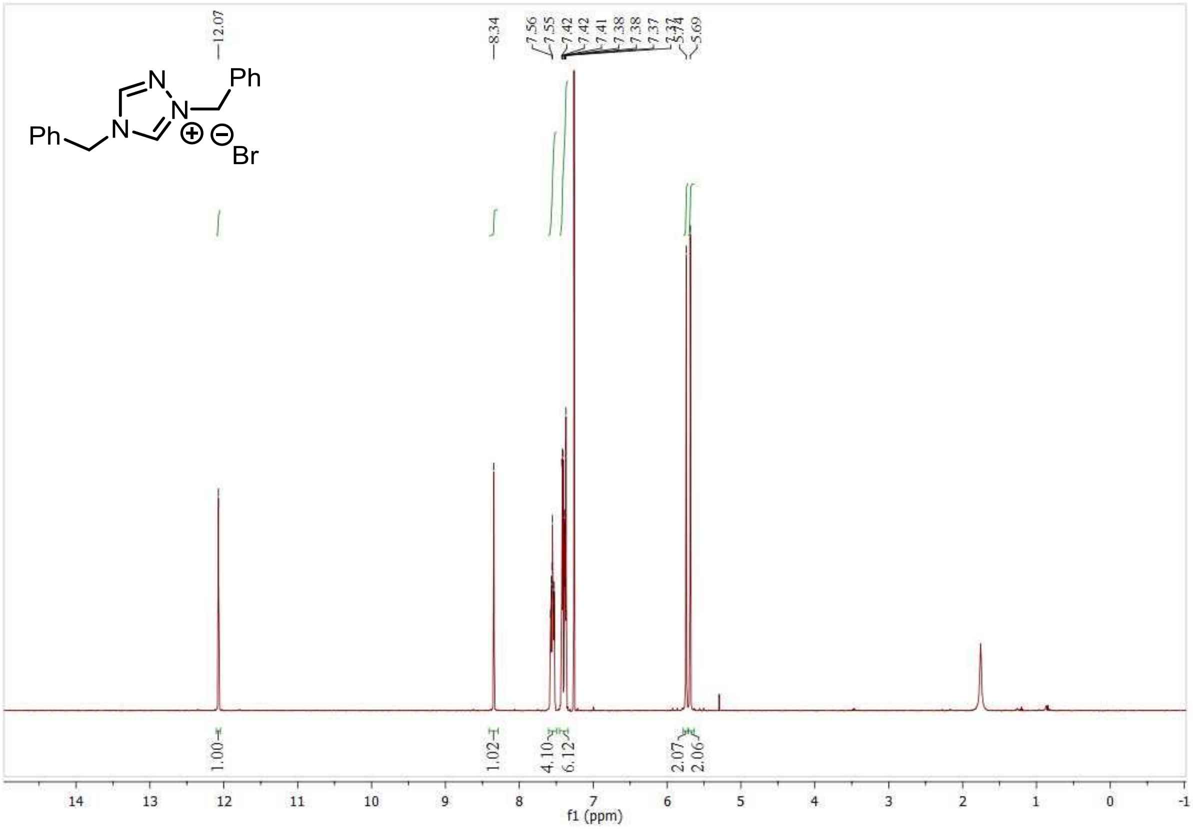

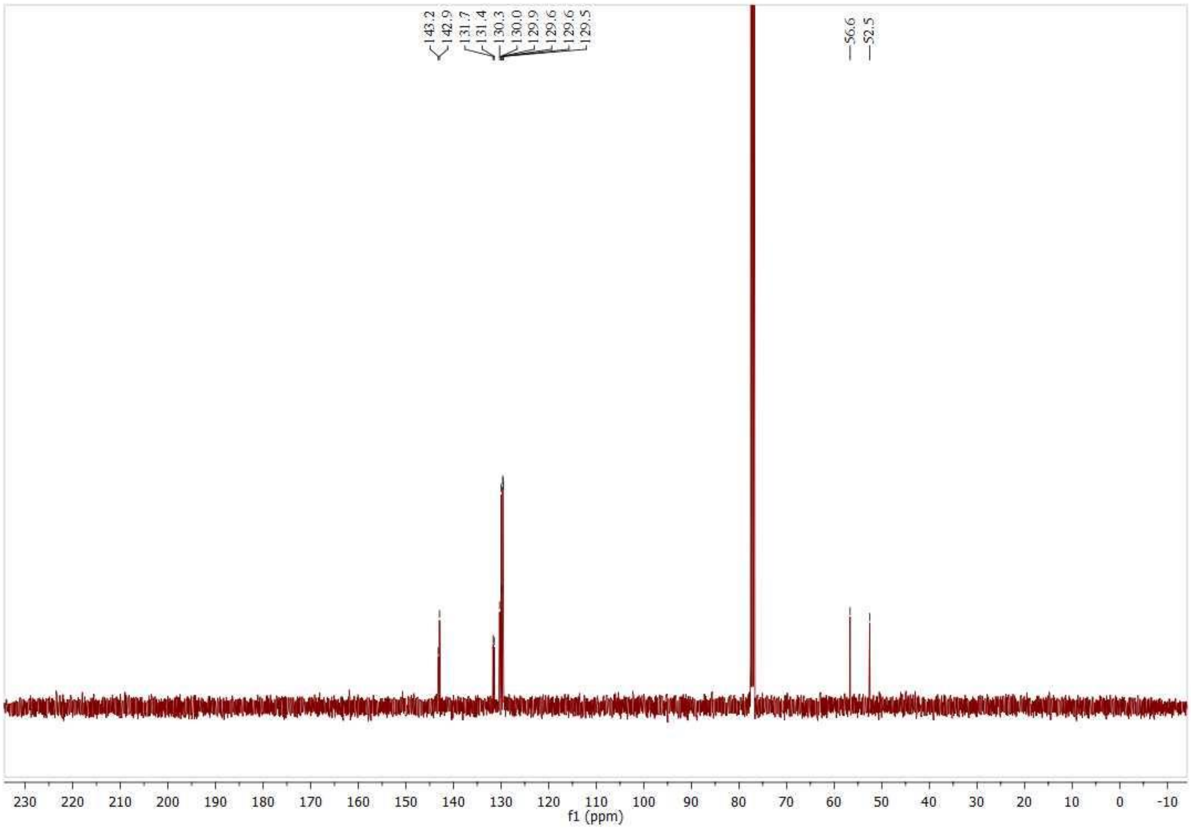

1,3-dibenzyl-1H-imidazol-3-ium tetrafluoroborate (6)

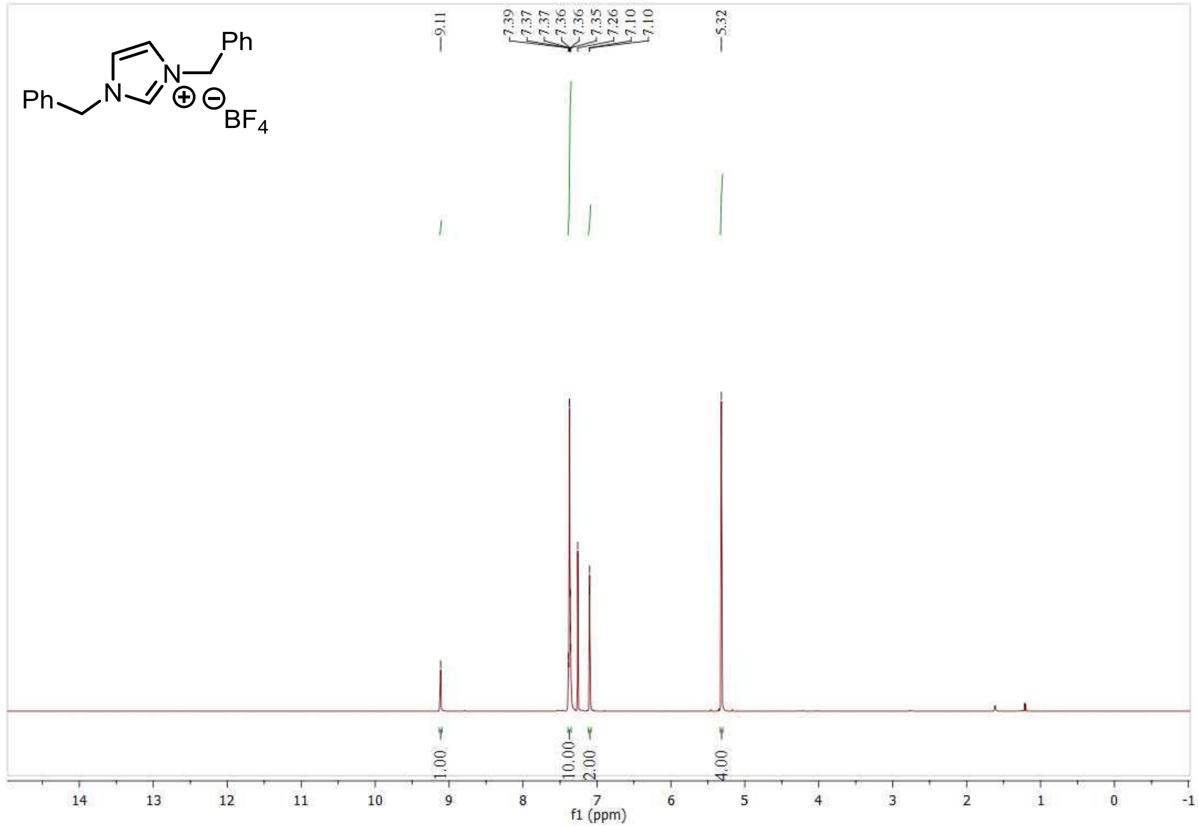

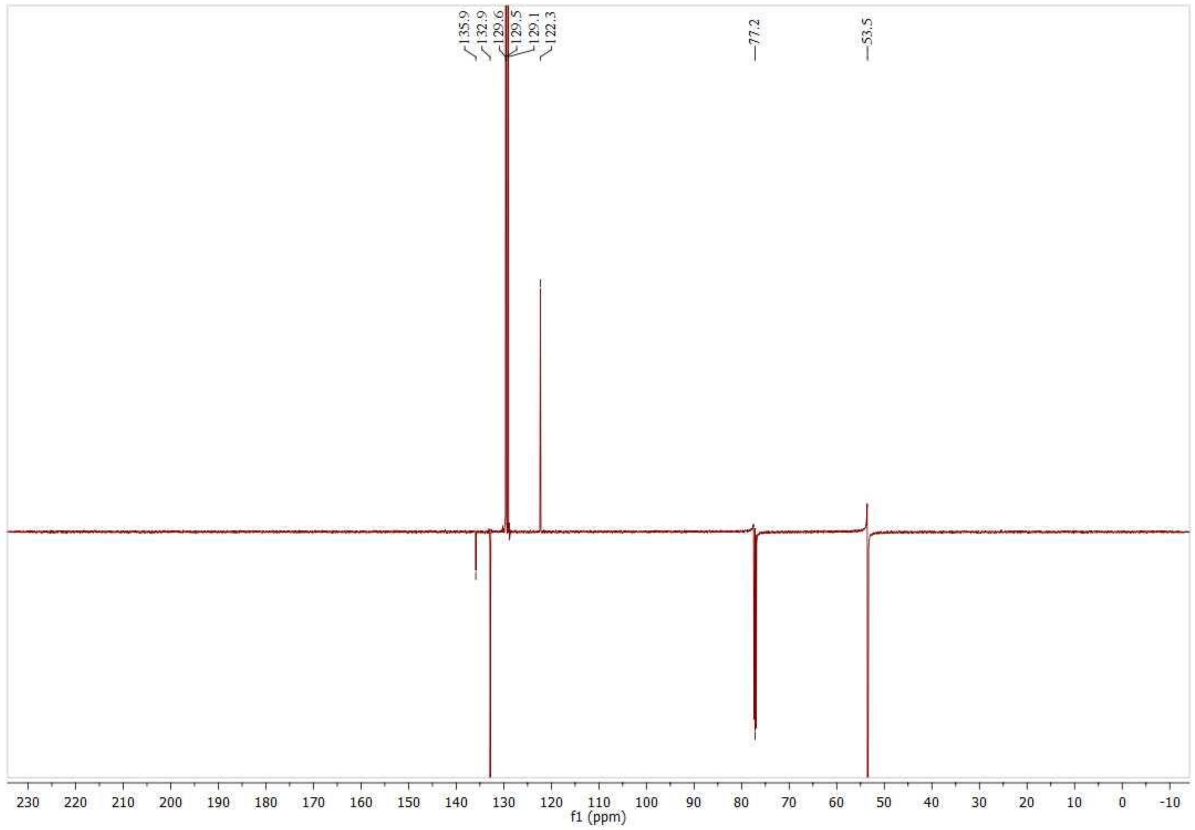

2-phenyl-6,7-dihydro-5H-pyrrolo[2,1-c][1,2,4]triazol-2-ium tetrafluoroborate (9)

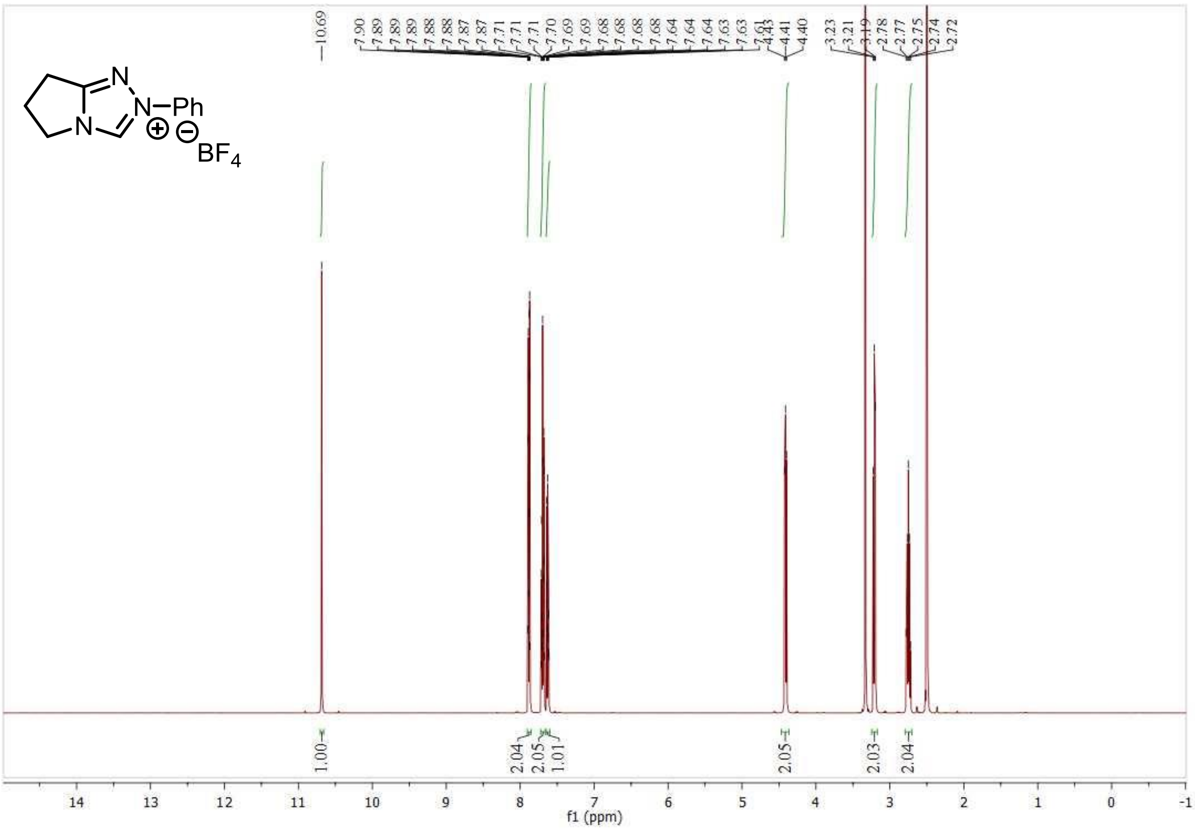

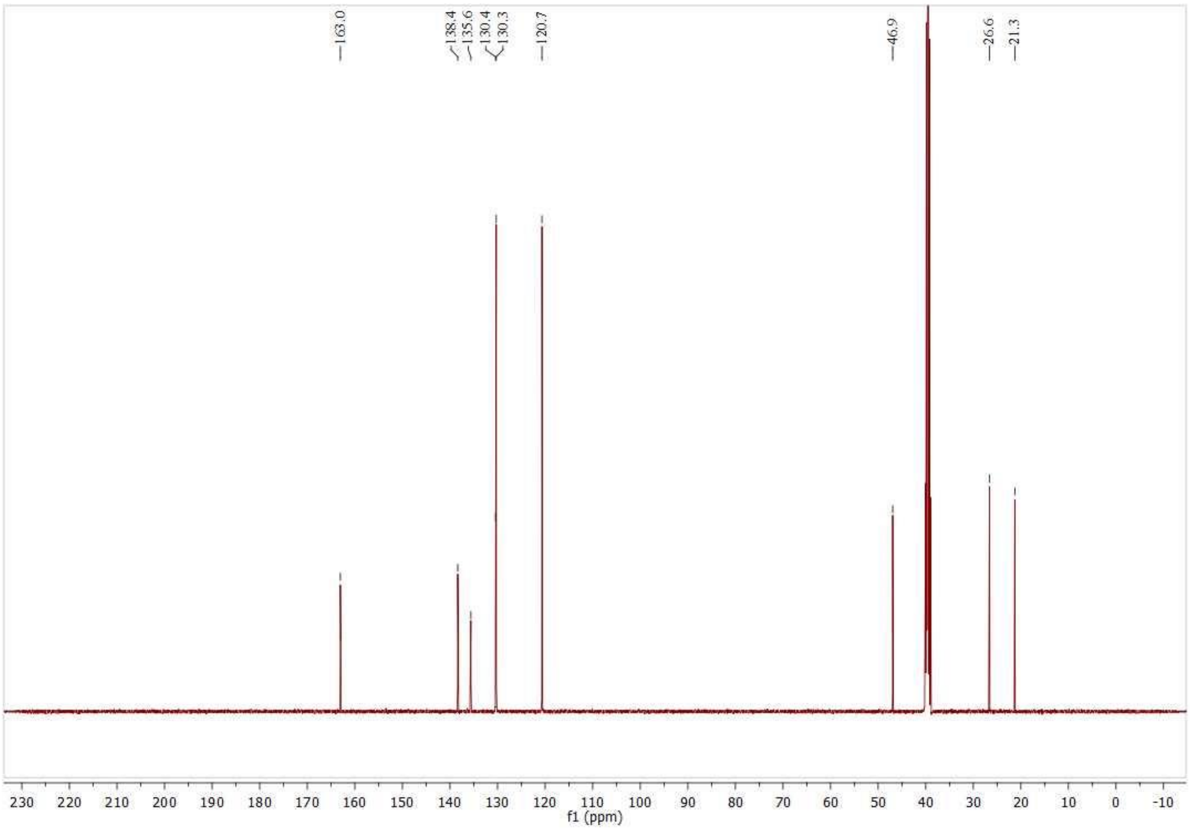

2-(perfluorophenyl)-6,7-dihydro-5H-pyrrolo[2,1-c][1,2,4]triazol-2-ium tetrafluoroborate (8)

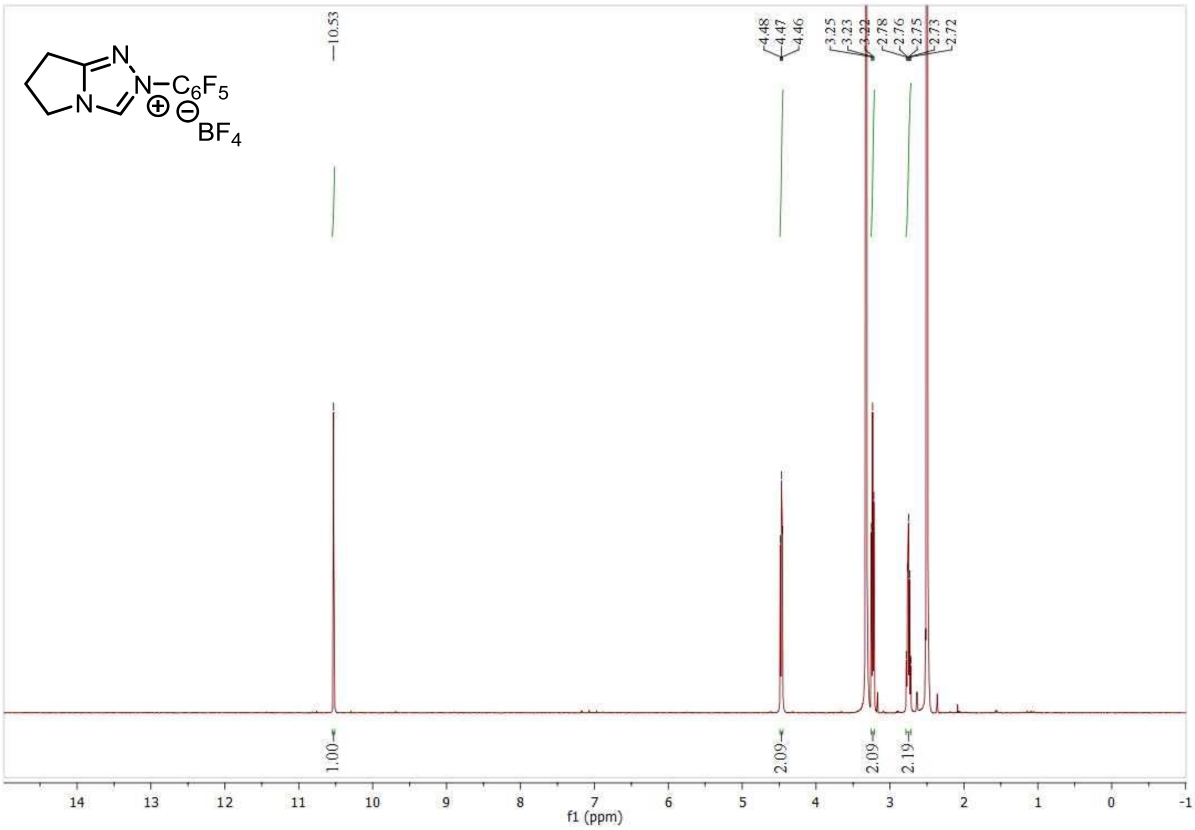

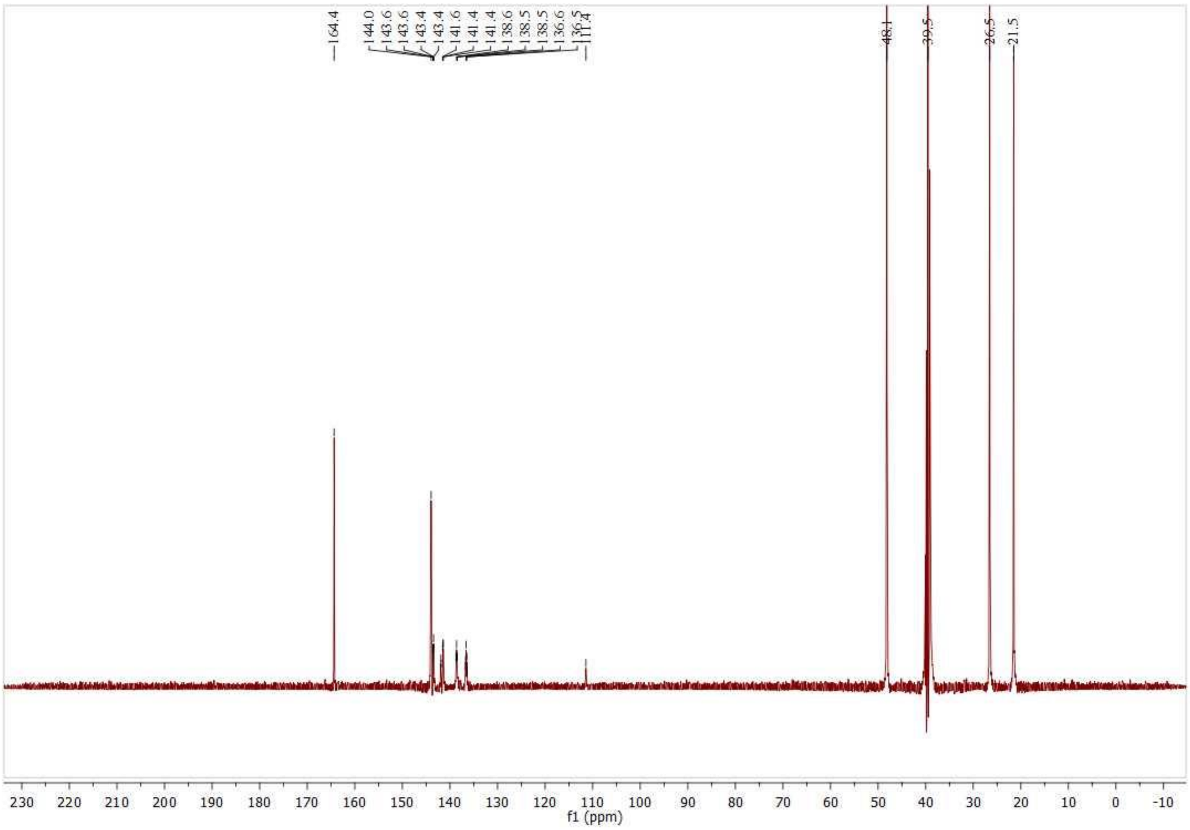

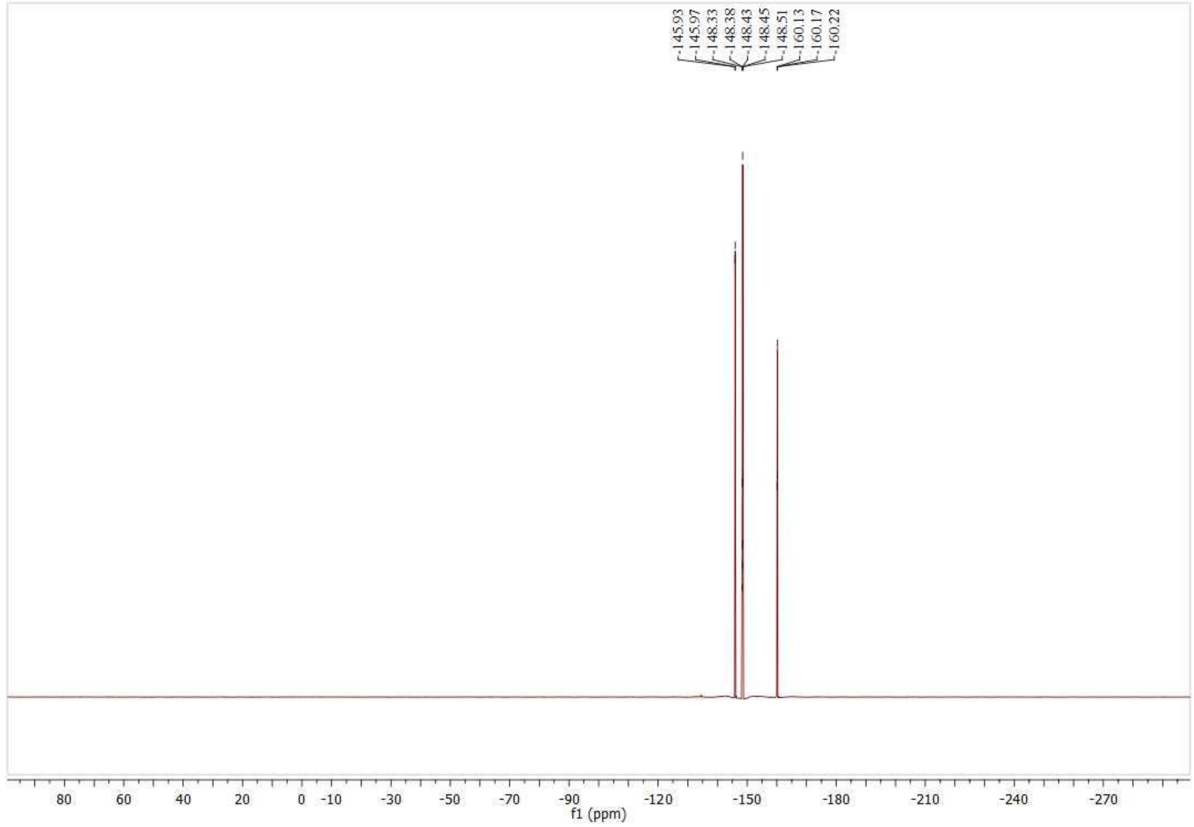
-145.93
-145.97
-148.33
-148.38
-148.43
-148.45
-148.51
-160.13
-160.17
-160.22
80 60 40 20 0 -10 -30 -50 -70 -90 -120 -150 -180 -210 -240 -270
f1 (ppm)

2-(Perfluorophenyl)-5,6,7,8-tetrahydro-[1,2,4]triazolo[4,3-a]pyridin-2-ium tetrafluoroborate (10)

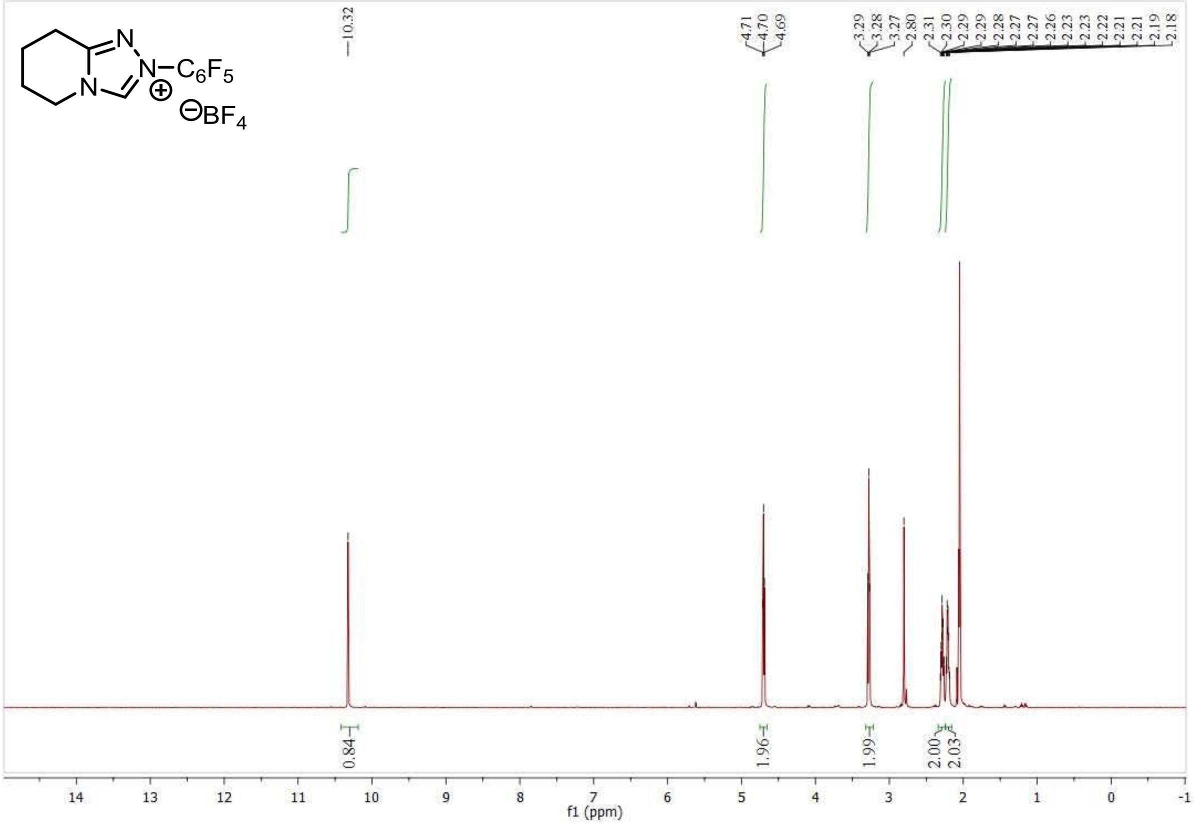

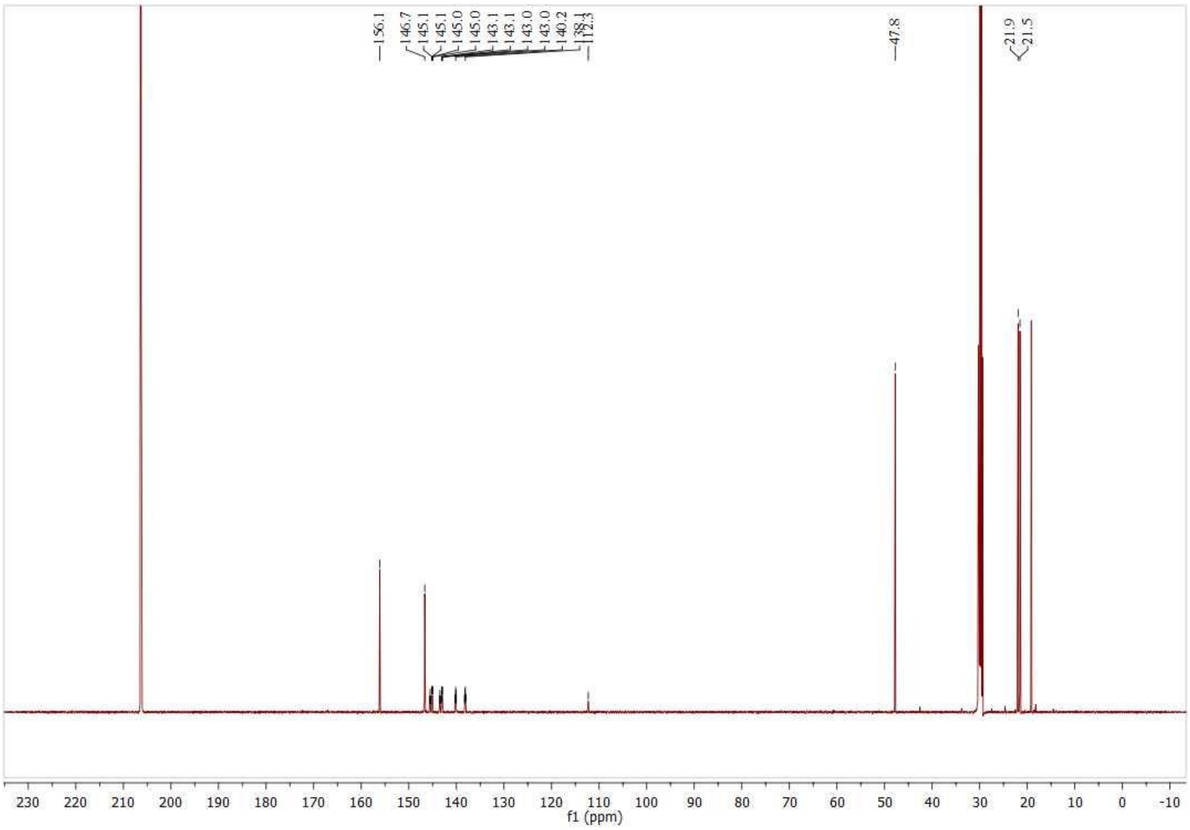

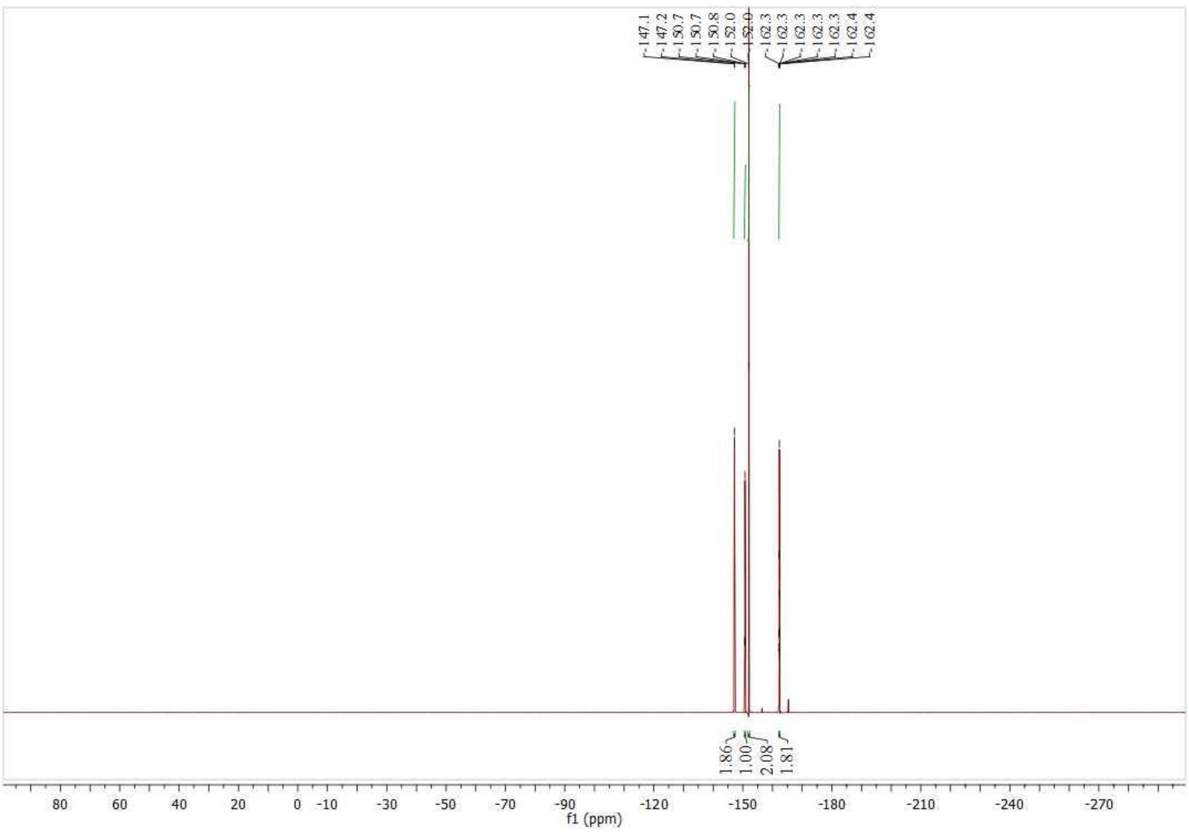
-147.1
-147.2
-150.7
-150.7
-150.8
-152.0
-152.0
-162.3
-162.3
-162.3
-162.3
-162.3
-162.4
-162.4
1.86
1.00
2.08
1.81
80 60 40 20 0 -10 -30 -50 -70 -90 -120 -150 -180 -210 -240 -270
f1 (ppm)

2-benzyl-6,7-dihydro-5H-pyrrolo[2,1-c][1,2,4]triazol-2-ium tetrafluoroborate (37)

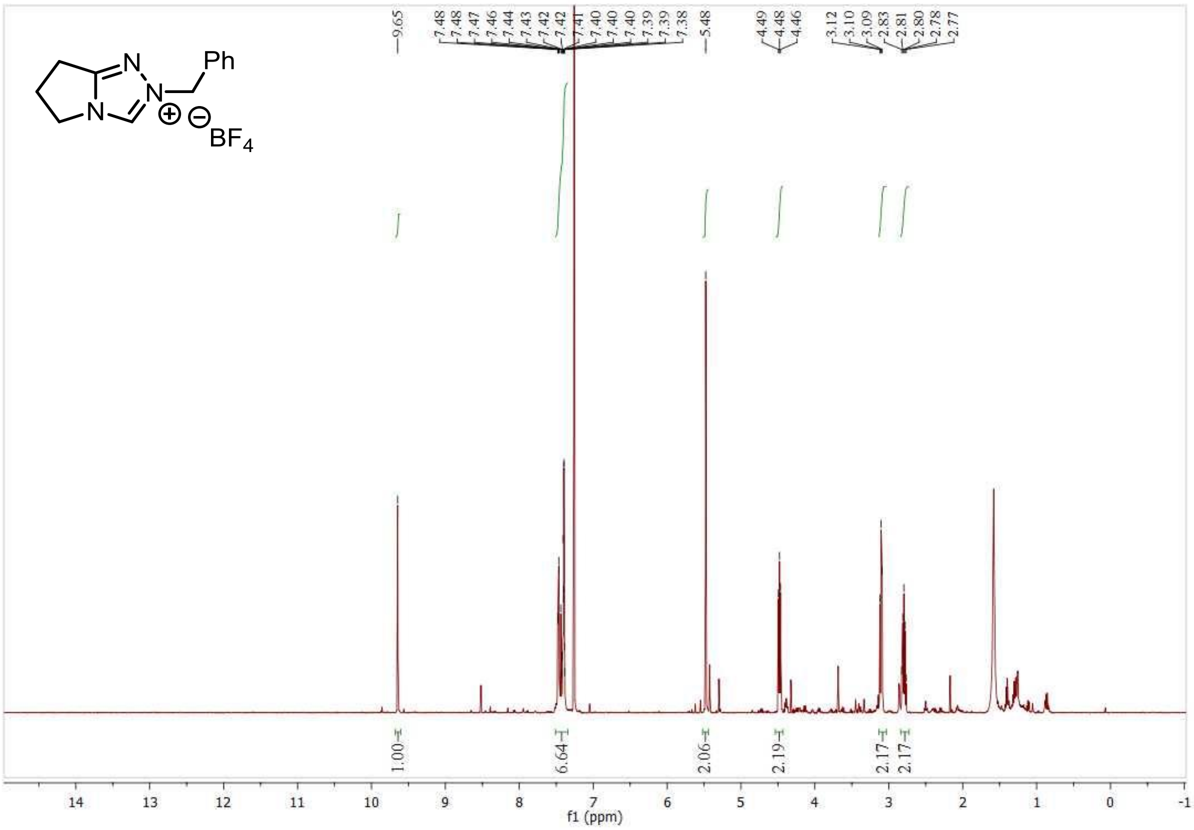

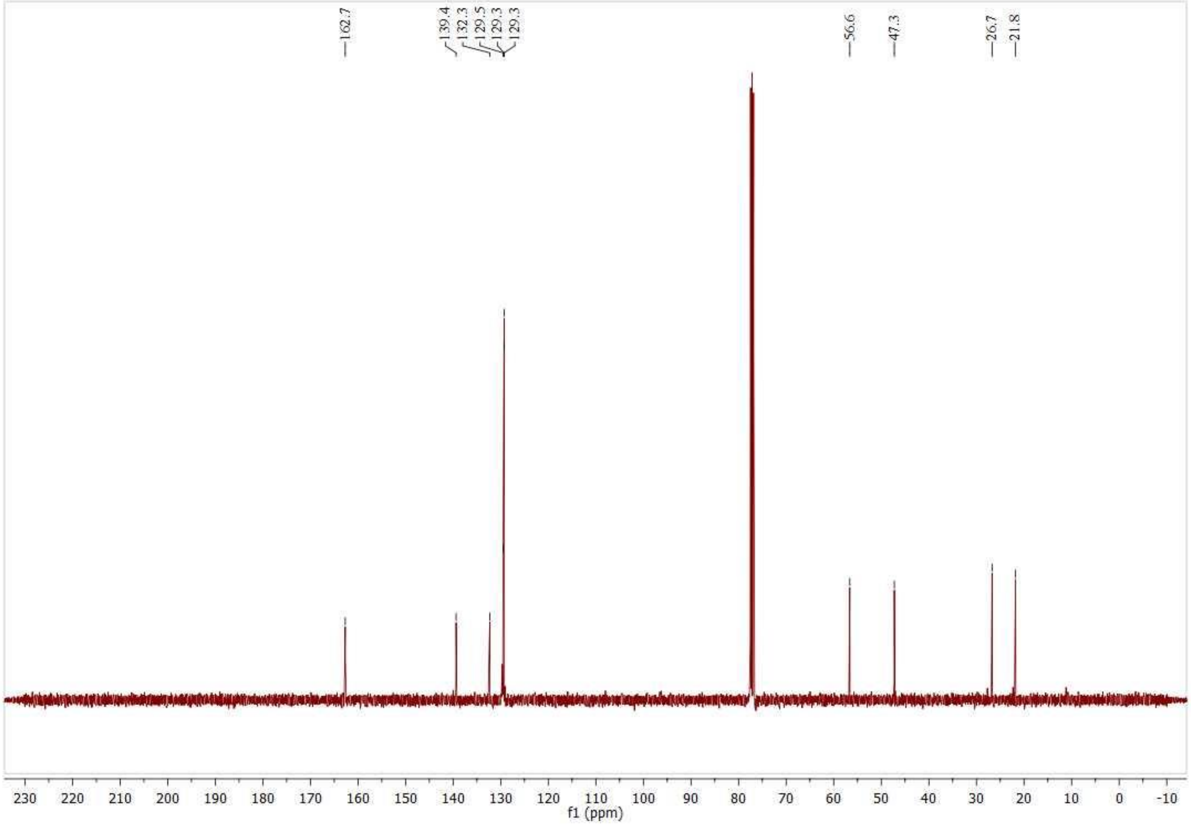

1,2-bis(4-chlorophenyl)-2-hydroxyethan-1-one (11)

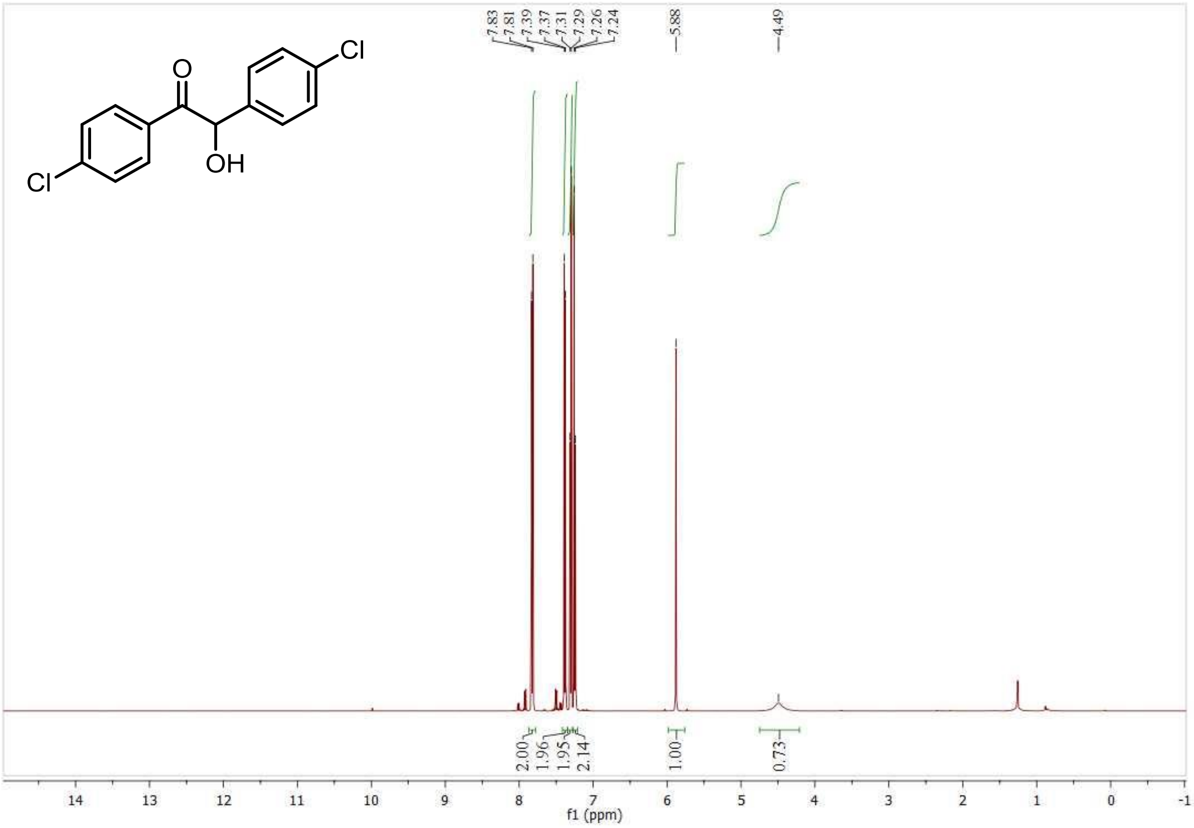

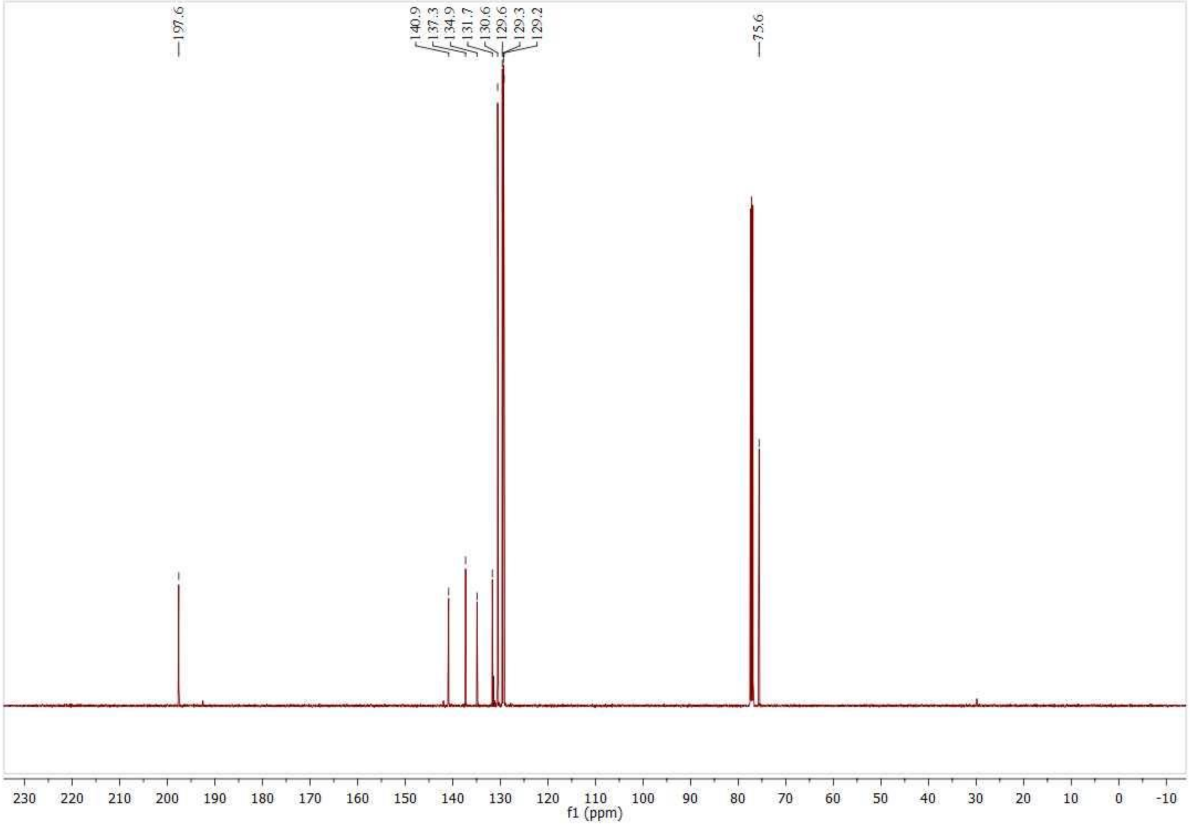

2-hydroxy-1,2-diphenylethan-1-one (14)

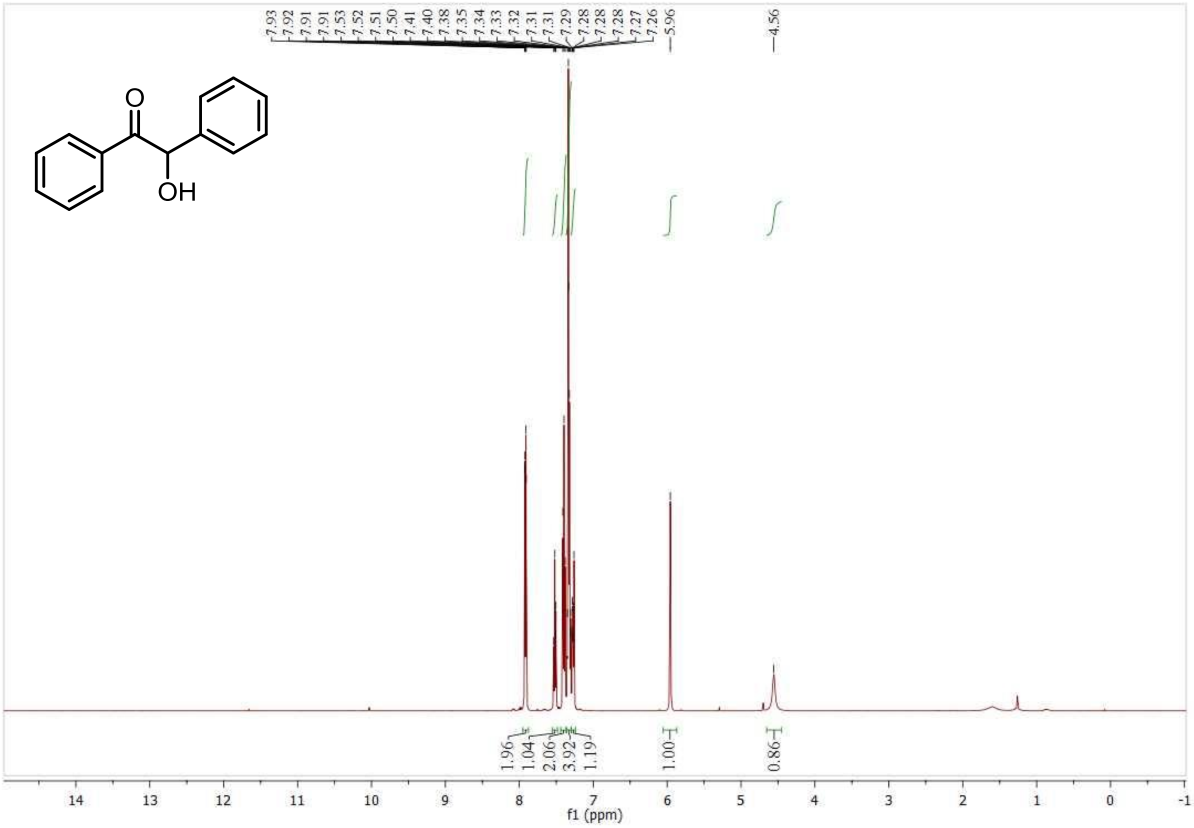

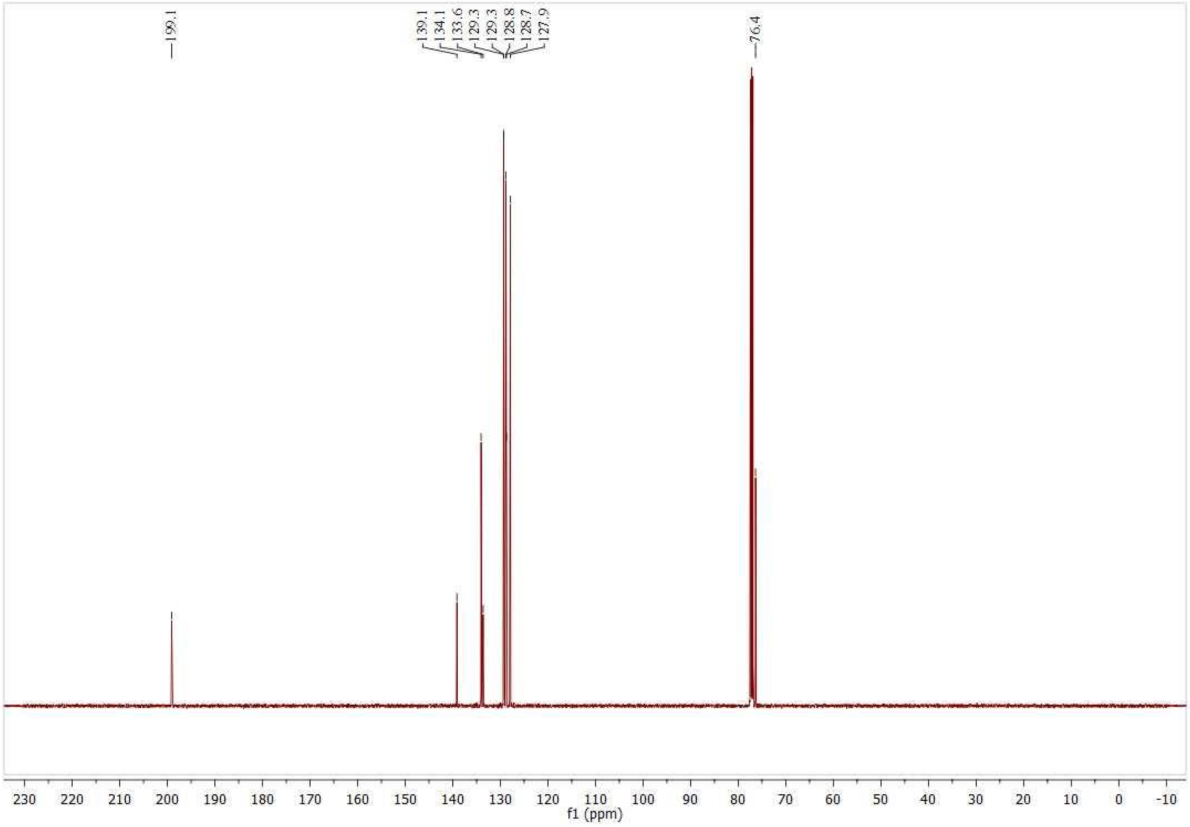

2-hydroxy-1,2-di-p-tolylethan-1-one (12)

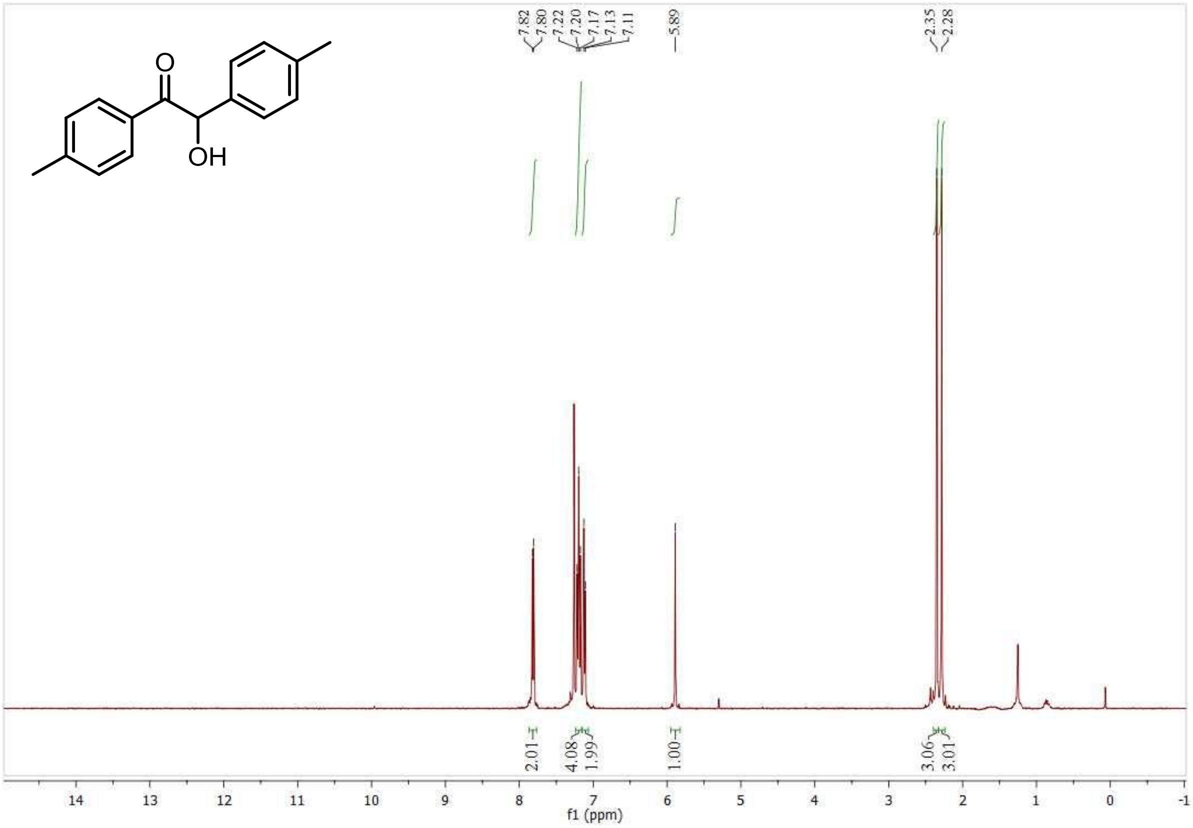

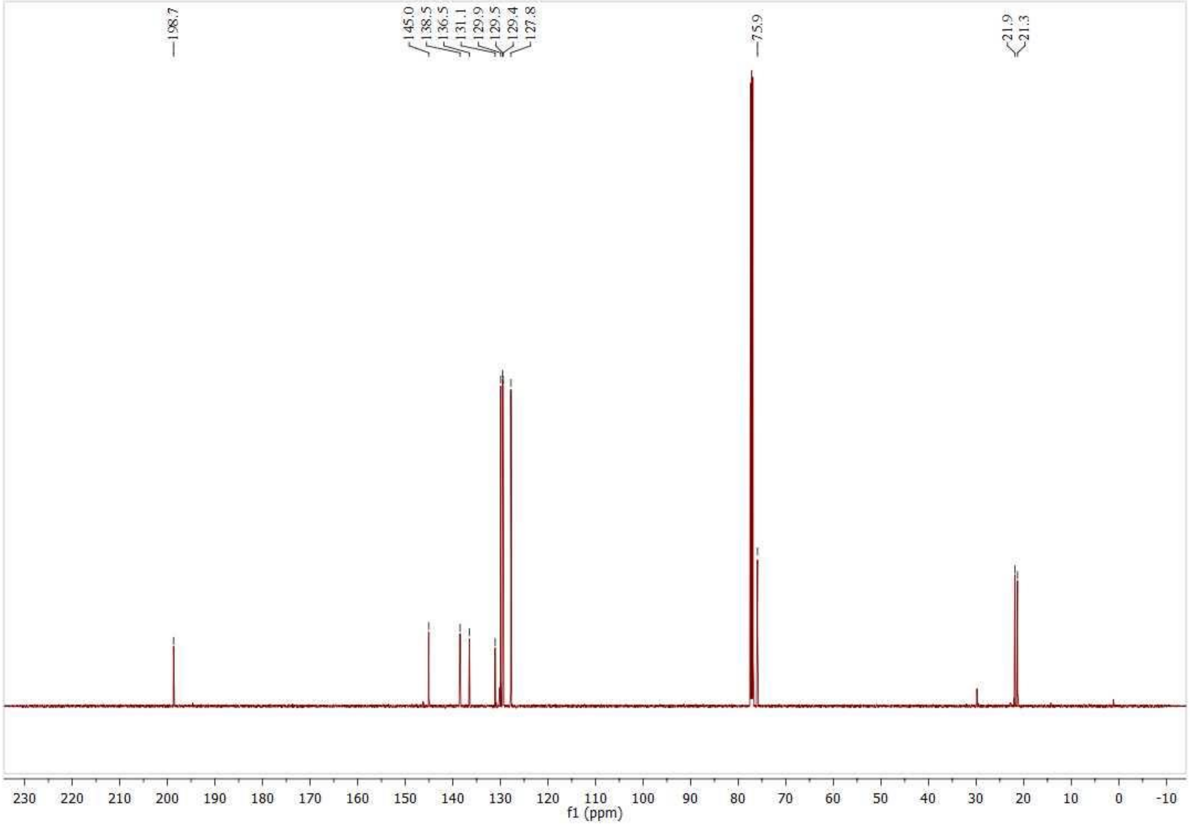

1,2-bis(4-fluorophenyl)-2-hydroxyethan-1-one (15)

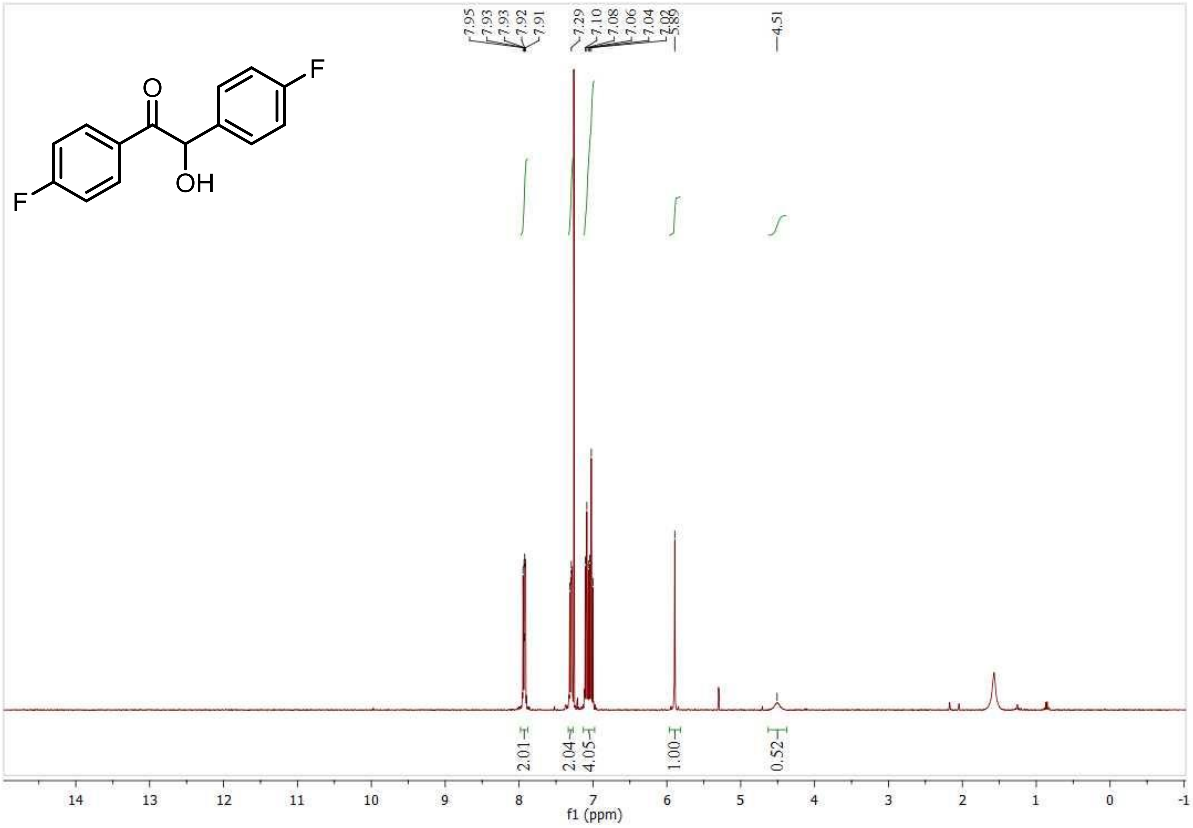

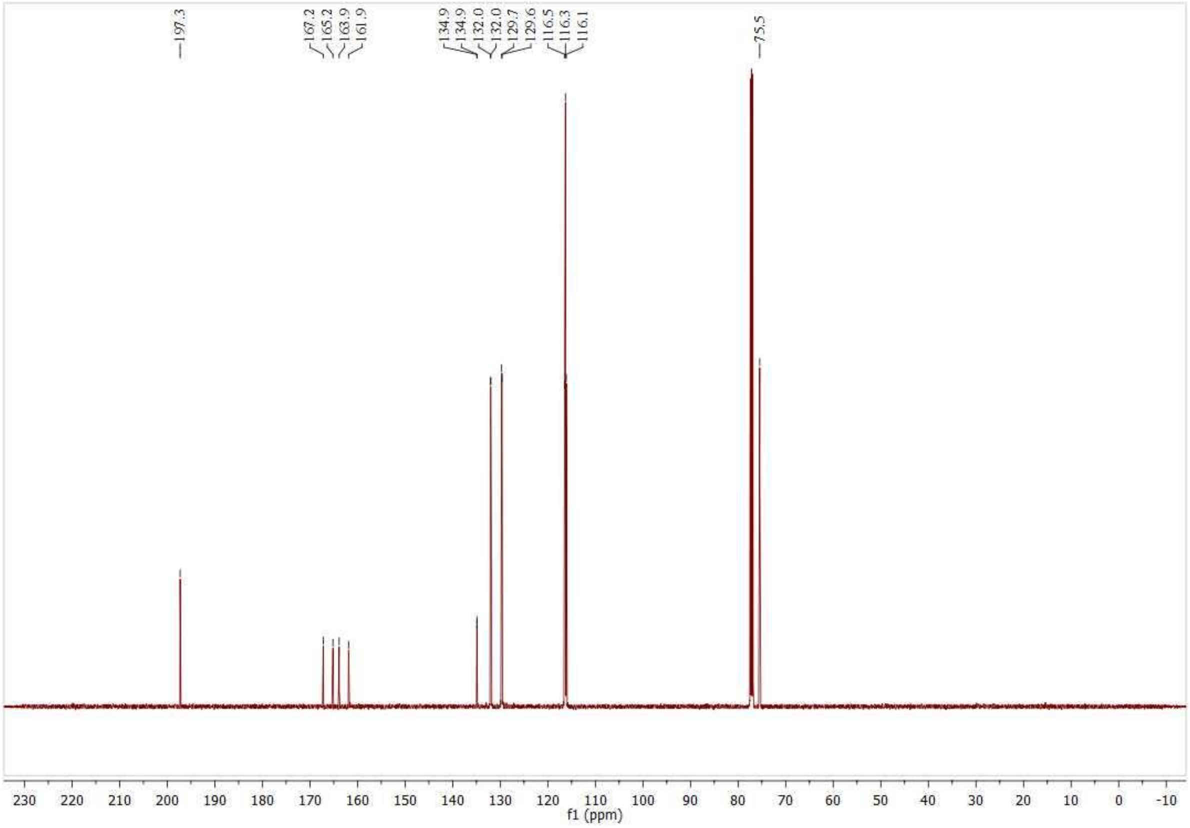

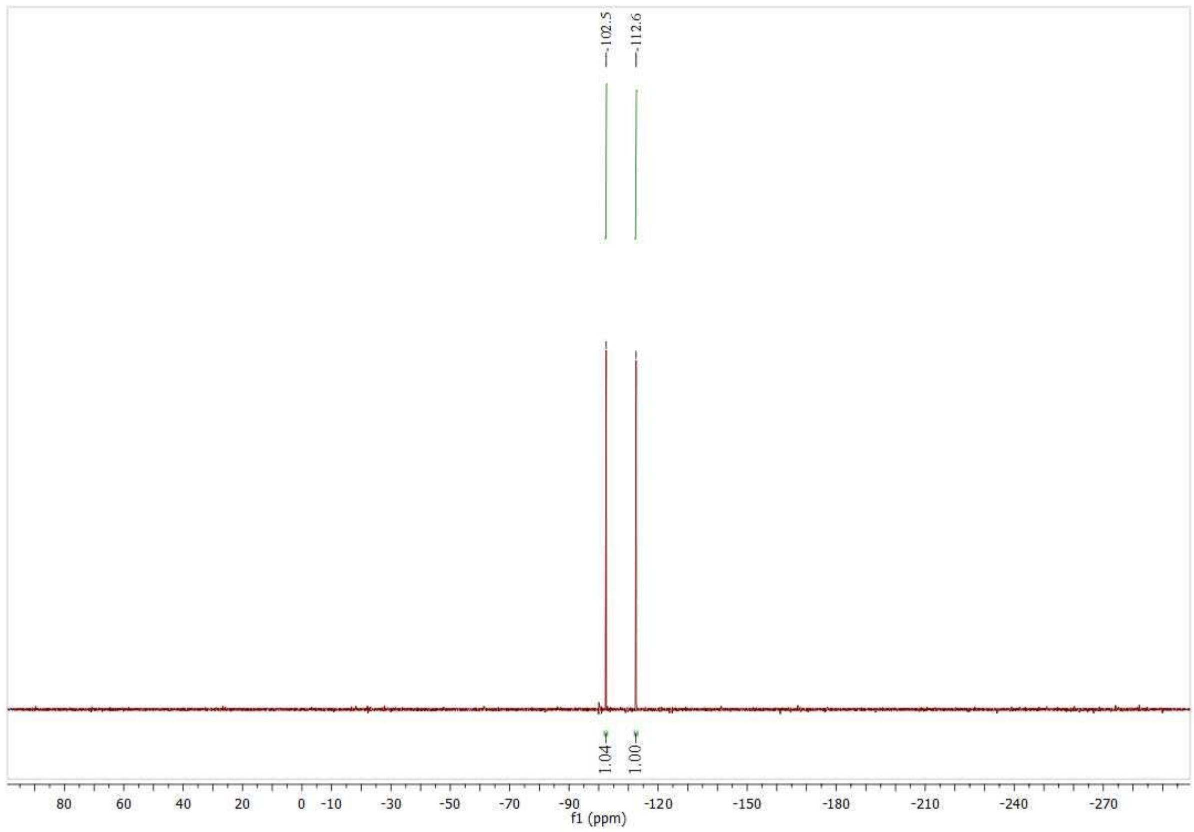

-102.5
-112.6
1.04
1.00
80
60
40
20
0
-10
-30
-50
-70
-90
-120
-150
-180
-210
-240
-270
f1 (ppm)

2-hydroxy-1,2-bis(4-methoxyphenyl)ethan-1-one (13)

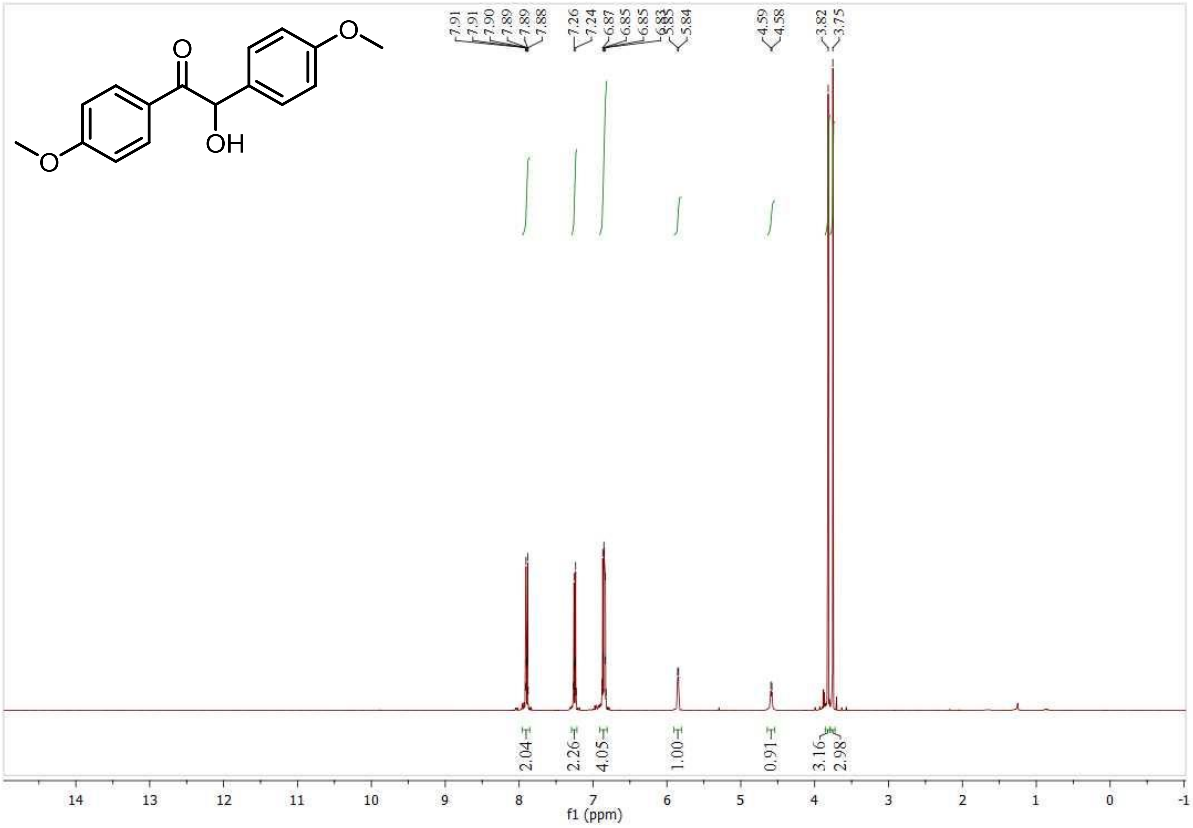

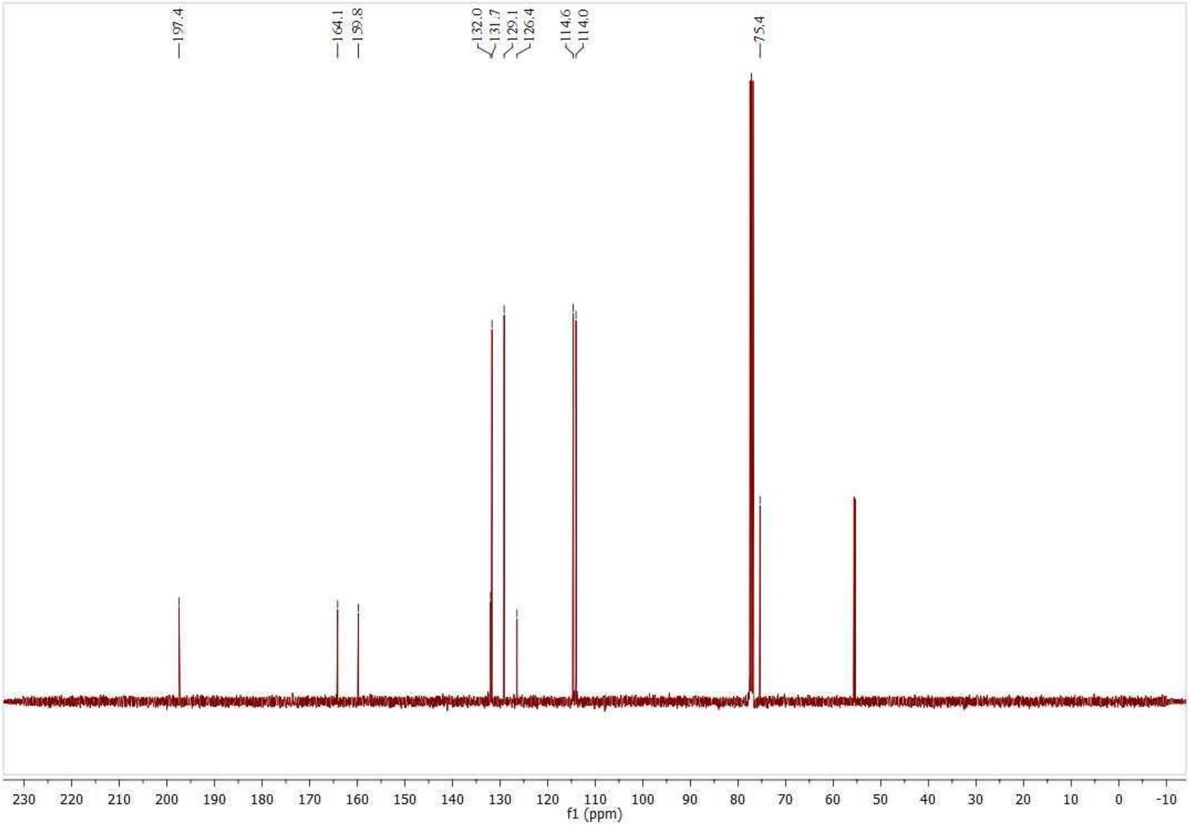

2-hydroxy-1,2-bis(4-(trifluoromethyl)phenyl)ethan-1-one (16)

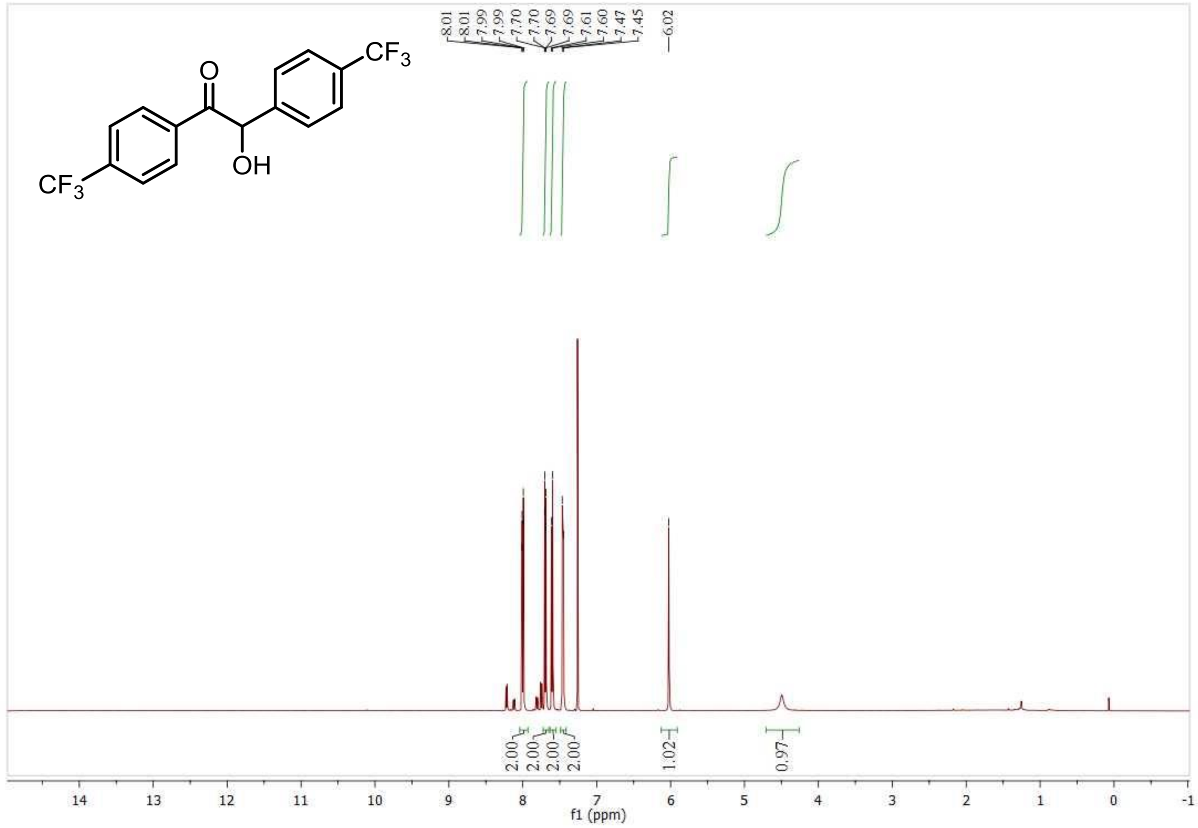

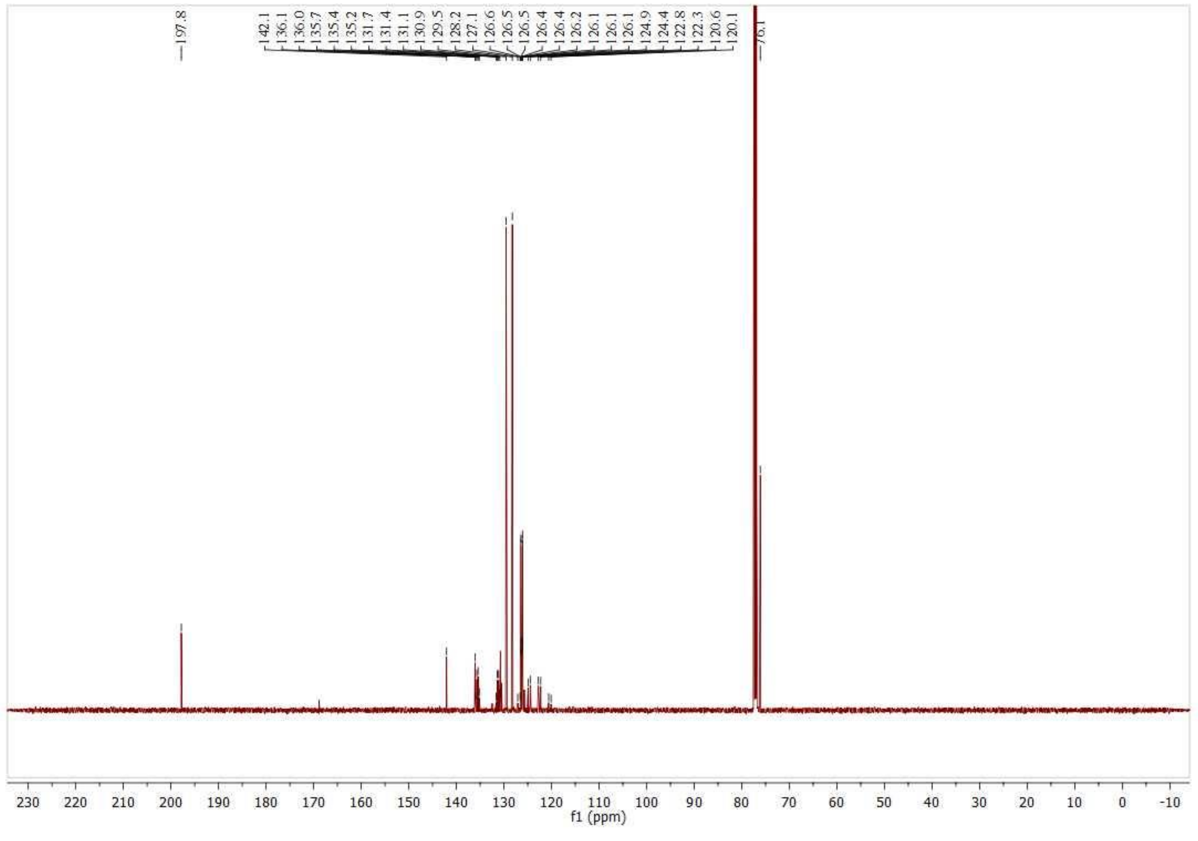

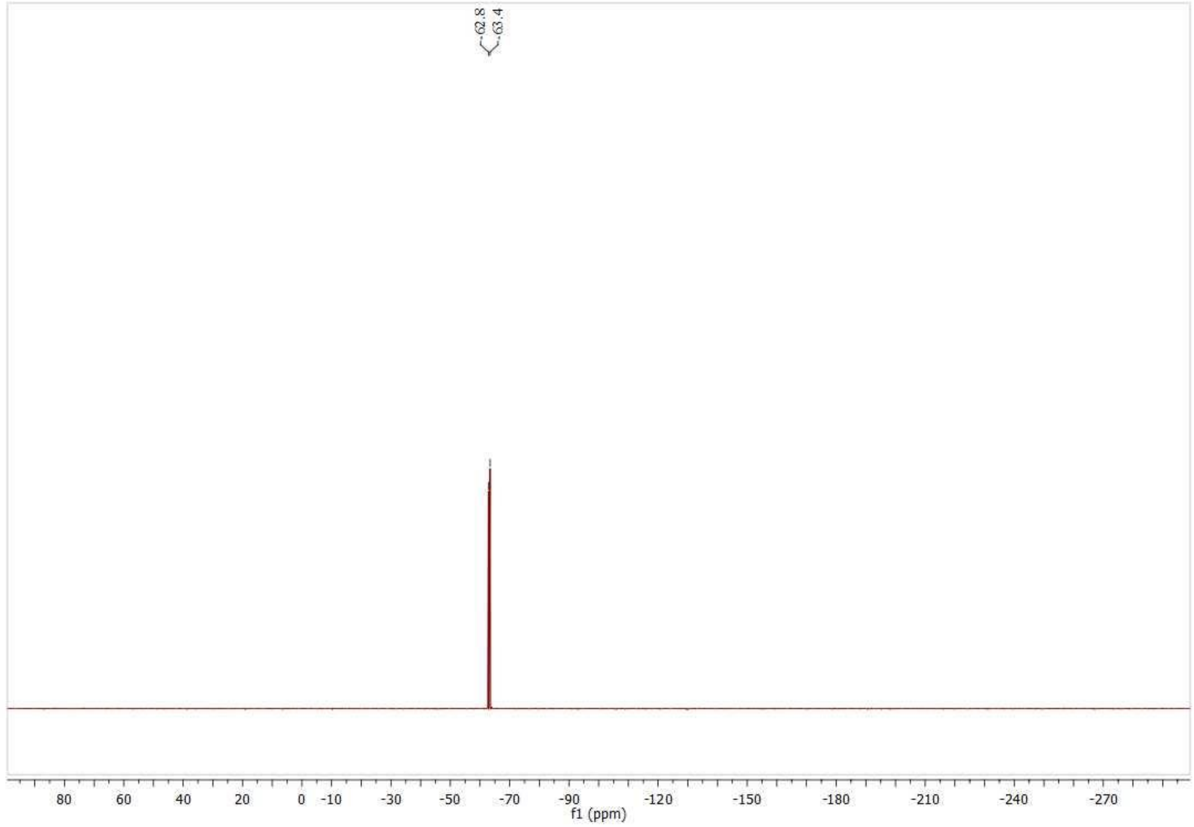

-62.8
-63.4
80 60 40 20 0 -10 -30 -50 -70 -90 -120 -150 -180 -210 -240 -270
f1 (ppm)

2-(2-oxo-2-phenylethoxy)benzaldehyde (17a)

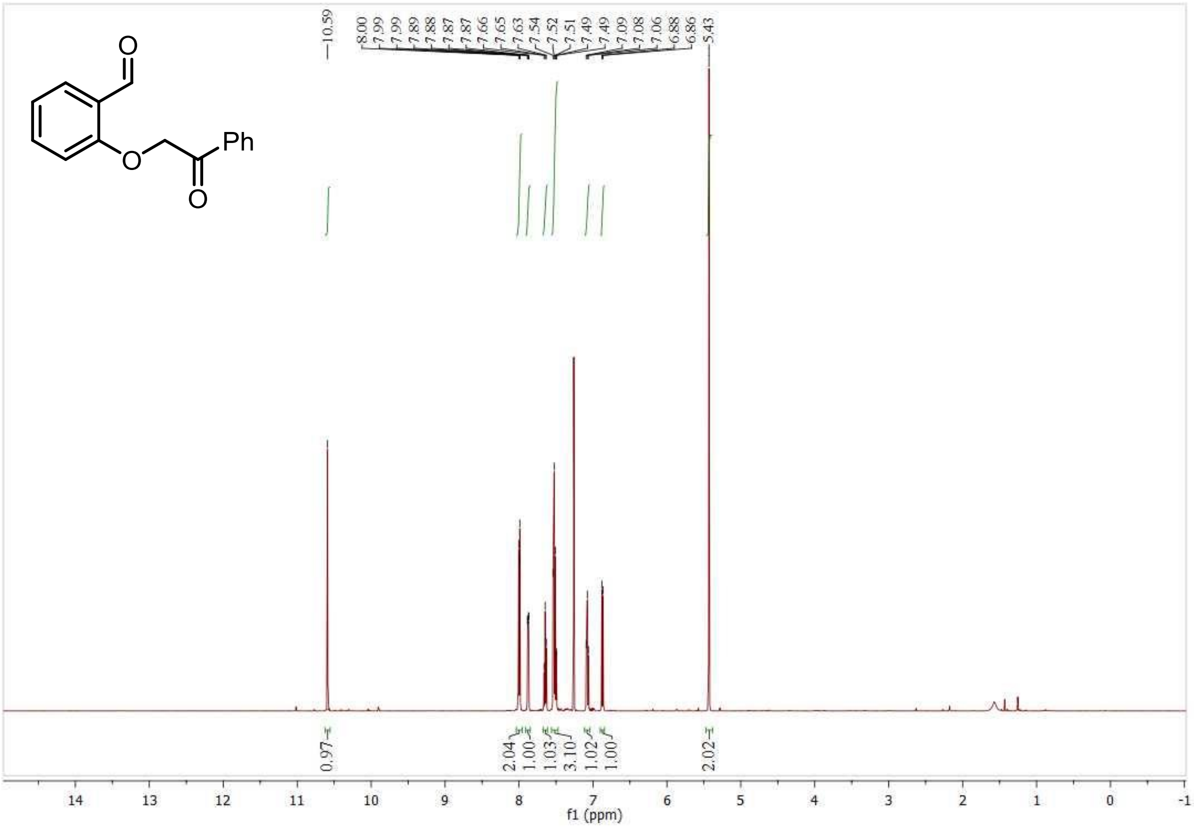

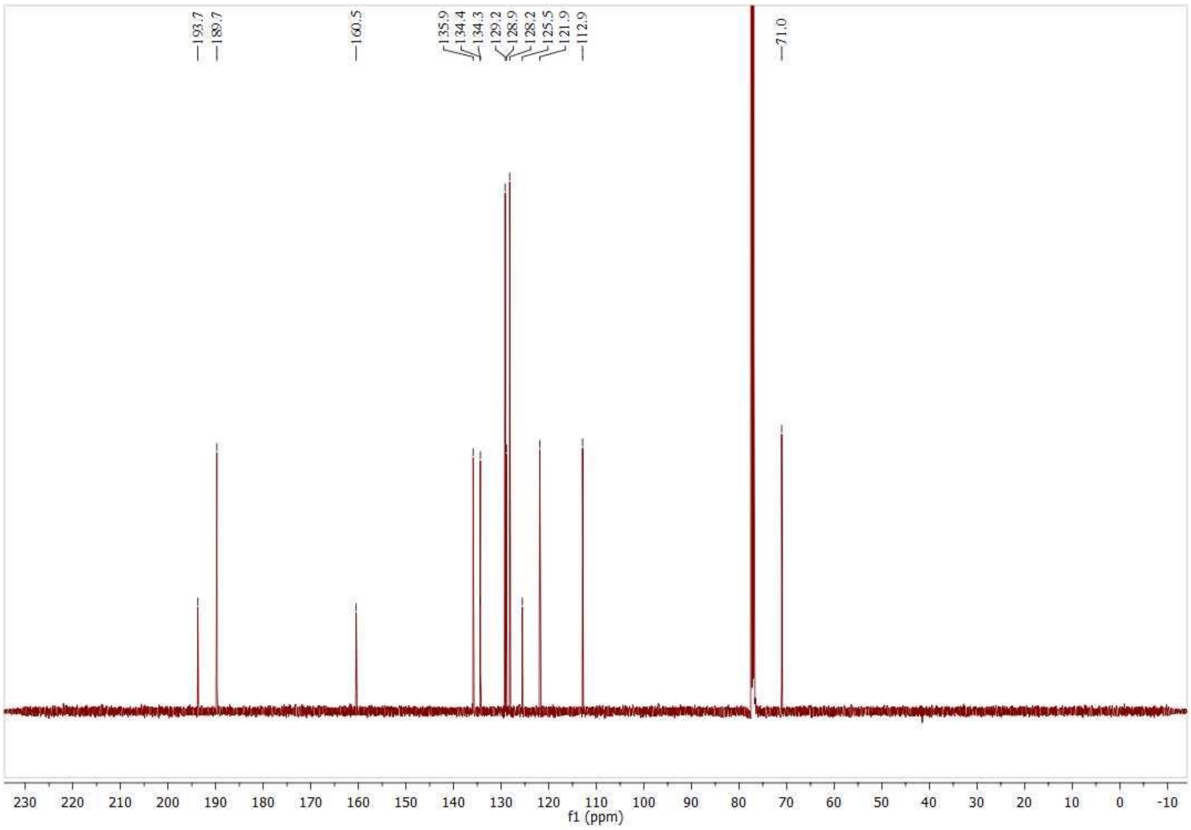

5-methyl-2-(2-oxo-2-phenylethoxy)benzaldehyde (19a)

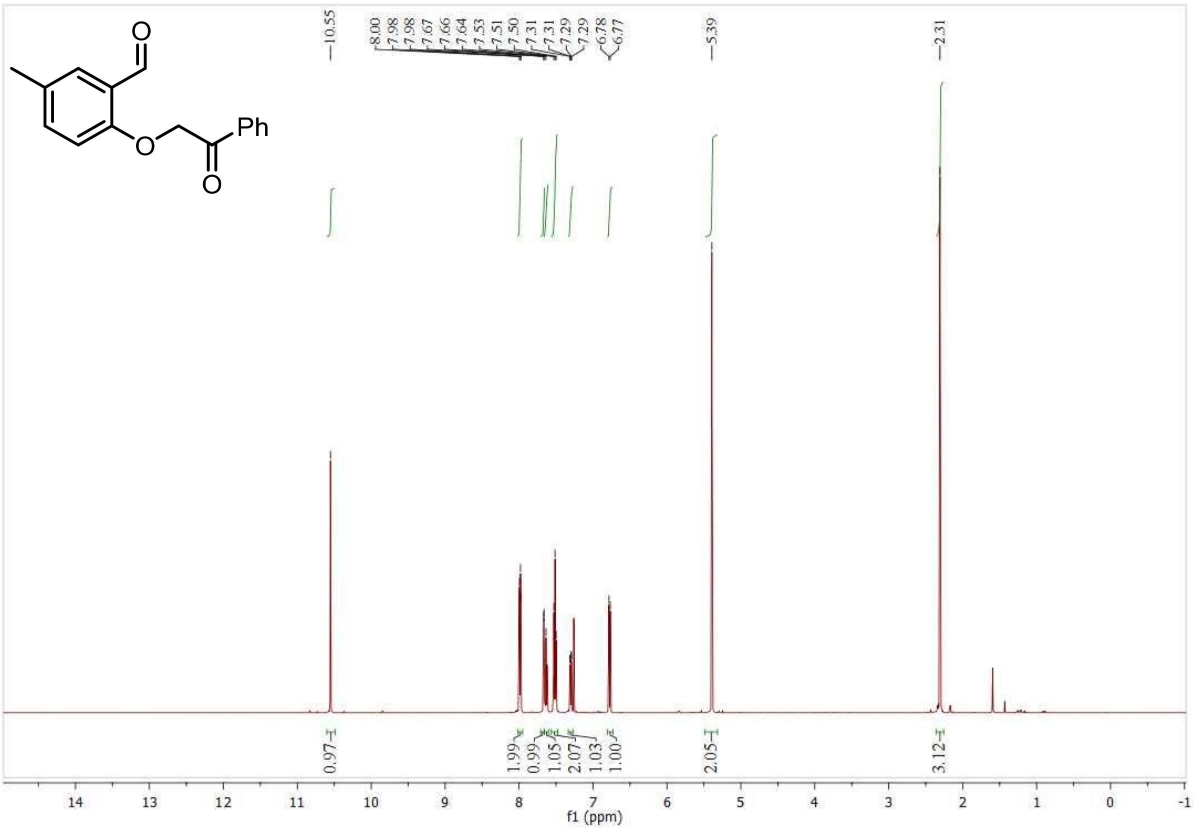

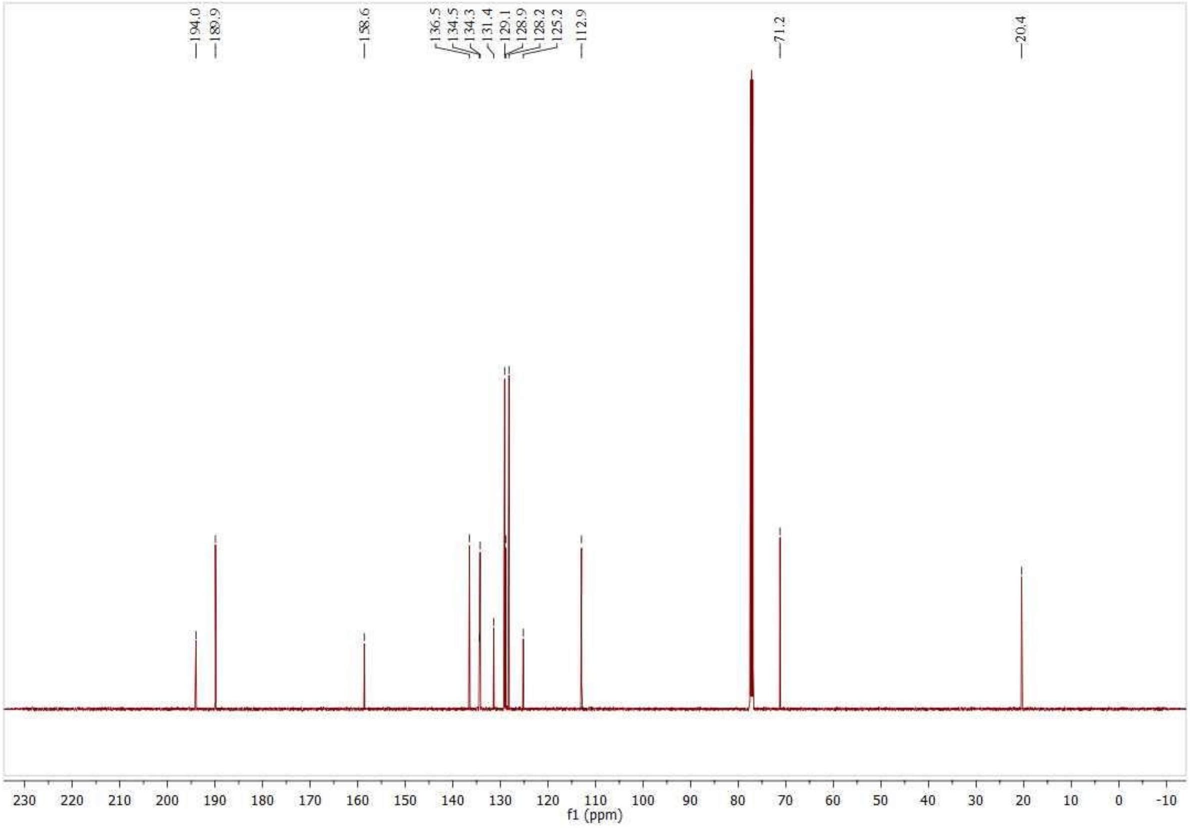

5-nitro-2-(2-oxo-2-phenylethoxy)benzaldehyde (18a)

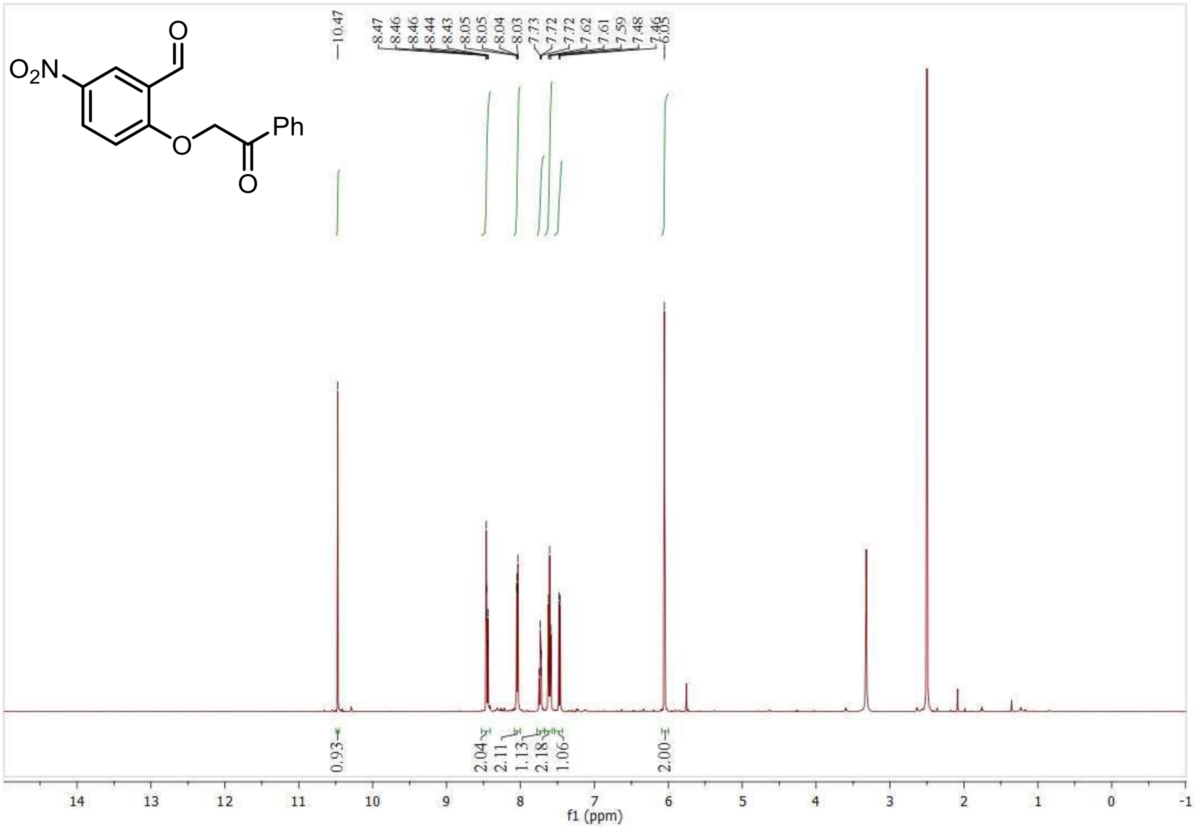

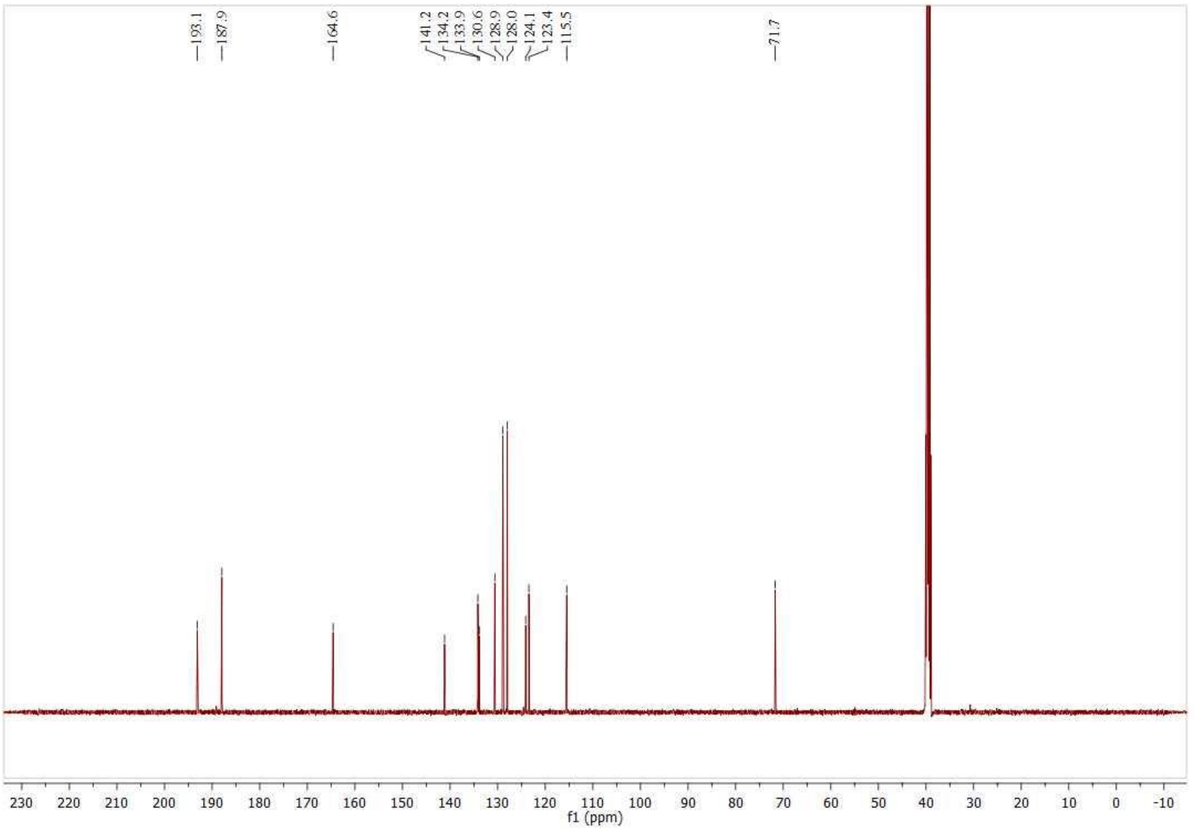

3-hydroxy-3-phenylchroman-4-one (17)

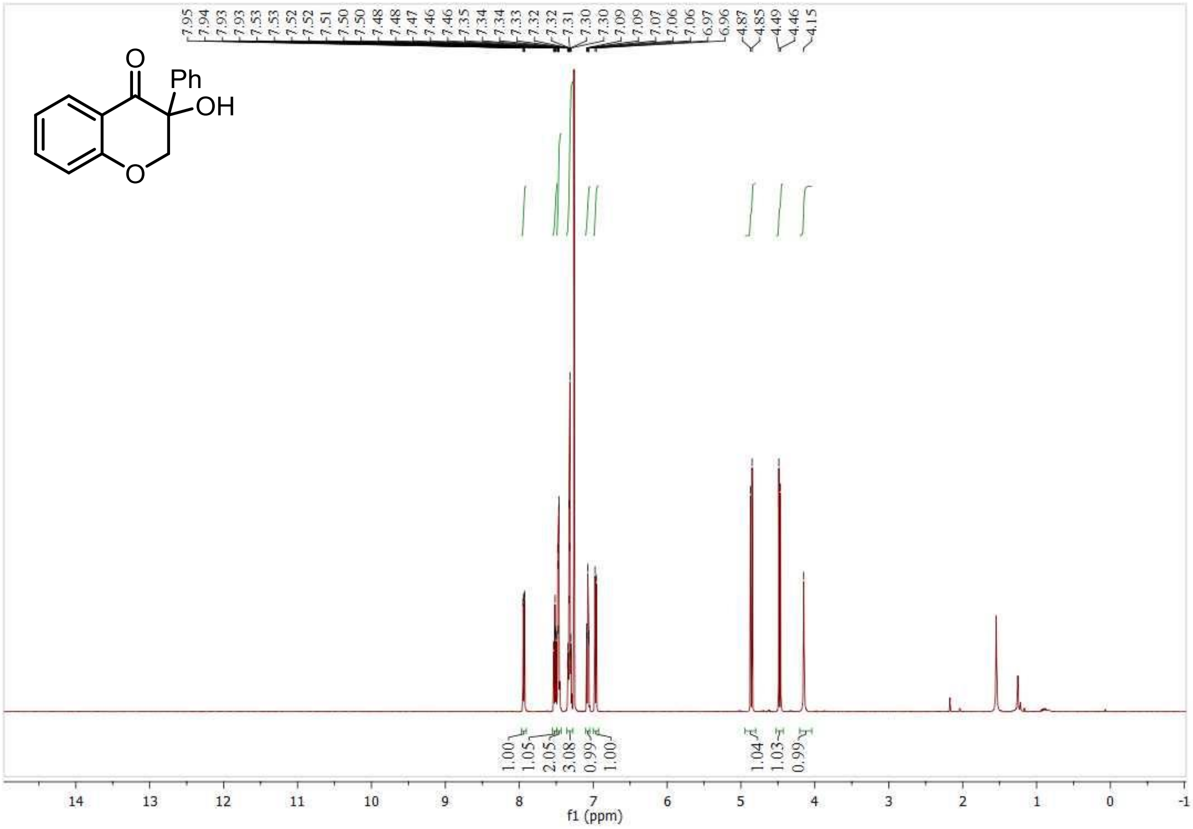

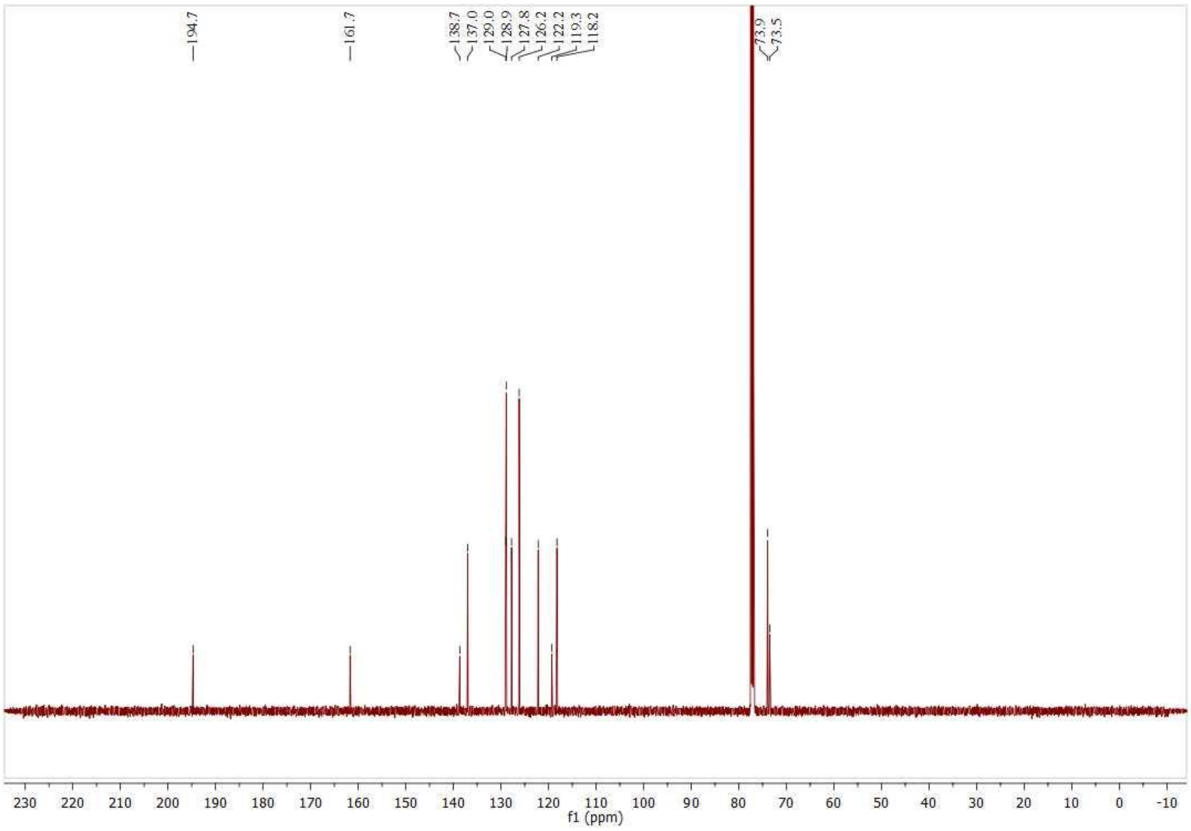

3-hydroxy-6-methyl-3-phenylchroman-4-one (19)

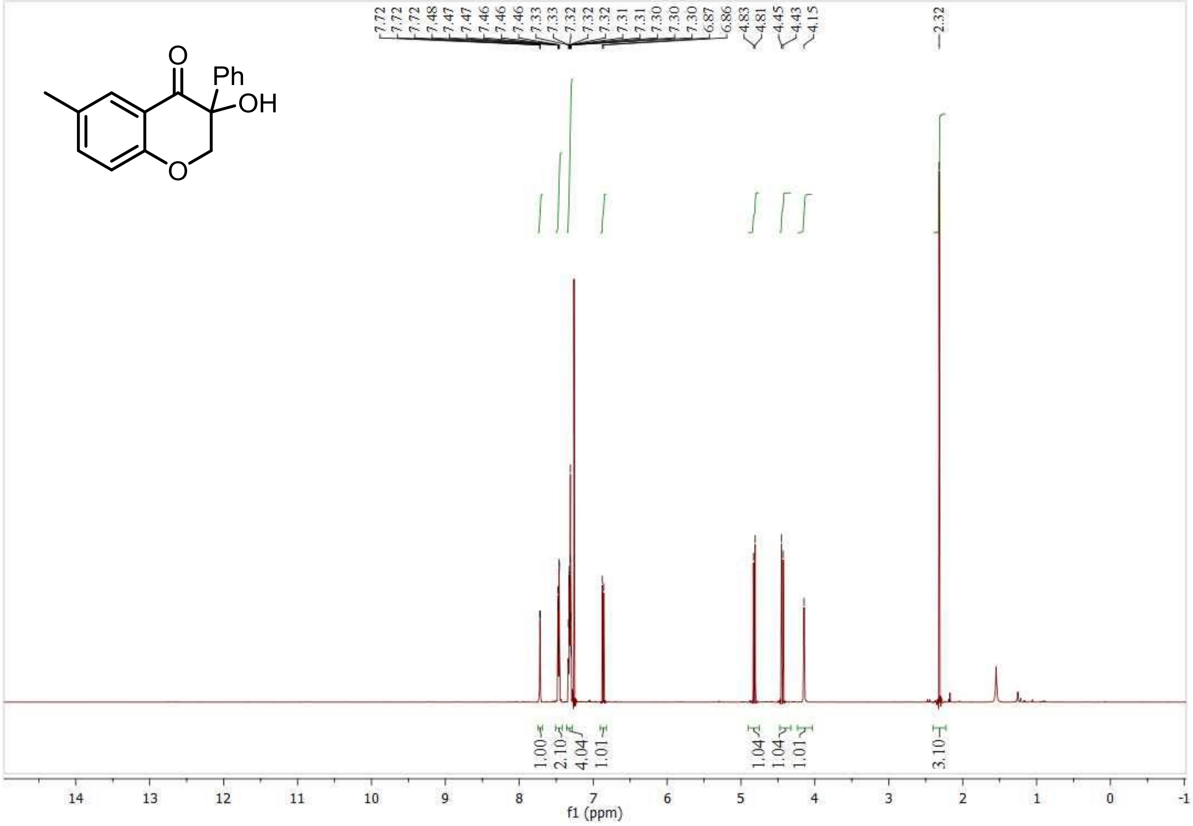

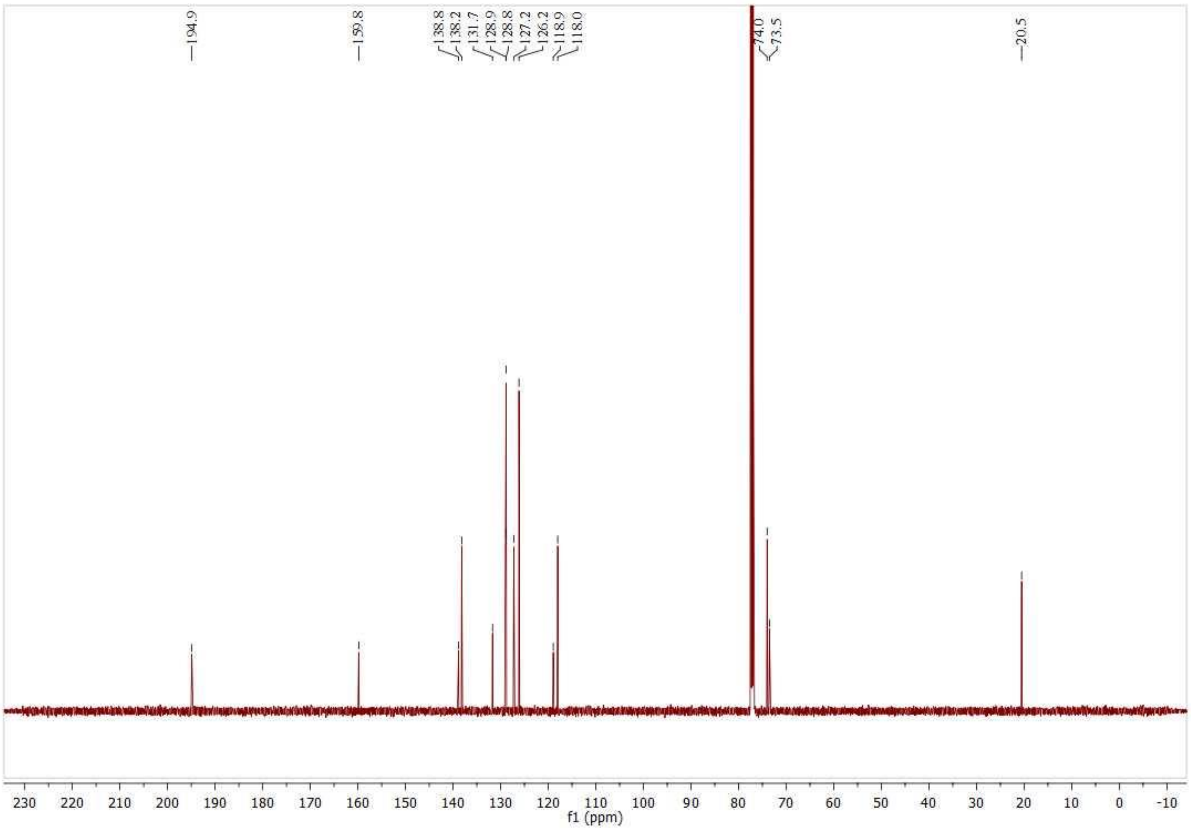

3-hydroxy-6-nitro-3-phenylchroman-4-one (18)

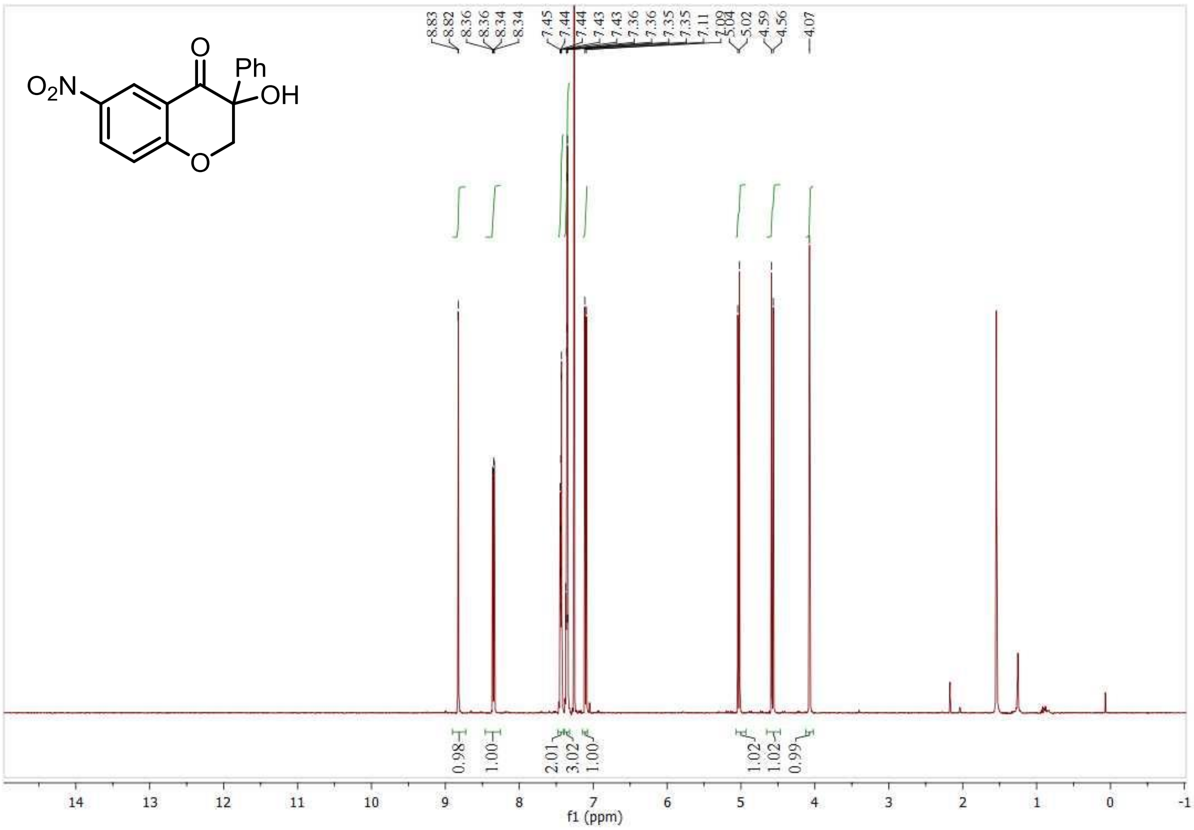

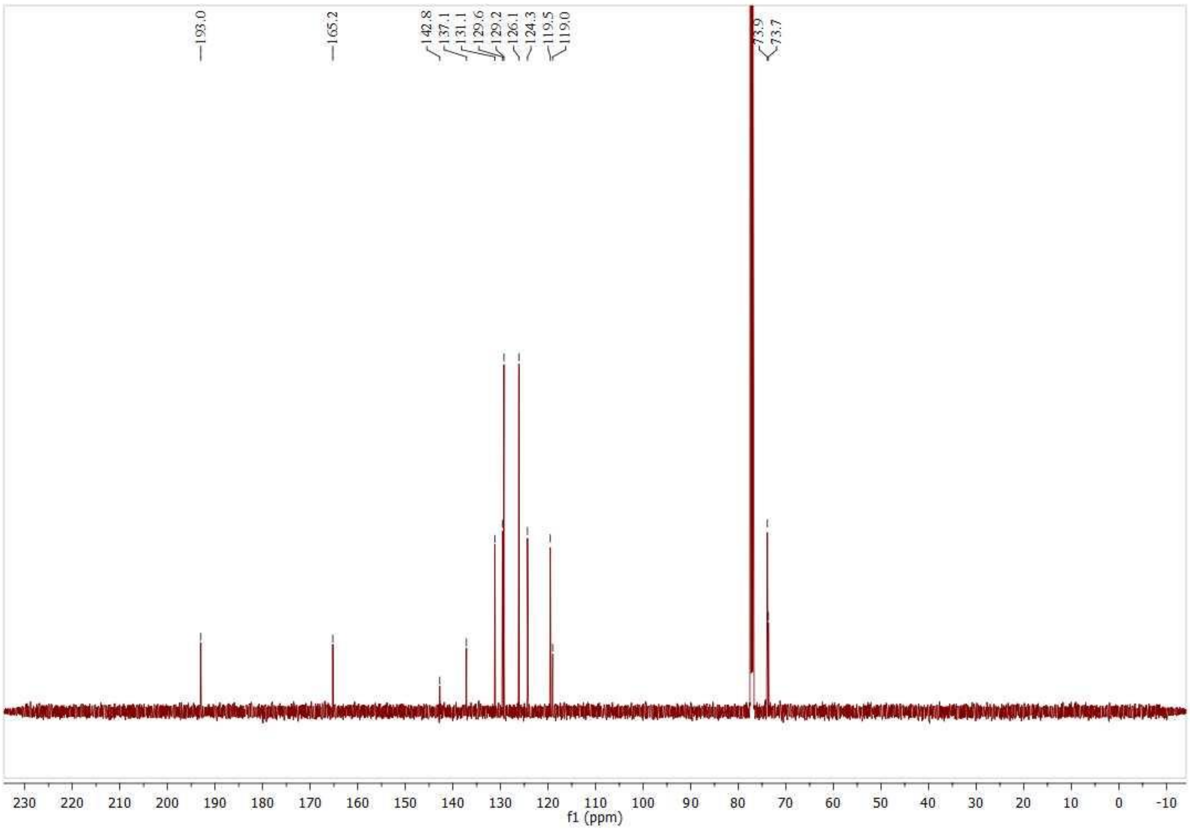

ethyl (E)-4-(2-formylphenoxy)but-2-enoate (29b)

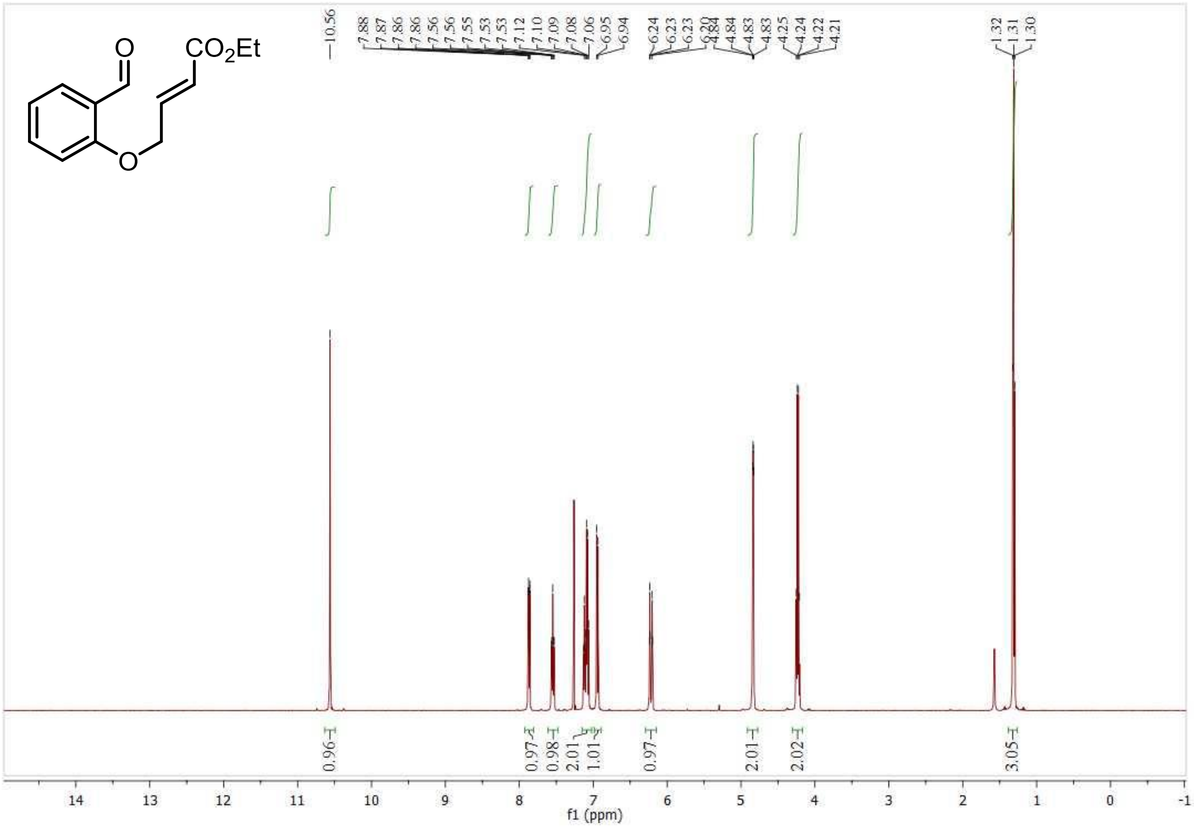

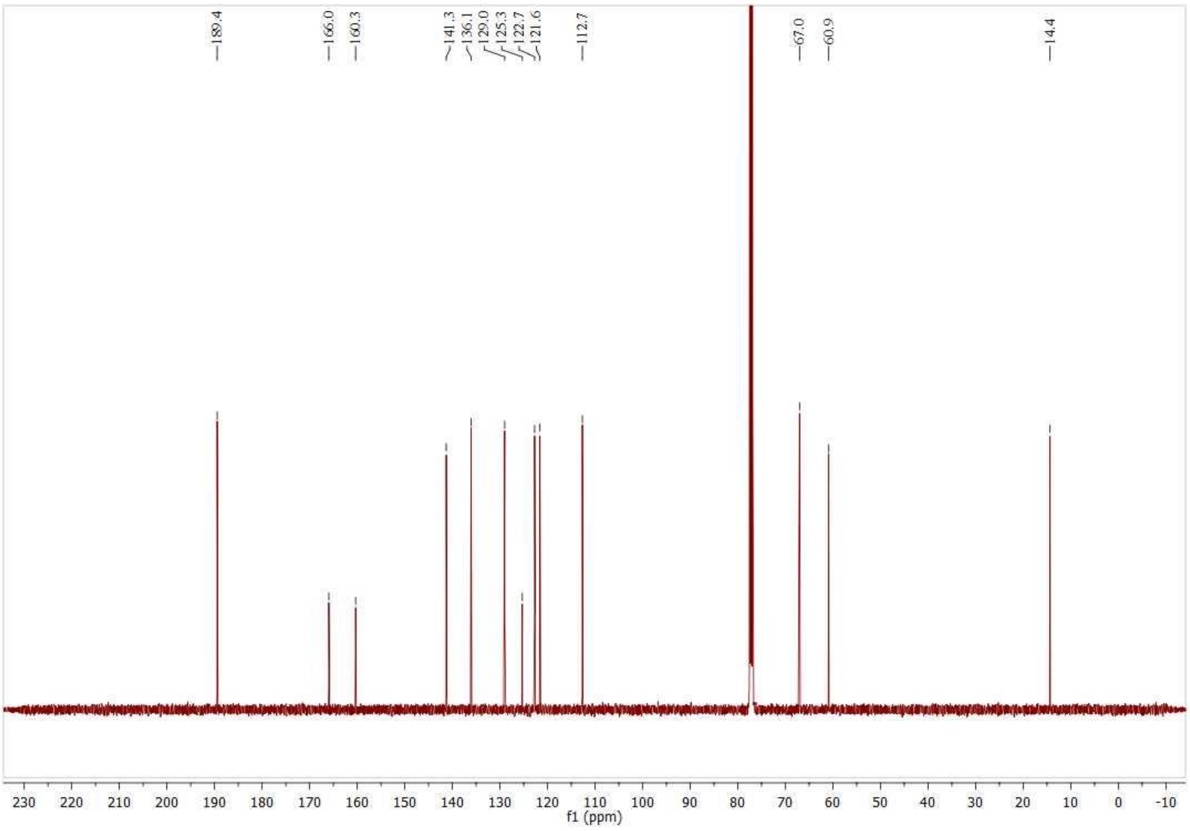

ethyl (E)-4-(2-formyl-4-methylphenoxy)but-2-enoate (30a)

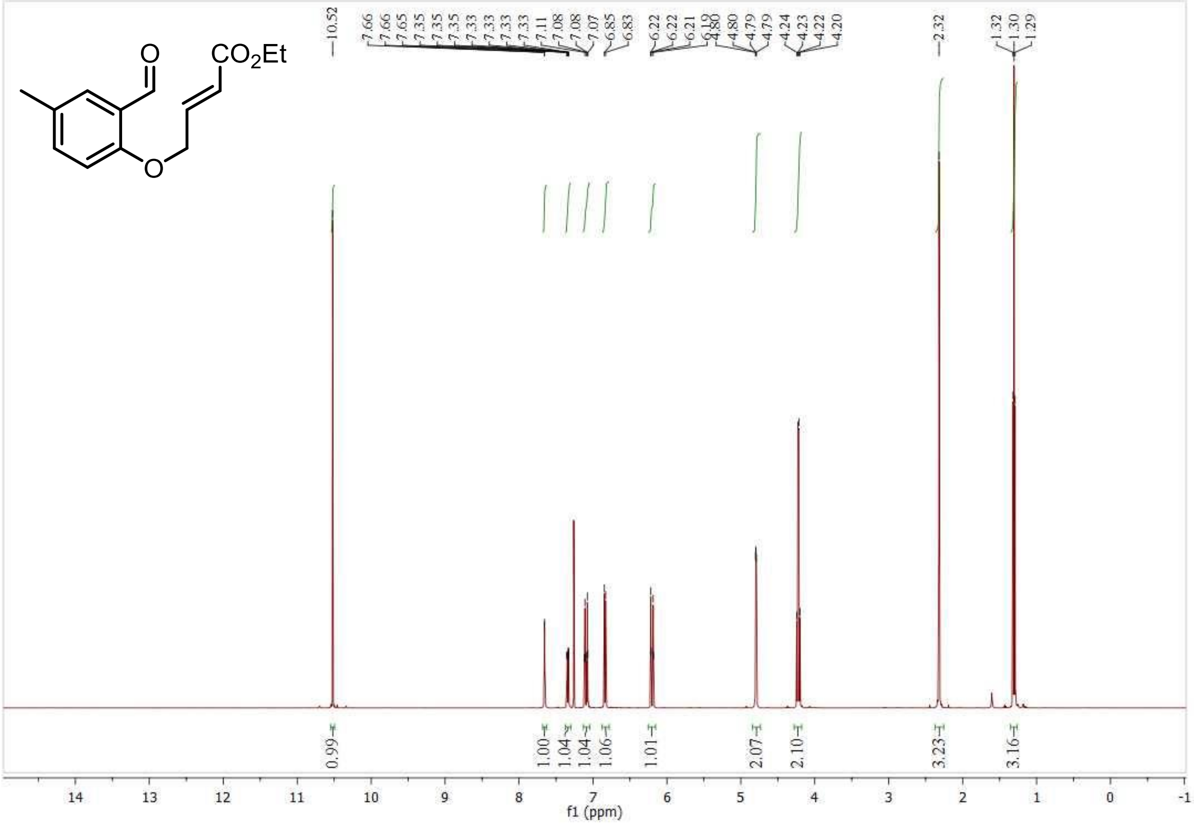

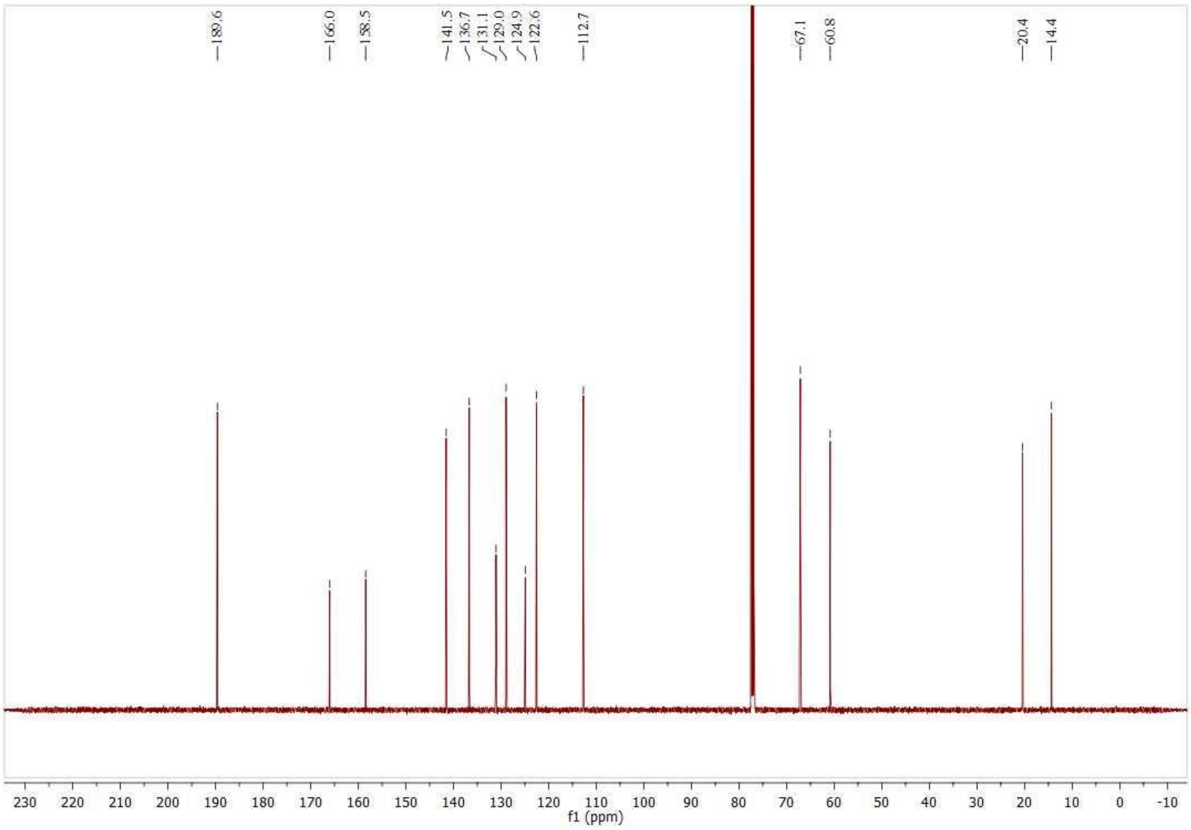

ethyl (E)-4-(4-bromo-2-formylphenoxy)but-2-enoate (31a)

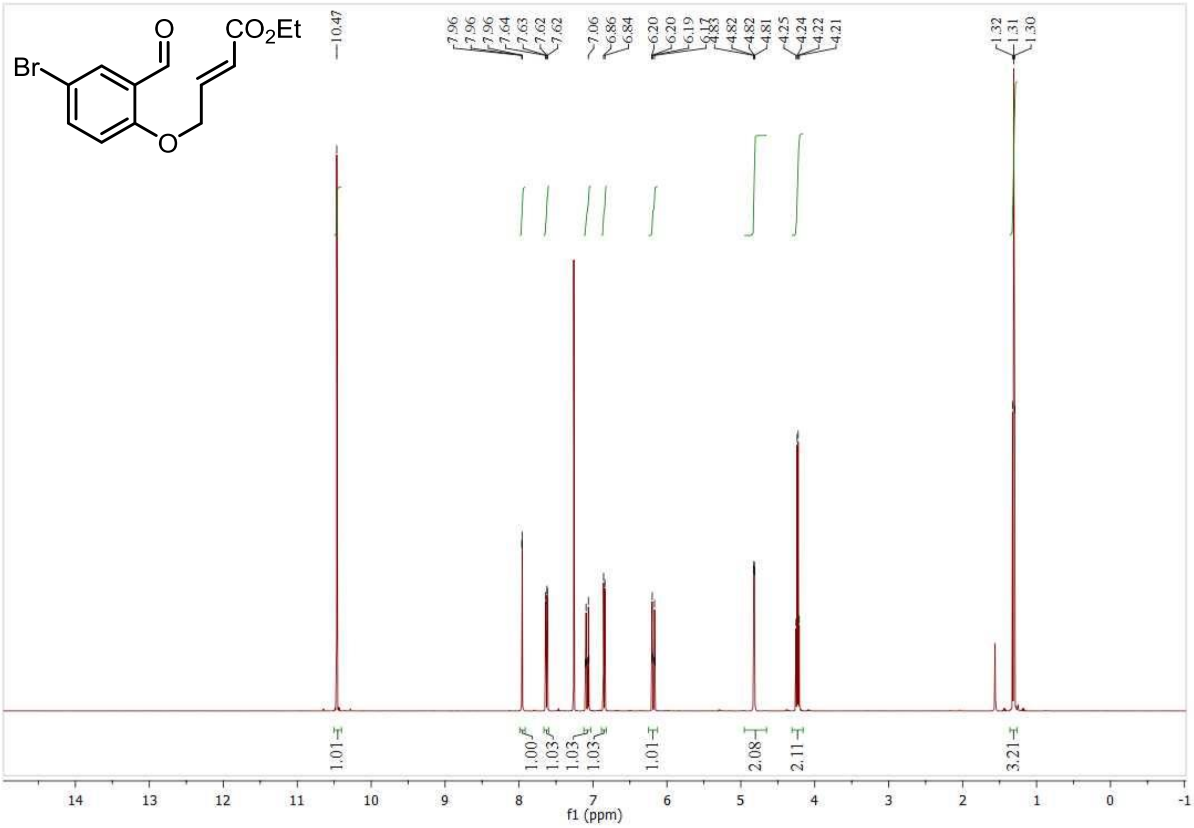

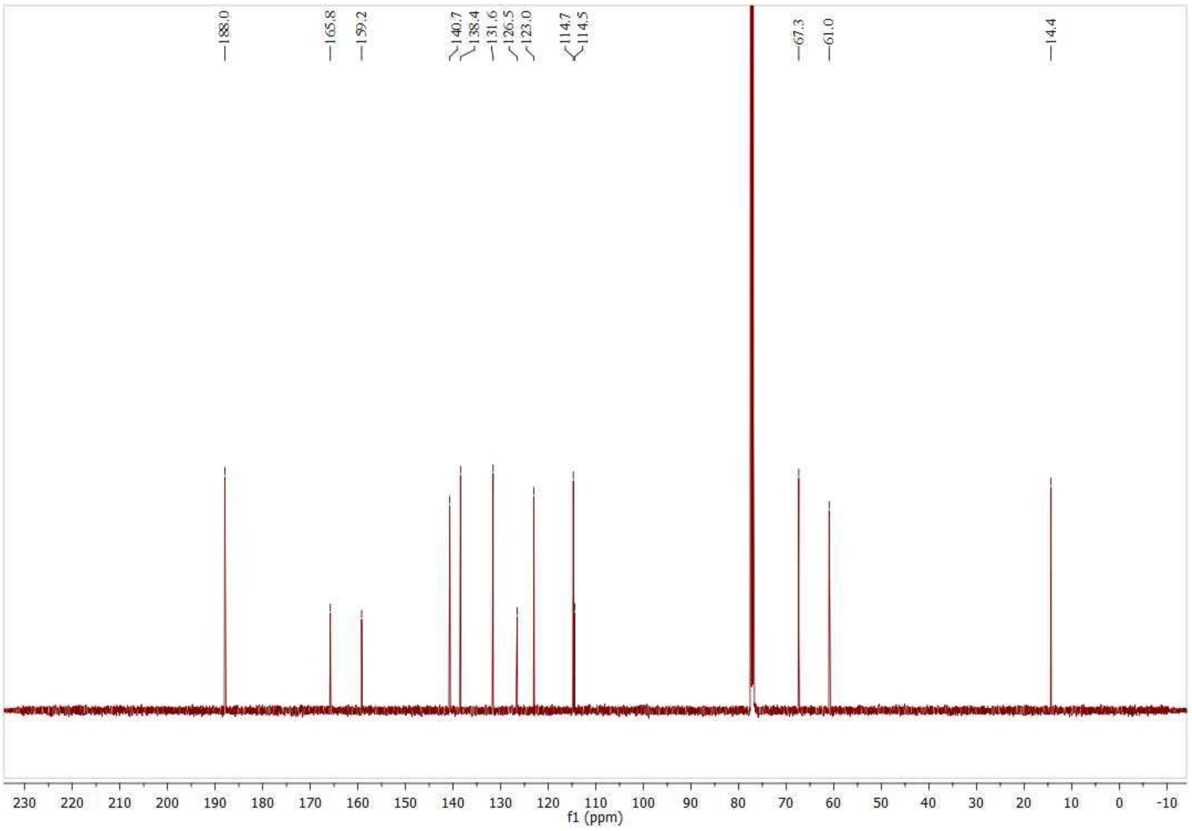

dimethyl 2-(2-formylphenoxy)maleate (32a)

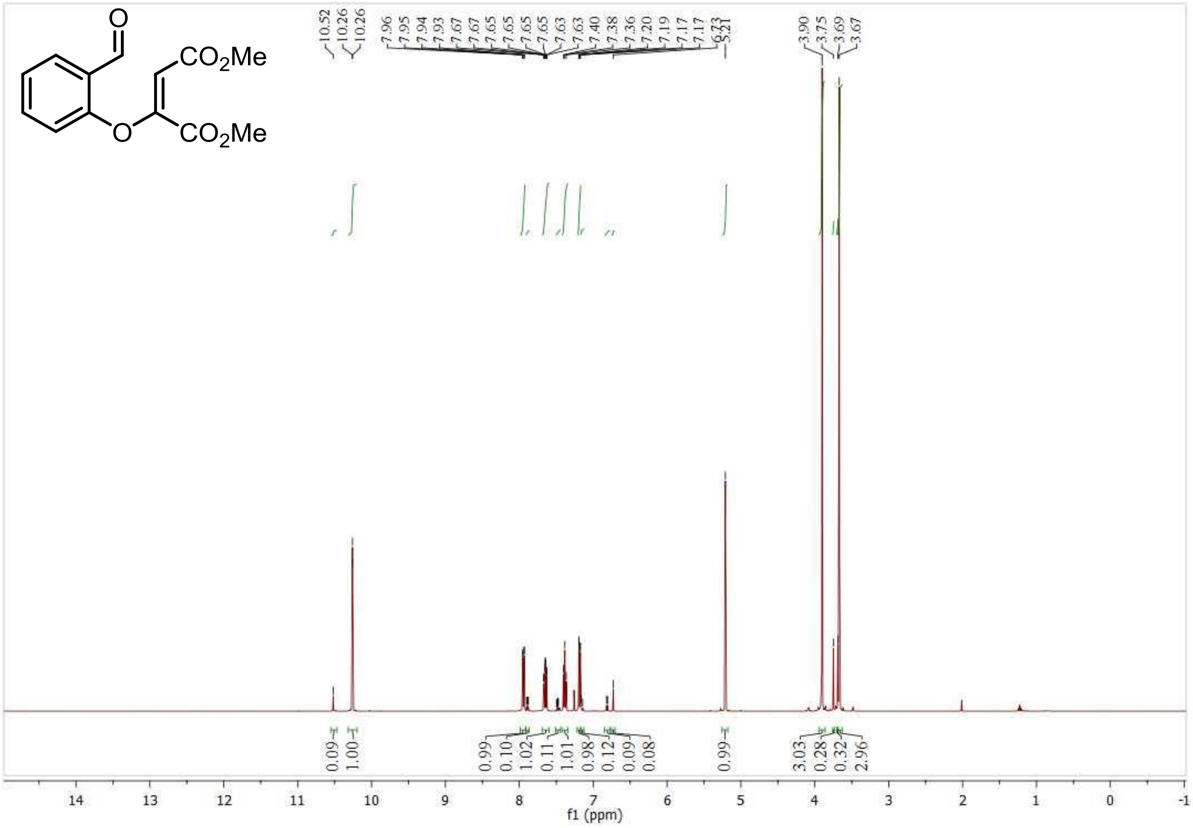

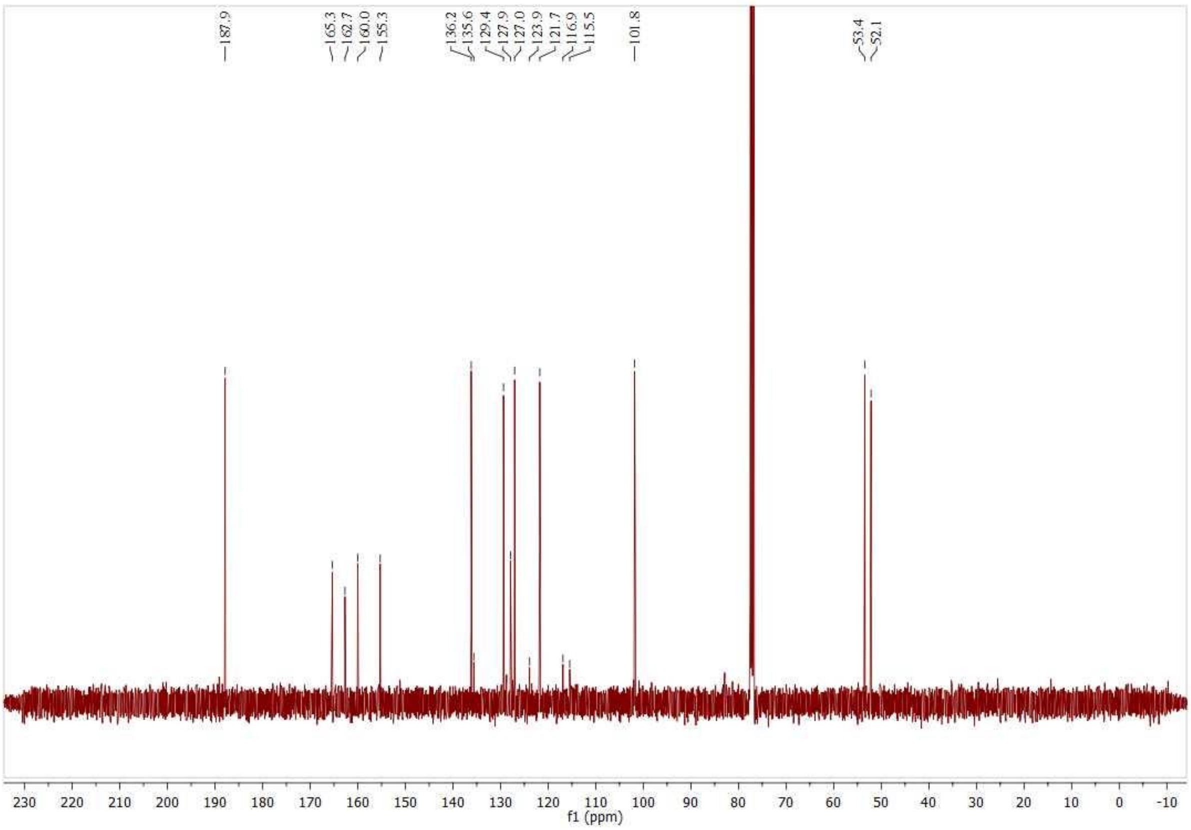

dimethyl 2-(2-formyl-4-methylphenoxy)maleate (33a)

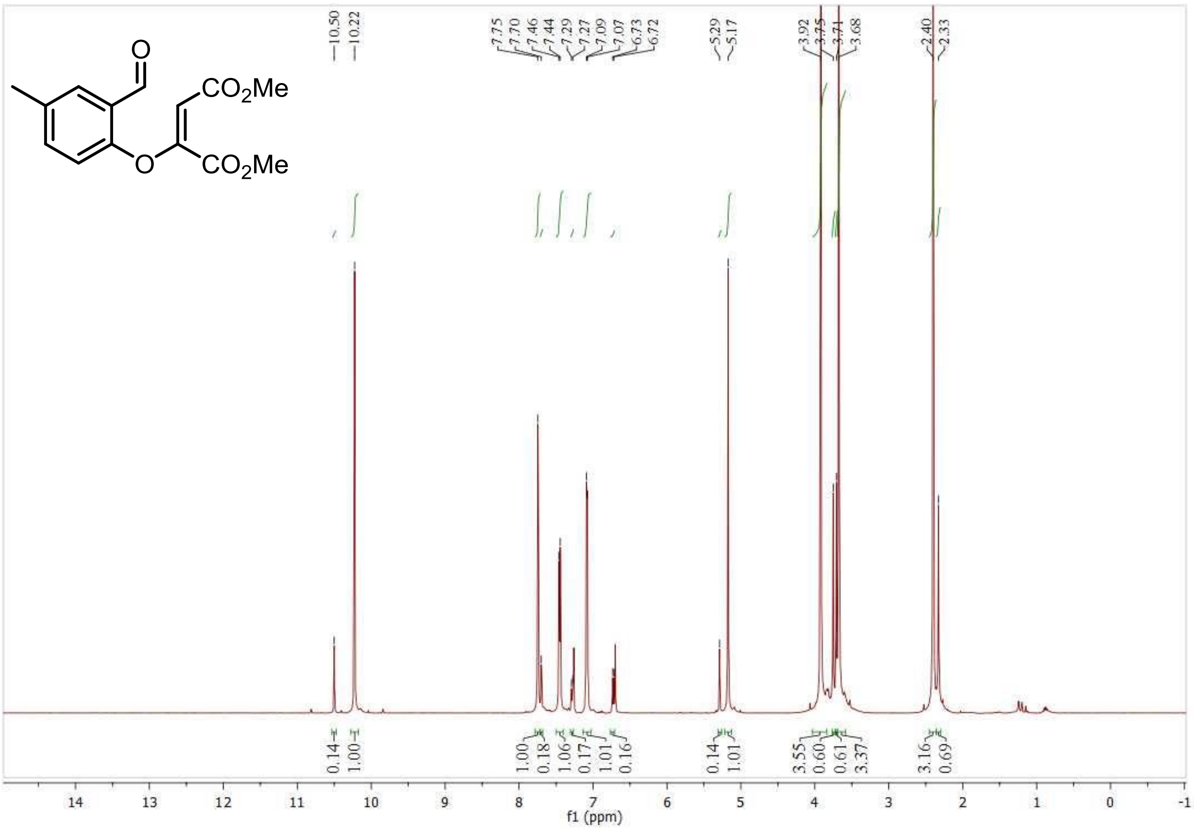

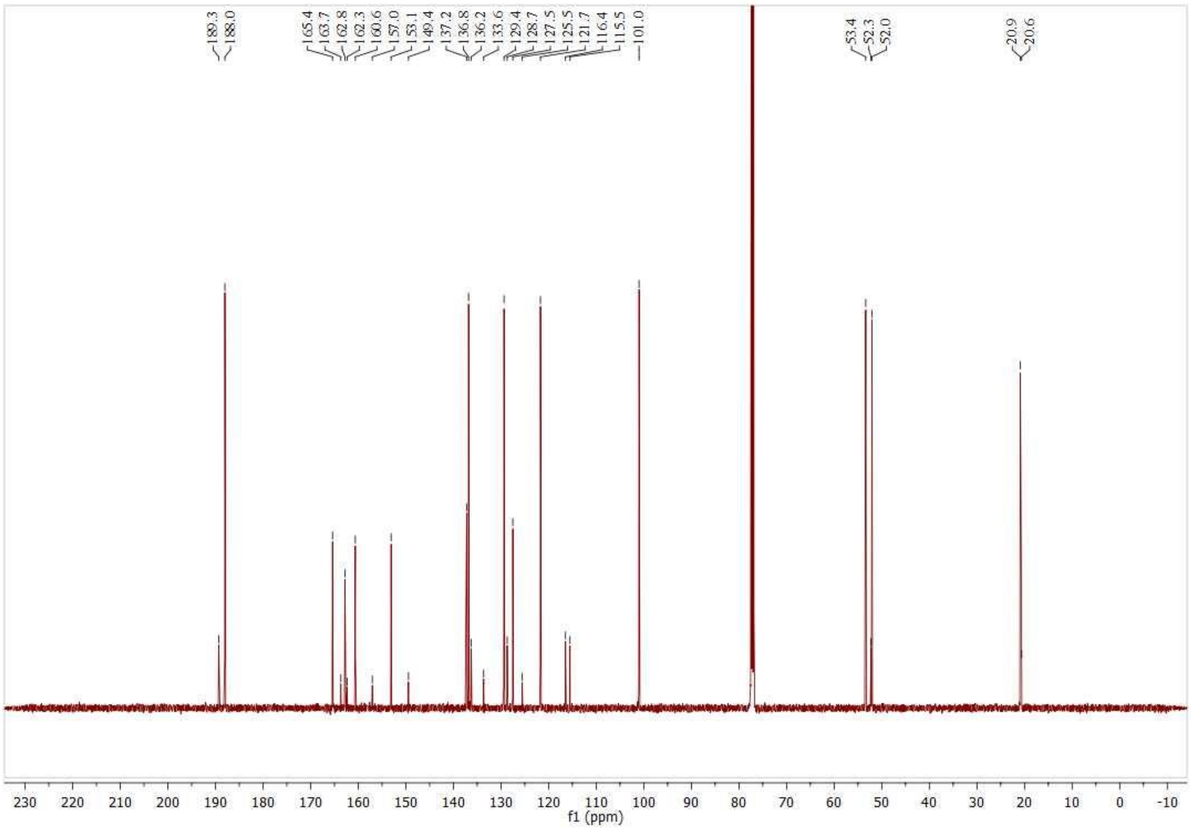

dimethyl 2-(4-bromo-2-formylphenoxy)maleate (34a)

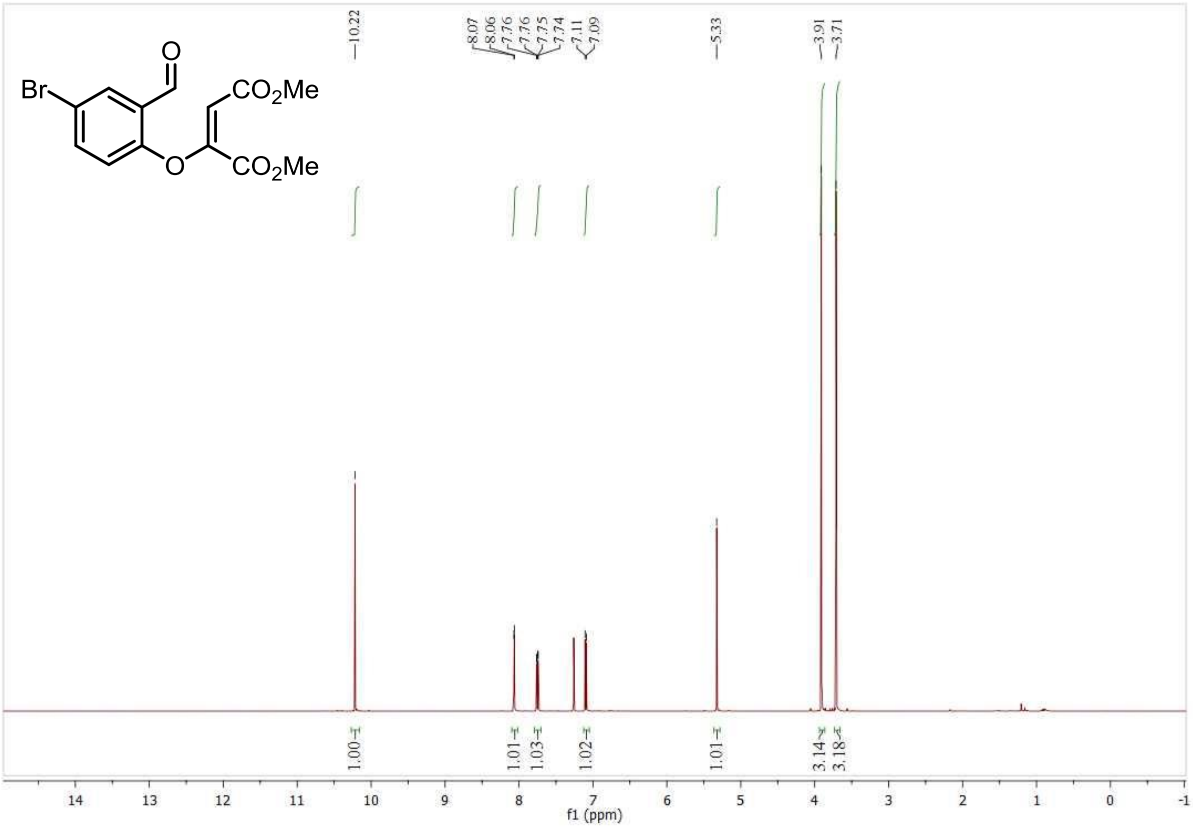

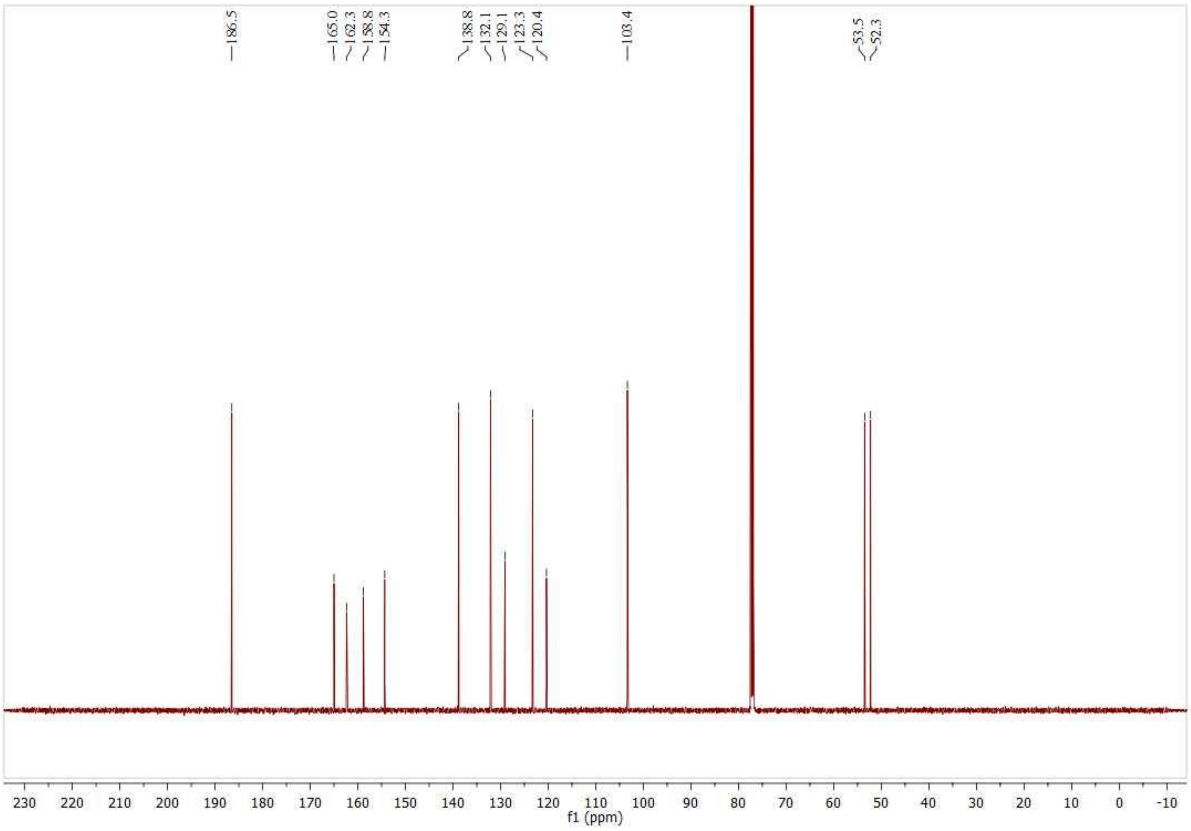

ethyl 2-(4-oxochroman-3-yl)acetate (29)

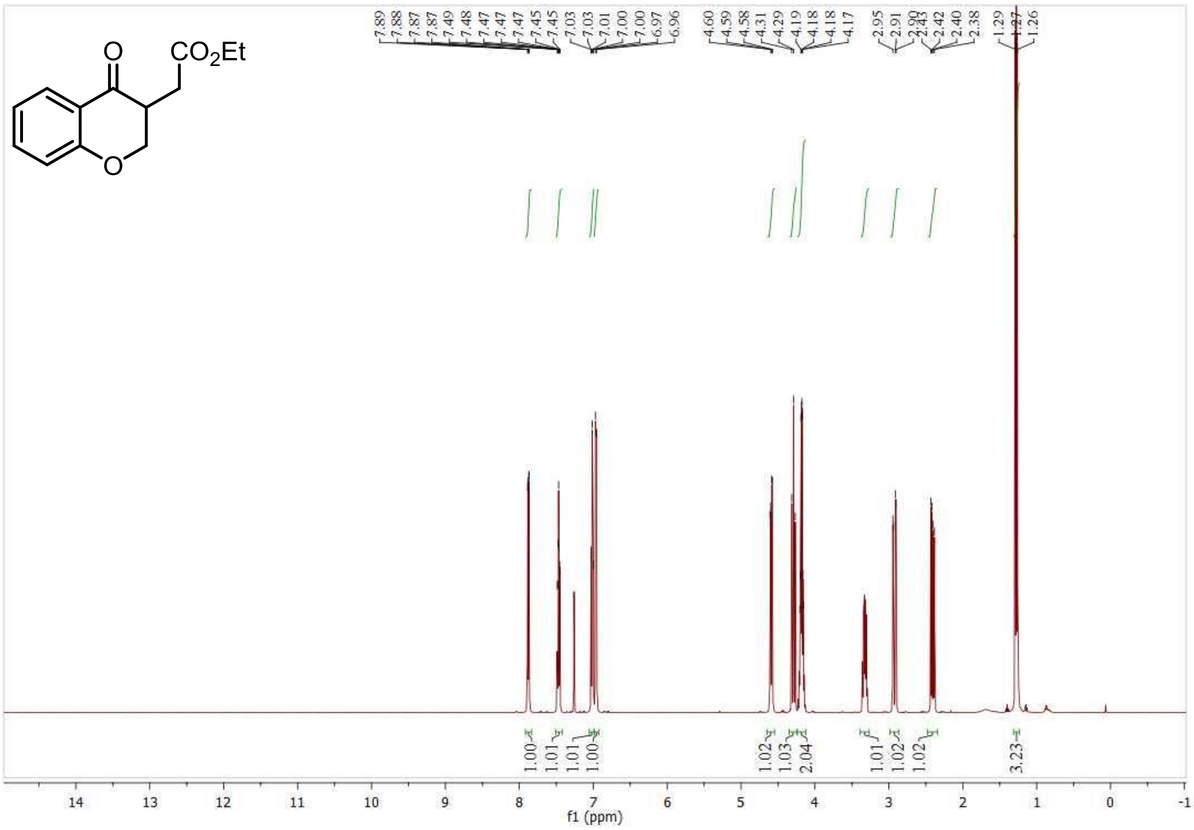

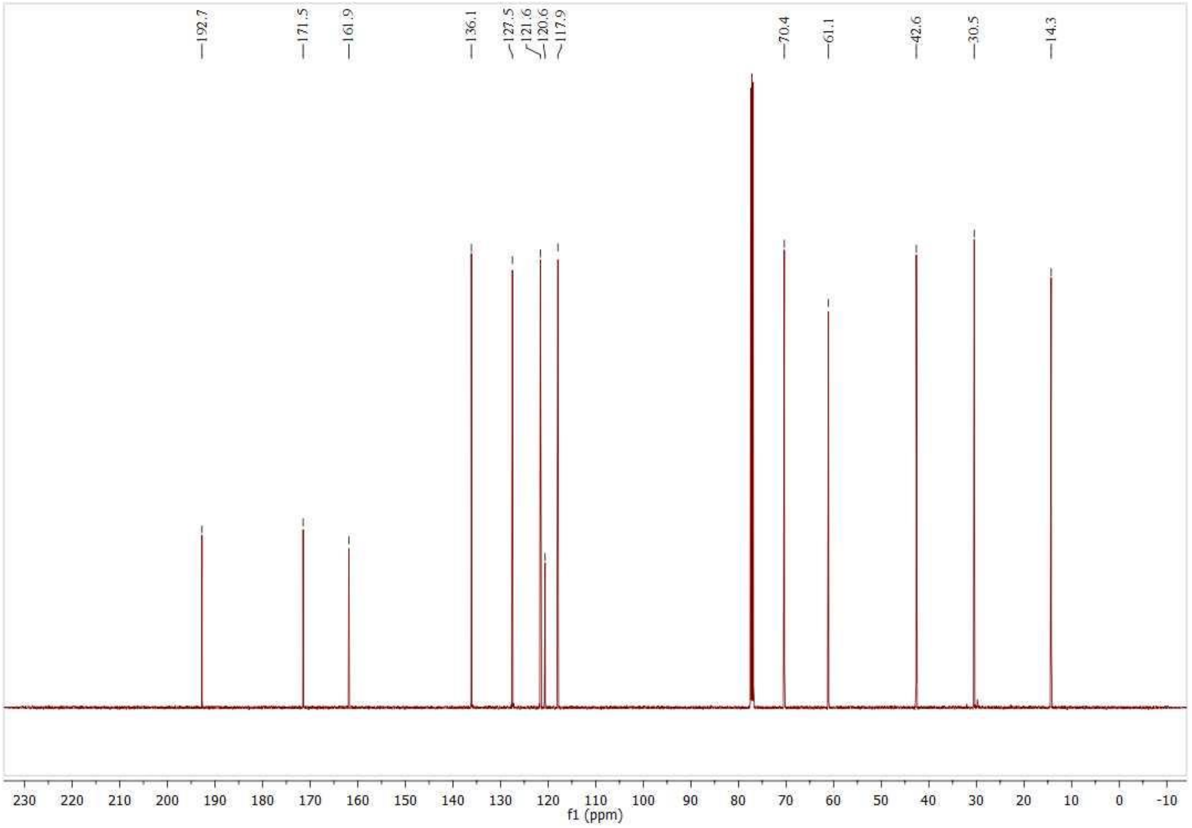

ethyl 2-(6-methyl-4-oxochroman-3-yl)acetate (30)

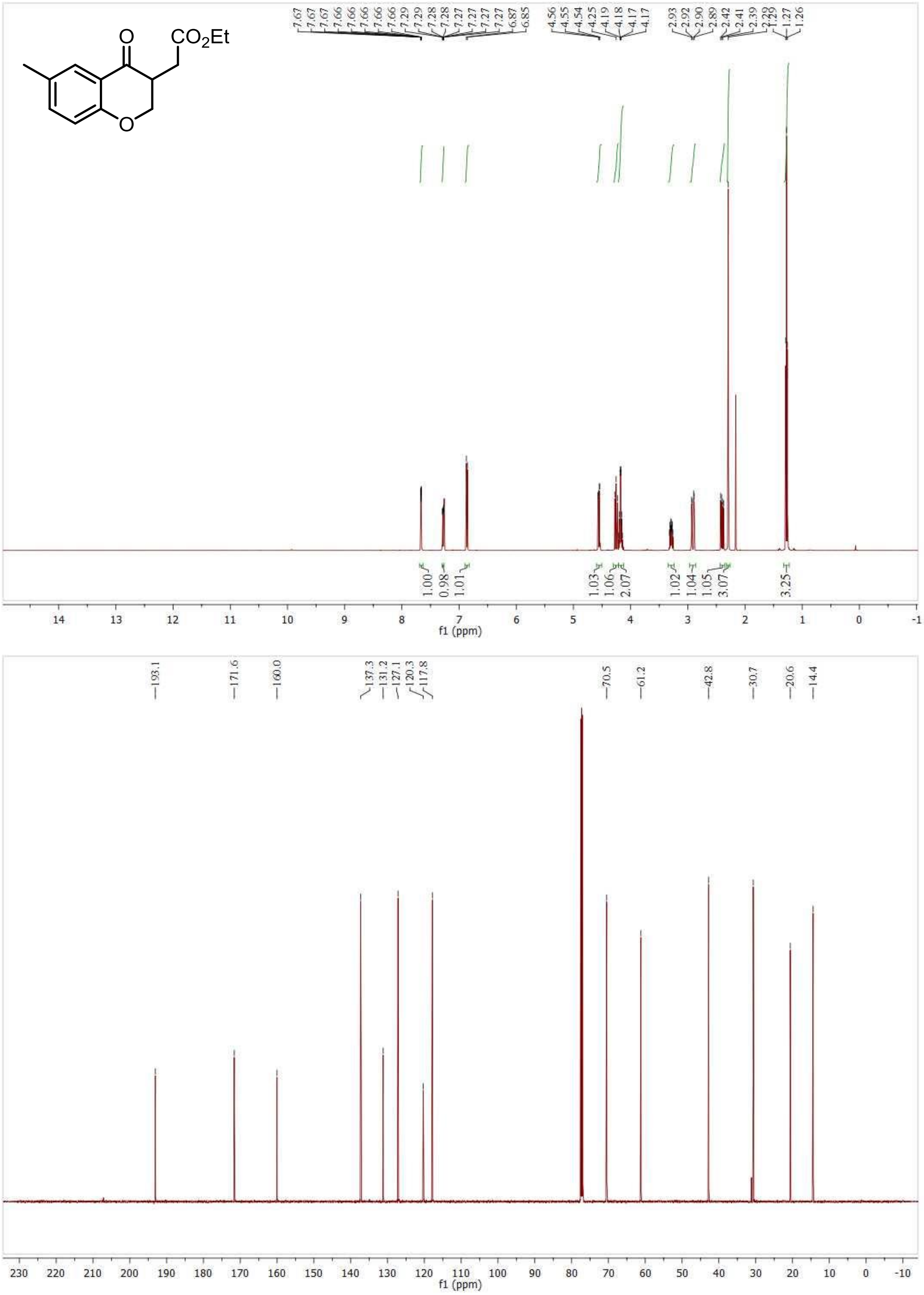

ethyl 2-(6-bromo-4-oxochroman-3-yl)acetate (31)

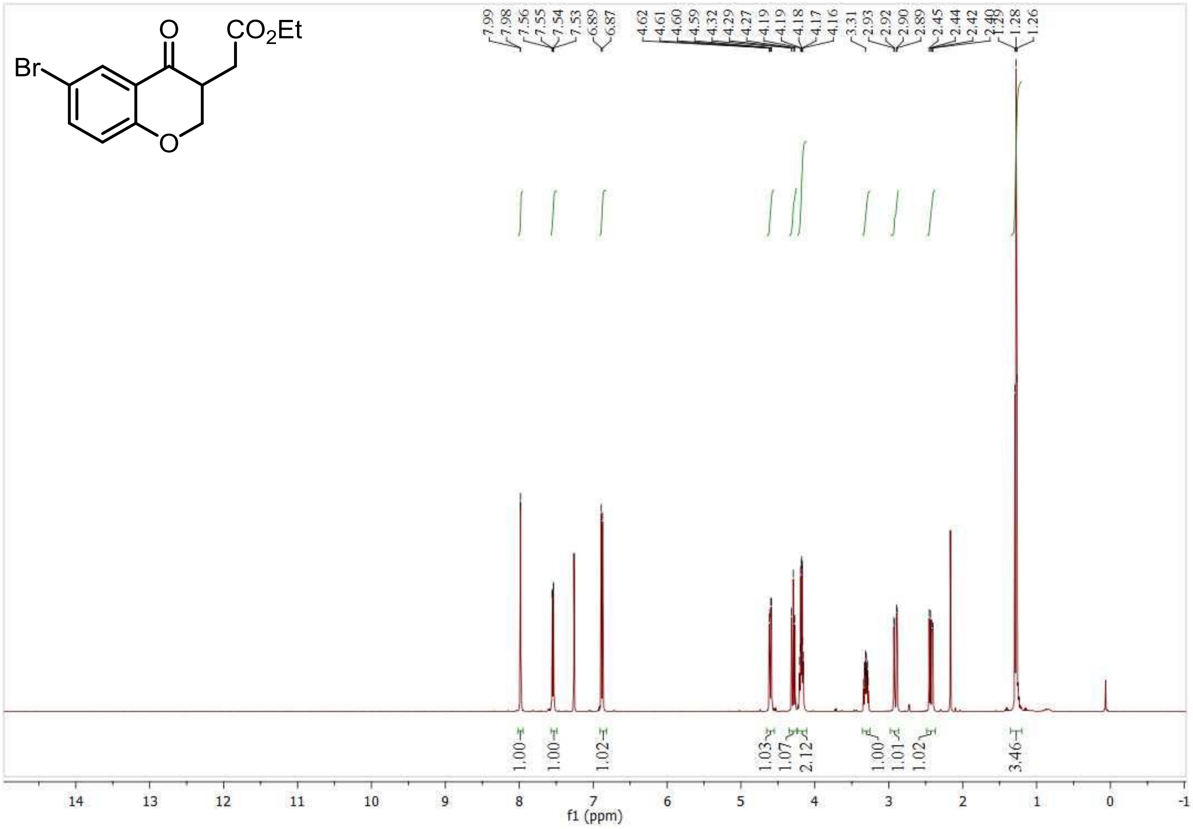

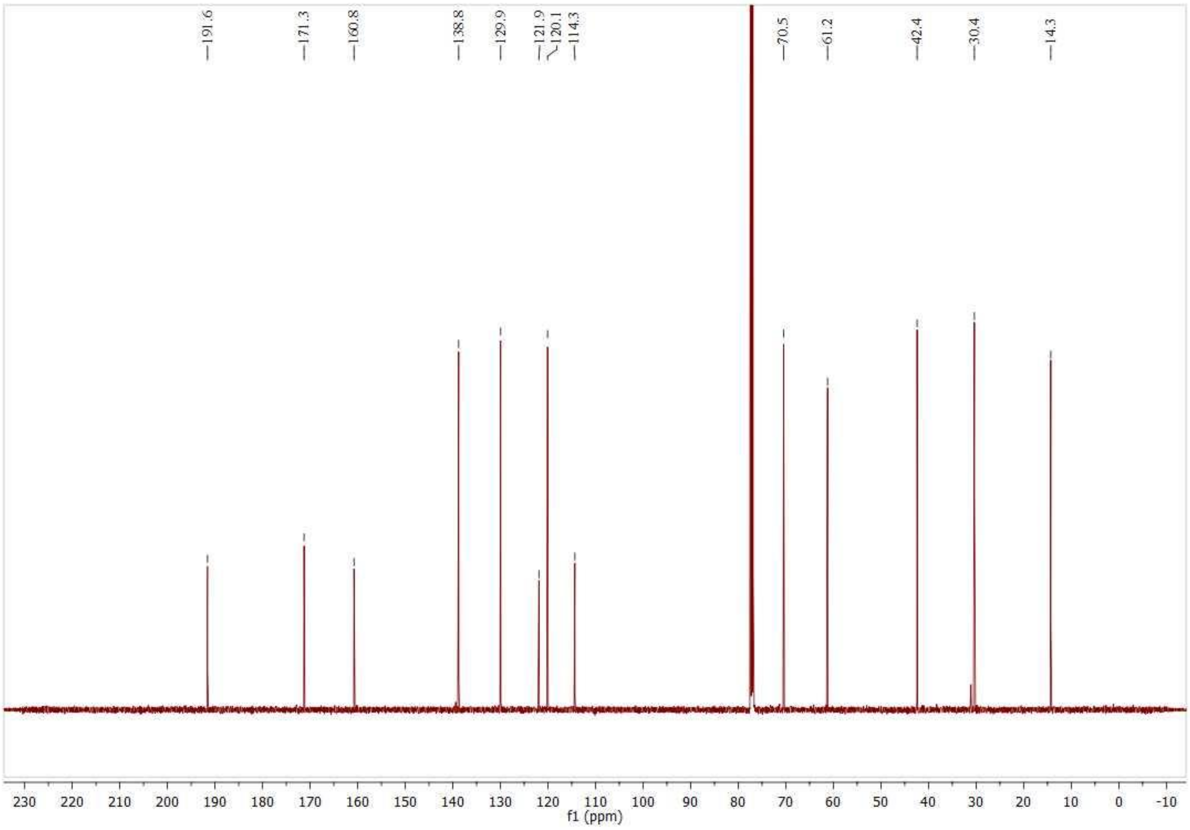

methyl 2-(2-methoxy-2-oxoethyl)-3-oxo-2,3-dihydrobenzofuran-2-carboxylate (32)

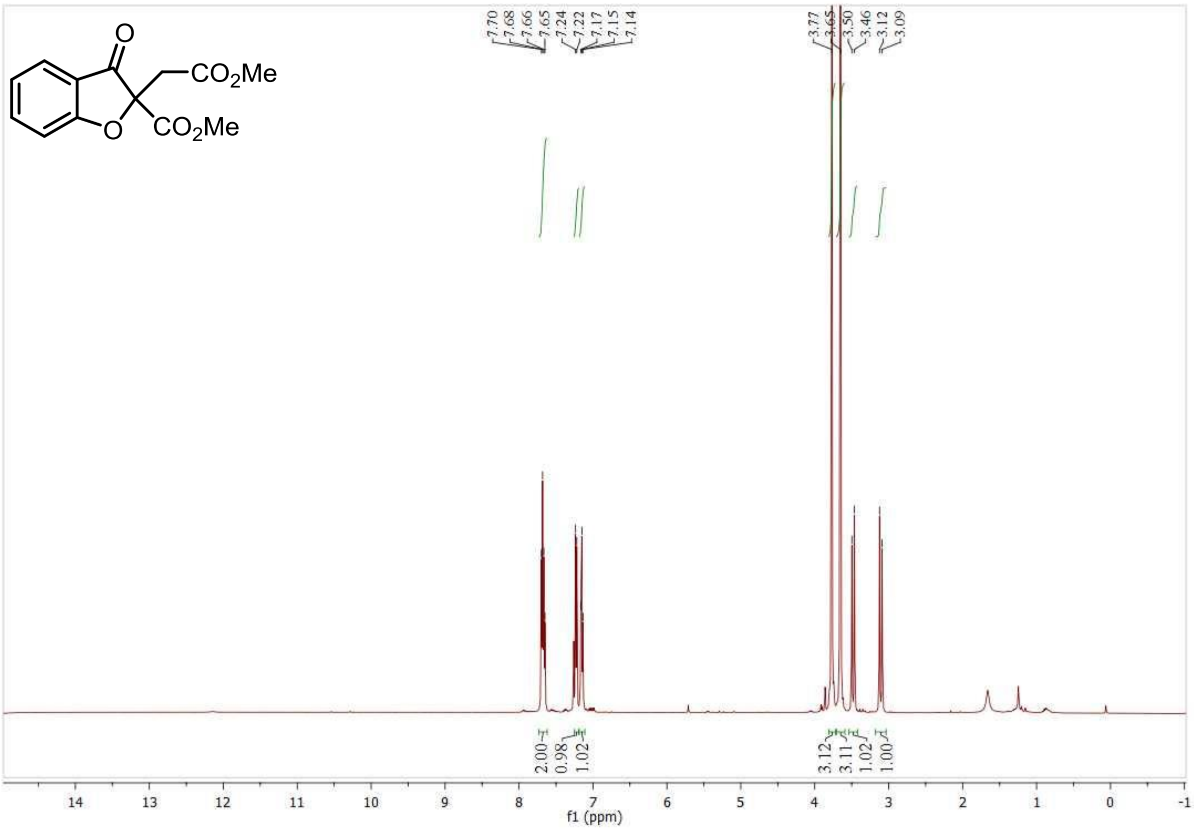

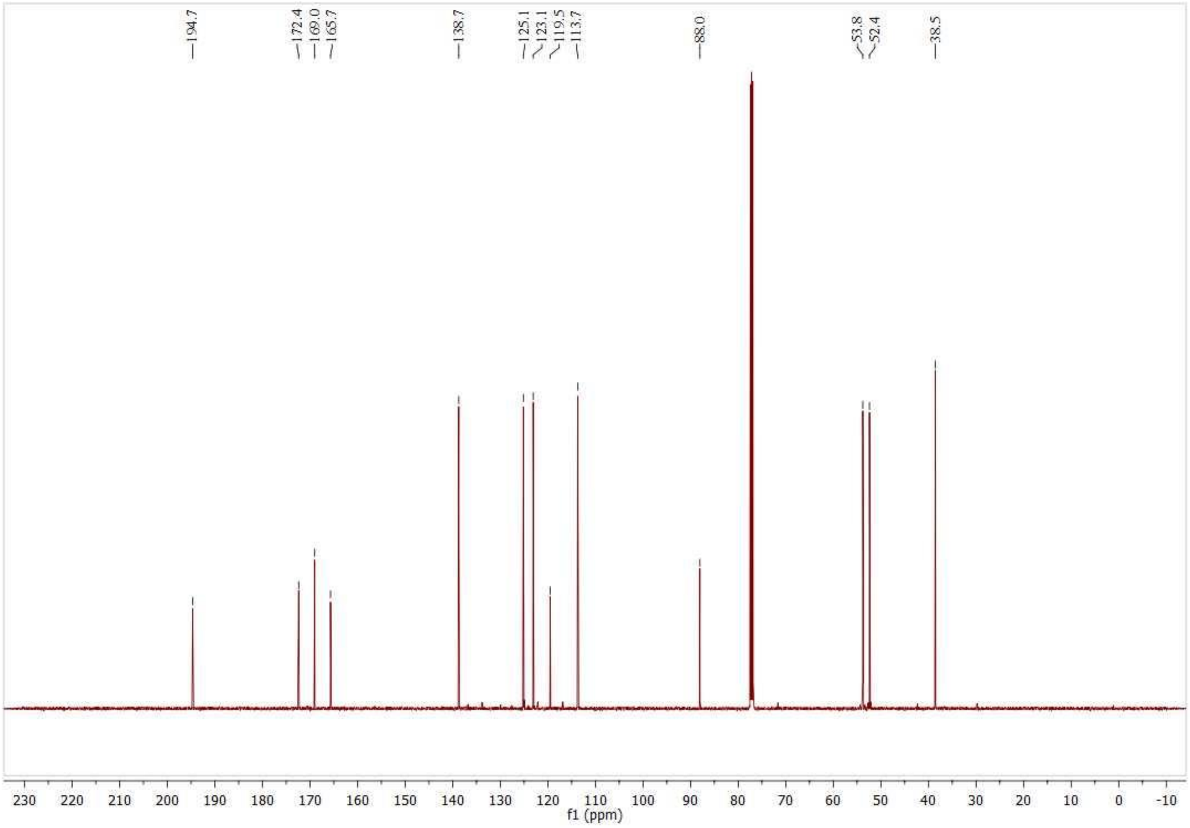

methyl 2-(2-methoxy-2-oxoethyl)-5-methyl-3-oxo-2,3-dihydrobenzofuran-2-carboxylate (33)

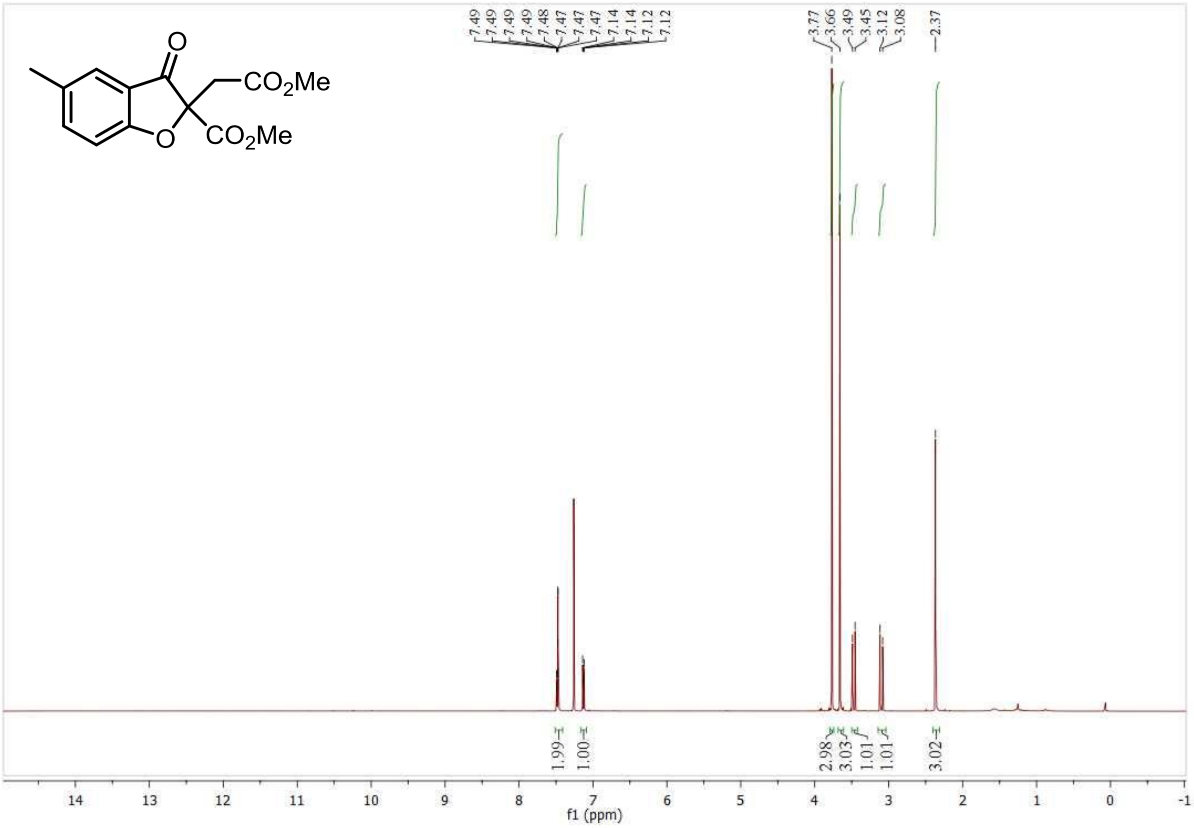

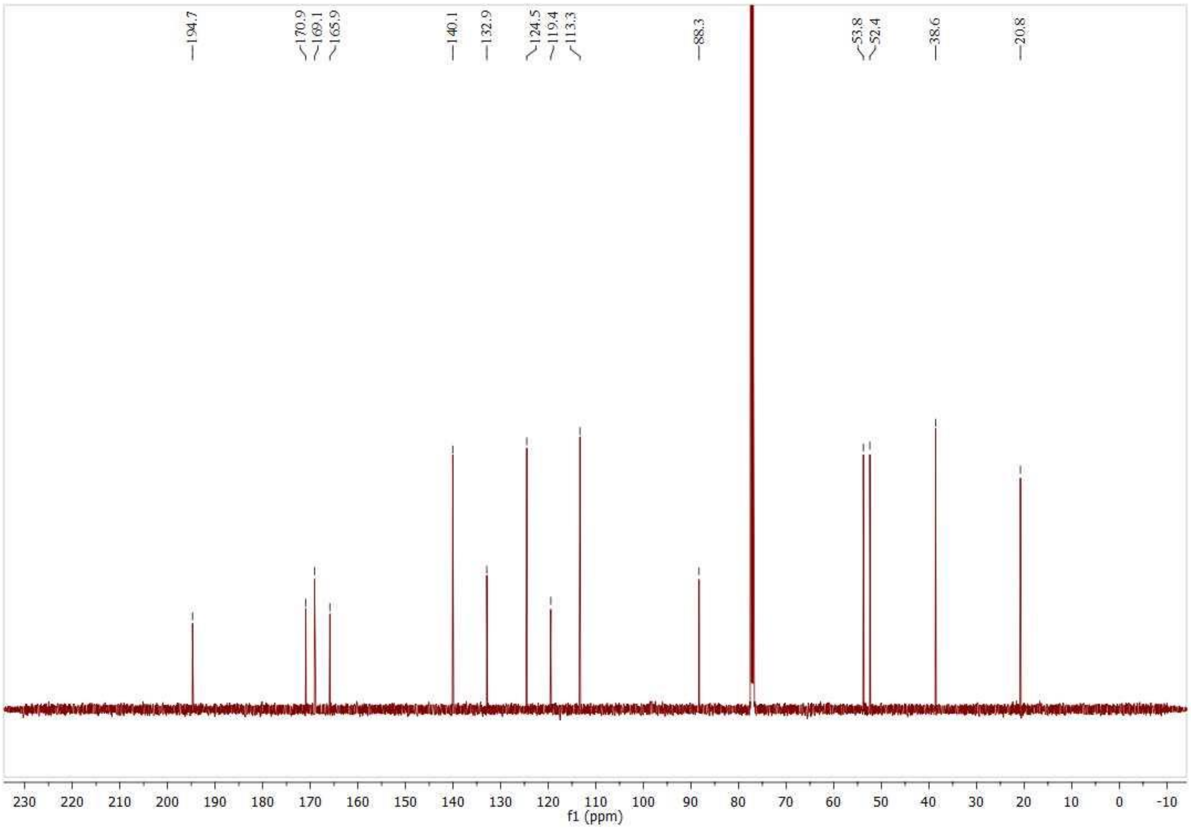

methyl 5-bromo-2-(2-methoxy-2-oxoethyl)-3-oxo-2,3-dihydrobenzofuran-2-carboxylate (34)

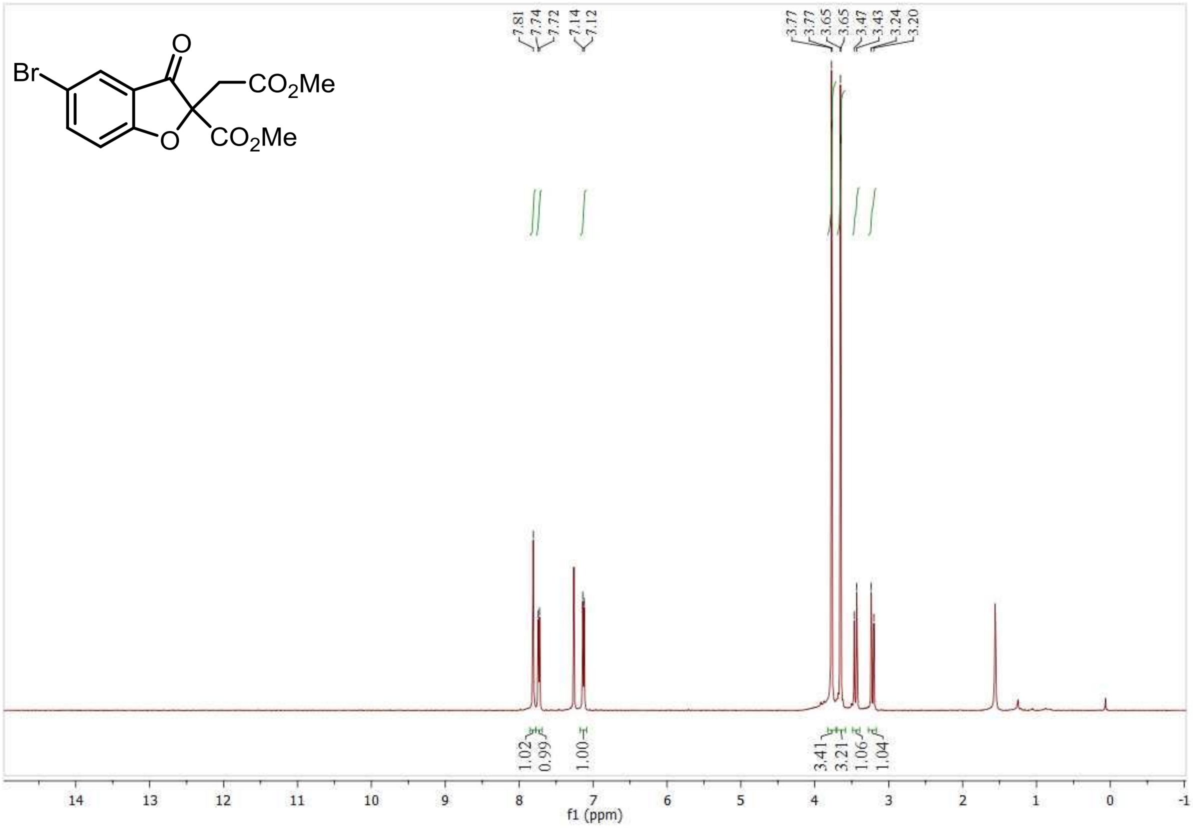

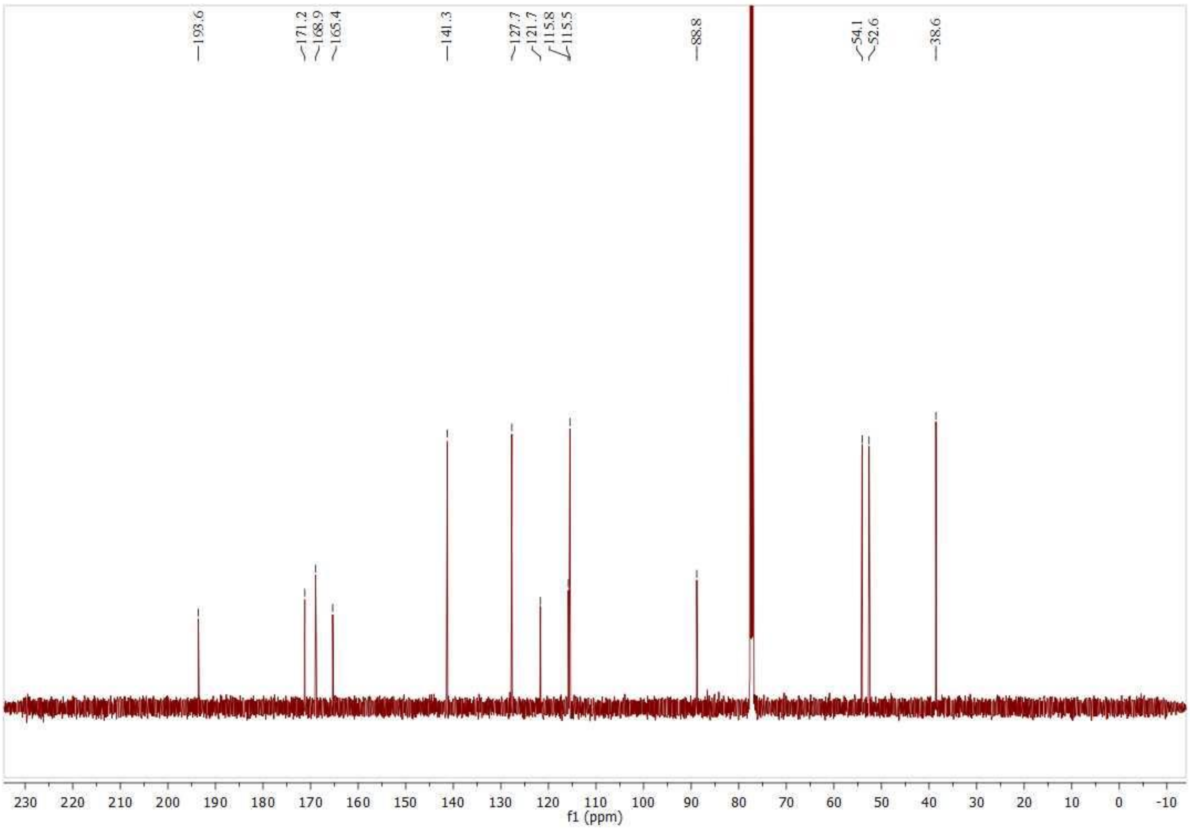

(E)-1-phenyl-3-(p-tolyl)prop-2-en-1-one (23a)

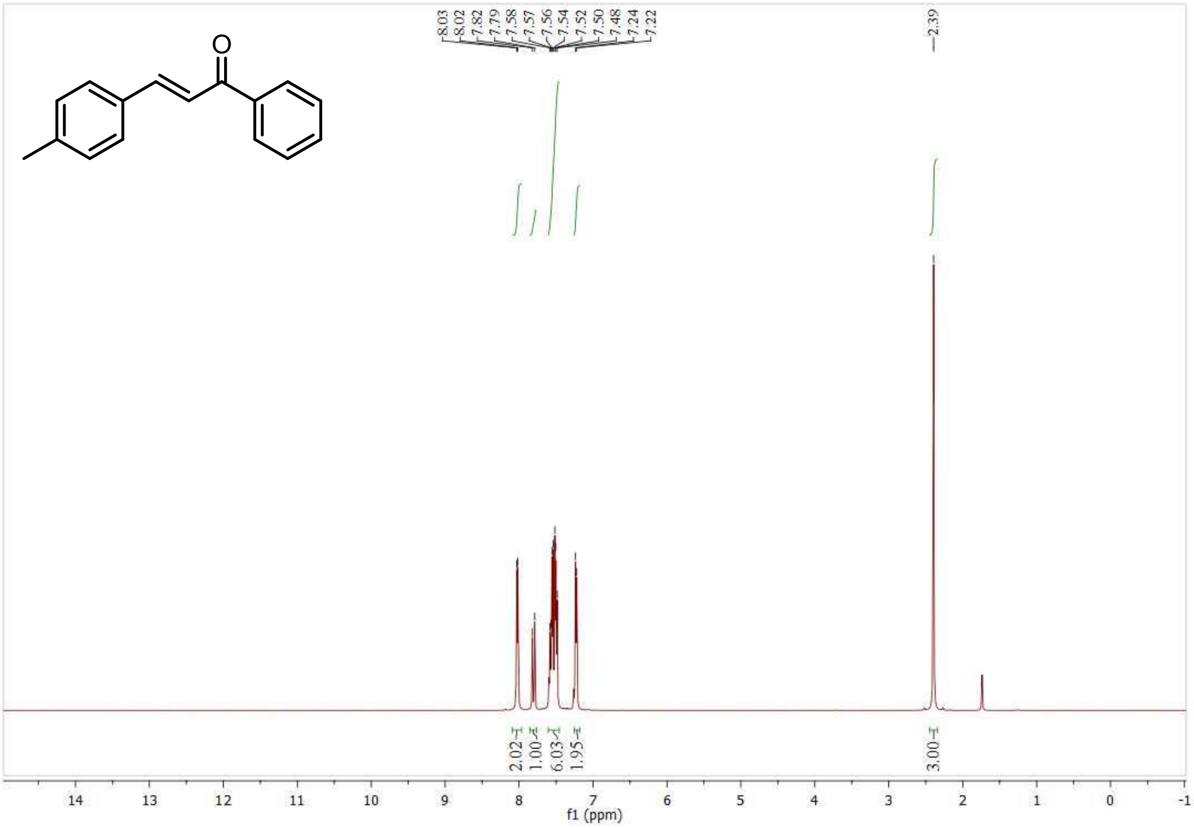

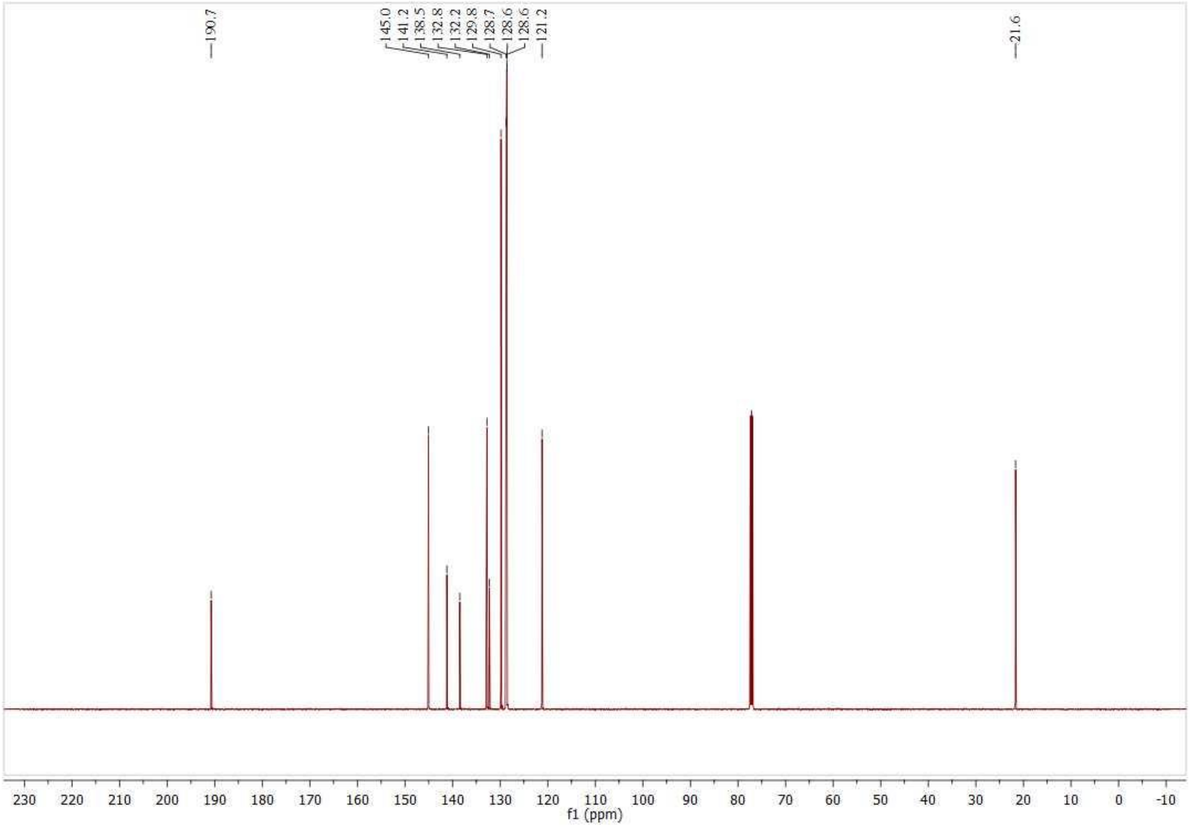

(E)-3-(4-chlorophenyl)-1-phenylprop-2-en-1-one (26a)

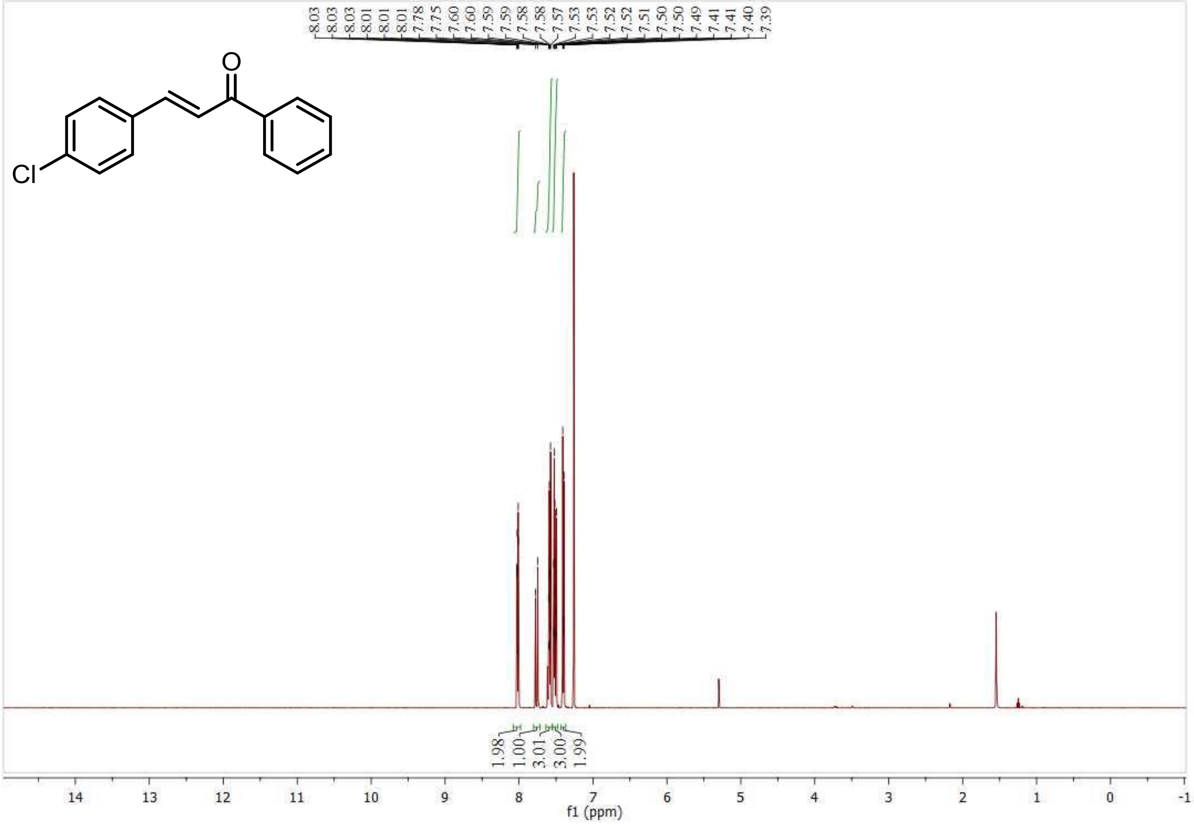

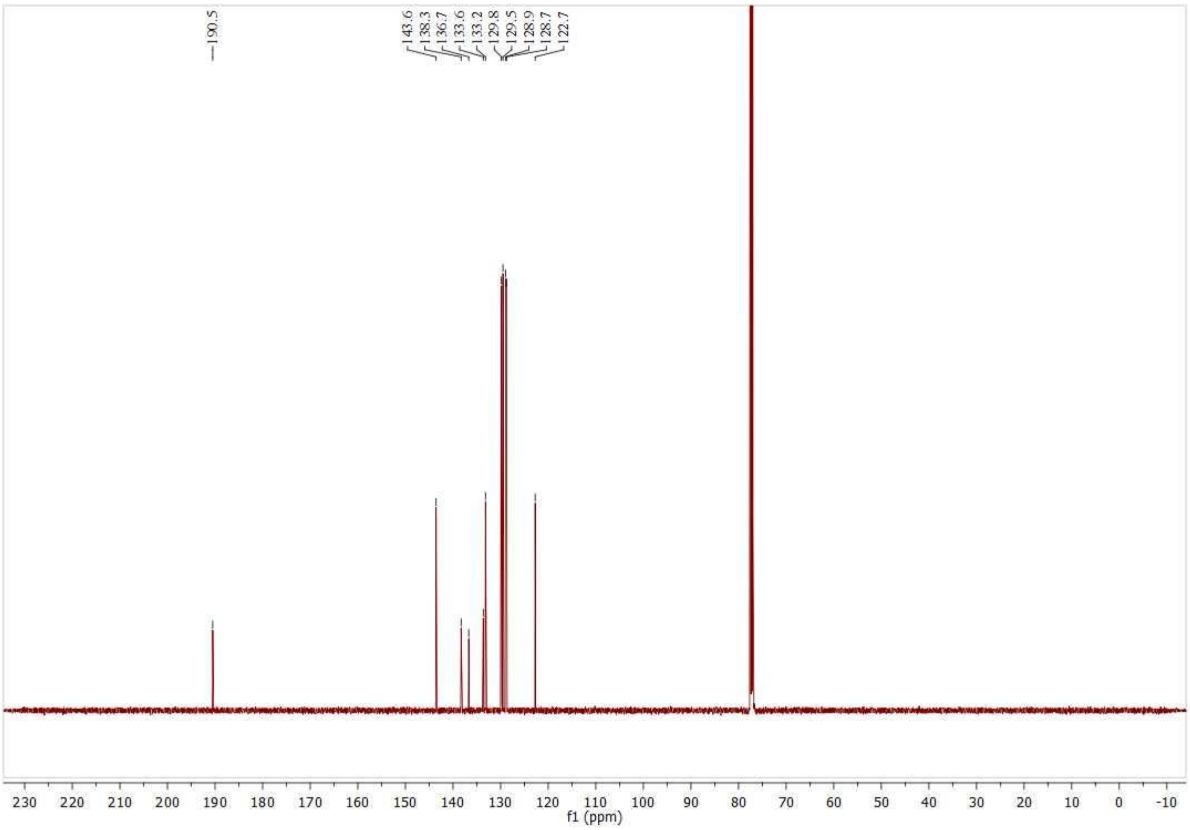

1,2,4-triphenylbutane-1,4-dione (20)

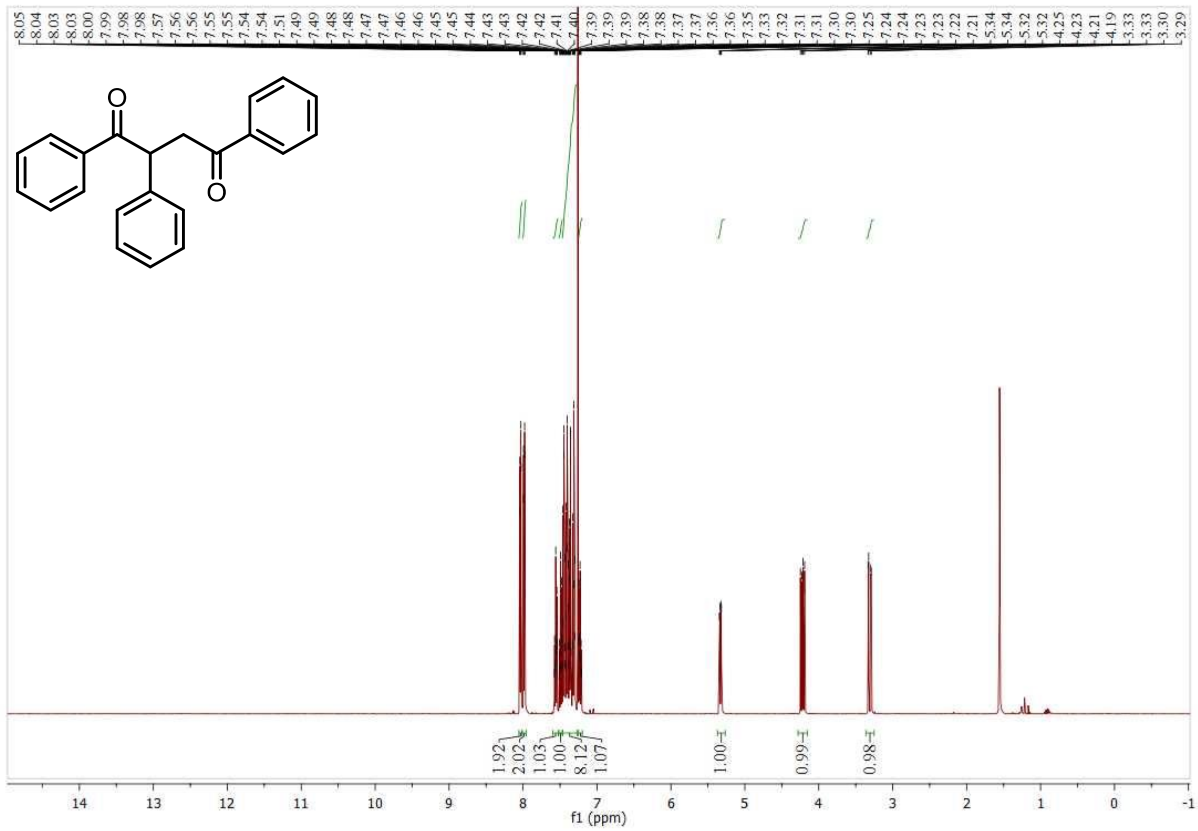

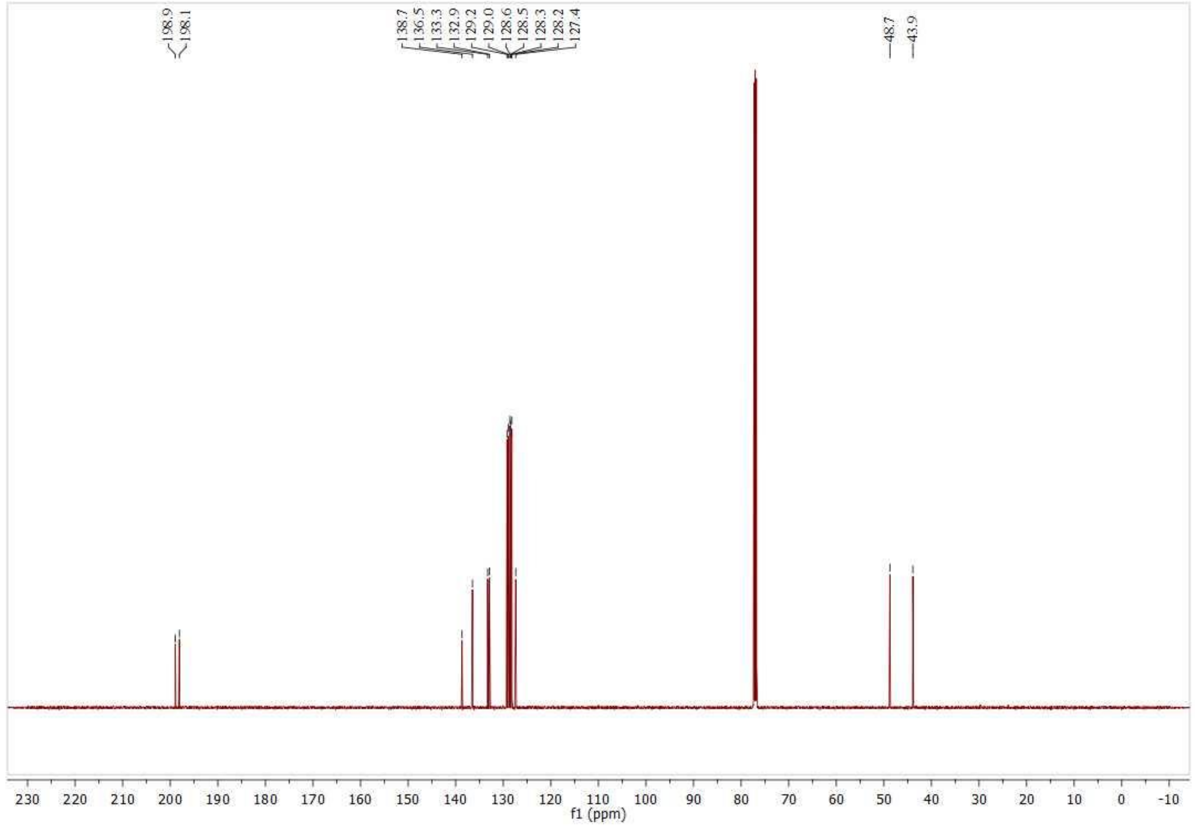

1-(4-chlorophenyl)-2,4-diphenylbutane-1,4-dione (21)

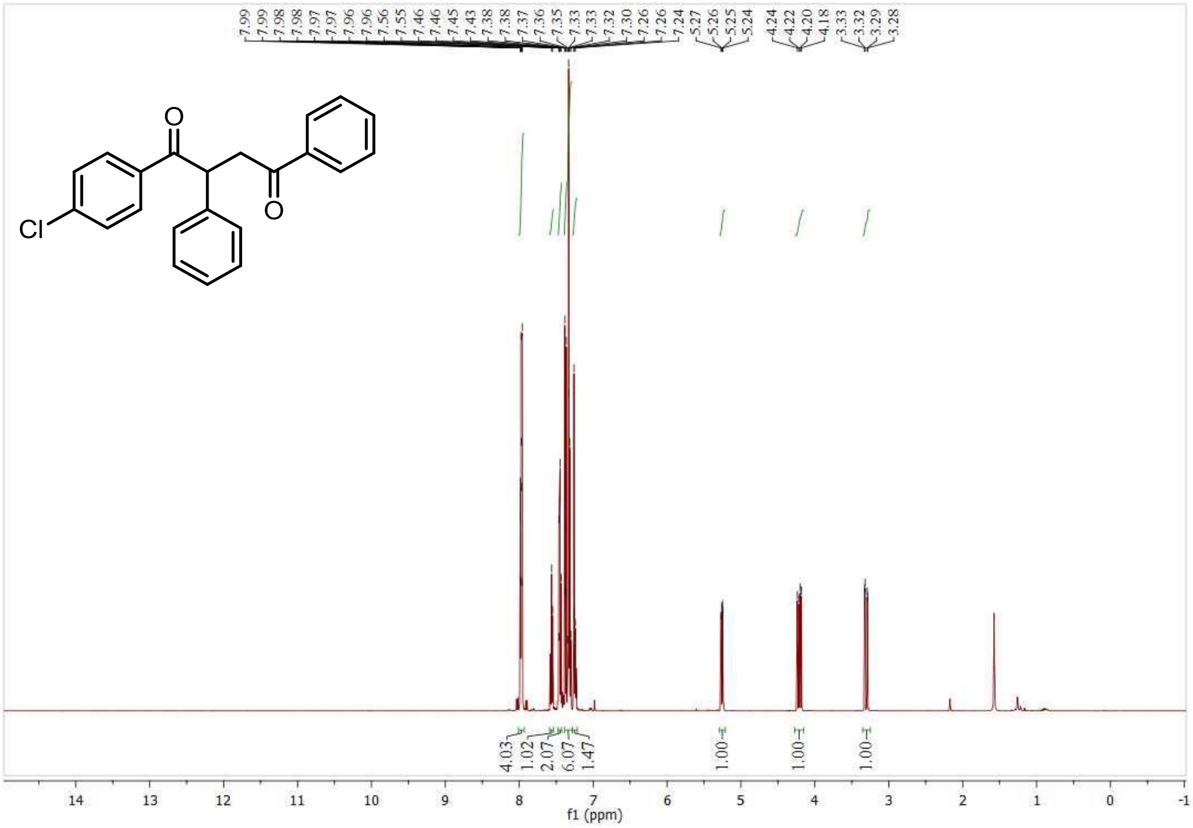

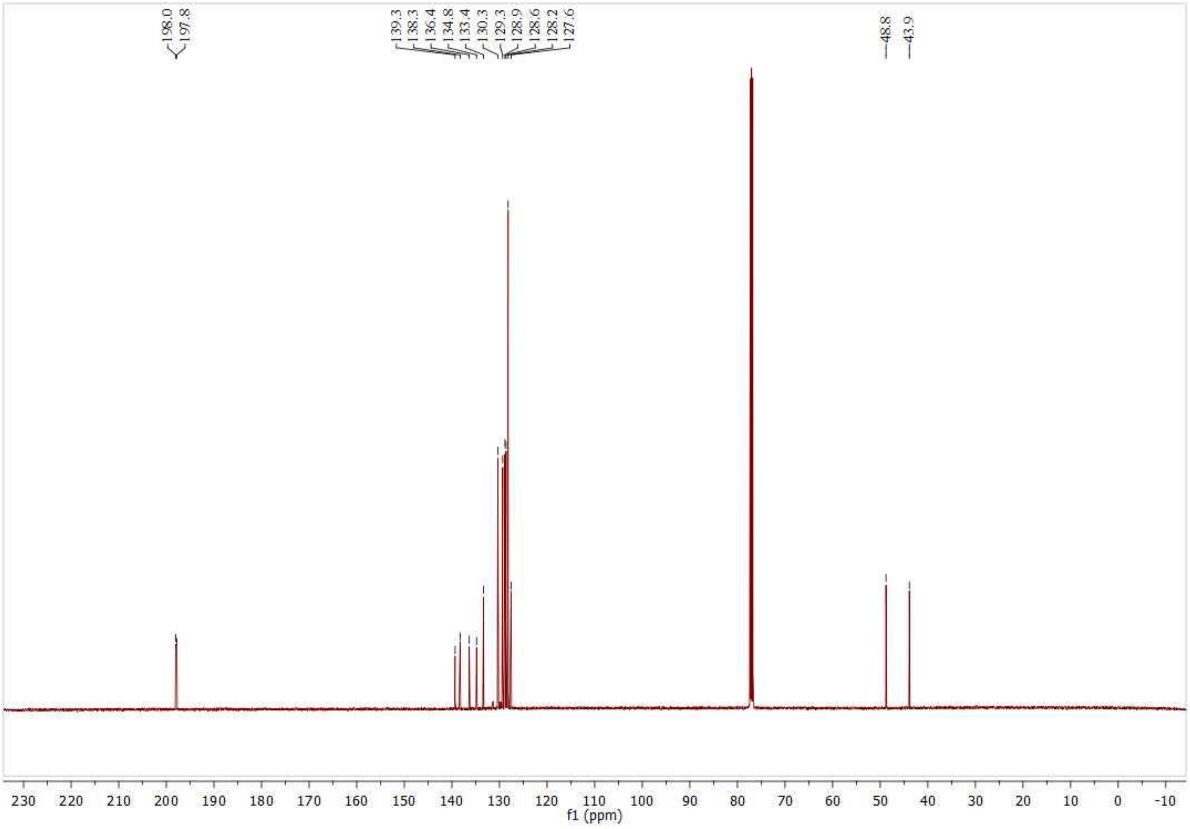

2,4-diphenyl-1-(p-tolyl)butane-1,4-dione (22)

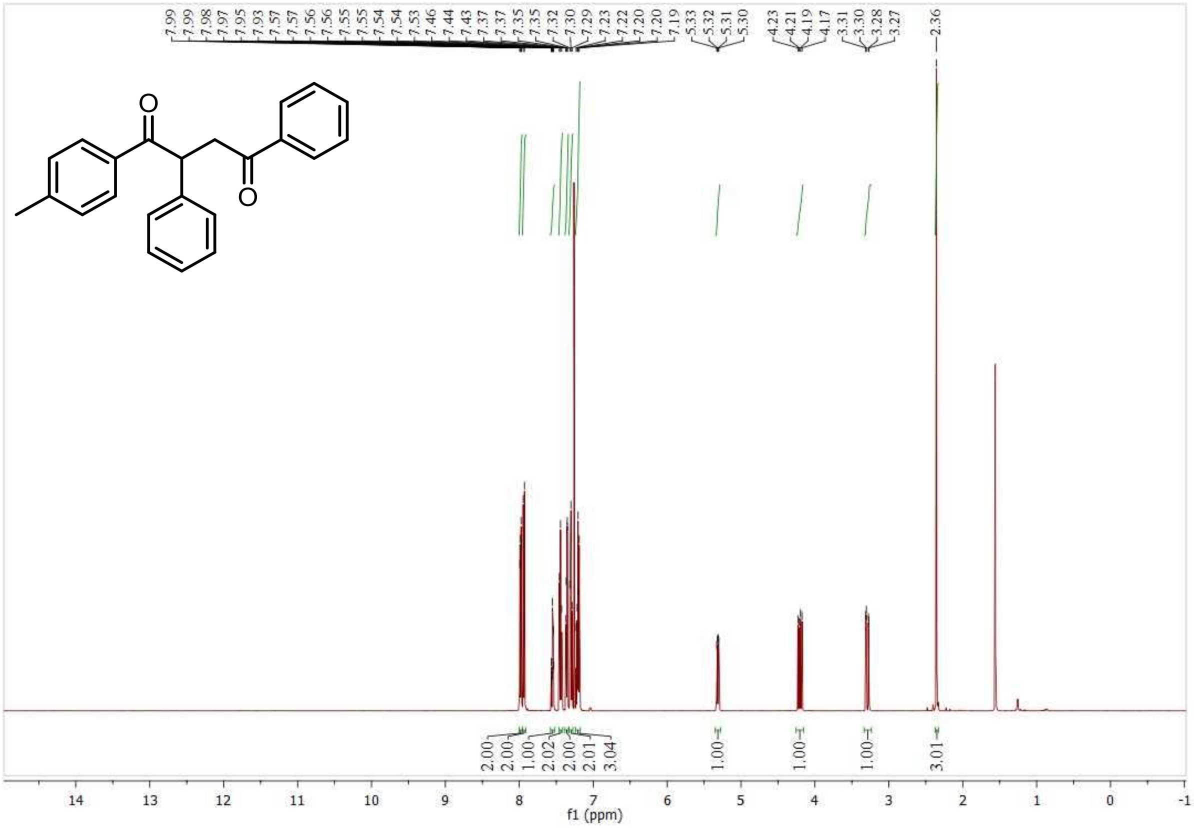

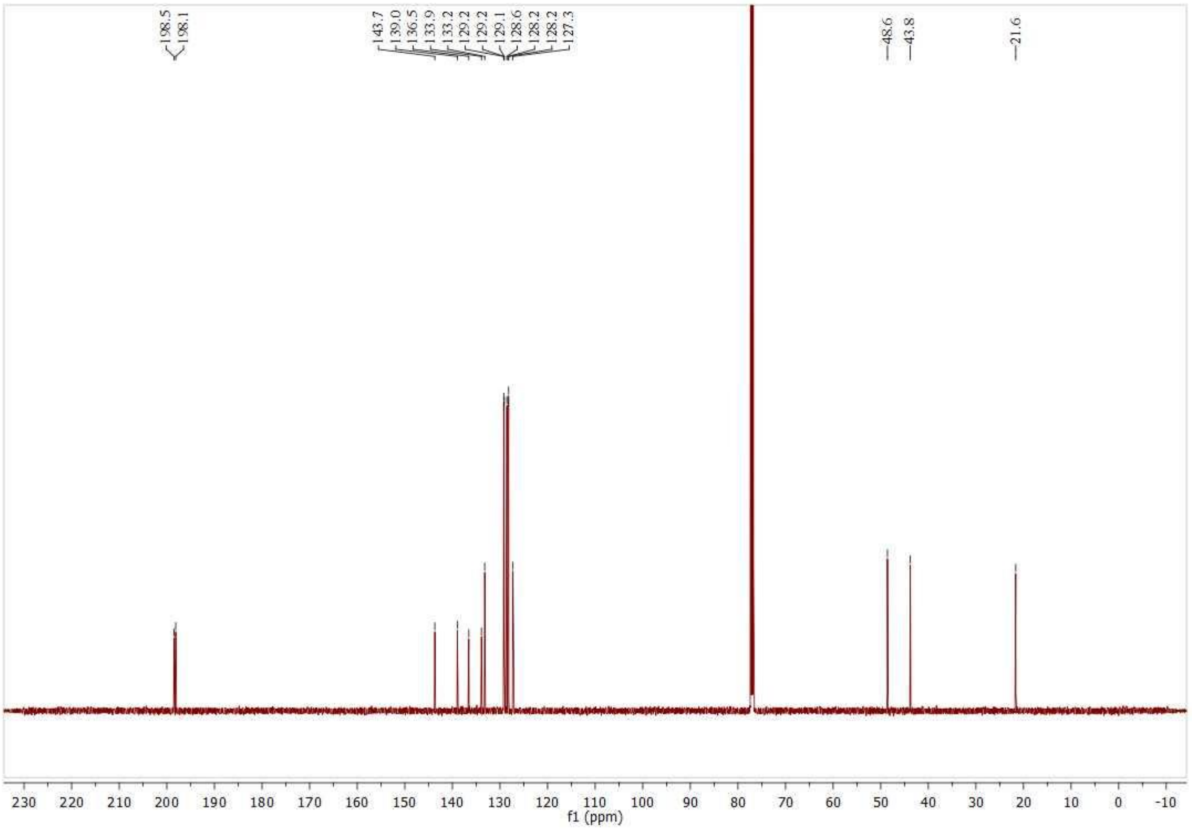

1,4-diphenyl-2-(p-tolyl)butane-1,4-dione (23)

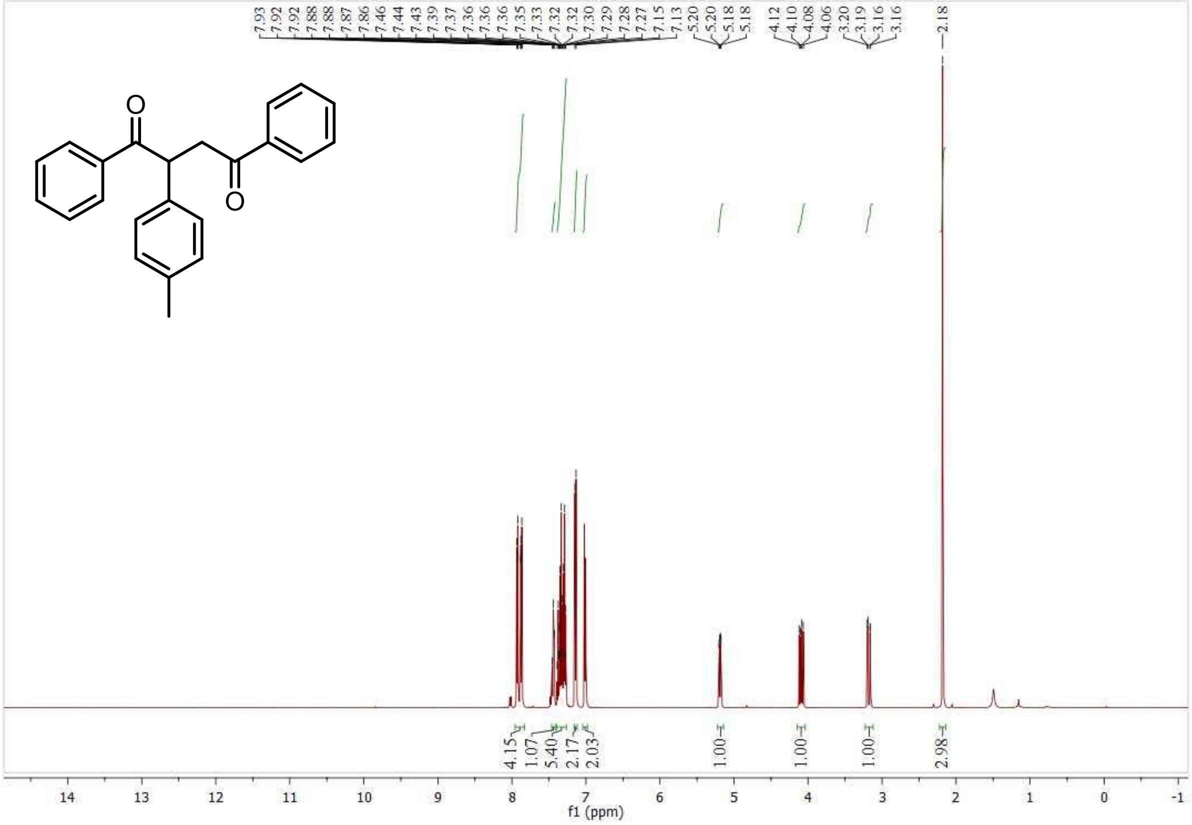

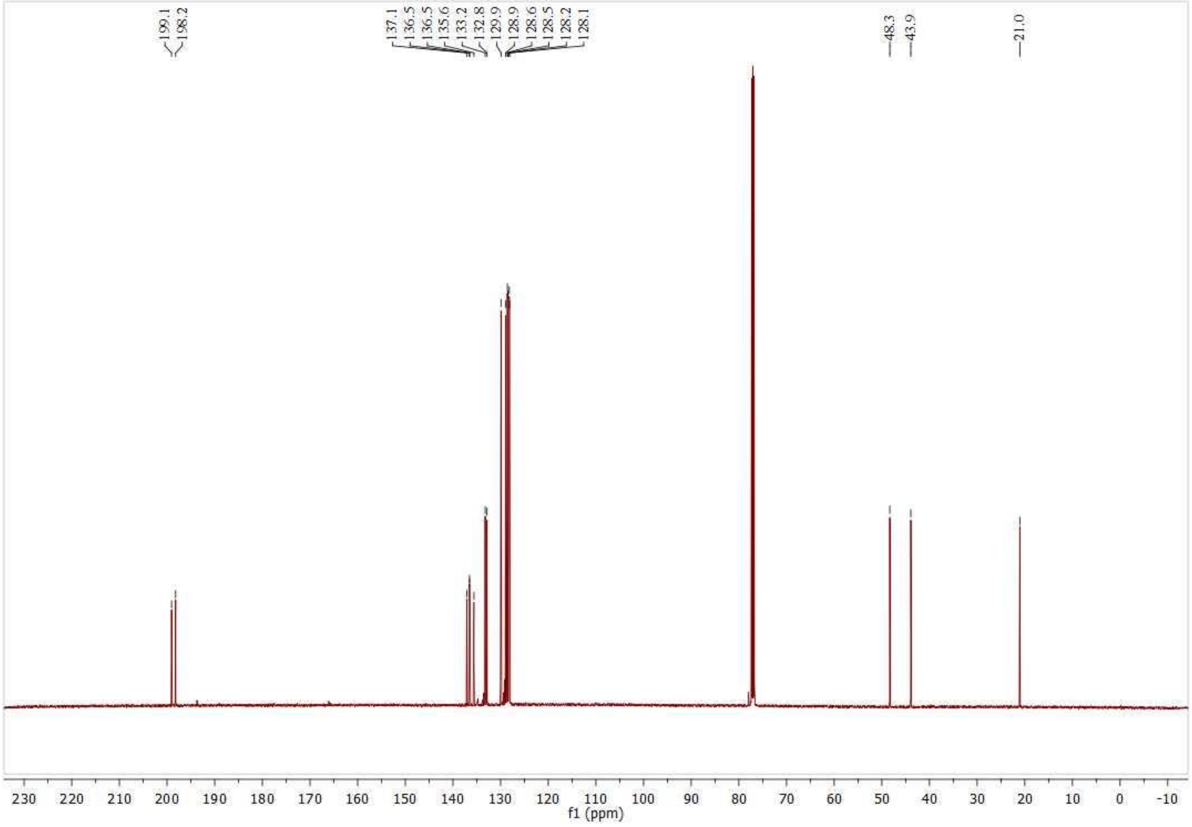

1-(4-chlorophenyl)-4-phenyl-2-(p-tolyl)butane-1,4-dione (24)

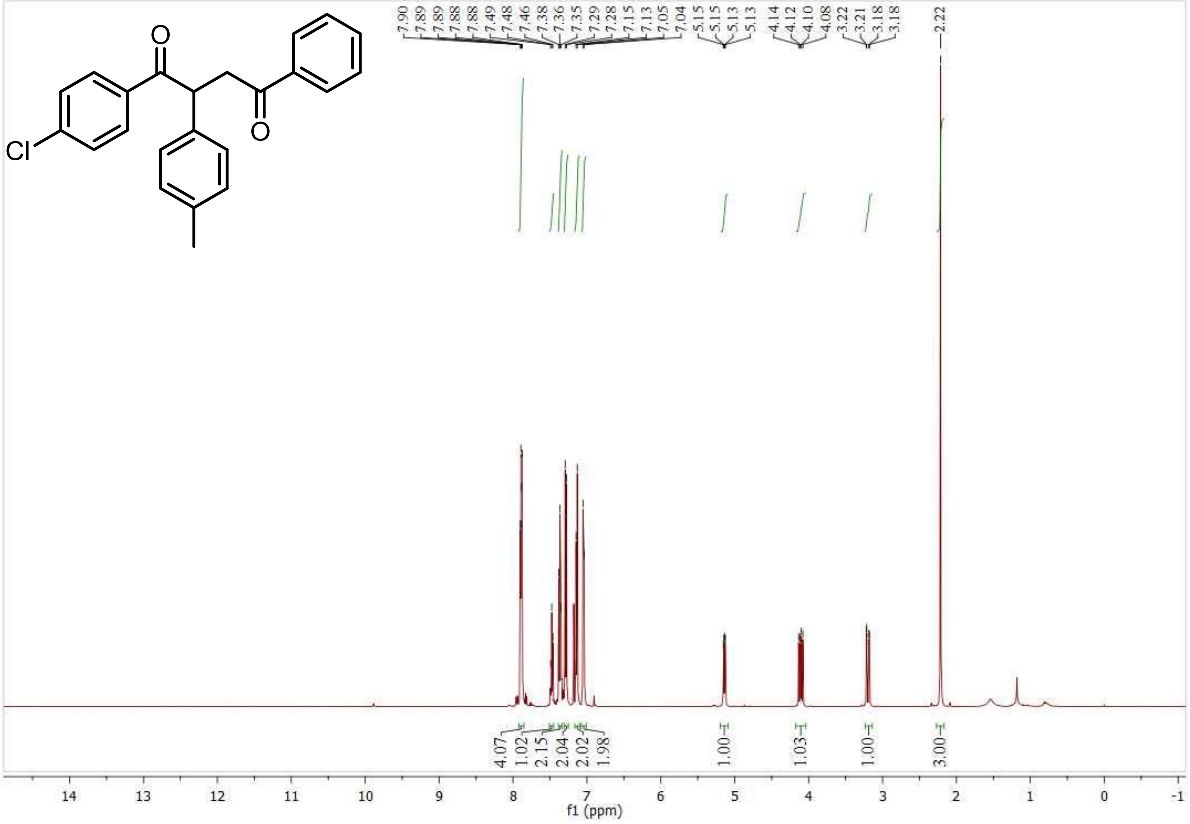

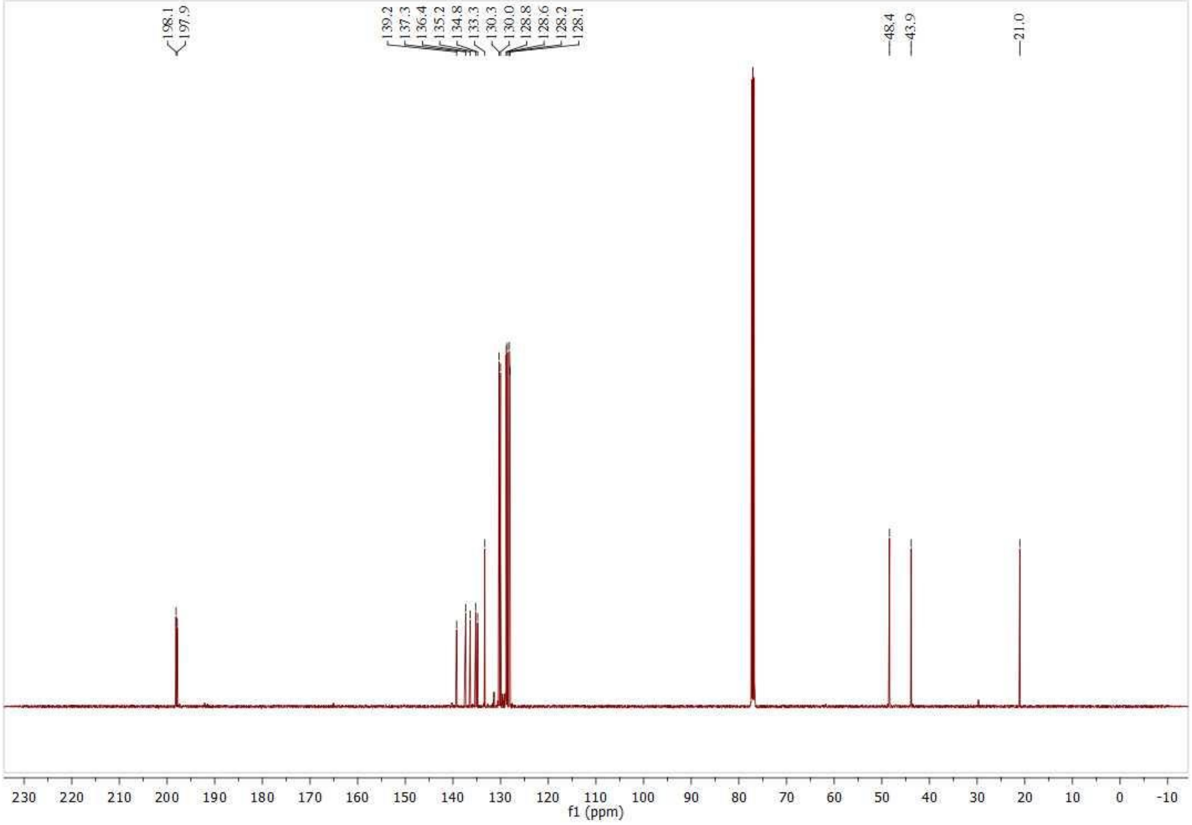

4-phenyl-1,2-di-p-tolylbutane-1,4-dione (25)

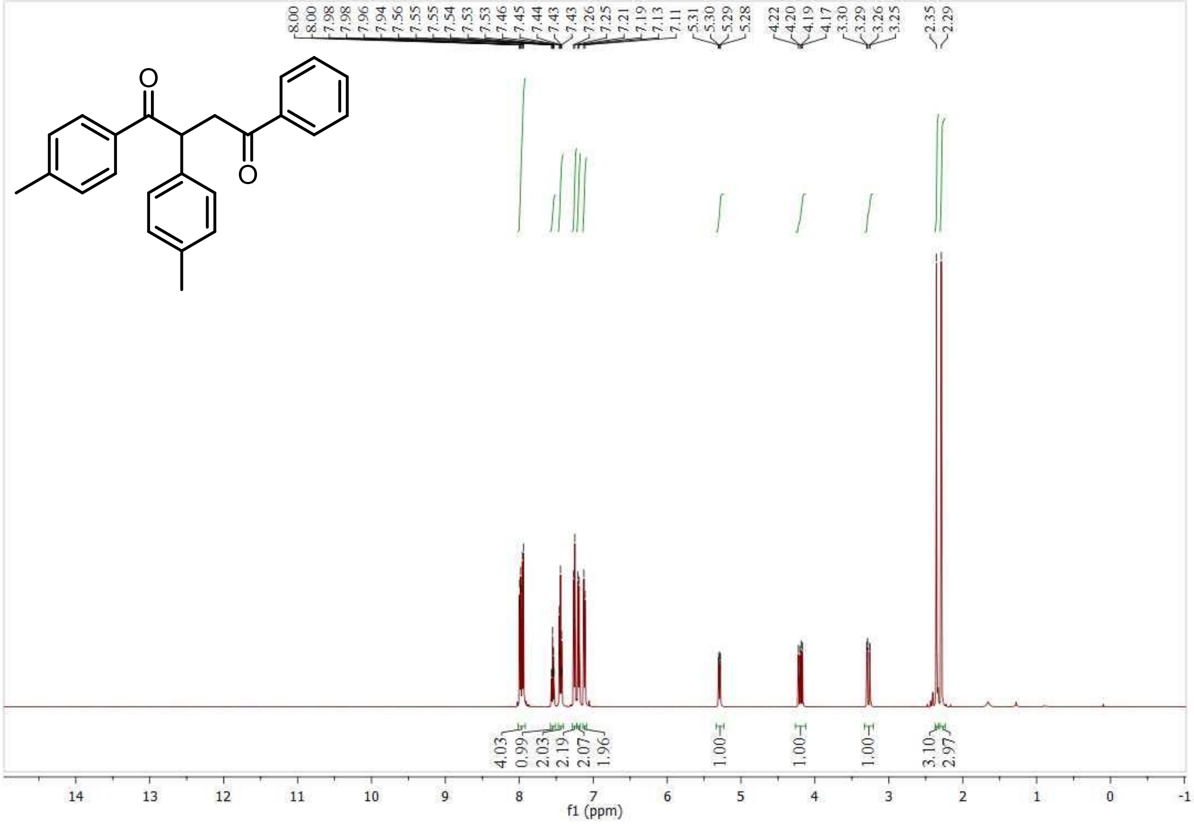

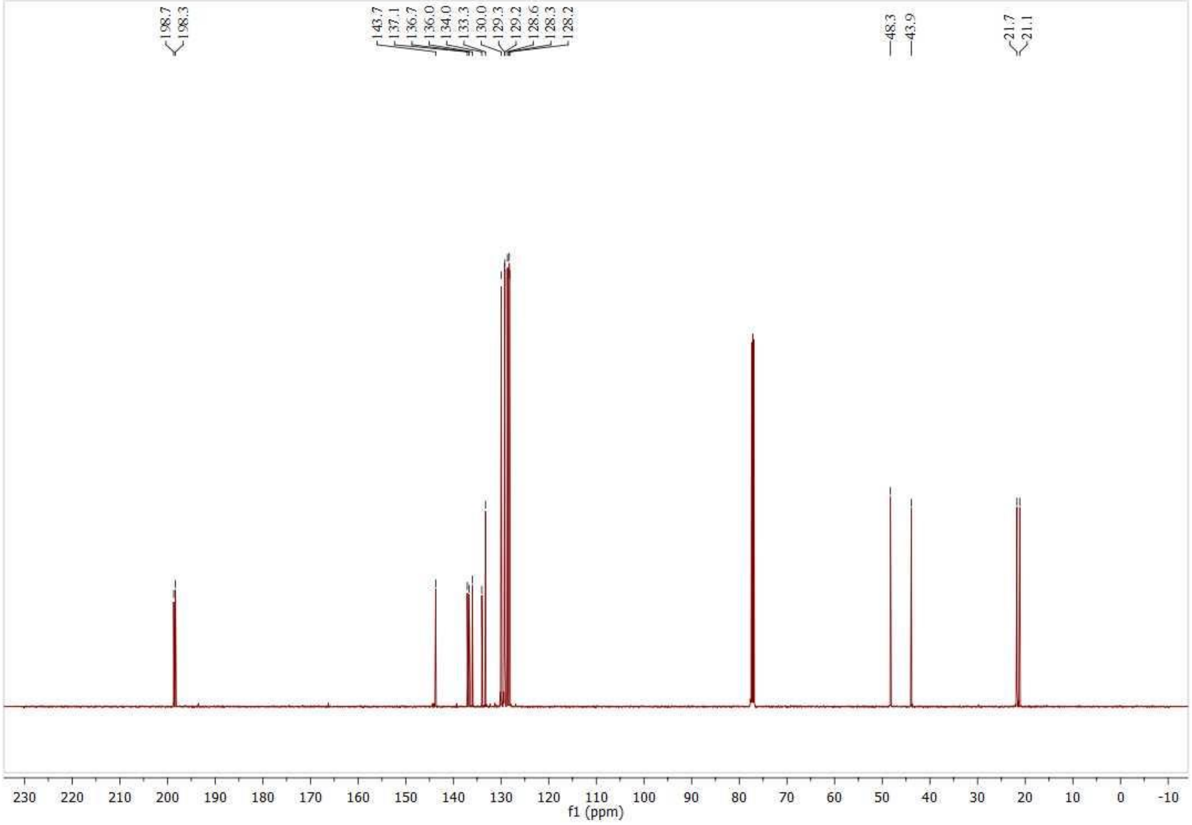

2-(4-chlorophenyl)-1,4-diphenylbutane-1,4-dione (26)

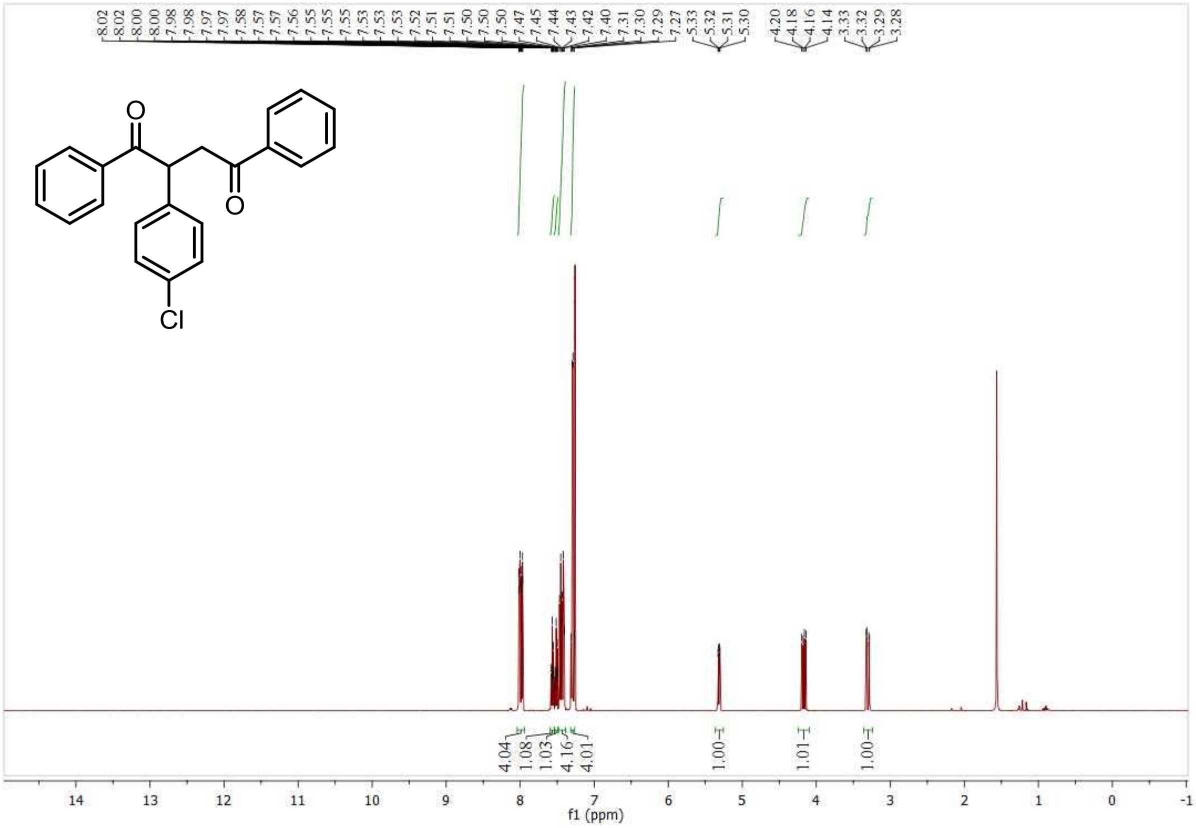

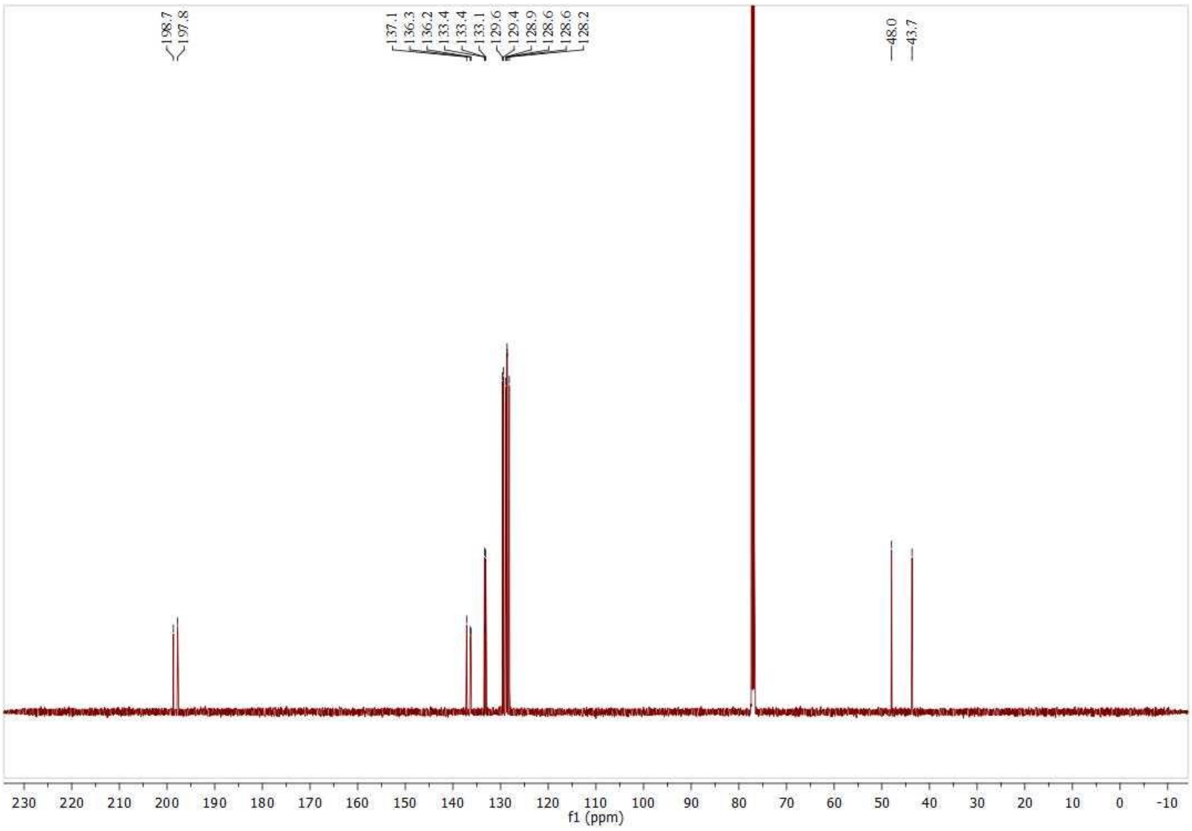

1,2-bis(4-chlorophenyl)-4-phenylbutane-1,4-dione (27)

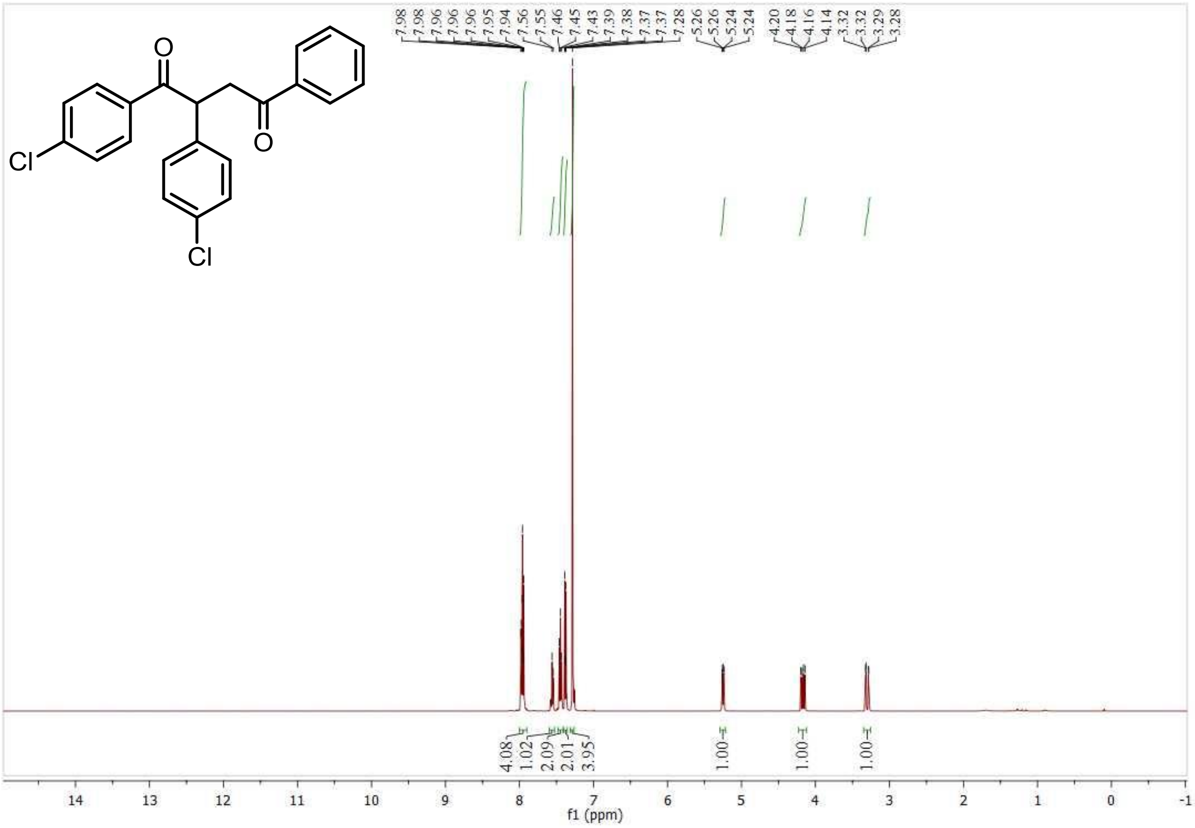

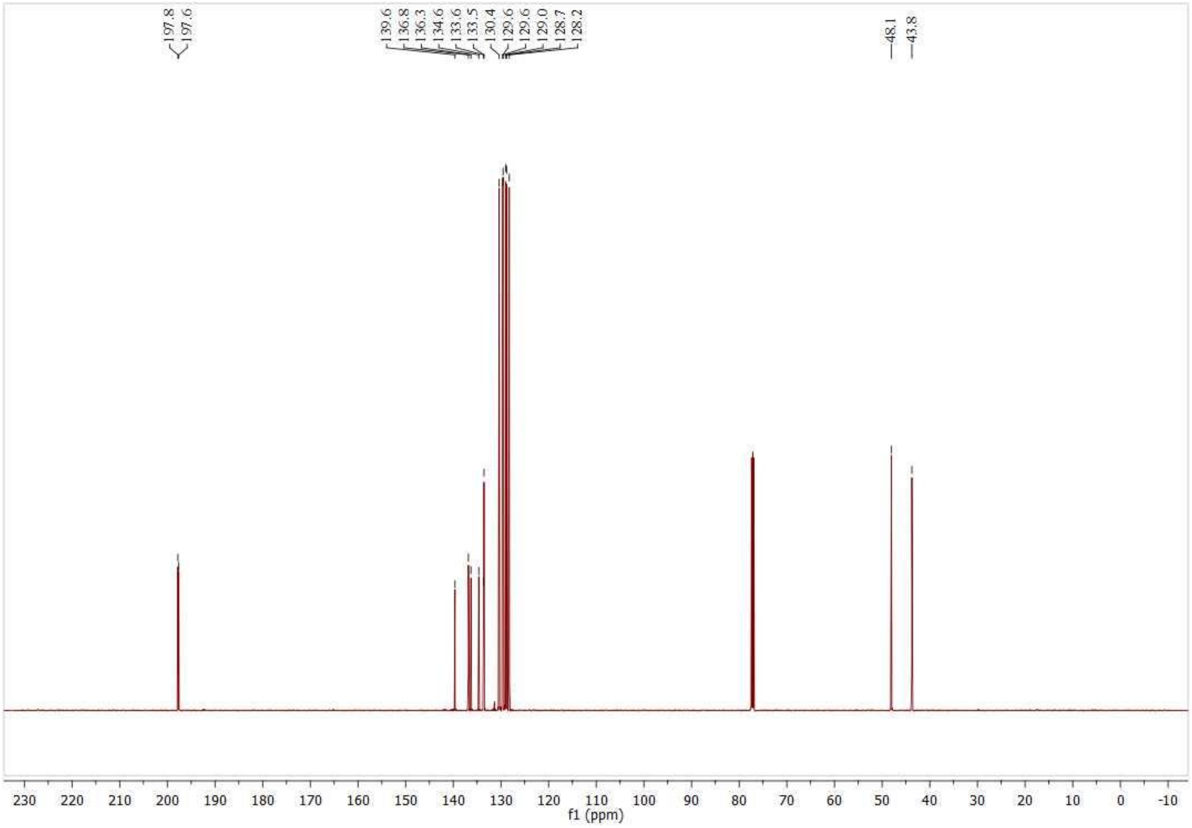

2-(4-chlorophenyl)-4-phenyl-1-(p-tolyl)butane-1,4-dione (28)

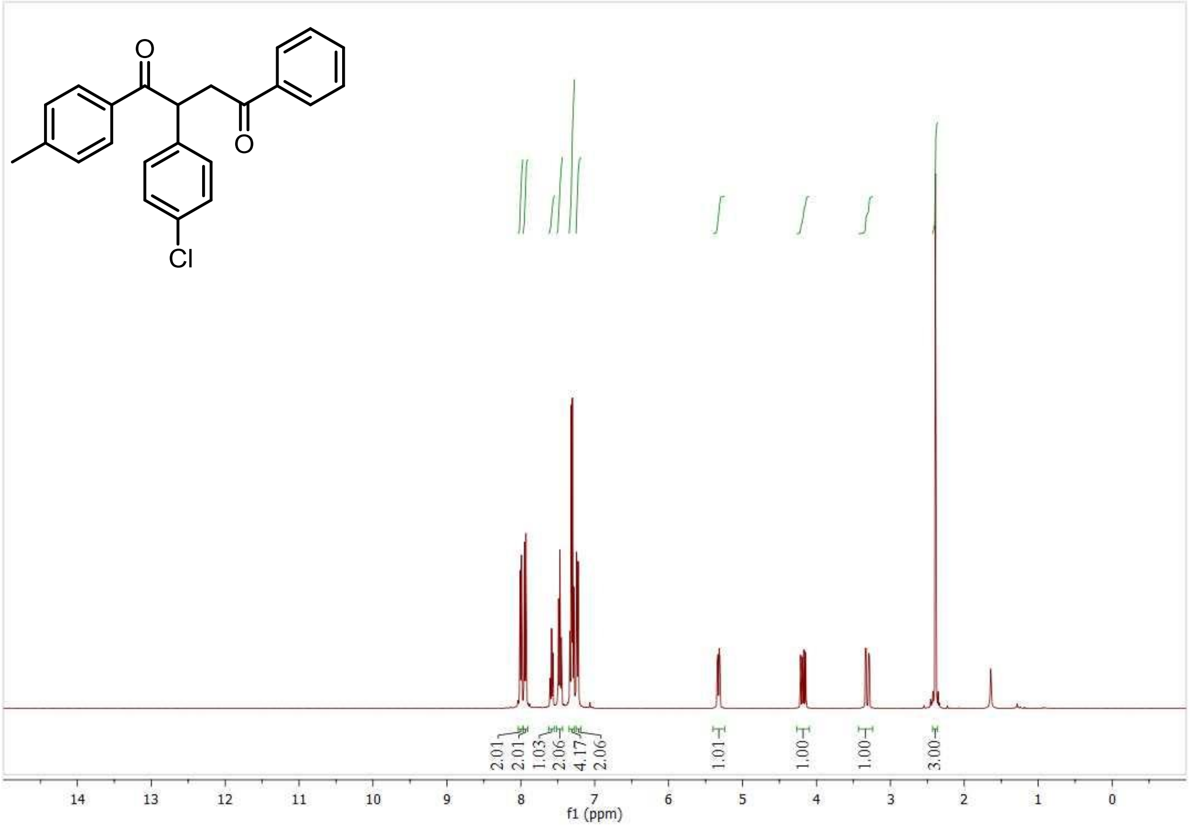

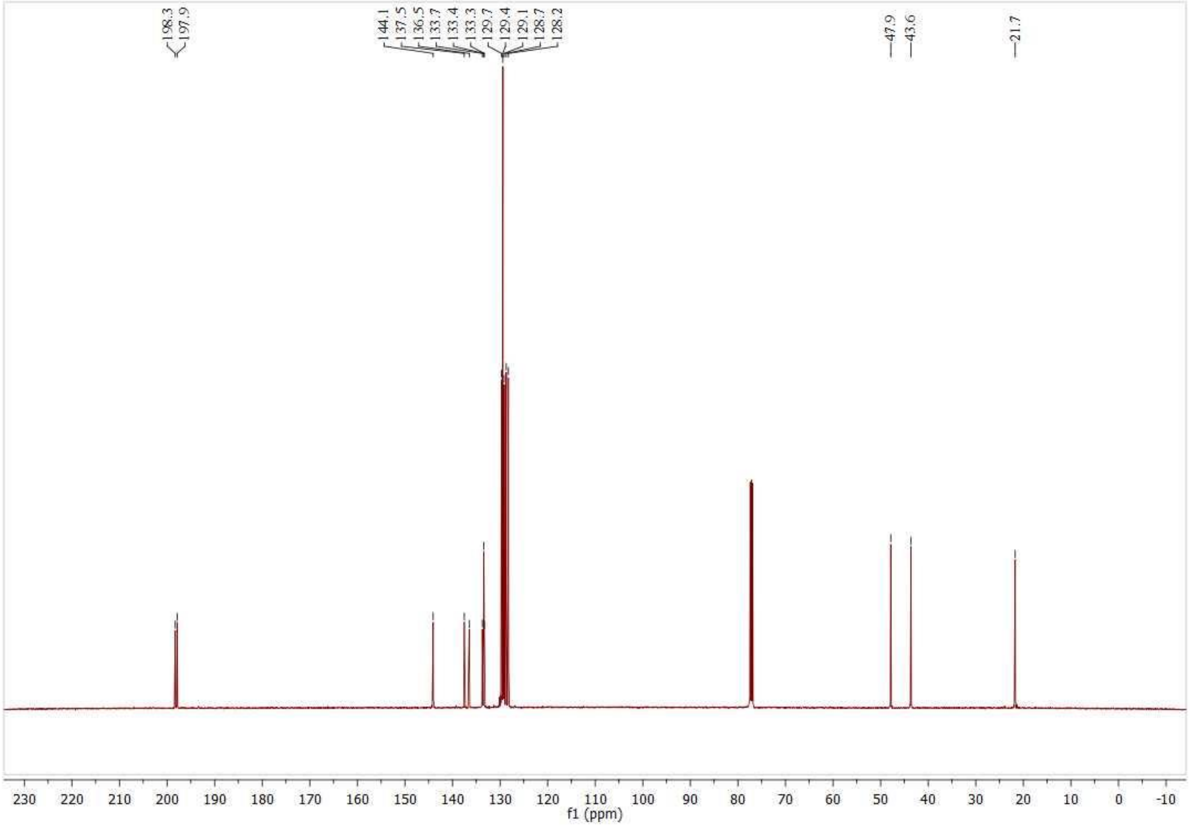

Supplement: Supplementary file 1 — Supplementary [file CSSC-13-131-s001.pdf]
